# Photoredox-mediated Minisci C–H alkylation of *N*-heteroarenes using boronic acids and hypervalent iodine†
†Electronic supplementary information (ESI) available. See DOI: 10.1039/c6sc02653b



**DOI:** 10.1039/c6sc02653b

**Published:** 2016-07-12

**Authors:** Guo-Xing Li, Christian A. Morales-Rivera, Yaxin Wang, Fang Gao, Gang He, Peng Liu, Gong Chen

**Affiliations:** a State Key Laboratory and Institute of Elemento-Organic Chemistry , Collaborative Innovation Center of Chemical Science and Engineering (Tianjin) , Nankai University , Tianjin 300071 , China . Email: gongchen@nankai.edu.cn; b Department of Chemistry , University of Pittsburgh , Pittsburgh , PA 15260 , USA . Email: pengliu@pitt.edu; c Department of Chemistry , The Pennsylvania State University , 104 Chemistry Building , University Park , PA 16802 , USA . Email: guc11@psu.edu

## Abstract

A photoredox-mediated Minisci C–H alkylation reaction of *N*-heteroarenes with alkyl boronic acids is reported. A broad range of primary and secondary alkyl groups can be efficiently incorporated into various *N*-heteroarenes using [Ru(bpy)_3_]Cl_2_ as photocatalyst and acetoxybenziodoxole as oxidant under mild conditions. The reaction exhibits excellent substrate scope and functional group tolerance, and offers a broadly applicable method for late-stage functionalization of complex substrates. Mechanistic experiments and computational studies suggest that an intramolecularly stabilized *ortho*-iodobenzoyloxy radical intermediate might play a key role in this reaction system.

## Abstract 2

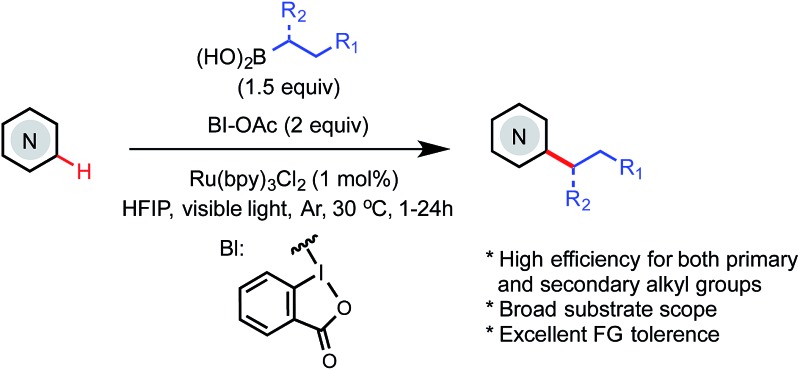
A photoredox-mediated Minisci C–H alkylation of *N*-heteroarenes with easily accessible primary and secondary alkyl boronic acids has been developed.

## Introduction

**Scheme 1:**
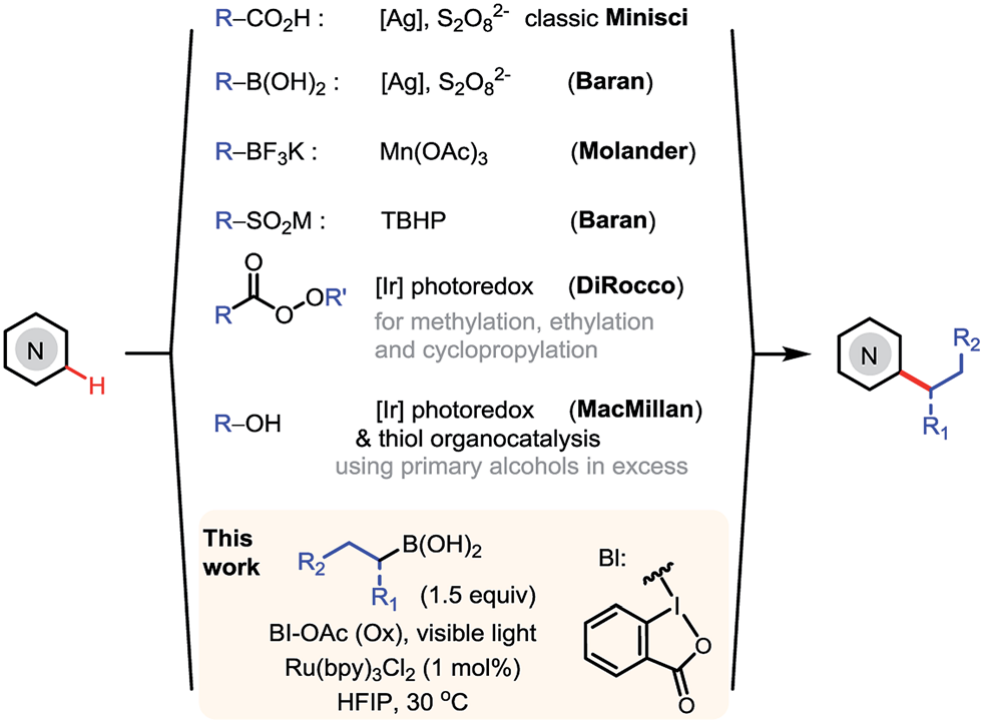
Minisci C–H alkylation of *N*-heteroarenes.

*N*-Heteroarenes are common structural motifs in natural products, drug molecules, organic materials and ligands for metal catalysts.^1^ Synthetic methods which enable the selective functionalization of the C–H bonds of *N*-heteroarenes could greatly facilitate their applications in these areas.^2^ Among the different types of C–H functionalizations, C–H alkylations could provide more stereochemically diverse modifications.^3^ Over the past few years, the C–H functionalization of electron-deficient heteroarenes *via* addition of carbon-centered radicals under oxidative conditions, known as the Minisci reaction, has undergone a remarkable renaissance, offering increasingly powerful methods for synthesizing alkyl-substituted heteroarenes (Scheme 1).^4^ While the classical Minisci alkylation reaction involves alkyl carboxylic acids and halides, Baran recently demonstrated that aryl boronic acids are also viable reagents in Minisci-type C–H arylation reactions using Ag(i)/S_2_O_8_^2–^ oxidant.^5^ Molander demonstrated that alkyl trifluoroborates, particularly secondary alkyl trifluoroborates, can effect efficient Minisci alkylation using Mn(OAc)_3_ oxidant.^6^ In addition, Minisci C–H alkylation transformations have been achieved using a variety of other alkylating reagents, including sulfinates, aldehydes, and even simple alkanes, using different radical initiators and oxidants.^7^

More recently, DiRocco reported the first photoredox-mediated Minisci alkylation reaction of *N*-heteroarenes using peroxides as the alkylating reagent.^8^ MacMillan demonstrated a Minisci alkylation reaction of *N*-heteroarenes using primary alcohols as the alkylation reagent, *via* photoredox- and organo- catalysis.^9*a*^ However, despite these significant advances, practical and broadly applicable methods for Minisci C–H alkylation of *N*-heteroarenes capable of coupling complex alkyl groups are still lacking. Herein, we report a photoredox-mediated Minisci C–H alkylation reaction of *N*-heteroarenes with a variety of easily accessible primary and secondary alkyl boronic acids. Its high efficiency, broad substrate scope, excellent functional group tolerance, and mild operation conditions make it particularly suitable for late-stage functionalization of complex substrates such as drug molecules.

## Results and discussion

Although alkyl boron reagents are readily available and are well-known precursors for alkyl radicals, they have been rarely applied in photoredox-mediated C–C coupling reactions.^10–13^ In 2012, Akita reported that alkyl trifluoroborates or cyclic triolborates can couple with 2,2,6,6-tetramethylpiperidinyl-1-oxy (TEMPO) or Michael acceptors under Ru or Ir photoredox catalysis.^11^ In 2015, Chen reported a decarboxylative alkenylation of alkyl trifluoroborates with vinyl carboxylic acids using a hypervalent iodine oxidant, acetoxybenziodoxole (BI-OAc), under Ru photoredox catalysis.^12*a*^ More recently, Molander achieved coupling of alkyl trifluoroborates with aryl halides by merging photoredox with Ni cross-coupling catalysis.^13^ During our recent investigation of radical-mediated sp^3^ C–H azidation reactions, we discovered that azidobenziodoxole (Bl-N_3_) can be readily activated by visible light in the presence of [Ru(bpy)_3_]Cl_2_, initiating a radical chain reaction.^14^ Intrigued by the unique radical reactivity of benziodoxole reagents with photocatalysts, we questioned whether they can facilitate Minisci C–H alkylation with alkyl boron reagents under photoredox-mediated conditions.^15–17^

We commenced our investigation with C–H butylation of 4-chloroquinoline **1**, a common model substrate for Minisci reactions, using butyl boronic acid **2a** or trifluoroborate **2b** under the visible light (VL) irradiation (Table 1). We were delighted to find that the desired C2-alkylated product **3a** can be formed in excellent yield with **2a** using [Ru(bpy)_3_]Cl_2_ photocatalyst and BI-OAc oxidant under optimized conditions (entry 5). Alkylation with **2b** proceeded in lower yield (entry 6). In comparison with Bl-OAc, hydroxylbenziodoxole (BI-OH) gave slightly lower yield, methoxylbenziodoxole (Bl-OMe) was notably less effective, BI-N_3_ gave low yield, chlorobenziodoxole (Bl-Cl) and PhI(OAc)_2_ showed little reactivity (entries 7–11). Hexafluoroisopropanol (HFIP) solvent is critical for obtaining high yield (entries 3–5). No **3a** was formed in the absence of either Ru catalysis or light irradiation (entries 13–14). Formation of **3a** was completely suppressed when 2 equiv of TEMPO was added, forming side product *O*-butyl TEMPO in 16% yield (entry 15).

**Table 1:** Minisci C–H alkylation of **1** under visible light

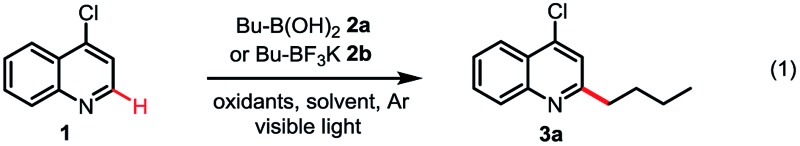				
Entry	Reagents (equiv.)	Solvents	*t* (°C)/time (h)	Yield^*a*^ (%) **3a**
1	**2a** (1.5), AgNO_3_ (0.2), K_2_S_2_O_8_ (3)	DCM/H_2_O	30/24	18
2	**2b** (1.5), Mn(OAc)_3_ (2.5), TFA (1)	AcOH/H_2_O	50/18	30
3	**2a** (1.5), Bl-OAc (2), Ru(bpy)_3_Cl_2_ (0.01), VL^*b*^	DCM	30/24	33
4	**2a** (1.5), Bl-OAc (2), Ru(bpy)_3_Cl_2_ (0.01), VL	CH_3_CN	30/24	38
5	**2a** (1.5), Bl-OAc (2), Ru(bpy)_3_Cl_2_ (0.01), VL, Ar	HFIP	30/24	88 (82^*c*^)
6	**2b** (1.5), Bl-OAc (2), Ru(bpy)_3_Cl_2_ (0.01), VL	HFIP	30/24	59
7	**2a** (1.5), Bl-OH (2), Ru(bpy)_3_Cl_2_ (0.01), VL	HFIP	30/24	82
8	**2a** (1.5), Bl-OMe (2), Ru(bpy)_3_Cl_2_ (0.01), VL	HFIP	30/24	61
9	**2a** (1.5), Bl-N_3_ (2), Ru(bpy)_3_Cl_2_ (0.01), VL	HFIP	30/24	25
10	**2a** (1.5), Bl-Cl (2), Ru(bpy)_3_Cl_2_ (0.01), VL	HFIP	30/24	<2
11	**2a** (1.5), PhI(OAc)_2_ (2), Ru(bpy)_3_Cl_2_ (0.01), VL	HFIP	30/24	<2
12	**2a** (1.5), Bl-OAc (2), Ir(ppy)_3_ (0.01), VL	HFIP	30/24	22
13	**2a** (1.5), Bl-OAc (2), VL	HFIP	30/24	<2
14	**2a** (1.5), Bl-OAc (2), Ru(bpy)_3_Cl_2_ (0.01), in darkness	HFIP	30/24	<2
15	**2a** (1.5), Bl-OAc (2), TEMPO (2), Ru(bpy)_3_Cl_2_ (0.01), VL	HFIP	30/24	<2

^*a*^Yields are based on ^1^H-NMR analysis on a 0.2 mmol scale.

^*b*^VL: compact household fluorescent bulb, 20 W.

^*c*^Isolated yield.

**Scheme 2:**
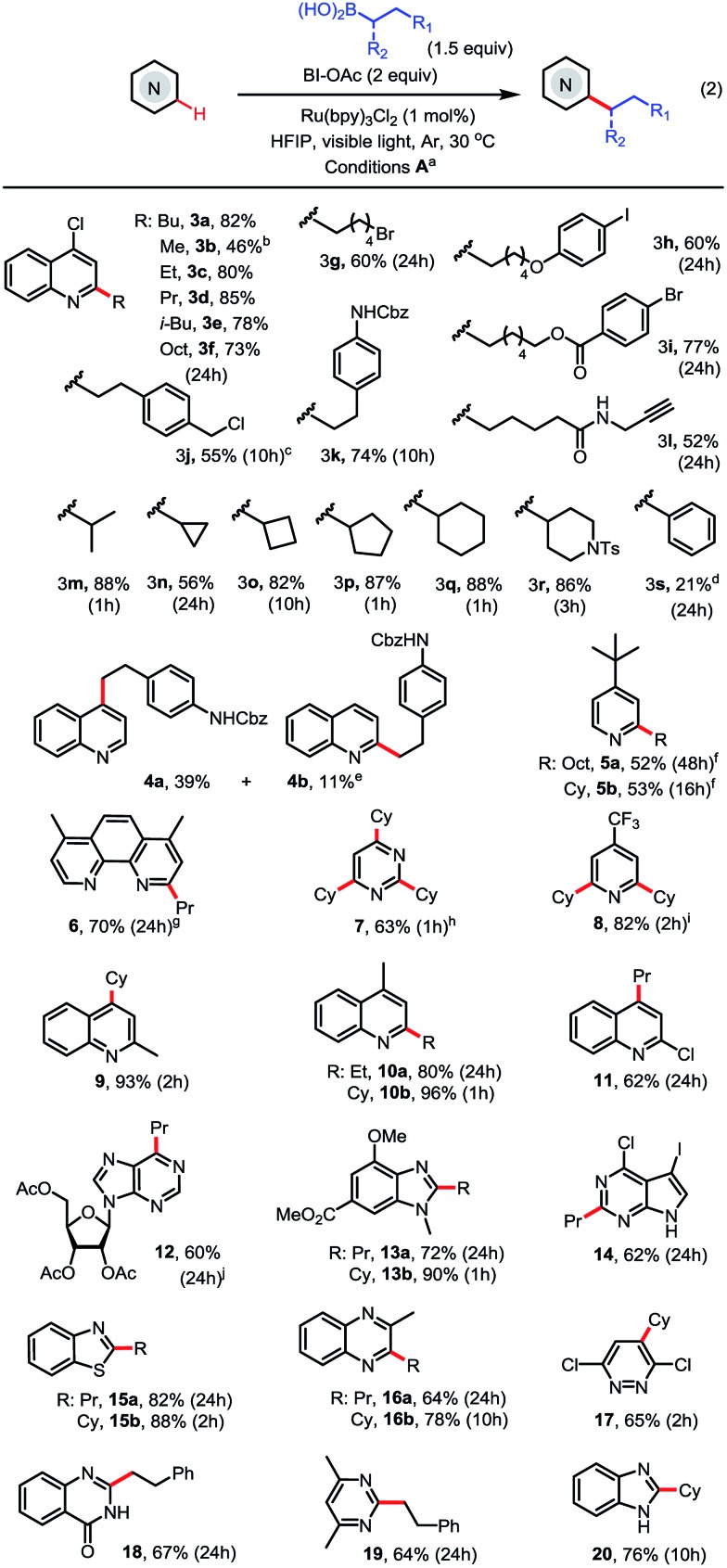
Substrate scope of photoredox-mediated C–H alkylation of *N*-heteroarenes. (a) Isolated yield on 0.2 mmol scale; (b) 2 equiv. of MeB(OH)_2_, 48 h; (c) 2 equiv. of RB(OH)_2_; (d) 1.5 equiv. of PhB(OH)_2_; (e) 1 equiv. of RB(OH), 2,4-dialkylated product was formed in <5% yield; (f) 2,6-dialkylated product was formed in <3% yield; (g) 2,2′-dialkylated product was formed in <5% yield; (h) 4.5 equiv. of CyB(OH)_2_; (i) 3 equiv. of CyB(OH)_2_. (j) other alkylated regioisomer was formed in <5% yield.

With the optimized conditions in hand, we next explored the substrate scope (Scheme 2). As seen in **3c–3l**, a range of primary alkyl boronic acids reacted with 4-chloroquinoline **3** to give C2-alkylated products in good to excellent yield. Methylation with MeB(OH)_2_ gave moderate yield (see **3b**). Primary alkyl radicals are more challenging reactants in Minisci reactions than secondary alkyl radicals due to their lower stability and nucleophilicity.^18^ We were pleased to observe that primary alkyl substituents carrying various functional groups, including alkyl bromide, aryl iodide, ester, amide, carbamate, terminal alkyne, and benzyl chloride, can be incorporated in good yield (see **3g–3l**). As seen in **3m–3r**, the alkylation reactions of secondary alkyl boronic acids are much faster than the primary and typically proceed in good to excellent yield under the standard conditions. In contrast to alkylation, arylation with PhB(OH)_2_ gave product **3s** in low yield (21%).

As seen in **4–11**, alkylation of pyridines and pyridine-based heteroarenes selectively took place at C2 and/or C4 positions. A mixture of C2-substituted **4a** and C4-substituted **4b** was obtained for unsubstituted quinoline. Alkylation of 2-methylquinoline selectively occurred at C4 to give **9** in excellent yield. In general, electron-deficient *N*-heteroarenes show higher reactivity toward alkylation. For instance, cyclohexylation of 4-*t*-butylpyridine predominantly resulted in mono-alkylation (see **5b**) while cyclohexylation of 4-CF_3_-pyridine gave 2,6-dialkylated **8** in excellent yield using 3 equiv. of boronic acid. A variety of other *N*-heteroarenes can also be alkylated in good yield and regioselectivity (see **12–20**). For instance, a purine riboside substrate was selectively alkylated at C6 to give **12** in excellent selectivity;^7*d*^ and a pyrrole-fused pyrimidine was selectively alkylated on the electron-deficient pyrimidine ring to give **14** in good yield. Without protection of the NH group, benzimidazole can be alkylated at C2 to give **20** in good yield.

**Scheme 3:**
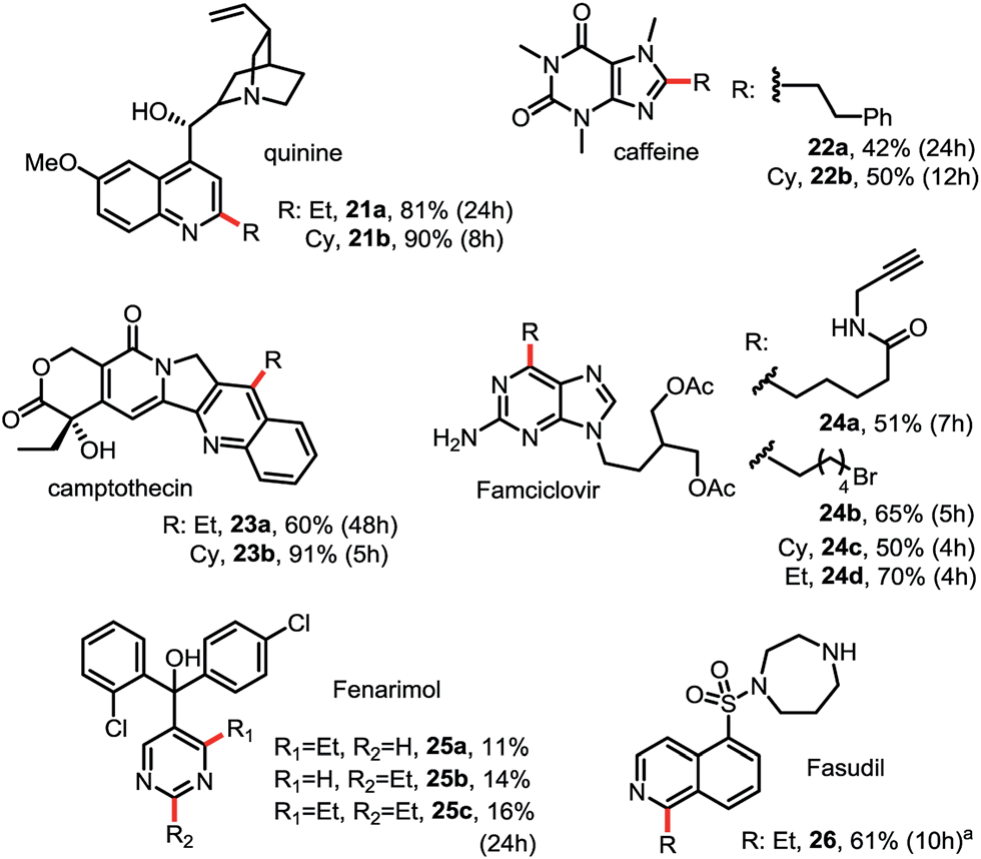
Minisci C–H alkylation for functionalization of natural products and drug molecules. Isolated yield on 0.2 mmol scale under the standard conditions with 1.5 equiv. of R-B(OH)_2_ (see Scheme 2). (a) Product was isolated in *N*-Boc protected form.

As shown in Scheme 3, this Minisci C–H alkylation can be readily applied to functionalize complex natural products and drug molecules.^2c,8,9a^ For instance, quinine with a free OH and vinyl group can be selectively alkylated at C2 position with both ethyl and cyclohexyl groups in excellent yield (see **21**). Camptothecin can be selectively alkylated at the C4 position of the pyridine ring (see **23**). Caffeine, a challenging substrate for previous Minisci reactions, can be selectively alkylated at C2 (see **22**).^7*b*^ Alkyl chains carrying an alkynyl or alkyl bromide group can be installed at C6 of Famciclovir in good yield (see **24a**, **24b**). Fasudil carrying a free secondary NH group was selectively alkylated at C1 in good yield (see **26**).

**Scheme 4:**
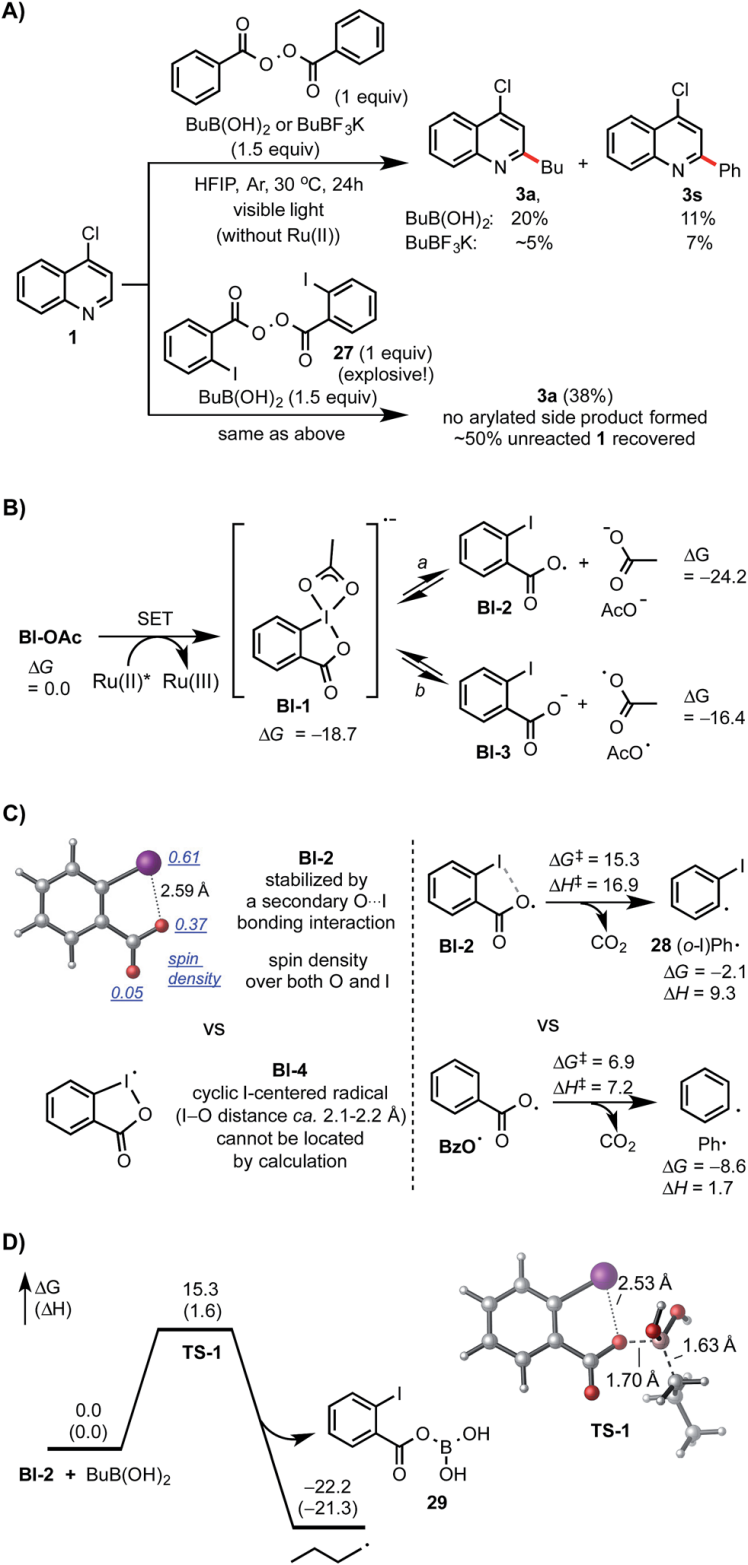
Mechanistic studies. DFT calculations were performed at the M06-2X/6-311++G(d,p)-SDD/SMD(HFIP)//M06-2X/6-31+G(d)-SDD level of theory. All energies are in kcal mol^–1^.

Control experiments and density functional theory (DFT) calculations have been carried out to probe the mechanism of this photoredox-mediated Minisci alkylation with alkyl boronic acids and Bl-OAc oxidant.^19^ As shown in Scheme 4A, we were surprised to observe that reaction of **1** with BuB(OH)_2_ and 1 equiv. of benzoyl peroxide in HFIP under visible light irradiation without photocatalyst also gave the alkylated product **3a** in 20% yield along with 11% yield of arylated side product **3s**, which is presumably formed from Ph˙ *via* the decarboxylation of BzO˙. In comparison with BuB(OH)_2_, BuBF_3_K showed much lower reactivity. Furthermore, **3a** was formed as the only C–H functionalized product in 38% yield when *ortho*-iodobenzoyl peroxide **27** (highly explosive) was used as the oxidant for reaction of **1** with BuB(OH)_2_ under the same conditions.^20^ These experiments suggest that benzoyloxy radicals can react with alkyl boronic acids to generate the requisite alkyl radical for the subsequent C–H alkylation. As shown in Scheme 4B, our DFT calculation showed that oxidant Bl-OAc can be readily reduced by photoexcited Ru(ii)* *via* single electron transfer (SET) to form a radical anion intermediate **Bl-1**, which then can undergo I–O bond cleavage to form radical **Bl-2** and acetate anion *via* pathway *a* or form **Bl-3** and acetoxy radical AcO˙ *via* pathway b.^21^ Formation of **Bl-2** is considerably more thermodynamically favorable than formation of AcO˙. Although a pair of interconvertible radical species, I-centered radical **Bl-4** and O-centered radical **Bl-2**, have been invoked in a number of previous studies,^22^ the postulated cyclic structure of **Bl-4** with a typical I–O bond length of *ca.* 2.1–2.2 Å cannot be located in our DFT calculation (Scheme 4C).^23^ Instead, the acyclic radical intermediate **Bl-2** is stabilized by a secondary I–O bonding interaction (∼2.6 Å) and its spin density is distributed between the O and I atoms.^24^ Calculation also revealed that **Bl-2** is notably more stable than benzoyloxy radical BzO˙ and is much less prone to undergo decarboxylation to form the corresponding aryl radical, which could cause the C–H arylation side reaction.^25^ Similar to the nucleo-homolytic substitution reaction of more reactive alkylboranes with O-centered radicals, **Bl-2** could react with the less Lewis-acidic boronic acids to form an alkyl radical R˙ *via* a radical “ate” transition state.^26–28^ The DFT calculation showed that this is a facile process at ambient temperature and highly exothermic (Scheme 4D).

**Scheme 5:**
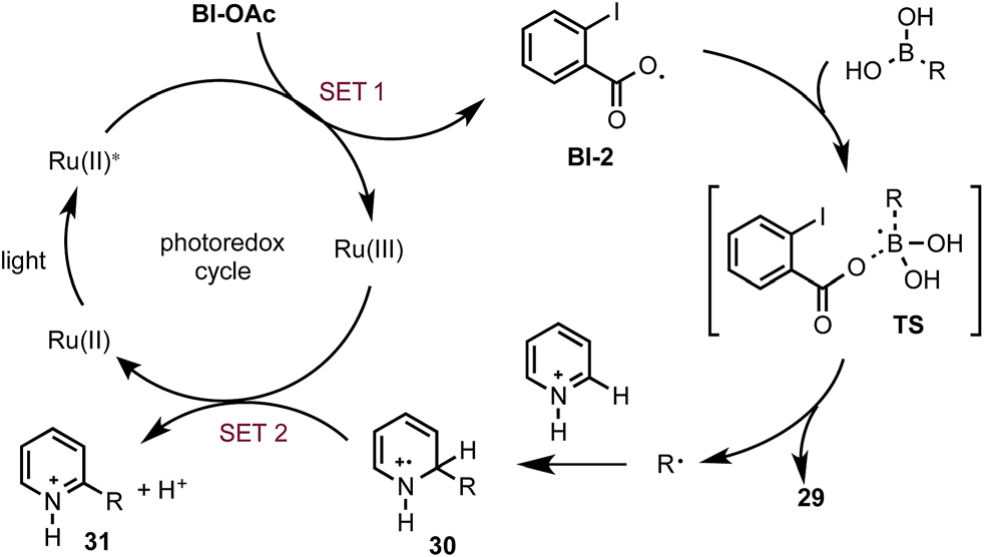
Proposed mechanism.

Based on the above studies, we propose that the reaction with boronic acid substrates is initiated with the SET from photoexcited Ru(ii)* to Bl-OAc (Scheme 5). The resulting **Bl-2** reacts with boronic acid to form a R˙, which then undergoes nucleophilic addition reaction with protonated *N*-heteroarenes to form a σ-complex. Single-electron oxidation of this intermediate by Ru(iii) and deprotonation gives the final C–H alkylated product and closes the photoredox cycle.^29^

## Conclusions

In summary, we have developed a photoredox-mediated Minisci C–H alkylation reaction of *N*-heteroarenes with easily accessible alkyl boronic acids. A broad range of alkyl groups, including challenging primary alkyl groups, can be readily incorporated into various *N*-heteroarenes with high efficiency under mild conditions. These reactions exhibit excellent substrate scope and functional group tolerance, and offer a broadly applicable method for the late-stage functionalization of complex substrates. Mechanistic studies have revealed that acetoxybenziodoxole serves as a facile precursor for an *ortho*-iodobenzoyloxy radical intermediate under photoredox catalysis. The unique property of this intramolecularly stabilized benzoyloxy radical might be critical for the efficient transformation of usually less reactive alkyl boronic acids to form alkyl radicals. Further mechanistic studies and application of benziodoxole reagents in other photoredox-mediated reaction systems are currently underway.

## References

[cit1] (a) Bioactive heterocycles V, Topics in Heterocyclic Chemistry, ed. R. R. Gupta, Springer Verlag, New York, 2008, vol. 11.

[cit2] Seregin I. V., Gevorgyan V. (2007). Chem. Soc. Rev..

[cit3] Ackermann L. (2010). Chem. Commun..

[cit4] Minisci F., Vismara E., Fontana F. (1989). Heterocycles.

[cit5] Seiple I. B., Su S., Rodriguez R. A., Gianatassio R., Fujiwara Y., Sobel A. L., Baran P. S. (2010). J. Am. Chem. Soc..

[cit6] Molander G. A., Colombel V., Braz V. A. (2011). Org. Lett..

[cit7] Correia C. A., Yang L., Li C.-J. (2011). Org. Lett..

[cit8] DiRocco D. A., Dykstra K., Krska S., Vachal P., Conway D. V., Tudge M. (2014). Angew. Chem., Int. Ed..

[cit9] Jin J., MacMillan D. W. C. (2015). Nature.

[cit10] Studer A., Curran D. P. (2016). Angew. Chem., Int. Ed..

[cit11] Yasu Y., Koike T., Akita M. (2012). Adv. Synth. Catal..

[cit12] Huang H., Jia K., Chen Y. (2015). Angew. Chem., Int. Ed..

[cit13] Tellis J. C., Primer D. N., Molander G. A. (2014). Science.

[cit14] Wang Y., Li G.-X., Yang G., He G., Chen G. (2016). Chem. Sci..

[cit15] Moteki S. A., Usui A., Selvakumar S., Zhang T., Maruoka K. (2014). Angew. Chem., Int. Ed..

[cit16] Yoshimura A., Zhdankin V. V. (2016). Chem. Rev..

[cit17] Narayanam J. M. R., Stephenson C. R. J. (2011). Chem. Soc. Rev..

[cit18] Togo H., Advanced Free Radical Reactions for Organic Synthesis, Elsevier, 2004.

[cit19] (a) Frisch M. J., et al., DFT calculations were performed using Gaussian 09, Revision D.01, Gaussian, Inc., Wallingford CT, 2009.

[cit20] (a) Extreme caution should be used for preparation and handling of **27**: Leffler J. E. Faulkner R. D. Petropoulos C. C., J. Am. Chem. Soc., 1958, 80 , 5435 .

[cit21] Winget P., Cramer C. J., Truhlar D. G. (2004). Theor. Chem. Acc..

[cit22] Zhdankin V. V., Krasutsky A. P., Kuehl C. J., Simonsen A. J., Woodward J. K., Mismash B., Bolz J. T. (1996). J. Am. Chem. Soc..

[cit23] (c) An unusually long I–O bond of 2.48 Å was observed in an arylbenziodoxle compound; the I–O interaction was described as a weak primary bond or strong intramolecular secondary bond: Batchelor R. J. Birchall T. Sawyer J. F., Inorg. Chem., 1986, 25 , 1415 .

[cit24] **Bl-2** is probably best described as a resonance structure between the O-centered and I-centered radicals

[cit25] No detectable C–H *ortho*-iodoarylated side products were observed in our alkylation reaction system

[cit26] Although the intramolecular I–O interaction stabilizes **Bl-2**, there is still sufficient radical character on the O atom that manifests similar reactivity as that of typical O-centered radicals

[cit27] Ollivier C., Renaud P. (2001). Chem. Rev..

[cit28] For a leading review on homolytic substitution reaction: Davies A. G. Roberts B. P., Acc. Chem. Res., 1972, 5 , 387 .

[cit29] The control experiment in Scheme 4A suggest that the reaction of alkyl boronic acids and trifluobororates might proceed with different mechanisms under our reaction conditions. It is plausible that Ru(iii) formed *via* the oxidation by Bl-OAc might react with the easily oxidizable alkyl trifluoroborates to give the alkyl radical and Ru(ii). Such process has been proposed in the previous studies of Bl-mediated photoredox-catalyzed reaction system using alkyl trifluoroborates (ref. 12). Formation of alkyl radical *via* SET oxidation of alkyl trifluoroborates by Ru(iii) or Ir(x) have been proposed in other photoredox-mediated system, see ref. 11 and 13. As seen in the control experiment with benzoyl peroxide, benzoyloxy radical might also be able to react with σ complex **30** *via* H-abstraction to form the final alkylated product and close the photoredox cycle

