# Correction: Photoredox-mediated Minisci C–H alkylation of *N*-heteroarenes using boronic acids and hypervalent iodine

**DOI:** 10.1039/c6sc90052f

**Published:** 2016-08-11

**Authors:** Guo-Xing Li, Cristian A. Morales-Rivera, Yaxin Wang, Fang Gao, Gang He, Peng Liu, Gong Chen

**Affiliations:** a State Key Laboratory and Institute of Elemento-Organic Chemistry, Collaborative Innovation Center of Chemical Science and Engineering (Tianjin), Nankai University Tianjin 300071 China gongchen@nankai.edu.cn; b Department of Chemistry, University of Pittsburgh Pittsburgh PA 15260 USA pengliu@pitt.edu; c Department of Chemistry, The Pennsylvania State University 104 Chemistry Building University Park PA 16802 USA guc11@psu.edu

## Abstract

Correction for ‘Photoredox-mediated Minisci C–H alkylation of *N*-heteroarenes using boronic acids and hypervalent iodine’ by Guo-Xing Li *et al.*, *Chem. Sci.*, 2016, DOI: 10.1039/c6sc02653b.

The authors regret that the name of the second author was presented incorrectly in the original article. Cristian A. Morales-Rivera's name is shown correctly herein.

The Royal Society of Chemistry apologises for these errors and any consequent inconvenience to authors and readers.

## Supplementary Material

