# Supplementary material for: Photoredox-mediated Minisci C–H alkylation of N-heteroarenes using boronic acids and hypervalent iodine
Source: Chem Sci. 2016 Jul 12;7(10):6407–12. doi: 10.1039/c6sc02653b (PMC5356021; doi:10.1039/c6sc02653b)
Supplement: Supplementary file 1 [file SC-007-C6SC02653B-s001.pdf]

Supporting Information

Photoredox-Mediated Minisci C-H Alkylation of *N*-Heteroarenes Using Boronic Acids and Hypervalent Iodine

Guo-Xing Li,[a] Cristian A Morales-Rivera,[b] Yaxin Wang, [a] Fang Gao,[a] Gang He,[a]
Peng Liu,*[b] and Gong Chen*[a,c]

[a]State Key Laboratory and Institute of Elemento-Organic Chemistry,
Collaborative Innovation Center of Chemical Science and Engineering (Tianjin),
Nankai University, Tianjin 30071, China

[b]Department of Chemistry, University of Pittsburgh, Pittsburgh, Pennsylvania 15260, USA

[c]Department of Chemistry, The Pennsylvania State University, University Park, Pennsylvania 16802, USA

Content

1. Reagents, S2

2. Instruments, S2

3. Synthesis of *N*-heteroaromatic substrates, S3

4. Synthesis of alkyl boronic acids, S5

5. Reaction optimization for Minisci C-H alkylation of compound 1, S11

6. General procedures and substrate scope for Minisci C-H alkylation, S12

7. Mechanism study, S32

8. DFT calculations, S34

9. References, S49

10. ^{1}H-NMR and ^{13}C-NMR spectra, S51

1. Reagents

All commercial materials were used as received unless otherwise noted. DCM was dried by distillation over CaH_2. Anhydrous CH_3CN was purchased from Acros Organics and stored under nitrogen atmosphere. TLC were performed on silica gel Huanghai HSGF254 plates and visualization of the developed chromatogram was performed by fluorescence quenching (λ_{max} = 254 nm). Flash chromatography was performed using Silica gel (200-300 mesh) purchased from Qingdao Haiyang Chemical Co., China. Hydroxylbenziodoxole (BI-OH),[1] acetoxybenziodoxole (BI-OAc),[1] azidobenziodoxole (BI-N_3)[1], methoxylbenziodoxole (BI-OMe),[2] chlorobenziodoxole (BI-Cl)[3] were synthesized according to reported procedures and used as freshly prepared. $Ru(bpy)_3Cl_2$ (98%, Ru＞15.75%, Energy Chemical) and HFIP (99.0%, J&K Chemical) were used as received.

2. Instruments

NMR spectra were recorded on Bruker AVANCE AV 400 instruments and all NMR experiments were reported in units, parts per million (ppm). Peaks recorded are relative to internal standards: TMS (δ = 0.00) for ^1H and $CDCl_3$ (δ = 77.00) for ^{13}C spectra. Multiplicities are recorded as: s = singlet, d = doublet, t = triplet, dd = doublet of doublets, br s = broad singlet, m = multiplet. High resolution ESI mass experiments were operated on a Waters LCT Premier instrument. All reactions were carried out in a 4 mL glass vial (Thermo SCIENTIFIC National B7999-2, made from superior quality 33 expansion borosilicate clear glass), sealed with PTEF cap on bench top.

3. Synthesis of *N*-heteroaromatic substrates

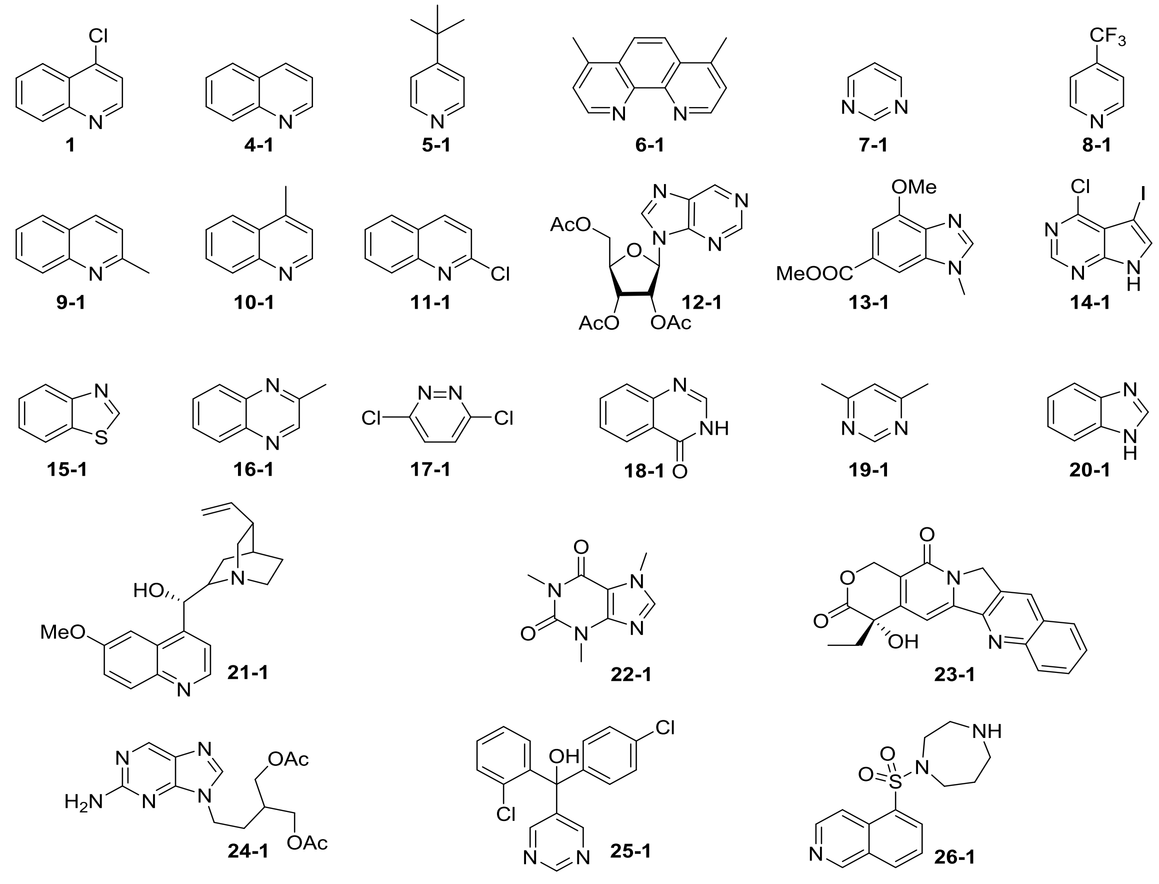

Scheme S1. List of all *N*-heteroaromatic substrates used in this study

Compounds **1**, **4-1**, **5-1**, **6-1**, **7-1**, **8-1**, **9-1**, **10-1**, **11-1**, **14-1**, **15-1**, **16-1**, **17-1**, **18-1**, **19-1**, **20-1**, **21-1**, **22-1**, **23-1**, **24-1**, **25-1**, **26-1** were commercial available and used as received.

3.1 Synthesis of 12-1

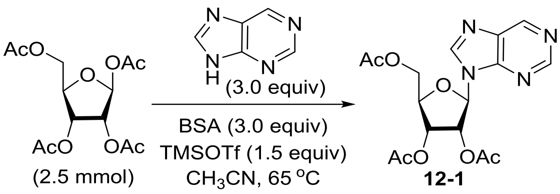

Scheme S2.

Compound **12-1** was prepared following a reported procedure[4]: 1,2,3,5-*O*-Tetracetyl-β-L-ribofuranose (800 mg, 2.5 mmol, 1.0 equiv) and purine (900 mg, 7.5 mmol, 3.0 equiv) were co-evaporated three times with anhydrous CH_3CN under argon. Then anhydrous CH_3CN (50 mL) and BSA (*N,O-bis*(trimethylsilyl)acetamide, 1.85 mL, 7.5 mmol, 3.0 equiv) were added

at room temperature. The mixture was stirred at 60-65 ℃ until all the base was dissolved. TMSOTf (435 μL, 3.75 mmol, 1.5 equiv) was added, and the resulting mixture was stirred overnight at 65 ℃. After cooled to room temperature, the mixture was diluted with DCM (250 mL), washed with saturated aqueous $NaHCO_3$ solution (20 mL), dried over Na_2SO_4 and filtrated. The filtrate was concentrated *in vacuo* and the residue was purified by flash chromatography on silica gel (ethyl acetate/petroleum ether = 4:1) to afford **12-1** (473 mg, 50%) as a colorless oil. Spectra data are consistent with those reported in the literature.[4]

3.2 Synthesis of 13-1

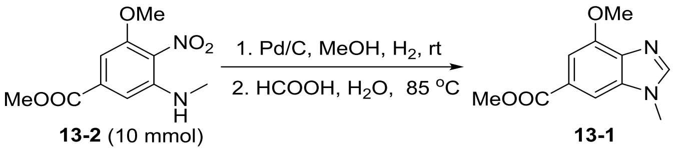

Scheme S3.

To a solution of compound **13-2** (2.41 g, 10 mmol, 1.0 equiv) in anhydrous methanol (100 mL) was added 5% Pd/C (2.12 g). The mixture was stirred at room temperature under an atmosphere of H_2 (balloon) for 20 h then filtrated through a pad of Celite and washed with 20 mL of methanol. The filtrate was concentrated *in vacuo* and the residue was dissolved in a mixture of water (20 mL) and formic acid (20 mL). The solution was then heated at 85 °C for 12 h. After cooled to room temperature, the solvent was removed under reduced pressure. The residue was partitioned between EtOAc (150 mL) and saturated aqueous $NaHCO_3$ solution (50 mL). The organic layer was washed with brine, dried over Na_2SO_4 and filtrated. The filtrate was concentrated *in vacuo* and the resulting residue was purified by flash chromatography on silica gel (ethyl acetate/petroleum ether = 4:1) to afford compound **13-1** (1.40 g, 63% for 2 steps) as a white solid. **^{1}H NMR** (400 MHz, $CDCl_3$) δ 8.13 (s, 1H), 7.79 (s, 1H), 7.39 (s, 1H), 4.06 (s, 3H), 3.99 (s, 3H), 3.94 (s, 3H); **^{13}C NMR** (101 MHz, $CDCl_3$) δ 167.62, 147.08, 145.20, 145.12, 127.56, 124.77, 116.08, 104.24, 55.74, 52.11, 33.93; **HRMS** Calcd for $C_{11}H_{13}N_2O_3$ [M+H$^+$]: 221.0926, Found: 221.0925.

4. Synthesis of alkyl boronic acids

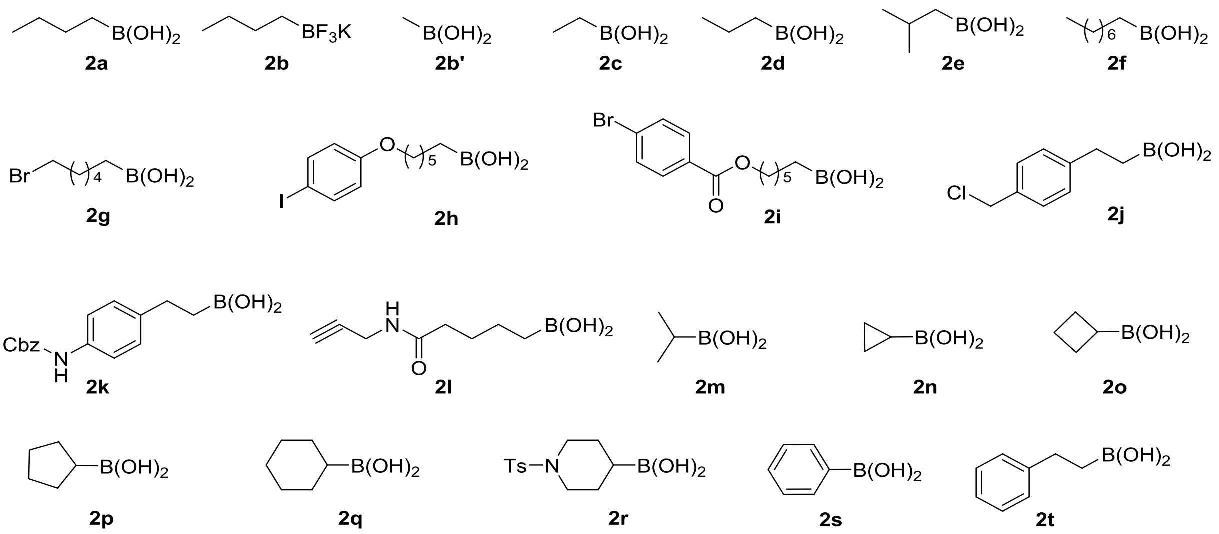

Scheme S4. List of all alkyl boronic acids used in this study

Compounds **2a**, **2b**, **2b'**, **2c**, **2d**, **2e**, **2f**, **2m**, **2n**, **2o**, **2p**, **2q**, **2s**, and **2t** were commercial available and used as received.

4.1 Synthesis of alkene substrates 2h-1, 2i-1 and 2k-1

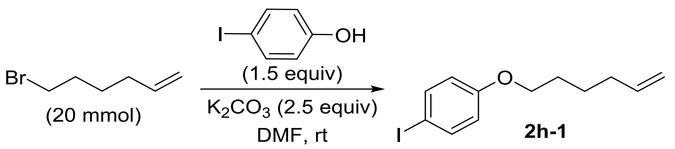

Scheme S5.

To a solution of 6-bromohex-1-ene (3.24 g, 20 mmol, 1.0 equiv) in DMF (30 mL) was added 4-iodophenol (6.60 g, 30 mmol, 1.5 equiv) and K_2CO_3 (7.46 g, 50 mmol, 2.5 equiv). The mixture was stirred at room temperature until 6-bromohex-1-ene was completely consumed monitoring by TLC. The reaction mixture was poured into brine (200 mL) and extracted with Et_2O (40 mL x 3). The combined organic phase was dried over Na_2SO_4 and filtrated. The filtrate was concentrated *in vacuo* and the residue was purified by flash chromatography on silica gel (ethyl acetate/petroleum ether = 5:95) to afford compound **2h-1** (5.22 g, 86%) as a colorless oil. Spectra data are consistent with those reported in the literature.[5]

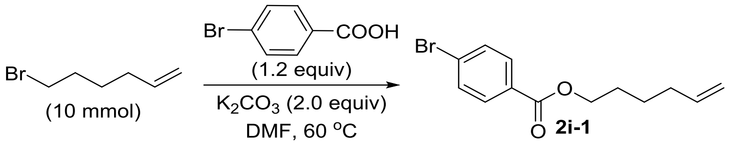

Scheme S6.

To a solution of 6-bromohex-1-ene (3.24 g, 20 mmol, 1.0 equiv) in DMF (30 mL) was added 4-bromobenzoic acid (4.84 g, 24 mmol, 1.2 equiv) and K_2CO_3 (5.53 g, 40 mmol, 2.0 equiv) at room temperature. Then the mixture was stirred at 60 °C until 6-bromohex-1-ene was completely consumed monitoring by TLC. After cooled to room temperature, the reaction mixture was poured into brine (200 mL) and extracted with Et_2O (40 mL x 3). The combined organic phase was dried over Na_2SO_4 and filtrated. The filtrate was concentrated *in vacuo* and the residue was purified by flash chromatography on silica gel (ethyl acetate/petroleum ether = 5:95) to afford compound **2h-1** (3.80 g, 67%) as a colorless oil. Spectra data are consistent with those reported in the literature.[6]

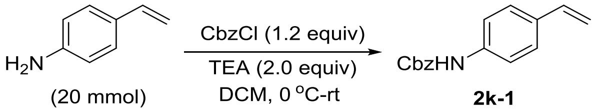

Scheme S7.

To a solution of 4-vinylaniline (2.39 g, 20 mmol, 1.0 equiv) and TEA (4.07g, 40 mmol, 2.0 equiv) in DCM (50 mL) was slowly added CbzCl (4.10 g, 24 mmol, 1.2 equiv) at 0 ℃. After addition completed, the mixture was stirred at room temperature until 4-vinylaniline was completely consumed monitoring by TLC. The reaction mixture was diluted with DCM (100 mL), and washed with 1 N HCl (20 mL), water (20 mL) and brine (20 mL) successively. The organic layer was dried over Na_2SO_4 and filtrated. The filtrate was concentrated *in vacuo* and the residue was purified by flash chromatography on silica gel (ethyl acetate/petroleum ether = 5:95) to afford compound **2k-1** (4.10 g, 81%) as a colorless solid. Spectra data are consistent with those reported in the literature.[7]

4.2 General Procedure for Synthesis of alkyl boronic acids 2g-2k

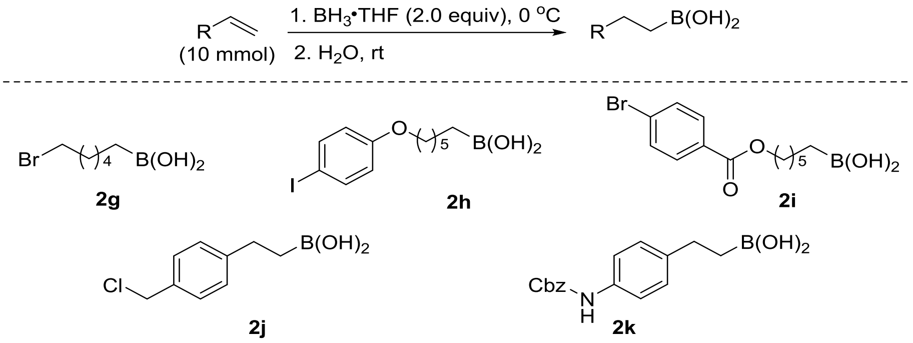

Scheme S8.

The alkyl boronic acid substrates were prepared following a reported procedure[8]: to a solution of alkene (10 mmol, 1.0 equiv) in THF (2 mL) was added a solution of BH_3•THF (20 mL, 20 mmol, 2.0 equiv, 1.0 M solution in THF) dropwise at 0 ℃. The mixture was stirred for 2 h at room temperature and H_2O (2 mL) was added carefully. After stirred for additional 3 h at room temperature, the reaction mixture was concentrated *in vacuo*. The residue was dissolved in EtOAc (30 mL), washed with saturated aqueous $NaHCO_3$ solution (20 mL) and brine (20 mL). The organic layer was dried over Na_2SO_4 and filtrated. The filtrate was concentrated *in vacuo* to approximately 5 mL. Petroleum ether was then added under stirring. The resultant precipitate was collected by filtration, washed with petroleum ether and dried under high vacum to afford the desired alkyl boronic acid as a white solid.

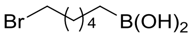

2g

Compound **2g** was prepared in 42% yield following the general procedure. **^{1}H NMR** (DMSO-*d*$_6$, 400 MHz) δ 7.37 (s, 2H), 3.52 (t, *J* = 6.7 Hz, 2H), 1.82-1.75 (m, 2H), 1.40-1.21 (m, 6H), 0.58 (t, *J* = 7.6 Hz, 2H); **^{13}C NMR** (DMSO-*d*$_6$, 100 MHz) δ 35.75, 32.72, 31.61, 27.92, 24.51, 15.80; **HRMS** Calcd for $C_6H_{14}BBrNaO_2$ [M+Na$^+$]: 231.0168, Found: 231.0134.

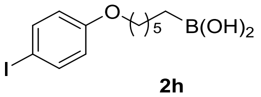

2h

Compound **2h** was prepared in 45% yield following the general procedure. **^{1}H NMR** (DMSO-*d*$_6$, 400 MHz) δ 7.60 (d, *J* = 8.4 Hz, 2H), 7.40 (s, 2H), 6.81 (d, *J* = 8.4 Hz, 2H), 3.95 (t, *J* = 6.3

Hz, 2H), 1.74-1.67 (m, 2H), 1.40-1.31 (m, 6H), 0.61 (t, *J* = 7.3 Hz, 2H); **^{13}C NMR** (DMSO-*d_6*, 100 MHz) δ 159.03, 138.37, 117.67, 83.25, 68.07, 32.29, 29.02, 25.86, 24.64, 15.88; **HRMS** Calcd for $C_{12}H_{18}BINaO_3$ [M+Na$^+$]: 371.0291, Found: 371.0273.

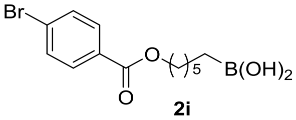

2i

Compound **2i** was prepared in 31% yield following the general procedure. **^{1}H NMR** (DMSO-*d_6*, 400 MHz) δ 7.88 (d, *J* = 8.5 Hz, 2H), 7.74 (d, *J* = 8.5 Hz, 2H), 7.39 (s, 2H), 4.25 (t, *J* = 6.5 Hz, 2H), 1.72-1.65 (m, 2H), 1.41-1.23 (m, 6H), 0.58 (t, *J* = 7.4 Hz, 2H); **^{13}C NMR** (DMSO-*d_6*, 100 MHz) δ 165.56, 132.38, 131.53, 129.51, 127.77, 65.51, 32.17, 28.56, 25.85, 24.57, 15.84; **HRMS** Calcd for $C_{13}H_{18}BBrNaO_4$ [M+Na$^+$]: 351.0379, Found: 351.0394.

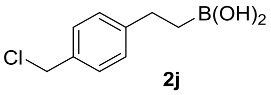

2j

Compound **2j** was prepared in 46% yield following the general procedure. **^{1}H NMR** (DMSO-*d_6*, 400 MHz) δ 7.53 (s, 2H), 7.31 (d, *J* = 8.0 Hz, 2H), 7.18 (d, *J* = 8.0 Hz, 2H), 4.71 (s, 2H), 2.63 (t, *J* = 8.2 Hz, 2H), 0.90 (t, *J* = 8.2 Hz, 2H); **^{13}C NMR** (DMSO-*d_6*, 100 MHz) δ 145.90, 134.98, 129.26, 128.47, 46.79, 30.38, 17.84; **HRMS** Calcd for $C_9H_{12}BClNaO_2$ [M+Na$^+$]: 221.0517, Found: 221.0509.

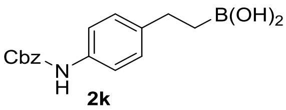

2k

Compound **2k** was prepared in 31% yield following the general procedure. **^{1}H NMR** (DMSO-*d_6*, 400 MHz) δ 9.64 (s, 1H), 7.51 (s, 2H), 7.42-7.33 (m, 7H), 7.09 (d, *J* = 8.3 Hz, 2H), 5.14 (s, 2H), 2.57 (t, *J* = 8.1 Hz, 2H), 0.88 (t, *J* = 8.1 Hz, 2H); **^{13}C NMR** (DMSO-*d_6*, 100 MHz) δ 153.56, 139.34, 136.88, 136.51, 128.58, 128.21, 128.14, 128.08, 118.36, 65.73, 29.65, 17.76; **HRMS** Calcd for $C_{16}H_{18}BNNaO_4$ [M+Na$^+$]: 322.1227, Found: 322.1242.

4.3 Synthesis of alkyl boronic acid 2l

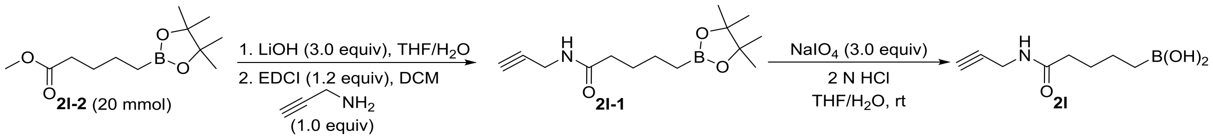

Scheme S9

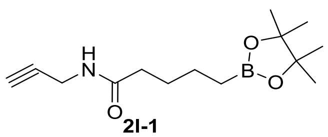

To a solution of compound **2l-2** (4.84 g, 20 mmol, 1.0 equiv) in a mixture of THF (40 mL) and H_2O (20 mL) was added LiOH (0.96 g, 60 mmol, 3.0 equiv). The mixture was stirred at room temperature until compound **2l-2** was completely consumed monitoring by TLC. The solvent was concentrated *in vacuo*, then ethyl acetate (50 mL) and water (10 mL) were added. Under vigorous stirring, the reaction was acidified with 2N HCl to pH 1-2. The organic phase was separated, and the aqueous phase was extracted with ethyl acetate (20 mL x 2). The combined organic phase was dried over Na_2SO_4 and filtrated. The filtrate was concentrated *in vacuo* and the residue was dissolved in DCM (50 mL). To this solution were added EDCI (2.18 g, 24 mmol, 1.2 equiv) and prop-2-yn-1-amine (1.1 g, 20 mmol, 1.0 equiv), and the mixture was stirred at room temperature for 1 h. Then the reaction mixture was diluted with DCM (150 mL), washed with water (20 mL), 1 N HCl (20 mL) and brine (20 mL) successively. The organic layer was dried over Na_2SO_4 and filtrated. The filtrate was concentrated *in vacuo* and the residue was purified by flash chromatography on silica gel (ethyl acetate/petroleum ether = 1:1) to afford compound **2l-1** (3.0 g, 56% for 2 steps) as a colorless oil. **^{1}H NMR** (400 MHz, $CDCl_3$) δ 5.85 (s, 1H), 4.06-4.05 (m, 2H), 2.23 (t, *J* = 2.6 Hz, 1H), 2.20 (d, *J* = 7.7 Hz, 1H), 1.66-1.61 (m, 2H), 1.49-1.41 (m, 2H), 1.25 (s, 12H), 0.81 (t, *J* = 7.7 Hz, 2H); **^{13}C NMR** (100 MHz, $CDCl_3$) δ 172.66, 83.03, 79.72, 71.45, 36.15, 29.05, 27.95, 24.79, 24.56, 23.48; **HRMS** Calcd for $C_{14}H_{24}BNNaO_3$ [M+Na$^+$]: 288.1747, Found: 288.1740.

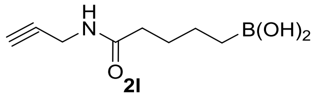

Compound **2l** was prepared following a reported procedure[9]: to a solution of compound **2l-1** (662 mg, 2.5 mmol, 1.0 equiv) in a mixture of THF (16 mL) and H_2O (4 mL), 2 N HCl (0.8

mL) and $NaIO_4$ (1.60 g, 7.5 mmol, 3.0 equiv) were added. The mixture was stirred at room temperature until compound **2l-1** was completely consumed monitoring by TLC. The reaction mixture was extracted with EtOAc, and the combined organic phases were washed with brine, dried over Na_2SO_4 and filtrated. The filtrate was concentrated *in vacuo* to approximately 5 mL. Petroleum ether was then added under stirring. The resultant precipitate was collected by filtration, washed with petroleum ether and dried under high vacum to afford the desired product **2l** as a white solid (180 mg, 39%). **^{1}H NMR** (DMSO-d_6, 400 MHz) δ 8.24 (t, J = 4.8 Hz, 1H), 7.39 (s, 2H), 3.83-3.81 (m, 2H), 3.08 (t, J = 2.0 Hz, 1H), 2.04 (t, J = 7.5 Hz, 2H), 1.49-1.43 (m, 2H), 1.35-1.27 (m, 2H), 0.60 (t, J = 7.7 Hz, 2H); ^{13}C NMR (DMSO-d_6, 100 MHz) δ 172.06, 81.36, 72.72, 35.16, 28.13, 27.66, 23.97, 15.16; **HRMS** Calcd for $C_8H_{14}BNNaO_3$ [M+Na$^+$]: 206.0964, Found: 206.0928.

4.4 Synthesis of alkyl boronic acid 2r

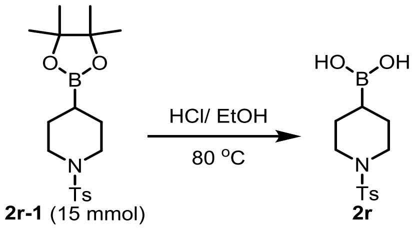

Scheme S10.

Compound **2r** was prepared following a reported procedure[9]: to a mixture of EtOH (10 mL) and concentrated hydrogen chloride (18 mL) was added compound **2r-1** (4.7 g, 15 mmol, 1.0 equiv). The mixture was stirred for 10 minutes at room temperature and then heated at 80 °C until the boronic esters were completely consumed monitoring by TLC. After cooled to room temperature, the reaction mixture was diluted with water and extracted with EtOAc. The combined organic phases were washed with brine, dried over Na_2SO_4 and filtrated. The filtrate was concentrated *in vacuo* and residue was recrystallized from EtOAc and petroleum ether to afford compound **2r** as a white solid (1.06 g, 55%). Spectra data are consistent with those reported in the literature.[9]

5. Reaction optimization for Minisci C-H alkylation of compound 1

All screening reactions were carried out at a 0.2 mmol scale in a 4 mL glass vial (Thermo Scientific, National B7999-2). The vials were purged with Ar for 1 min, sealed with PTEF cap and stirred on bench top. Stock solution of $Ru(bpy)_3Cl_2$ (0.04 mmol in 10 mL of HFIP) was used if necessary. A 20 W compact household fluorescent bulb was positioned 10 cm aside from the reaction vials if necessary.

Compound **1** (32.8 mg, 0.2 mmol, 1.0 equiv) and other specified reagents were dispersed in 0.5 mL of solvent containing photocatalyst. The mixture was stirred at specified temperature with or without light irradiation for 24 h. After removal of the solvent *in vacuo*, the residue was dissolved in 3 mL of $CDCl_3$ along with $Cl_2CHCHCl_2$ (20 μL) as an internal standard for ^1H NMR analysis. The composition of reaction mixture was analyzed based on the methylene group peaks at 2.95 ppm (t, J = 8.0 Hz, 2H) for compound **3a**.

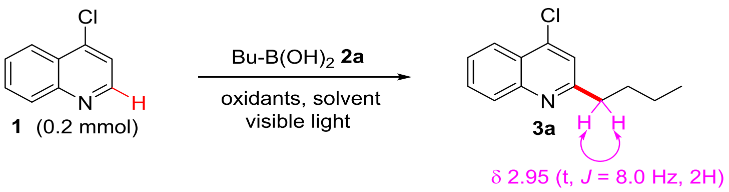

Entry	Reagents (equiv) / atmosphere	Solvents/ mL	t (°C) /Time (h)	Yield (%)[a] **3a**
1	**2a** (1.5), BI-OH (2), Ar	HFIP/0.5	80 / 48	20
2	**2a** (1.5), BI-OAc (2), Ar	HFIP/0.5	80 / 48	28
3	**2a** (1.5), BI-OAc (2), Ar	$CHCl_3$/0.5	80 / 48	<2
4	**2a** (1.5), BI-OAc (2), Ar	CH_3CN/0.5	80 / 48	<2
5	**2a** (1.5), BI-OAc (2), Ar	TFE/0.5	30 / 24	34
6	**2a** (1.5), $PhI(CF_3COO)_2$ (2), $Ru(bpy)_3Cl_2$ (0.01), VL[b], Ar	HFIP/0.5	30 / 24	<2
7	**2a** (1.5), BI-OAc (2), $Ru(bpy)_3Cl_2$ (0.01), VL, Ar	HFIP/0.25	30 / 24	69
8	**2a** (1.5), BI-OAc (2), $Ru(bpy)_3Cl_2$ (0.01), VL, Ar	HFIP/1.0	30 / 24	48

a) Yields are based on ^1H-NMR analysis on a 0.2 mmol scale.*b*) VL: compact household fluorescent bulb, 20 W.

Table S1. Minisci C-H alkylation of compound 1

6. General procedure and substrate scope for Minisci C-H alkylation

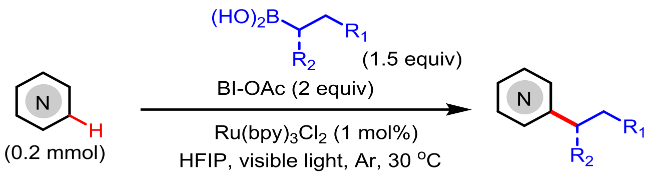

General condition: *N*-heteroaromatic substrate (0.2 mmol, 1.0 equiv), alkyl boronic acid (0.3 mmol, 1.5 equiv) and BI-OAc (0.4 mmol, 2.0 equiv) were added to a solution of $Ru(bpy)_3Cl_2$ (0.002 mmol, 0.01 equiv) in HFIP (0.5 mL). The reaction vial was purged with Ar for 1 min and then the mixture was stirred at 30 °C under the fluorescent light irradiation for 1-48 h. The solvent was removed *in vacuo* and the residue was dissolved in DCM (3 mL). To the solution was added K_2CO_3 (approximate 150 mg), and the resulting mixture was vigorously stirred for 5 min. Then the mixture was filtrated through a pad of Celite and washed with DCM. The filtrate was concentrated *in vacuo* and the residue was purified by preparative thin layer chromatography or flash chromatography on silica gel to afford the desired product.

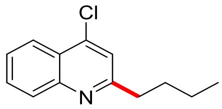

3a R_f = 0.6, 20% EtOAc in Hexane

Compound **3a** was prepared following the general procedure (24 h) in 82% yield as a colorless oil. **^{1}H NMR** (400 MHz, $CDCl_3$) δ 8.18 (dd, *J* = 8.3, 0.6 Hz, 1H), 8.05 (d, *J* = 8.4 Hz, 1H), 7.75-7.71 (m, 1H), 7.60-7.56 (m, 1H), 7.40 (s, 1H), 2.95 (t, J = 8.0 Hz, 2H), 1.83-1.76 (m, 2H), 1.49-1.40 (m, 2H), 0.97 (t, *J* = 7.4 Hz, 3H); **^{13}C NMR** (100 MHz, $CDCl_3$) δ 163.04, 148.73, 142.48, 130.23, 129.14, 126.60, 124.89, 123.89, 121.33, 38.84, 31.91, 22.59, 13.93; **HRMS** Calcd for $C_{13}H_{15}ClN$ [$M+H^+$]: 220.0893, Found: 220.0929.

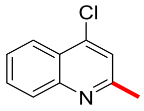

3b R_f = 0.2, 10% Methyl *tert*-butyl ether in Hexane

Compound **3b** was prepared following the general procedure (**2b'** 2.0 equiv, 48 h) in 46% yield as a colorless oil. **^{1}H NMR** (400 MHz, $CDCl_3$) δ 8.19 (dd, *J* = 8.4, 0.9 Hz, 1H), 8.03 (d, *J* = 8.4 Hz, 1H), 7.76-7.72 (m, 1H), 7.61-7.56 (m, 1H), 7.41 (s, 1H), 2.73 (s, 3H). Spectra data are consistent with those reported in the literature.[10]

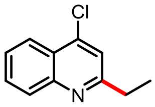

3c R_f = 0.5, 20% EtOAc in Hexane

Compound **3c** was prepared following the general procedure (24 h) in 80% yield as a colorless oil. **^{1}H NMR** (400 MHz, $CDCl_3$) δ 8.19 (dd, *J* = 8.4, 0.9 Hz, 1H), 8.05 (dd, *J* = 8.5, 0.4 Hz, 1H), 7.76-7.72 (m, 1H), 7.60-7.56 (m, 1H), 7.42 (s, 1H), 2.99 (q, *J* = 7.6 Hz, 2H), 1.40 (t, *J* = 7.6 Hz, 3H); **^{13}C NMR** (101 MHz, $CDCl_3$) δ 163.91, 148.71, 142.62, 130.27, 129.14, 126.63, 124.92, 123.91, 120.88, 32.11, 13.75; **HRMS** Calcd for $C_{11}H_{11}ClN$ [M+H$^+$]: 192.0580, Found: 192.0577.

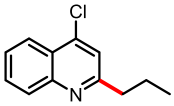

3d R_f = 0.5, 20% EtOAc in Hexane

Compound **3d** was prepared following the general procedure (24 h) in 85% yield as a colorless oil. **^{1}H NMR** (400 MHz, $CDCl_3$) δ 8.18 (d, *J* = 8.4 Hz, 1H), 8.05 (d, *J* = 8.4 Hz, 1H), 7.73 (dd, *J* = 8.4, 7.2 Hz, 1H), 7.58 (dd, *J* = 8.4, 7.2 Hz, 1H), 7.40 (s, 1H), 2.93 (t, *J* = 7.6 Hz, 2H), 1.89-1.80 (m, 2H), 1.03 (t, *J* = 7.3 Hz, 2H); **^{13}C NMR** (100 MHz, $CDCl_3$) δ 162.82, 148.73, 142.48, 130.24, 129.15, 126.61, 124.91, 123.89, 121.35, 41.01, 23.05, 13.93; **HRMS** Calcd for $C_{12}H_{13}ClN$ [M+H$^+$]: 206.0737, Found:206.0773.

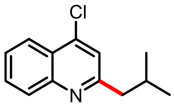

3e R_f = 0.6, 20% EtOAc in Hexane

Compound **3e** was prepared following the general procedure (24 h) in 78% yield as a colorless oil. **^{1}H NMR** (400 MHz, $CDCl_3$) δ 8.19 (dd, *J* = 8.4, 0.7 Hz, 1H), 8.06 (d, *J* = 8.2 Hz, 1H), 7.74-7.72 (m, 1H), 7.60-7.56 (m, 1H), 7.38 (s, 1H), 2.82 (d, *J* = 7.4 Hz, 2H), 2.27-2.16 (m, 1H), 0.99 (d, *J* = 6.6 Hz, 6H); **^{13}C NMR** (100 MHz, $CDCl_3$) δ 162.16, 148.72, 142.26, 130.18, 129.19, 126.59, 124.85, 123.86, 121.89, 121.85, 48.02, 29.30, 22.46; **HRMS** Calcd for $C_{13}H_{15}ClN$ [M+H$^+$]: 220.0893, Found: 220.0890.

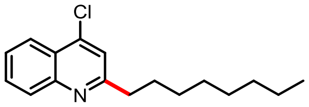

3f

R_f = 0.6, 10% EtOAc in Hexane

Compound **3f** was prepared following the general procedure (24 h) in 73% yield as a colorless oil. **^{1}H NMR** (400 MHz, $CDCl_3$) δ 8.18 (dd, *J* = 8.4, 1.0 Hz, 1H), 8.05 (d, *J* = 8.4 Hz, 1H), 7.76-7.71 (m, 1H), 7.60-7.56 (m, 1H), 7.40 (s, 1H), 2.94 (t, *J* = 8.0 Hz, 2H), 1.84-1.76 (m, 2H), 1.43-1.27 (m, 10H), 0.87 (t, *J* = 7.0 Hz, 3H); **^{13}C NMR** (100 MHz, $CDCl_3$) δ 163.07, 148.73, 142.47, 130.23, 129.15, 126.59, 124.89, 123.89, 121.33, 39.14, 31.82, 29.83, 29.48, 29.42, 29.18, 22.64, 14.09; **HRMS** Calcd for $C_{17}H_{23}ClN$ [M+H$^+$]: 276.1519, Found: 276.1515.

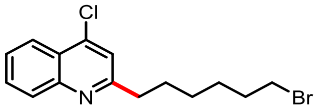

3g

R_f = 0.5, 10% EtOAc in Hexane

Compound **3g** was prepared following the general procedure (24 h) in 60% yield as a colorless oil. **^{1}H NMR** (400 MHz, $CDCl_3$) δ 8.19 (dd, *J* = 8.4, 0.8 Hz, 1H), 8.05 (d, *J* = 8.4 Hz, 1H), 7.76-7.72 (m, 1H), 7.61-7.57 (m, 1H), 7.40 (s, 1H), 3.41 (t, *J* = 6.8 Hz, 2H), 2.95 (t, *J* = 7.8 Hz, 2H), 1.91-1.80 (m, 4H), 1.55-1.41 (m, 4H); **^{13}C NMR** (100 MHz, $CDCl_3$) δ162.65, 148.71, 142.58, 130.31, 129.13, 126.69, 124.92, 123.92, 121.31, 38.90, 33.87, 32.60, 29.50, 28.52, 27.97; **HRMS** Calcd for $C_{15}H_{18}BrClN$ [M+H$^+$]: 326.0311, Found: 326.0308.

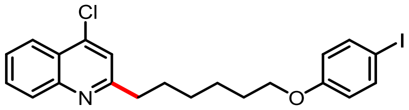

3h

R_f = 0.4, 10% EtOAc in Hexane

Compound **3h** was prepared following the general procedure (24 h) in 60% yield as a colorless solid. **^{1}H NMR** (400 MHz, $CDCl_3$) δ 8.19 (dd, *J* = 8.4, 0.9 Hz, 1H), 8.05 (d, *J* = 8.1 Hz, 1H), 7.76-7.72 (m, 1H), 7.61-7.56 (m, 1H), 7.54-7.51 (m, 2H), 7.40 (s, 1H), 6.67-6.63 (m, 2H), 3.90 (t, *J* = 6.5 Hz, 2H), 2.95 (t, *J* = 7.8 Hz, 2H), 1.86-1.76 (m, 4H), 1.56-1.43 (m, 4H); **^{13}C NMR** (100 MHz, $CDCl_3$) δ 162.73, 158.90, 148.69, 142.55, 138.10, 130.29, 129.12, 126.67, 124.90,

123.91, 121.30, 116.86, 82.41, 67.89, 38.93, 29.60, 29.07, 28.95, 25.82; **HRMS** Calcd for $C_{21}H_{22}ClINO$ [M+H$^+$]: 466.0435, Found: 466.0430.

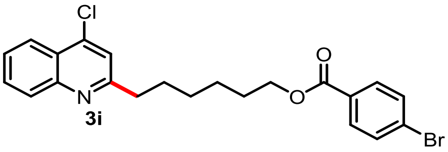

3i

R$_f$ = 0.5, 20% EtOAc in Hexane

Compound **3i** was prepared following the general procedure (24 h) in 77% yield as a colorless solid. **^{1}H NMR** (400 MHz, $CDCl_3$) δ 8.19 (d, *J* = 8.3 Hz, 1H), 8.04 (d, *J* = 8.4 Hz, 1H), 7.89 (d, *J* = 8.5 Hz, 2H), 7.79 -7.69 (m, 1H), 7.61-7.56 (m, 3H), 7.40 (s, 1H), 4.31 (t, *J* = 6.6 Hz, 2H), 2.96 (t, *J* = 7.8 Hz, 3H), 1.89-1.75 (m, 4H), 1.56-1.43 (m, 4H); **^{13}C NMR** (100 MHz, $CDCl_3$) δ 165.86, 162.64, 148.71, 142.52, 131.62, 131.03, 130.27, 129.29, 129.13, 127.87, 126.65, 124.88, 123.89, 121.26, 65.22, 38.91, 29.55, 29.03, 28.52, 25.88; **HRMS** Calcd for $C_{22}H_{22}BrClNO_2$ [M+H$^+$]: 446.0522, Found: 446.0520.

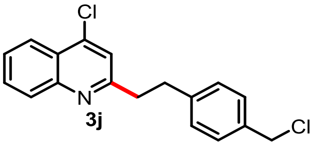

3j

R$_f$ = 0.65, 20% EtOAc in Hexane

Compound **3j** was prepared following the general procedure (**2j** 2.0 equiv, 10 h) in 55% yield as a colorless solid. **^{1}H NMR** (400 MHz, $CDCl_3$) δ 8.19 (d, *J* = 8.3 Hz, 1H), 8.08 (d, *J* = 8.4 Hz, 1H), 7.83-7.71 (m, 1H), 7.65 – 7.54 (m, 1H), 7.35 (s, 1H), 7.32 (d, *J* = 8.0 Hz, 2H), 7.25 (d, *J* = 8.4 Hz, 2H), 4.57 (s, 2H), 3.27-3.22 (m, 2H), 3.18-3.14 (m, 2H); **^{13}C NMR** (100 MHz, $CDCl_3$) δ 161.41, 148.77, 142.59, 141.55, 135.31, 130.37, 129.19, 128.85, 128.76, 126.84, 125.00, 123.96, 121.47, 46.15, 40.49, 35.19; **HRMS** Calcd for $C_{18}H_{16}Cl_2N$ [M+H$^+$]: 316.0660, Found: 301.0663.

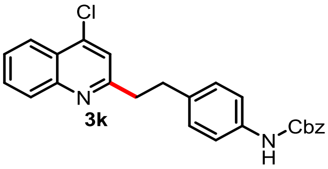

3k

R$_f$ = 0.15, 20% EtOAc in Hexane

Compound **3k** was prepared following the general procedure (10 h) in 74% yield as a colorless solid. **^{1}H NMR** (400 MHz, $CDCl_3$) δ 8.18 (d, *J* = 8.3 Hz, 1H), 8.07 (d, *J* = 8.4 Hz, 1H), 7.76-

7.72 (m, 1H), 7.61-7.59 (m, 1H), 7.38-7.29 (m, 8H), 7.17 (d, *J* = 8.3 Hz, 2H), 6.72 (s, 1H), 5.19 (s, 2H), 3.24-3.20 (m, 2H), 3.12-3.08 (m, 2H); **^{13}C NMR** (100 MHz, $CDCl_3$) δ 161.60, 153.34, 148.74, 142.54, 136.33, 136.05, 135.83, 130.33, 129.15, 129.03, 128.58, 128.30, 128.28, 126.78, 124.98, 123.94, 121.50, 118.84, 66.94, 40.69, 34.88; **HRMS** Calcd for $C_{25}H_{22}ClN_2O_2$ [M+H$^+$]: 417.1370, Found: 417.1368.

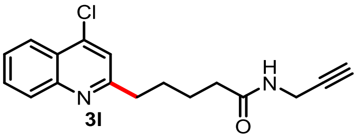

3l

R_f = 0.5, 100% EtOAc

Compound **3l** was prepared following the general procedure (24 h) in 52% yield as a colorless solid. **^{1}H NMR** (400 MHz, $CDCl_3$) δ 8.19 (dd, *J* = 8.3, 0.6 Hz, 1H), 8.04 (d, *J* = 8.4 Hz, 1H), 7.76-7.72 (m, 1H), 7.61-7.57 (m, 1H), 7.40 (s, 1H), 5.85 (s, 1H), 4.06-4.04 (m, 2H), 2.97 (t, *J* = 7.5 Hz, 2H), 2.27 (t, *J* = 7.3 Hz, 2H), 2.22 (t, *J* = 2.5 Hz, 1H), 1.91-1.84 (m, 2H), 1.81-1.74 (m, 2H); **^{13}C NMR** (100 MHz, $CDCl_3$) δ 172.25, 162.19, 148.66, 142.67, 130.34, 129.07, 126.74, 124.94, 123.94, 121.35, 79.58, 1.54, 38.44, 36.04, 29.11, 28.90, 25.08; **HRMS** Calcd for $C_{17}H_{18}ClN_2O$ [M+H$^+$]: 301.1108, Found: 301.1198.

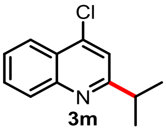

3m

R_f = 0.6, 10% EtOAc in Hexane

Compound **3m** was prepared following the general procedure (1 h) in 88% yield as a colorless oil. **^{1}H NMR** (400 MHz, $CDCl_3$) δ 8.17 (d, *J* = 8.0 Hz, 1H), 8.06 (d, *J* = 8.4 Hz, 1H), 7.75-7.71 (m, 1H), 7.59-7.55 (m, 1H), 7.43 (s, 1H), 3.28-3.18 (m, 1H), 1.39 (d, *J* = 6.9 Hz, 6H). Spectra data are consistent with those reported in the literature.[11]

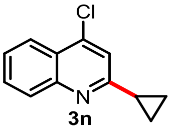

3n

R_f = 0.5, 10% EtOAc in Hexane

Compound **3n** was prepared following the general procedure (24 h) in 56% yield as a colorless oil. **^{1}H NMR** (400 MHz, $CDCl_3$) δ 8.14 (dd, *J* = 8.4, 0.9 Hz, 1H), 7.96 (d, *J* = 8.4 Hz, 1H),

7.72-7.68 (m, 1H), 7.55-7.51 (m, 1H), 7.29 (s, 1H), 2.23-2.17 (m, 1H), 1.19-1.14 (m, 1H), 1.13-1.08 (m, 1H); **^{13}C NMR** (100 MHz, $CDCl_3$) δ 163.49, 148.80, 142.13, 130.18, 128.97, 126.09, 124.85, 123.89, 119.56, 17.95, 10.47; **HRMS** Calcd for $C_{12}H_{11}ClN$ [M+H$^+$]: 204.0580, Found: 204.0627.

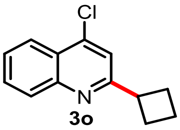

3o

R_f = 0.5, 10% EtOAc in Hexane

Compound **3o** was prepared following the general procedure (10 h) in 82% yield as a colorless oil. **^{1}H NMR** (400 MHz, $CDCl_3$) δ 8.14 (dd, *J* = 8.4, 0.9 Hz, 1H), 8.07 (d, *J* = 8.3 Hz, 1H), 7.75-7.70 (m, 1H), 7.59-7.54 (m, 1H), 7.43 (s, 1H), 3.87-3.78 (m, 1H), 2.48-2.42 (m, 4H), 2.18-2.06 (m, 1H), 2.00-1.92 (m, 1H). Spectra data are consistent with those reported in the literature.[12]

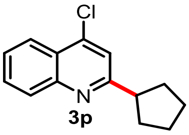

3p

R_f = 0.6, 10% EtOAc in Hexane

Compound **3p** was prepared following the general procedure (1 h) in 87% yield as a colorless oil. **^{1}H NMR** (400 MHz, $CDCl_3$) δ 8.16 (dd, *J* = 8.3, 1.0 Hz, 1H), 8.04 (d, *J* = 8.4 Hz, 1H), 7.74-7.70 (m, 1H), 7.58-7.54 (m, 1H), 7.43 (s, 1H), 3.39-3.30 (m, 1H), 2.21-2.14 (m, 2H), 1.94-1.73 (m, 6H); **^{13}C NMR** (100 MHz, $CDCl_3$) δ 166.25, 148.49, 142.40, 130.11, 129.24, 126.51, 124.99, 123.82, 120.15, 48.61, 33.45, 25.93; **HRMS** Calcd for $C_{14}H_{15}ClN$ [M+H$^+$]: 232.0893, Found: 232.0892.

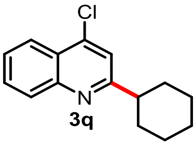

3q

R_f = 0.6, 10% EtOAc in Hexane

Compound **3q** was prepared following the general procedure (1 h) in 88% yield as a colorless oil. **^{1}H NMR** (400 MHz, $CDCl_3$) δ 8.17 (d, *J* = 8.3 Hz, 1H), 8.05 (d, *J* = 8.5 Hz, 1H), 7.74-7.70 (m, 1H), 7.59-7.54 (m, 1H), 7.42 (s, 1H), 2.92-2.86 (m, 1H), 2.04-2.00 (m, 2H), 1.92-1.87 (m,

2H), 1.80-1.77 (m, 1H), 1.66-1.56 (m, 2H), 1.51-1.40 (m, 2H), 1.38-1.30 (m, 1H); **^{13}C NMR** (100 MHz, $CDCl_3$) δ 166.79, 148.59, 142.57, 130.13, 129.24, 126.55, 125.08, 123.86, 119.74, 47.38, 32.65, 26.38, 25.95; **HRMS** Calcd for $C_{15}H_{17}ClN$ [M+H$^+$]: 246.1050, Found: 246.1048.

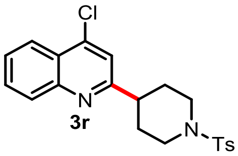

3r R_f = 0.25, 20% Etowah in Hexane

Compound **3r** was prepared following the general procedure (3 h) in 86% yield as a colorless solid. **^{1}H NMR** (400 MHz, $CDCl_3$) δ 8.18 (dd, *J* = 8.3, 0.7 Hz, 1H), 8.00 (d, *J* = 8.4 Hz, 1H), 7.78-7.72 (m, 1H), 7.70 (d, *J* = 8.2 Hz, 2H), 7.62-7.58 (m, 1H), 7.36 (d, *J* = 8.2 Hz, 2H), 7.34 (s, 1H), 3.99-3.94 (m, 2H), 2.84-2.76 (m, 1H), 2.49-2.42 (m, 5H), 2.07-1.98 (m, 4H); **^{13}C NMR** (100 MHz, $CDCl_3$) δ 163.62, 148.55, 143.49, 142.92, 133.25, 130.40, 129.62, 129.33, 127.69, 127.00, 125.17, 123.88, 119.42, 46.27, 44.05, 30.74, 21.51; **HRMS** Calcd for $C_{21}H_{22}ClN_2O_2S$ [M+H$^+$]: 401.1091, Found: 401.1088.

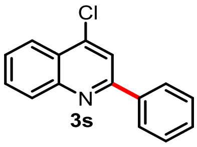

3s R_f = 0.85, 10% EtOAc in Hexane

Compound **1** (32.8 mg, 0.2 mmol, 1.0 equiv)**,** phenyl boronic acid **2t** (36.8 mg, 0.3 mmol, 1.5 equiv), BI-OAc (122.4 mg, 0.4 mmol, 2.0 equiv) and TFA (38.8 mg, 0.4 mmol, 2.0 equiv) were added to a solution of $Ru(bpy)_3Cl_2$ (1.28 mg, 0.002 mmol, 0.01 equiv) in HFIP (0.5 mL). The reaction vial was purged with Ar for 1 min and then the mixture was stirred at 30 ^{o}C under the fluorescent light irradiation for 24 h. The solvent was removed *in vacuo* and the residue was dissolved in DCM (3 mL). To the solution was added K_2CO_3 (approximate 150 mg), the mixture was vigorously stirred for 5 min. After that, the mixture was filtrated through a pad of Celite and washed with DCM. The filtrate was concentrated *in vacuo* and the residue was purified by flash chromatography on silica gel to afford compound **3r** as a colorless solid (10.0 mg, 21% yield). **^{1}H NMR** (400 MHz, $CDCl_3$) δ 8.24 (dd, *J* = 8.3, 0.7 Hz, 1H), 8.20-8.14 (m, 3H), 7.98 (s, 1H), 7.81-7.76 (m, 1H), 7.65-7.61 (m, 1H), 7.56-7.47 (m, 3H). Spectra data are consistent with those reported in the literature.[13]

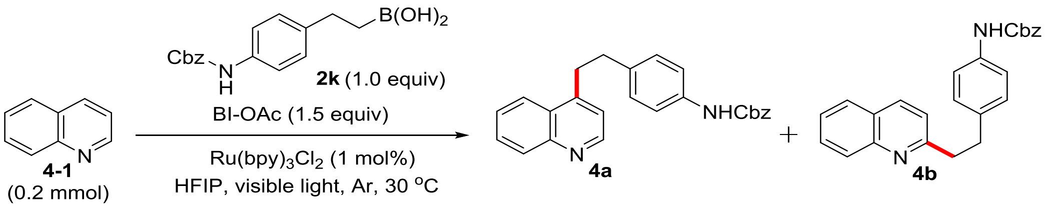

Scheme S11.

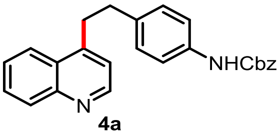

R_f = 0.25, 40% EtOAc in Hexane

Compound **4a** was prepared following the general procedure (**2k** 1.0 equiv, BI-OAc 1.5 equiv, 10 h) in 39% yield as a colorless solid. **^{1}H NMR** (400 MHz, $CDCl_3$) δ 8.77 (d, *J* = 4.4 Hz, 1H), 8.13 (d, *J* = 8.4 Hz, 1H), 8.06 (d, *J* = 8.4 Hz, 1H), 7.72-7.68 (m, 1H), 7.59-7.55 (m, 1H), 7.41-7.31 (m, 7H), 7.14 (d, *J* = 4.5 Hz, 1H), 7.12 (d, *J* = 8.4 Hz, 2H), 6.88 (br s, 1H), 5.20 (s, 2H), 3.36-3.32 (m, 2H), 3.04-3.00 (m, 2H); **^{13}C NMR** (100 MHz, $CDCl_3$) δ 153.38, 150.09, 148.24, 147.32, 136.09, 136.03, 130.22, 129.57, 129.06, 128.92, 128.57, 128.31, 128.26, 127.35, 126.42, 123.32, 120.89, 118.89, 66.94, 35.41, 34.07; **HRMS** Calcd for $C_{25}H_{23}N_2O_2$ [M+H$^+$]: 383.1760, Found: 383.1819.

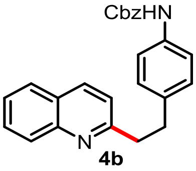

R_f = 0.6, 40% EtOAc in Hexane

Compound **4b** was prepared following the general procedure (**2k** 1.0 equiv, BI-OAc 1.5 equiv, 10 h) in 11% yield as a colorless solid. **^{1}H NMR** (400 MHz, $CDCl_3$) δ 8.06 (d, *J* = 8.6 Hz, 1H), 8.04 (d, *J* = 8.6 Hz, 1H), 7.78 (d, *J* = 8.1 Hz, 1H), 7.72-7.68 (m, 1H), 7.52-7.48 (m, 1H), 7.41-7.28 (m, 7H), 7.21 (d, *J* = 8.4 Hz, 1H), 7.17 (d, *J* = 8.4 Hz, 2H), 6.65 (br s, 1H), 5.19 (s, 2H), 3.28-3.24 (m, 2H), 3.13-3.09 (m, 2H); **^{13}C NMR** (100 MHz, $CDCl_3$) δ 161.70, 147.96, 136.74, 136.20, 136.08, 135.71, 129.39, 129.09, 128.83, 128.60, 128.32, 128.30, 127.52, 126.78, 125.79, 121.58, 118.79, 66.95, 40.98, 35.21; **HRMS** Calcd for $C_{25}H_{23}N_2O_2$ [M+H$^+$]: 383.1760, Found: 383.1819.

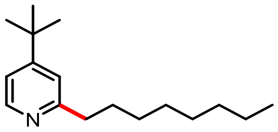

5a

R_f = 0.4, 10% EtOAc in Hexane

Compound **5a** was prepared following the general procedure (48 h) in 52% yield as a colorless oil. **^{1}H NMR** (400 MHz, $CDCl_3$) δ 8.41 (d, *J* = 5.3 Hz, 1H), 7.11 (s, 1H), 7.08 (dd, *J* = 5.3, 1.7 Hz, 1H), 2.76 (t, *J* = 7.8 Hz, 2H), 1.75-1.68 (m, 2H), 1.38-1.26 (m, 19H), 0.87 (t, *J* = 6.7 Hz, 3H); **^{13}C NMR** (100 MHz, $CDCl_3$) δ 162.25, 160.18, 148.94, 119.54, 118.00, 38.66, 34.56, 31.85, 30.55, 30.10, 29.50, 29.45, 29.23, 22.65, 14.09; **HRMS** Calcd for $C_{17}H_{30}N$ [M+H$^+$]: 248.2378, Found: 248.2376.

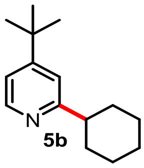

5b

R_f = 0.4, 10% EtOAc in Hexane

Compound **5b** was prepared following the general procedure (16 h) in 53% yield as a colorless oil. **^{1}H NMR** (400 MHz, $CDCl_3$) δ 8.43 (d, *J* = 5.3 Hz, 1H), 7.12 (d, *J* = 1.9 Hz, 1H), 7.09 (dd, *J* = 5.3, 1.9 Hz, 1H),2.71-2.64 (m, 1H), 1.96-1.84 (m, 4H), 1.77-1.73 (m, 1H), 1.60-1.51 (m, 2H), 1.47-1.39 (m, 2H), 1.37-1.27 (m, 10H); **^{13}C NMR** (100 MHz, $CDCl_3$) δ 166.23, 160.18, 148.82, 118.18, 117.90, 46.76, 34.63, 32.99, 30.57, 26.63, 26.05; **HRMS** Calcd for $C_{15}H_{24}N$ [M+H$^+$]: 218.1909, Found: 218.1906.

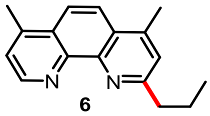

6

R_f = 0.2, 100% EtOAc

Compound **6** was prepared following the general procedure (24 h) in 70% yield as a colorless oil. ^{1}H NMR (400 MHz, $CDCl_3$) δ 9.08 (d, *J* = 4.5 Hz, 1H), 8.01-7.95 (m, 2H), 7.43 (dd, *J* = 4.5, 0.5 Hz, 1H), 7.38 (d, *J* = 0.5 Hz, 1H), 3.17-3.13 (m, 2H), 2.79 (s, 3H), 2.77 (s, 3H), 1.96-1.86 (m, 2H), 1.07 (t, *J* = 7.3 Hz, 3H); ^{13}C NMR (100 MHz, $CDCl_3$) δ 162.91, 149.81, 145.97, 145.75, 144.06, 143.98, 127.90, 126.15, 123.66, 123.59, 122.00, 120.93, 41.46, 23.66, 19.11, 14.20; **HRMS** Calcd for $C_{17}H_{19}N_2$ [M+H$^+$]: 251.1548, Found: 251.1544.

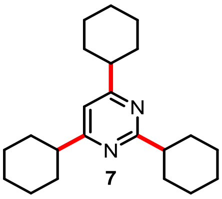

7

R_f = 0.5, 5% EtOAc in Hexane

Compound **7-1** (16.2 mg, 0.2 mmol, 1.0 equiv)**,** boronic acid **2q** (115.2 mg, 0.9 mmol, 4.5 equiv) and BI-OAc (367.4 mg, 1.2 mmol, 6.0 equiv) were added to a solution of $Ru(bpy)_3Cl_2$ (1.28 mg, 0.002 mmol, 0.01 equiv) in HFIP (0.5 mL). The reaction vial was purged with Ar for 1 min and then the mixture was stirred at 30 ^oC under the fluorescent light irradiation for 1 h. The solvent was removed *in vacuo* and the residue was dissolved in DCM (3 mL). To the solution was added K_2CO_3 (approximate 450 mg), the mixture was vigorously stirred for 5 min. After that, the mixture was filtrated through a pad of Celite and washed with DCM. The filtrate was concentrated *in vacuo* and the residue was purified by flash chromatography on silica gel to afford compound **7** as a colorless oil (41.2 mg, 63% yield). **^{1}H NMR** (400 MHz, $CDCl_3$) δ 6.77 (s, 1H), 2.83-2.75 (m, 1H), 2.61-2.55 (m, 2H), 1.95-1.63 (m, 17H), 1.52-1.25 (m, 13H). Spectra data are consistent with those reported in the literature.[14]

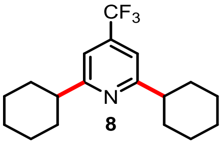

8

R_f = 0.8, 10% EtOAc in Hexane

Compound **8-1** (29.5 mg, 0.2 mmol, 1.0 equiv)**,** boronic acid **2q** (76.6 mg, 0.6 mmol, 3.0 equiv) and BI-OAc (244.8 mg, 0.8 mmol, 4.0 equiv) were added to a solution of $Ru(bpy)_3Cl_2$ (1.28 mg, 0.002 mmol, 0.001 equiv) in HFIP (0.5 mL). The reaction vial was purged with Ar for 1 min and then the mixture was stirred at 30 ^oC under the fluorescent light irradiation for 2 h. The solvent was removed *in vacuo* and the residue was dissolved in DCM (3 mL). To the solution was added K_2CO_3 (approximate 450 mg), the mixture was vigorously stirred for 5 min. After that, the mixture was filtrated through a pad of Celite and washed with DCM. The filtrate was concentrated *in vacuo* and the residue was purified by flash chromatography on silica gel to afford compound **8** as a colorless oil (51.0 mg, 82% yield). **^{1}H NMR** (400 MHz, $CDCl_3$) δ 7.15 (s, 2H), 2.79-2.72 (m, 2H), 1.98-1.74 (m, 10H), 1.56-1.27 (m, 10H); **^{13}C NMR** (100 MHz,

$CDCl_3$) δ 167.14, 138.67 (q, *J* = 32.8 Hz), 123.35 (q, *J* = 272.9 Hz), 138.67 (q, *J* = 32.8 Hz), 46.56, 32.82, 26.41, 25.99; **HRMS** Calcd for $C_{18}H_{25}F_3N$ [M+H$^+$]: 312.1939, Found: 312.1938.

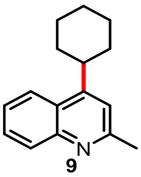
9

R_f = 0.4, 10% EtOAc in Hexane

Compound **9** was prepared following the general procedure (2 h) in 93% yield as a colorless oil. **^{1}H NMR** (400 MHz, $CDCl_3$) δ 8.03 (d, *J* = 9.2 Hz, 2H), 7.66-7.62 (m, 1H), 7.50-7.46 (m, 1H), 7.16 (s, 1H), 3.31-3.26 (m, 1H), 2.71 (s, 3H), 2.01-1.83 (m, 5H), 1.60-1.48 (m, 4H), 1.40-1.28 (m, 1H). Spectra data are consistent with those reported in the literature.[14]

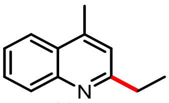
10a

R_f = 0.5, 10% EtOAc in Hexane

Compound **10a** was prepared following the general procedure (24 h) in 80% yield as a colorless oil. **^{1}H NMR** (400 MHz, $CDCl_3$) δ 8.04 (d, *J* = 8.4 Hz, 1H), 7.95 (dd, *J* = 8.3, 0.6 Hz, 1H), 7.69-7.65 (m, 1H), 7.52-7.48 (m, 1H), 7.16 (s, 1H), 2.96 (q, *J* = 7.6 Hz, 2H), 2.68 (s, 3H), 1.39 (t, *J* = 7.6 Hz, 3H). Spectra data are consistent with those reported in the literature.[15]

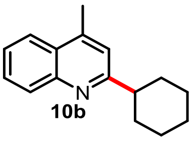
10b

R_f = 0.6, 10% EtOAc in Hexane

Compound **10b** was prepared following the general procedure (1 h) in 96% yield as a colorless oil. **^{1}H NMR** (400 MHz, $CDCl_3$) δ 8.04 (d, *J* = 8.5 Hz, 1H), 7.93 (d, *J* = 8.4 Hz, 1H), 7.68-7.64 (m, 1H), 7.50-7.46 (m, 1H), 7.16 (s, 1H), 2.90-2.84 (m, 1H), 2.67 (s, 3H), 2.01 (d, *J* = 12.7 Hz, 2H), 1.86-1.91 (m, 2H), 1.80-1.76 (m, 1H), 1.67-1.57 (m, 2H), 1.52-1.41 (m, 2H), 1.39-1.30 (m, 1H). Spectra data are consistent with those reported in the literature.[12]

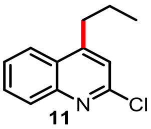

11 $R_f = 0.4$, 10% EtOAc in Hexane

Compound **11** was prepared following the general procedure (24 h) in 62% yield as a colorless solid. **^{1}H NMR** (400 MHz, $CDCl_3$) δ 8.03-7.99 (m, 2H), 7.73-7.69 (m, 1H), 7.58-7.54 (m, 1H), 7.24 (s, 1H), 3.02 (t, J = 7.7 Hz, 2H), 1.86-1.76 (m, 2H), 1.06 (t, J = 7.3 Hz, 3H); **^{13}C NMR** (100 MHz, $CDCl_3$) δ 151.94, 150.61, 148.02, 130.08, 129.31, 126.58, 126.31, 123.62, 121.50, 34.00, 22.96, 14.07; **HRMS** Calcd for $C_{12}H_{13}ClN$ [M+H$^+$]: 206.0737, Found: 206.0773.

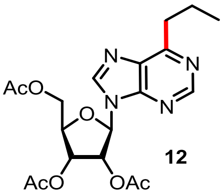

12 $R_f = 0.6$, 100% EtOAc

Compound **12** was prepared following the general procedure (24 h) in 60% yield as a colorless oil. **^{1}H NMR** (400 MHz, $CDCl_3$) δ 8.90 (s, 1H), 8.18 (s, 1H), 6.24 (d, J = 5.1 Hz, 1H), 5.99 (t, J = 5.3 Hz, 1H), 5.71 (t, J = 5.1 Hz, 1H), 4.49-4.46 (m, 2H), 4.41-4.36 (m, 1H), 3.18 (t, J = 7.6 Hz, 2H), 2.16 (s, 3H), 2.13 (s, 3H), 2.10 (s, 3H), 1.98-1.89 (m, 2H), 1.04 (t, J = 7.4 Hz, 3H). Spectra data are consistent with those reported in the literature.[16]

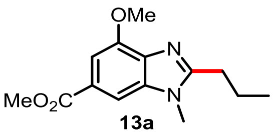

13a $R_f = 0.6$, 100% EtOAc

Compound **13a** was prepared following the general procedure (24 h) in 72% yield as a colorless solid. **^{1}H NMR** (400 MHz, $CDCl_3$) δ 8.06 (s, 1H), 7.37 (s, 1H), 4.00 (s, 3H), 3.98 (s, 3H), 3.93 (s, 3H), 2.83 (t, J = 7.7 Hz, 2H), 1.94-1.84 (m, 2H), 1.07 (t, J = 7.4 Hz, 3H); **^{13}C NMR** (100 MHz, $CDCl_3$) δ 167.78, 156.72, 146.39, 143.42, 128.36, 124.09, 115.03, 104.09, 55.72, 52.06, 32.40, 29.25, 20.86, 13.96; **HRMS** Calcd for $C_{14}H_{19}N_2O_3$ [M+H$^+$]: 263.1396, Found: 263.1391.

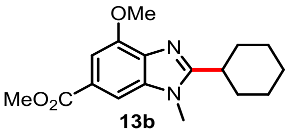

13b $R_f = 0.5$, 50% EtOAc in Hexane

Compound **13b** was prepared following the general procedure (1 h) in 90% yield as a colorless solid. **^{1}H NMR** (400 MHz, $CDCl_3$) δ 8.10 (d, *J* = 1.2 Hz, 1H), 7.36 (d, *J* = 1.2 Hz, 1H), 4.02 (s, 3H), 3.98 (s, 3H), 3.92 (m, 3H), 2.84-2.77 (m, 1H), 2.00-1.90 (m, 4H), 1.82-1.73 (m, 4H), 1.47-1.34 (m, 2H); **^{13}C NMR** (100 MHz, $CDCl_3$) δ 167.83, 160.47, 146.45, 143.64, 128.33, 123.93, 115.31, 104.03, 55.73, 52.04, 36.02, 32.14, 31.32, 26.25, 25.76; **HRMS** Calcd for $C_{17}H_{23}N_2O_3$ [M+H$^+$]: 303.1709, Found: 303.1708.

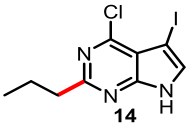

14 R_f = 0.5, 50% EtOAc in Hexane

Compound **14** was prepared following the general procedure (24 h) in 62% yield as light yellow solid. **^{1}H NMR** (400 MHz, $CDCl_3$) δ 11.63 (s, 1H), 8.60 (s, 1H), 2.91 (t, *J* = 7.6 Hz, 2H), 1.87-1.78 (m, 2H), 1.06 (t, *J* = 7.3 Hz, 3H); **^{13}C NMR** (100 MHz, $CDCl_3$) δ 151.93, 151.65, 149.24, 144.13, 117.97, 53.31, 31.08, 22.13, 13.73; **HRMS** Calcd for $C_9H_{10}ClIN_3$ [M+H$^+$]: 321.9608, Found: 321.9605.

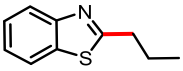

15a R_f = 0.65, 20% EtOAc in Hexane

Compound **15a** was prepared following the general procedure (24 h) in 82% yield as colorless oil. **^{1}H NMR** (400 MHz, $CDCl_3$) δ 7.97 (d, *J* = 7.9 Hz, 1H), 7.84 (dd, *J* = 8.0, 0.6 Hz, 1H), 7.47-7.43 (m, 1H), 7.36-7.32 (m, 1H), 3.10 (t, *J* = 7.6 Hz, 2H), 1.96-1.87 (m, 2H), 1.06 (t, *J* = 7.4 Hz, 3H). Spectra data are consistent with those reported in the literature.[17]

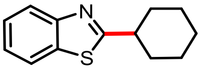

15b R_f = 0.65, 20% EtOAc in Hexane

Compound **15b** was prepared following the general procedure (2 h) in 88% yield as colorless oil. **^{1}H NMR** (400 MHz, $CDCl_3$) δ 7.97 (d, *J* = 8.1 Hz, 1H), 7.84 (dd, *J* = 8.0, 0.4 Hz, 1H), 7.46-7.42 (m, 1H), 7.35-7.31 (m, 1H), 3.14-3.07 (m, 1H), 2.22-2.18 (m, 2H), 1.91-1.86 (m, 2H), 1.78-1.74 (m, 1H), 1.69-1.59 (m, 2H), 1.50-1.39 (m, 2H), 1.37-1.29 (m, 1H). Spectra data are consistent with those reported in the literature.[18]

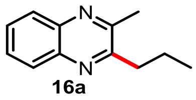

R_f = 0.65, 20% EtOAc in Hexane

Compound **16a** was prepared following the general procedure (24 h) in 64% yield as colorless oil. **^{1}H NMR** (400 MHz, $CDCl_3$) δ 8.03-7.96 (m, 2H), 7.69-7.65 (m, 2H), 2.99 (t, J = 7.8 Hz, 2H), 2.77 (s, 3H), 1.92-1.83 (m, 2H), 1.09 (t, J = 7.4 Hz, 3H). Spectra data are consistent with those reported in the literature.[19]

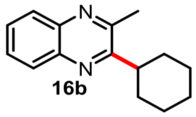

R_f = 0.4, 10% EtOAc in Hexane

Compound **16b** was prepared following the general procedure (10 h) in 78% yield as colorless oil. **^{1}H NMR** (400 MHz, $CDCl_3$) δ 8.03-7.99 (m, 1H), 7.98-7.94 (m, 1H), 7.66-7.62 (m, 2H), 3.07-3.00 (m, 1H), 2.79 (s, 3H), 1.96-1.91 (m, 4H), 1.82-1.73 (m, 3H), 1.52-1.36 (m, 3H). Spectra data are consistent with those reported in the literature.[14]

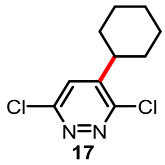

R_f = 0.7, 20% EtOAc in Hexane

Compound **17** was prepared following the general procedure (2 h) in 65% yield as colorless solid. **^{1}H NMR** (400 MHz, $CDCl_3$) δ 7.36 (s, 1H), 2.91-2.83 (m, 1H), 1.98-1.90 (m, 4H), 1.85-1.81 (m, 1H), 1.52-1.41 (m, 2H), 1.37-1.26 (m, 3H). Spectra data are consistent with those reported in the literature.[20]

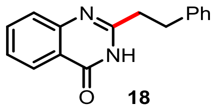

R_f = 0.6, 100% EtOAc

Compound **18** was prepared following the general procedure (24 h) in 67% yield as colorless solid. **^{1}H NMR** (400 MHz, $CDCl_3$) δ 12.0 (br s, 1H), 8.31 (dd, J = 7.9, 0.9 Hz, 1H), 7.83-7.78 (m, 1H), 7.75 (d, J = 7.7 Hz, 1H), 7.52-7.48 (m, 1H), 7.35 (d, J = 7.2 Hz, 2H), 7.31-7.27 (m,

2H), 7.23-7.16 (m, 1H), 3.23-3.19 (m, 2H), 3.12-3.08 (m, 2H). Spectra data are consistent with those reported in the literature.[21]

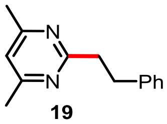

19 R_f = 0.5, 50% EtOAc in Hexane

Compound **19** was prepared following the general procedure (24 h) in 64% yield as colorless oil. **^{1}H NMR** (400 MHz, $CDCl_3$) δ 7.28 (d, *J* = 4.4 Hz, 4H), 7.21-7.16 (m, 1H), 6.87 (s, 1H), 3.22-3.17 (m, 2H), 3.14-3.09 (m, 2H), 2.46 (s, 6H); **^{13}C NMR** (100 MHz, $CDCl_3$) δ 169.69, 166.53, 141.65, 128.47, 128.27, 125.83, 117.48, 41.27, 34.97, 23.98; **HRMS** Calcd for $C_{14}H_{17}N_2$ [M+H$^+$]: 213.1392, Found: 213.1423.

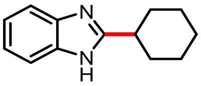

20 R_f = 0.8, 100% EtOAc

Compound **20** was prepared following the general procedure (10 h) in 76% yield as colorless solid. ^{1}H NMR (400 MHz, CD_3OD) δ 7.50-7.46 (m, 2H), 7.19-7.15 (m, 2H), 2.93-2.86 (m, 1H), 2.10-2.06 (m, 2H), 1.91-1.87 (m, 2H), 1.81-1.77 (m, 1H), 1.69-1.62 (m, 2H), 1.53-1.42 (m, 2H), 1.40-1.31 (m, 1H). Spectra data are consistent with those reported in the literature.[14]

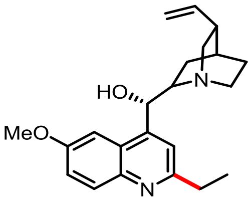

21a R_f = 0.4, 5% MeOH in DCM

Compound **21a** was prepared following the general procedure (24 h) in 81% yield as colorless solid. **^{1}H NMR** (400 MHz, $CDCl_3$) δ 7.90 (d, *J* = 9.2 Hz, 1H), 7.41 (s, 1H), 7.25 (d, *J* = 2.3 Hz, 1H), 7.14 (s, 1H), 5.75-5.67 (m, 1H), 5.54 (s, 1H), 4.97-4.90 (m, 2H), 3.84 (s, 3H), 3.54 (br s, 1H), 3.13-3.07 (m, 1H), 2.85 (q, *J* = 7.6 Hz, 2H), 2.76-2.62 (m, 2H), 2.28 (br s, 1H), 1.81-1.68 (m, 3H), 1.56-1.43 (m, 3H), 1.29 (t, *J* = 7.6 Hz, 3H); **^{13}C NMR** (100 MHz, $CDCl_3$) δ 161.01, 157.10, 147.62, 143.83, 141.62, 130.84, 124.81, 121.05, 118.01, 114.49, 101.27, 71.74, 59.92,

56.91, 55.62, 43.28, 39.83, 32.03, 27.85, 27.43, 21.43, 14.03; **HRMS** Calcd for $C_{22}H_{29}N_2O_2$ [M+H$^+$]: 353.2229, Found: 353.2228.

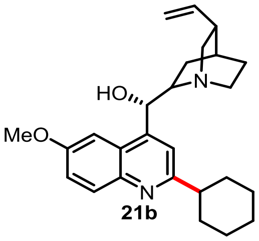

21b R$_f$ = 0.4, 5% MeOH in DCM

Compound **21b** was prepared following the general procedure (8 h) in 90% yield as colorless solid. **^{1}H NMR** (400 MHz, $CDCl_3$) δ 7.95 (d, *J* = 9.2 Hz, 1H), 7.49 (s, 1H), 7.30 (d, *J* = 9.2 Hz, 1H), 7.19 (s, 1H), 5.77-5.67 (m, 2H), 4.99-4.91 (m, 2H), 3.87 (s, 3H), 3.65-3.52 (m, 1H), 3.19-3.11 (m, 2H), 2.87-2.81 (m, 1H), 2.78-2.67 (m, 2H), 2.36-2.26 (m, 1H), 2.00-1.91 (m, 2H), 1.84-1.74 (m, 6H), 1.63-1.26 (m, 9H); **^{13}C NMR** (100 MHz, $CDCl_3$) δ 163.90, 157.18, 147.46, 143.74, 141.44, 131.06, 124.94, 120.99, 116.70, 114.60, 101.23, 71.63, 59.83, 56.86, 55.71, 47.29, 43.34, 39.74, 32.81, 27.87, 27.28, 26.46, 25.98, 20.92; **HRMS** Calcd for $C_{26}H_{35}N_2O_2$ [M+H$^+$]: 407.2699, Found: 407.2698.

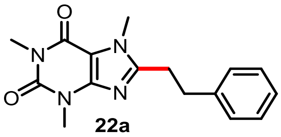

22a R$_f$ = 0.45, 5% MeOH in DCM

Compound **22a** was prepared following the general procedure (24 h) in 42% yield as colorless solid. **^{1}H NMR** (400 MHz, $CDCl_3$) δ 7.31-7.21 (m, 4H), 7.13 (d, *J* = 7.0 Hz, 2H), 3.61 (s, 3H), 3.60 (s, 3H), 3.39 (s, 3H), 3.11-3.08 (m, 2H), 3.04-3.00 (m, 2H); **^{13}C NMR** (100 MHz, $CDCl_3$) δ 155.26, 153.34, 151.67, 148.00, 139.97, 128.72, 128.31, 126.69, 107.15, 34.03, 31.39, 29.72, 28.95, 27.85; **HRMS** Calcd for $C_{16}H_{19}N_4O_2$ [M+H$^+$]: 299.1508, Found: 299.1508.

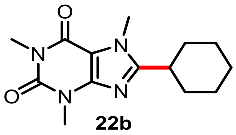

22b R$_f$ = 0.5, 5% MeOH in DCM

Compound **22b** was prepared following the general procedure (12 h) in 50% yield as colorless solid. ^{1}H NMR (400 MHz, $CDCl_3$) δ 3.93 (s, 3H), 3.57 (s, 3H), 3.40 (s, 3H), 2.75-2.68 (m, 1H),

1.92-1.85 (m, 4H), 1.78-1.64 (m, 3H), 1.44-1.31 (m, 3H). Spectra data are consistent with those reported in the literature.[14]

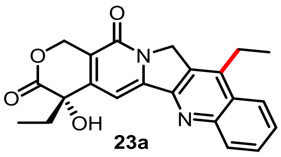

23a R_f = 0.45, 5% MeOH in DCM

(*S*)-camptothecin **23-1** (69.6 mg, 0.2 mmol, 1.0 equiv)**,** ethyl boronic acid **2c** (22.5 mg, 0.3 mmol, 1.5 equiv) and BI-OAc (122.4 mg, 0.4 mmol, 2.0 equiv) were added to a solution of $Ru(bpy)_3Cl_2$ (1.28 mg, 0.002 mmol, 0.01 equiv) in HFIP (0.5 mL). The reaction vial was purged with Ar for 1 min and then the mixture was stirred at 30 ^{o}C under the fluorescent light irradiation for 48 h. The solvent was removed *in vacuo* and the residue was purified by preparative thin layer chromatography to afford compound **23a** as a light yellow solid (45.2 mg, 60% yield). ^{1}H NMR (400 MHz, $CDCl_3$) δ 8.24 (d, *J* = 8.4 Hz, 1H), 8.14 (d, *J* = 8.4 Hz, 1H), 7.84-7.79 (m, 1H), 7.70-7.67 (m, 2H), 5.77 (d, *J* = 16.2 Hz, 1H), 5.32 (d, *J* = 16.2 Hz, 1H), 5.27 (s, 2H), 3.74 (s, 1H), 3.22 (q, *J* = 7.7 Hz, 2H), 1.96-1.84 (m, 2H), 1.42 (t, *J* = 7.7 Hz, 3H), 1.05 (t, *J* = 7.4 Hz, 3H). Spectra data are consistent with those reported in the literature.[22]

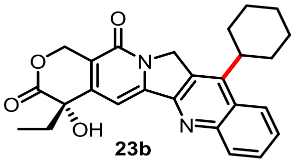

23b R_f = 0.45, 5% MeOH in DCM

(*S*)-camptothecin **23-1** (69.6 mg, 0.2 mmol, 1.0 equiv)**,** alkyl boronic acid **2q** (38.6 mg, 0.3 mmol, 1.5 equiv) and BI-OAc (122.4 mg, 0.4 mmol, 2.0 equiv) were added to a solution of $Ru(bpy)_3Cl_2$ (1.28 mg, 0.002 mmol, 0.01 equiv) in HFIP (0.5 mL). The reaction vial was purged with Ar for 1 min and then the mixture was stirred at 30 ^{o}C under the fluorescent light irradiation for 5 h. The solvent was removed *in vacuo* and the residue was purified by preparative thin layer chromatography to afford compound **23b** as a light yellow solid (78.1 mg, 91% yield). ^{1}H NMR (400 MHz, $CDCl_3$) δ 8.26-8.20 (m, 2H), 7.79 (t, *J* = 7.5 Hz, 1H), 7.69-7.04 (m, 2H), 5.76 (d, *J* = 16.2 Hz, 1H), 5.42 (s, 2H), 5.31 (d, *J* = 16.2 Hz, 1H), 3.81 (s,

1H), 3.66 (br s, 1H), 2.02-1.82 (m, 9H), 1.65-1.40 (m, 3H), 1.04 (t, J = 7.3 Hz, 3H). Spectra data are consistent with those reported in the literature.[23]

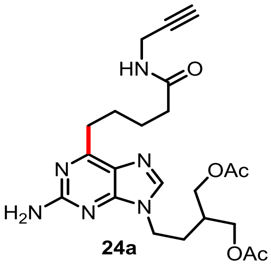

24a R_f = 0.25, 5% MeOH in DCM

Compound **24a** was prepared following the general procedure (7 h) in 51% yield as colorless solid. **^{1}H NMR** (400 MHz, $CDCl_3$) δ 7.74 (s, 1H), 6.19 (br s, 1H), 5.00 (br s. 2H), 4.19 (t, J = 7.1 Hz, 2H), 4.14 (d, J = 5.4 Hz, 4H), 4.04 (dd, J = 5.1, 2.5 Hz, 2H), 3.02 (t, J = 7.4 Hz, 2H), 2.30 (t, J = 7.5 Hz, 2H), 2.23 (t, J = 2.5 Hz, 1H), 2.07 (s, 6H), 2.05-1.86 (m, 5H), 1.77-1.70 (m, 2H); **^{13}C NMR** (100 MHz, $CDCl_3$) δ 172.52, 170.90, 163.29, 159.63, 152.64, 140.80, 126.83, 79.87, 71.44, 63.62, 40.88, 35.92, 34.90, 32.31, 29.05, 28.78, 27.47, 25.11, 20.84; **HRMS** Calcd for $C_{22}H_{31}N_6O_5$ [M+H$^+$]: 459.2356, Found: 459.2355.

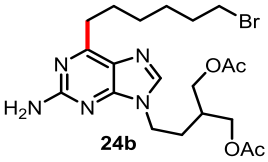

24b R_f = 0.6, 5% MeOH in DCM

Compound **24b** was prepared following the general procedure (5 h) in 65% yield as light brown oil. **^{1}H NMR** (400 MHz, $CDCl_3$) δ 7.72 (s, 1H), 4.97 (br s, 2H), 4.19 (t, J = 7.1 Hz, 2H), 4.14 (d, J = 5.4 Hz, 4H), 3.39 (t, J = 6.9 Hz, 2H), 2.99 (t, J = 7.8 Hz, 2H), 2.07 (s, 6H), 2.04-1.92 (m, 3H), 1.89-1.81 (m, 4H), 1.53-1.40 (m, 4H); **^{13}C NMR** (100 MHz, $CDCl_3$) δ 170.84, 163.74, 159.58, 152.55, 140.61, 126.71, 63.59, 40.79, 34.83, 33.85, 33.11, 32.60, 28.79, 28.69, 28.24, 27.91, 20.80; **HRMS** Calcd for $C_{20}H_{31}BrN_5O_4$ [M+H$^+$]: 484.1559, Found: 484.1562.

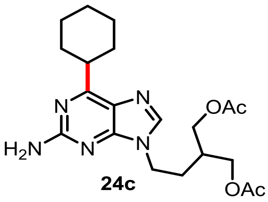

24c R_f = 0.3, 5% MeOH in DCM

Compound **24c** was prepared following the general procedure (4 h) in 50% yield as colorless solid. **^{1}H NMR** (400 MHz, $CDCl_3$) δ 7.71 (s, 1H), 5.02 (br s, 2H), 4.18 (t, J = 7.1 Hz, 2H), 4.14 (d, J = 5.4 Hz, 4H), 3.26-3.18 (m, 1H), 2.06 (s, 6H), 2.03-1.75 (m, 10H), 1.50-1.34 (m, 3H); **^{13}C NMR** (100 MHz, $CDCl_3$) δ 170.83, 167.66, 159.71, 152.66, 140.21, 125.83, 63.62, 42.26, 40.73, 34.82, 31.06, 28.80, 26.22, 25.90, 20.79; **HRMS** Calcd for $C_{20}H_{30}N_5O_4$ [M+H$^+$]: 404.2298, Found: 404.2295.

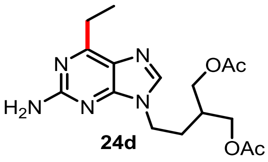

R_f = 0.3, 5% MeOH in DCM

Compound **24d** was prepared following the general procedure (4 h) in 70% yield as colorless oil. **^{1}H NMR** (400 MHz, $CDCl_3$) δ 7.71 (s, 1H), 4.95 (s, 2H), 4.19 (t, J = 7.1 Hz, 2H), 4.14 (d, J = 5.3 Hz, 4H), 3.03 (q, J = 7.6 Hz, 2H), 2.06 (s, 6H), 2.02-1.90 (m, 3H), 1.39 (t, J = 7.6 Hz, 3H); **^{13}C NMR** (100 MHz, $CDCl_3$) δ 170.83, 164.98, 159.66, 152.56, 140.53, 126.37, 63.61, 40.78, 34.84, 28.80, 26.40, 20.78, 12.57; **HRMS** Calcd for $C_{16}H_{24}N_5O_4$ [M+H$^+$]: 350.1828, Found: 350.1825.

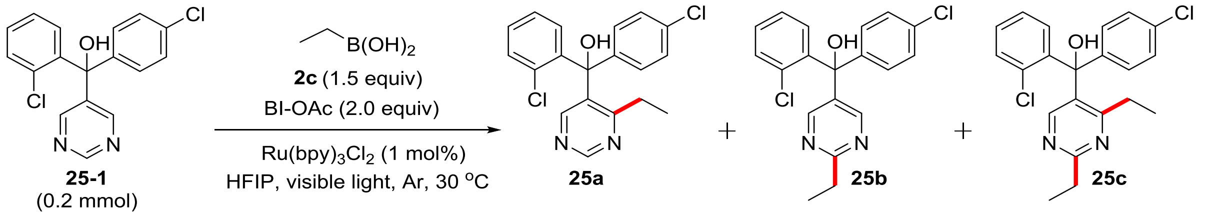

Scheme S12.

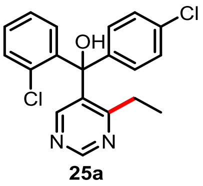

R_f = 0.4, 50% EtOAc in Hexane

Compound **25a** was prepared following the general procedure (24 h) in 11% yield as colorless solid. **^{1}H NMR** (400 MHz, $CDCl_3$) δ 9.10 (s, 1H), 7.91 (s, 1H), 7.48 (dd, J = 7.9, 1.2 Hz, 1H), 7.38-7.34 (m, 3H), 7.21-7.17 (m, 3H), 6.67 (dd, J = 7.9, 1.5 Hz, 1H), 4.59 (s, 1H), 2.81-2.67 (m, 2H), 1.14 (t, J = 7.4 Hz, 3H); **^{13}C NMR** (100 MHz, $CDCl_3$) δ 172.45, 157.70, 155.83,

141.95, 141.10, 135.71, 134.26, 132.60, 131.95, 130.64, 130.04, 129.20, 128.67, 127.06, 81.23, 29.96, 12.70; **HRMS** Calcd for $C_{19}H_{17}Cl_2N_2O$ [M+H$^+$]: 359.0718, Found: 359.0719.

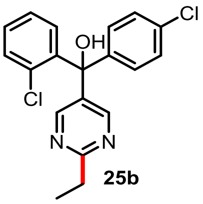

25b R$_f$ = 0.5, 50% EtOAc in Hexane

Compound **25b** was prepared following the general procedure (24 h) in 14% yield as colorless solid. ^{1}**H NMR** (400 MHz, $CDCl_3$) δ 8.52 (s, 2H), 7.44 (dd, *J* = 7.9, 1.0 Hz, 1H), 7.35-7.31 (m, 3H), 7.22-7.18 (m, 3H), 6.76 (dd, *J* = 7.9, 1.4 Hz, 1H), 4.46 (s, 1H), 3.02 (q, *J* = 7.6 Hz, 2H), 1.38 (t, *J* = 7.6 Hz, 3H); ^{13}**C NMR** (100 MHz, $CDCl_3$) δ 171.24, 156.37, 142.47, 141.34, 135.09, 134.10, 132.72, 131.82, 130.69, 130.06, 128.78, 128.65, 126.98, 79.77, 32.26, 12.43; **HRMS** Calcd for $C_{19}H_{17}Cl_2N_2O$ [M+H$^+$]: 359.0718, Found: 359.0719.

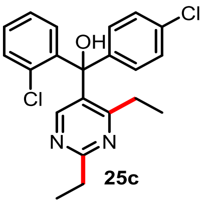

25c R$_f$ = 0.7, 50% EtOAc in Hexane

Compound **25c** was prepared following the general procedure (24 h) in 16% yield as colorless solid. ^{1}**H NMR** (400 MHz, $CDCl_3$) δ 7.80 (s, 1H), 7.45 (dd, *J* = 7.9, 0.9 Hz, 1H), 7.36-7.30 (m, 3H), 7.19-7.16 (m, 3H), 6.69 (dd, *J* = 7.9, 1.3 Hz, 1H), 4.56 (s, 1H), 2.97 (q, *J* = 7.6 Hz, 2H), 2.69 (q, *J* = 7.4 Hz, 2H), 1.37 (t, *J* = 7.6 Hz, 3H), 1.12 (t, *J* = 7.4 Hz, 3H); ^{13}**C NMR** (100 MHz, $CDCl_3$) δ 170.90, 156.12, 142.37, 141.39, 134.06, 132.65, 132.40, 131.85, 130.68, 129.86, 129.20, 128.55, 126.94, 81.21, 32.16, 30.00, 13.01, 12.41; **HRMS** Calcd for $C_{21}H_{21}Cl_2N_2O$ [M+H$^+$]: 387.1031, Found: 387.1028.

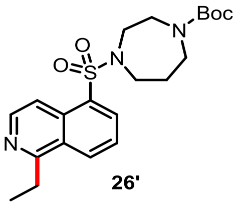

26' R$_f$ = 0.3, 50% EtOAc in Hexane

Fasudil (58.4 mg, 0.2 mmol, 1.0 equiv, prepared from commercial available fasudil hydrochloride by neutralization), ethyl boronic acids **2c** (24.5 mg, 0.3 mmol, 1.5 equiv) and BI-OAc (126.0 mg, 0.4 mmol, 2.0 equiv) were added to a solution of $Ru(bpy)_3Cl_2$ (1.8 mg, 0.002 mmol, 0.01 equiv) in HFIP (0.5 mL). The reaction vial was purged with Ar for 1 min and then the mixture was stirred at 30 ^oC under the fluorescent light irradiation for 10 h. The solvent was removed *in vacuo* and the residue was dissolved in DCM (3 mL). To the solution was added K_2CO_3 (approximate 300 mg), the mixture was vigorously stirred for 5 min. After that, the mixture was filtrated through a pad of Celite and washed with DCM. The filtrate was concentrated *in vacuo* and the residue was dissolved in DCM (5 mL). To the solution was added Boc_2O (87.5 mg, 0.4 mmol, 2.0 equiv) and TEA (61.0 mg, 0.6 mmol, 3.0 equiv), then the mixture was stirred at room temperature for 2 h. The solvent was removed *in vacuo* and the residue was purified by flash chromatography on silica gel (ethyl acetate/petroleum ether = 50:50) to afford compound **26'** (51.2 mg, 61% for 2 steps) as a colorless oil. **^{1}H NMR** (400 MHz, CD_3OD) δ 8.62 (d, *J* = 8.5 Hz, 1H), 8.49 (d, *J* = 6.3 Hz, 1H), 8.40-8.35 (m, 3H), 7.84-7.80 (m, 1H), 3.55-3.38 (m, 10H), 1.90-1.85 (m, 2H), 1.44 (s, 9H), 1.42 (t, *J* = 8.4 Hz, 3H); **^{13}C NMR** (100 MHz, CD_3OD) δ 164.45, 155.50, 155.35, 141.80, 141.76, 135.27, 132.66, 132.33, 131.09, 127.30, 126.28, 116.59, 80.06, 79.93, 48.62, 48.36, 47.97, 47.94, , 47.02, 45.76, 45.45, 28.79, 28.63, 28.08, 27.27, 27.24, 13.11; **HRMS** Calcd for $C_{17}H_{22}N_3O_4S$ [M–*t*-Bu+2H$^+$]: 364.1331, Found: 364.1436.

7. Mechanism study

7.1 Synthesis of compound 27

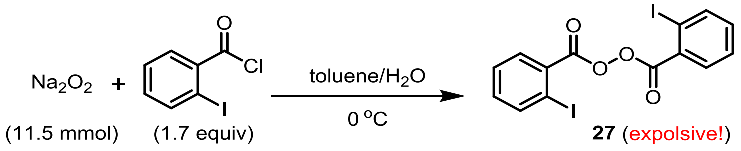

Scheme S13.

Compound **27** was prepared following a reported procedure[24]: to the solution of *o*-iodobenzoyl chloride (5.2 g, 19.5 mmol, 1.7 equiv) in toluene (5 mL) was added a solution of sodium peroxide (0.9 g, 11.5 mmol, 1.0 equiv) in ice-water (12 mL) dropwise at 0 ^oC. After addition completed, the mixture was stirred for 1 h at 0 ^oC. The precipitate was collected by filtration

and the solid product was quickly washed with water, methanol, sodium bicarbonate and again with water and methanol. The solid was then dissolved with chloroform at room temperature. To the clear solution was added cold methanol at 0 °C as soon as possible under stirring (**NOTE**: If the addition of the methanol was delayed, no compound **27** was obtained.) The precipitated peroxide was allowed to stand at 0 °C only until the mother liquor is clear. After filtration, compound **27** was obtained as a colorless crystals (1.3 g, 23%). Spectra data are consistent with those reported in the literature.[24] (**NOTE**: Compound **27** is explosive, all the operations above were performed behind a blast shield.)

7.2 Minisci C-H alkylation promoted by BPO

7.2.1 Minisci C-H alkylation with compound 2a promoted by BPO

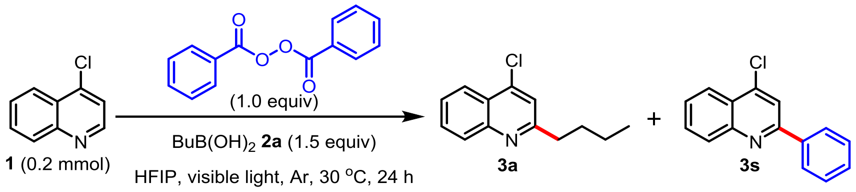

Scheme S14.

Compound **1** (32.8 mg, 0.2 mmol, 1.0 equiv)**,** alkyl boronic acids **2a** (30.8 mg, 0.3 mmol, 1.5 equiv) and BPO (benzoyl peroxide, 48.6 mg, 0.2 mmol, 1.0 equiv) were added to HFIP (0.5 mL). The reaction vial was purged with Ar for 1 min and then the mixture was stirred at 30 °C under the fluorescent light irradiation for 24 h. The solvent was removed *in vacuo* and the residue was dissolved in DCM (3 mL). To the solution was added K_2CO_3 (approximate 150 mg), the mixture was vigorously stirred for 5 min. Filtrated over a Celite plug and the residue was washed with DCM. The filtrate was concentrated *in vacuo* and the residue was purified by preparative thin layer chromatography to afford compound **3a** as a colorless oil in 20% yield. Compound **3s** was isolated in 11% yield (5.1 mg) as a colorless oil.

7.2.2 Minisci C-H alkylation with compound 2b promoted by BPO

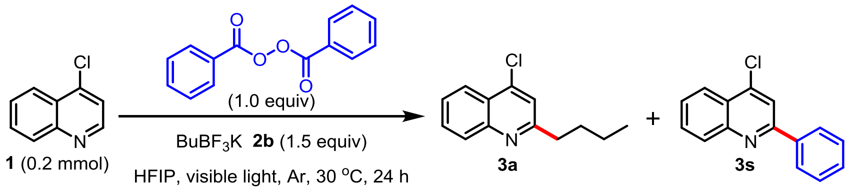

Scheme S15.

Compound **1** (32.8 mg, 0.2 mmol, 1.0 equiv)**,** compound **2b** (48.6 mg, 0.3 mmol, 1.5 equiv) and BPO (benzoyl peroxide, 48.8 mg, 0.2 mmol, 1.0 equiv) were added to HFIP (0.5 mL). The reaction vial was purged with Ar for 1 min and then the mixture was stirred at 30 °C under the fluorescent light irradiation for 24 h. The solvent was removed *in vacuo* and the residue was dissolved in DCM (3 mL). To the solution was added K_2CO_3 (approximate 150 mg), the mixture was vigorously stirred for 5 min. Filtrated over a Celite plug and the residue was washed with DCM. The filtrate was concentrated *in vacuo* and the residue was purified by preparative thin layer chromatography to afford compound **3a** as a colorless oil (~2.1 mg, ~5% yield). Compound **3s** was isolated in 7% yield (3.4 mg) as a colorless oil.

7.3 Minisci C-H alkylation promoted by compound 27

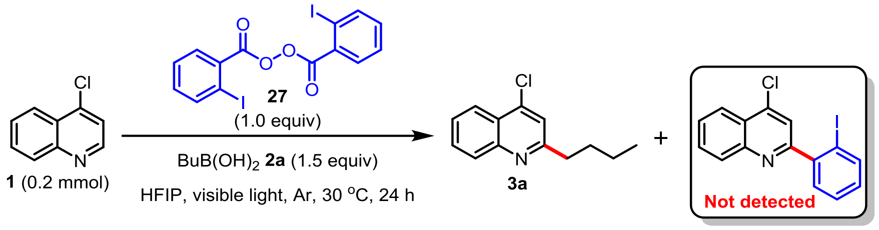

Scheme S16.

Compound **1** (32.8 mg, 0.2 mmol, 1.0 equiv)**,** alkyl boronic acids **2a** (30.6 mg, 0.3 mmol, 1.5 equiv) and compound **27** (100.0 mg, 0.2 mmol, 1.0 equiv) were added to HFIP (0.5 mL). The reaction vial was purged with Ar for 1 min and then the mixture was stirred at 30 °C under the fluorescent light irradiation for 24 h. The solvent was removed *in vacuo* and the residue was dissolved in DCM (3 mL). To the solution was added K_2CO_3 (approximate 150 mg), the mixture was vigorously stirred for 5 min. Filtrated over a Celite plug and the residue was washed with DCM. The filtrate was concentrated *in vacuo* and the residue was purified by preparative thin layer chromatography to afford compound **3a** as a colorless oil (16.7 mg, 38% yield). 16.1 mg of starting material compound **1** was recovered. Conversion based on recovered starting material compound **1**: 51%.

8. DFT calculations

8.1 Computational details

All DFT calculations were performed with the Gaussian 09 software package.[25] Geometries were optimized using the M06-2X functional and the 6-31+G(d) basis set in the gas phase.

Single point energies were calculated using M06-2X and 6-311++G(d,p) and the SMD solvation model in HFIP. Since the solvent parameters for HFIP are not available in Gaussian 09. The parameters of isopropanol were used and the dielectric constant of the solvent was modified to the dielectric constant of HFIP[2] (ε = 16.7) by using the "scrf=(smd,solvent=2-propanol,read)" keywords in the Gaussian 09 calculations. The reported Gibbs free energies and enthalpies include zero-point vibrational energies and thermal corrections computed at 298 K. All reported Gibbs free energies and enthalpies are corrected to the standard concentration in solution (1 mol/L).

The geometry of the key reaction intermediate, the *ortho*-iodobenzoyloxy radical **BI-2**, was also optimized using a few different levels of theories to evaluate the effects of density functional method, basis set, and solvation models on the weak intramolecular I···O interaction. The optimized I···O distance is highly dependent upon the choice of density functional, basis set. As expected, calculations without diffuse functions significantly overestimate the I···O distance (entries 1 and 4). B3LYP and ωB97X-D also predict longer I···O distance than M06-2X when using the same basis set (entries 1, 9 versus 5). Interestingly, calculations using the 6-31+G(d) and 6-311++G(d,p) basis sets give almost identical results (entries 2 versus 3, 5 versus 6). Replacing the SDD basis set with the LANL2DZ basis set for iodine leads to slight decrease of the I···O distance (entries 7 and 8). Finally, using solvation model in the geometry optimization also noticeably decreases the I···O distance (entries 10-12).

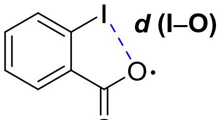

BI-2

entry	method for geometry optimization	*d*(I−O) / Å
1	B3LYP/6-31G(d)-SDD	2.81
2	B3LYP/6-31+G(d)-SDD	2.71
3	B3LYP/6-311++G(d,p)-SDD	2.71
4	M06-2X/6-31G(d)-SDD	2.78
5	M06-2X/6-31+G(d)-SDD	2.59
6	M06-2X/6-311++G(d,p)-SDD	2.59
7	M06-2X/6-31+G(d)-LANL2DZ	2.56
8	M06-2X/6-311++G(d,p)-LANL2DZ	2.56

9	ωB97X-D/6-31+G(d)-SDD	2.68
10	M06-2X/6-31G(d)-SDD/SMD(HFIP)	2.50
11	M06-2X/6-31+G(d)-SDD/SMD(HFIP)	2.47
12	M06-2X/6-311++G(d,p)-SDD/SMD(HFIP)	2.48

Table S2. Geometry of Bl-2 optimized at different levels of theories.

8.2 The reaction energy of the one electron reduction of Bl-OAc using the excited state Ru(II)*(bpy)$_3^{2+}$ *

The photoexicted Ru(II)*(bpy)$_3^{2+}$ is a potent reductant with an experimental standard reduction potential (SRP) of −0.81 V vs. SCE in MeCN.[26] Thus, the absolute SRP in MeCN can be calculated as follows.[27]

$$E^{\ominus,\mathrm{abs}}_{\mathrm{Ru(bpy)}_3^{3+/2+*}} = E^{\ominus,\mathrm{SCE}}_{\mathrm{Ru(bpy)}_3^{3+/2+*}} + 4.429\mathrm{V} = -0.81\mathrm{V} + 4.429\mathrm{V} = 3.62\mathrm{V}$$

Since

$$\Delta G = -nFE$$

where n is the number of electron transferred (one), F is the Faraday constant, the Gibbs free energy of the following reduction half reaction is

$$\mathrm{Ru(bpy)}_3^{3+} + e^- \rightarrow \mathrm{Ru^*(bpy)}_3^{2+} \qquad \Delta G_{\mathrm{MeCN}} = -83.5\ \mathrm{kcal/mol}\ (1)$$

The reaction Gibbs free energies of the following reduction half reactions are calculated using the DFT-computed Gibbs free energy in HFIP solution (see Computational Details) and the Gibbs free energy of electron (−0.867 kcal/mol).[28]

Bl-OAc + e^- → **Bl-1** $\Delta G_{\mathrm{HFIP}} = -102.2$ kcal/mol (2)

Bl-OAc + e^- → **Bl-2** + AcO$^-$ $\Delta G_{\mathrm{HFIP}} = -107.7$ kcal/mol (3)

Bl-OAc + e^- → **Bl-3** + AcO $\Delta G_{\text{HFIP}} = -99.9$ kcal/mol (4)

The reaction energies of the SET reactions (eq. 5-7) can be calculated from equations 1-4. Since the experimental reduction potential of $\text{Ru(bpy)}_3^{3+}/\text{Ru*(bpy)}_3^{2+}$ in HFIP is not available, the reduction half reaction energy in MeCN (eq. 1) was used in the calculations.

Ru*(bpy)_3^{2+} + **Bl-OAc** → Ru(bpy)_3^{3+} + **Bl-1** $\Delta G_{\text{HFIP}} = -18.7$ kcal/mol (5)

Ru*(bpy)_3^{2+} + **Bl-OAc** → Ru(bpy)_3^{3+} + **Bl-2** + AcO^- $\Delta G_{\text{HFIP}} = -24.2$ kcal/mol (6)

Ru*(bpy)_3^{2+} + **Bl-OAc** → Ru(bpy)_3^{3+} + **Bl-3** + AcO $\Delta G_{\text{HFIP}} = -16.4$ kcal/mol (7)

8.3 Cartesian coordinates and energies of optimized structures

BI-OAc

M06-2X/6-31+G(d) SCF energy: -659.12988545 a.u.

M06-2X/6-31+G(d) enthalpy: -658.968934 a.u.

M06-2X/6-31+G(d) free energy: -659.025297 a.u.

M06-2X/6-311++G(d,p) SCF energy in solution: -659.33123735 a.u.

M06-2X/6-311++G(d,p) enthalpy: -659.170286 a.u.

M06-2X/6-311++G(d,p) free energy: -659.226649 a.u.

Cartesian coordinates

ATOM	X	Y	Z
C	-2.186805	0.120762	-0.000647
C	-0.927377	0.691416	0.000044
C	-0.698798	2.057019	0.001759
C	-1.826774	2.881309	0.002990
C	-3.114640	2.340859	0.002362
C	-3.298844	0.961995	0.000464
C	-2.323585	-1.378609	-0.002036
H	0.305464	2.462057	0.002153
H	-1.690132	3.958426	0.004523

H	-3.975843	3.001240	0.003419
H	-4.285923	0.508927	-0.000044
O	-1.157565	-2.004279	-0.001678
O	-3.396361	-1.933395	-0.003267
I	0.590771	-0.805851	-0.000220
O	1.938123	0.857653	-0.001870
C	3.192014	0.434703	0.004572
O	3.474299	-0.751826	0.013357
C	4.206736	1.549693	-0.009164
H	4.115226	2.109978	-0.943825
H	4.012274	2.239723	0.815499
H	5.208559	1.129629	0.075526

BI-1

M06-2X/6-31+G(d) SCF energy: -659.22774316 a.u.

M06-2X/6-31+G(d) enthalpy: -659.068338 a.u.

M06-2X/6-31+G(d) free energy: -659.128858 a.u.

M06-2X/6-311++G(d,p) SCF energy in solution: -659.48981655 a.u.

M06-2X/6-311++G(d,p) enthalpy: -659.330411 a.u.

M06-2X/6-311++G(d,p) free energy: -659.390931 a.u.

Cartesian coordinates

ATOM	X	Y	Z
C	2.292165	0.239393	-0.000132
C	0.955654	0.617448	-0.000059
C	0.550822	1.950195	-0.000076
C	1.534851	2.937929	-0.000167
C	2.887448	2.590254	-0.000242
C	3.257938	1.249699	-0.000224
C	2.734746	-1.226952	-0.000112
H	-0.506314	2.196755	-0.000018
H	1.237647	3.983572	-0.000179
H	3.649032	3.366114	-0.000314

H	4.298181	0.936896	-0.000278
O	1.785153	-2.074538	0.000043
O	3.948227	-1.458906	-0.000238
I	-0.534303	-0.905164	0.000092
O	-2.374768	1.069516	0.000119
C	-3.449737	0.382201	0.000112
O	-3.535200	-0.856580	0.000089
C	-4.736464	1.218898	0.000130
H	-4.749433	1.865555	0.882798
H	-4.749428	1.865606	-0.882501
H	-5.613442	0.568872	0.000109

BI-2

M06-2X/6-31+G(d) SCF energy: -430.74156239 a.u.

M06-2X/6-31+G(d) enthalpy: -430.639153 a.u.

M06-2X/6-31+G(d) free energy: -430.684410 a.u.

M06-2X/6-311++G(d,p) SCF energy in solution: -659.48981655 a.u.

M06-2X/6-311++G(d,p) enthalpy: -659.387407 a.u.

M06-2X/6-311++G(d,p) free energy: -659.432664 a.u.

Cartesian coordinates

ATOM	X	Y	Z
C	1.261548	0.516621	-0.000005
C	0.459383	-0.617658	-0.000047
C	0.995309	-1.899331	0.000029
C	2.382684	-2.039015	0.000175
C	3.208614	-0.914240	0.000272
C	2.648845	0.358264	0.000190
C	0.700006	1.909792	-0.000153
H	0.352160	-2.773122	0.000015
H	2.814777	-3.035214	0.000251
H	4.287237	-1.033062	0.000405
H	3.260865	1.255478	0.000238

O	-0.606247	2.036954	-0.000780
O	1.410723	2.897249	0.000386
I	-1.643192	-0.335364	-0.000010

BI-3

M06-2X/6-31+G(d) SCF energy: -430.87638502 a.u.

M06-2X/6-31+G(d) enthalpy: -430.774583 a.u.

M06-2X/6-31+G(d) free energy: -430.820810 a.u.

M06-2X/6-311++G(d,p) SCF energy in solution: -431.07893180 a.u.

M06-2X/6-311++G(d,p) enthalpy: -430.977130 a.u.

M06-2X/6-311++G(d,p) free energy: -431.023357 a.u.

Cartesian coordinates

ATOM	X	Y	Z
C	1.343638	0.381596	-0.000009
C	0.337462	-0.583752	0.000027
C	0.625294	-1.953013	0.000056
C	1.946294	-2.388786	0.000026
C	2.975961	-1.448051	-0.000041
C	2.665427	-0.093633	-0.000051
C	1.162498	1.943047	0.000013
H	-0.185769	-2.674862	0.000114
H	2.161700	-3.454732	0.000047
H	4.014485	-1.771223	-0.000093
H	3.437745	0.669957	-0.000084
O	-0.012577	2.350335	0.000006
O	2.237323	2.579354	0.000039
I	-1.765388	-0.138700	-0.000009

AcO-

M06-2X/6-31+G(d) SCF energy: -228.43236595 a.u.

M06-2X/6-31+G(d) enthalpy: -228.378045 a.u.

M06-2X/6-31+G(d) free energy: -228.411558 a.u.

M06-2X/6-311++G(d,p) SCF energy in solution: -228.60628795 a.u.

M06-2X/6-311++G(d,p) enthalpy: -228.551967 a.u.

M06-2X/6-311++G(d,p) free energy: -228.585480 a.u.

Cartesian coordinates

ATOM	X	Y	Z
C	-1.347532	-0.051596	-0.000001
H	-1.729639	0.476278	0.882045
H	-1.729649	0.476376	-0.881983
C	0.209652	0.001190	-0.000002
O	0.802793	-1.103892	0.000000
O	0.697592	1.157847	0.000000
H	-1.716512	-1.081858	-0.000052

AcO radical

M06-2X/6-31+G(d) SCF energy: -228.30816108 a.u.

M06-2X/6-31+G(d) enthalpy: -228.254162 a.u.

M06-2X/6-31+G(d) free energy: -228.288393 a.u.

M06-2X/6-311++G(d,p) SCF energy in solution: -228.38359529 a.u.

M06-2X/6-311++G(d,p) enthalpy: -228.329596 a.u.

M06-2X/6-311++G(d,p) free energy: -228.363827 a.u.

Cartesian coordinates

ATOM	X	Y	Z
C	-1.353003	-0.104275	-0.001640
H	-1.685989	-0.335956	1.013724
H	-1.790422	0.846417	-0.314459
C	0.154364	-0.062290	-0.006706
O	0.910535	-1.003143	0.001711
O	0.631980	1.177475	0.000993
H	-1.671876	-0.905726	-0.670817

BzO radical

M06-2X/6-31+G(d) SCF energy: -419.97383060 a.u.

M06-2X/6-31+G(d) enthalpy: -419.862909 a.u.

M06-2X/6-31+G(d) free energy: -419.904056 a.u.

M06-2X/6-311++G(d,p) SCF energy in solution: -420.09336129 a.u.

M06-2X/6-311++G(d,p) enthalpy: -419.982440 a.u.

M06-2X/6-311++G(d,p) free energy: -420.023587 a.u.

Cartesian coordinates

ATOM	X	Y	Z
C	-1.833234	-1.210020	0.000249
C	-0.442239	-1.222506	0.000257
C	0.252718	-0.011936	-0.000018
C	-0.428048	1.207680	-0.000306
C	-1.819831	1.212859	-0.000315
C	-2.519219	0.005659	-0.000034
H	-2.383836	-2.145467	0.000467
H	0.117705	-2.152981	0.000471
H	-2.358461	2.155184	-0.000552
H	-3.605222	0.012262	-0.000034
C	1.734670	-0.058667	0.000032
O	2.435591	-1.050045	-0.000005
O	2.368883	1.106193	0.000130
H	0.125123	2.143400	-0.000542

Ph radical

M06-2X/6-31+G(d) SCF energy: -231.45729696 a.u.

M06-2X/6-31+G(d) enthalpy: -231.363600 a.u.

M06-2X/6-31+G(d) free energy: -231.396927 a.u.

M06-2X/6-311++G(d,p) SCF energy in solution: -231.52000248 a.u.

M06-2X/6-311++G(d,p) enthalpy: -231.426306 a.u.

M06-2X/6-311++G(d,p) free energy: -231.459633 a.u.

Cartesian coordinates

ATOM	X	Y	Z
C	-1.223860	-0.769470	0.000127
C	-0.000018	-1.400238	0.000085
C	1.224016	-0.769268	-0.000243
C	1.212335	0.631345	0.000121
C	-0.000156	1.322902	-0.000004
C	-1.212302	0.631388	-0.000169
H	-2.158310	-1.322692	0.000058
H	-0.000053	2.408699	0.000167
H	-2.151432	1.178126	-0.000029
H	2.151258	1.178331	0.000202
H	2.158445	-1.322422	0.000106

28

M06-2X/6-31+G(d) SCF energy: -242.22393023 a.u.

M06-2X/6-31+G(d) enthalpy: -242.139205 a.u.

M06-2X/6-31+G(d) free energy: -242.178367 a.u.

M06-2X/6-311++G(d,p) SCF energy in solution: -231.52000248 a.u.

M06-2X/6-311++G(d,p) enthalpy: 0.000000 a.u.

M06-2X/6-311++G(d,p) free energy: 0.000000 a.u.

Cartesian coordinates

ATOM	X	Y	Z
C	-2.645211	-1.269303	0.000000
C	-1.271348	-1.172315	0.000000
C	-0.569594	0.000607	0.000001
C	-1.284613	1.201078	0.000001
C	-2.679794	1.154193	0.000000
C	-3.359334	-0.065173	0.000000
H	-3.153922	-2.228532	-0.000001
H	-3.240120	2.084082	-0.000001
H	-4.445334	-0.082819	-0.000001
H	-0.761876	2.153180	-0.000001

I	1.555861	-0.019253	0.000000

CO_2

M06-2X/6-31+G(d) SCF energy: -188.51469500 a.u.

M06-2X/6-31+G(d) enthalpy: -188.499178 a.u.

M06-2X/6-31+G(d) free energy: -188.523423 a.u.

M06-2X/6-311++G(d,p) SCF energy in solution: -188.57200890 a.u.

M06-2X/6-311++G(d,p) enthalpy: -188.556492 a.u.

M06-2X/6-311++G(d,p) free energy: -188.580737 a.u.

Cartesian coordinates

ATOM	X	Y	Z
C	0.000000	0.000000	0.000000
O	0.000000	0.000000	1.163071
O	0.000000	0.000000	-1.163071

$BuB(OH)_2$

M06-2X/6-31+G(d) SCF energy: -334.31712425 a.u.

M06-2X/6-31+G(d) enthalpy: -334.148920 a.u.

M06-2X/6-31+G(d) free energy: -334.192915 a.u.

M06-2X/6-311++G(d,p) SCF energy in solution: -334.44056789 a.u.

M06-2X/6-311++G(d,p) enthalpy: -334.272364 a.u.

M06-2X/6-311++G(d,p) free energy: -334.316359 a.u.

Cartesian coordinates

ATOM	X	Y	Z
C	-2.173103	-0.572452	-0.000387
H	-2.211836	-1.230077	-0.879756
H	-2.211822	-1.231304	0.878062
C	-0.848326	0.188145	0.000155
H	-0.806721	0.850683	-0.874427
H	-0.807007	0.849916	0.875341
C	0.376504	-0.731075	-0.000109

H	0.333232	-1.401439	0.873193
H	0.333140	-1.400880	-0.873839
B	1.765279	0.022714	-0.000009
O	1.797506	1.386853	-0.000218
H	2.704750	1.725556	-0.000146
O	2.977231	-0.629947	0.000254
H	2.902412	-1.592263	0.000349
C	-3.384664	0.357659	0.000277
H	-3.378880	1.005767	-0.883286
H	-4.324976	-0.202720	-0.000292
H	-3.379052	1.004273	0.884935

TS-1

M06-2X/6-31+G(d) SCF energy: -765.05578593 a.u.

M06-2X/6-31+G(d) enthalpy: -764.785473 a.u.

M06-2X/6-31+G(d) free energy: -764.852915 a.u.

M06-2X/6-311++G(d,p) SCF energy in solution: -765.30591138 a.u.

M06-2X/6-311++G(d,p) enthalpy: -765.035598 a.u.

M06-2X/6-311++G(d,p) free energy: -765.103040 a.u.

Imaginary frequency: -270.7239 cm-1

Cartesian coordinates

ATOM	X	Y	Z
C	-4.059280	1.895144	-0.258200
C	-3.546927	0.608836	-0.424762
C	-2.189456	0.402334	-0.212224
C	-1.335022	1.436375	0.155893
C	-1.867827	2.716999	0.323198
C	-3.222943	2.946961	0.116041
H	-5.118339	2.069864	-0.421713
H	-4.198990	-0.210060	-0.711152
H	-1.190642	3.512487	0.619633
H	-3.630225	3.944273	0.246340

C	0.134423	1.235192	0.397933
O	0.846437	2.112622	0.824396
O	0.570769	0.022003	0.061058
B	1.590015	-0.938544	1.026843
C	2.993866	-0.110049	0.990802
H	2.871416	0.768727	1.633234
H	3.714395	-0.779943	1.485830
C	3.514943	0.309803	-0.379064
H	3.553339	-0.552039	-1.066863
H	2.817383	1.028664	-0.828764
C	4.908894	0.937814	-0.321559
H	5.613382	0.217085	0.114839
H	4.879965	1.795627	0.362811
O	0.954855	-0.990809	2.282713
H	0.608428	-1.876599	2.455218
O	1.539234	-2.183283	0.325745
H	2.140256	-2.205784	-0.430562
I	-1.365305	-1.543545	-0.425871
C	5.411114	1.386998	-1.692519
H	6.408965	1.833414	-1.630809
H	5.464686	0.540189	-2.386918
H	4.736025	2.132024	-2.128674

29

M06-2X/6-31+G(d) SCF energy: -607.41390059 a.u.

M06-2X/6-31+G(d) enthalpy: -607.270208 a.u.

M06-2X/6-31+G(d) free energy: -607.325398 a.u.

M06-2X/6-311++G(d,p) SCF energy in solution: -607.60702977 a.u.

M06-2X/6-311++G(d,p) enthalpy: -607.463337 a.u.

M06-2X/6-311++G(d,p) free energy: -607.518527 a.u.

Cartesian coordinates

ATOM X Y Z

C	-3.304999	1.495311	-0.447941
C	-2.675995	0.255999	-0.362228
C	-1.310459	0.176803	-0.087168
C	-0.563757	1.345231	0.109006
C	-1.222778	2.580751	0.047517
C	-2.578912	2.664415	-0.235775
H	-4.366720	1.537959	-0.671804
H	-3.251524	-0.652643	-0.504594
H	-0.632648	3.474230	0.226215
H	-3.064602	3.633308	-0.290619
C	0.908725	1.440816	0.380080
O	1.402878	2.346200	0.996757
I	-0.550985	-1.808729	0.129771
O	1.608222	0.424082	-0.159474
B	2.997073	0.350287	-0.297630
O	3.764717	1.461439	-0.342097
H	4.697815	1.249525	-0.482148
O	3.531690	-0.893507	-0.454789
H	2.863536	-1.592814	-0.410895

n-butyl radical

M06-2X/6-31+G(d) SCF energy: -157.68966288 a.u.

M06-2X/6-31+G(d) enthalpy: -157.564109 a.u.

M06-2X/6-31+G(d) free energy: -157.599579 a.u.

M06-2X/6-311++G(d,p) SCF energy in solution: -157.73738981 a.u.

M06-2X/6-311++G(d,p) enthalpy: -157.611836 a.u.

M06-2X/6-311++G(d,p) free energy: -157.647306 a.u.

Cartesian coordinates

ATOM	X	Y	Z
C	-0.503443	0.523498	-0.070447
H	-0.392725	1.268630	0.728414

H	-0.407185	1.067497	-1.018313
C	0.636519	-0.493999	0.037510
H	0.510920	-1.056713	0.980751
H	0.537711	-1.241700	-0.761714
C	1.987876	0.132763	-0.014419
H	2.859889	-0.435585	-0.315515
H	2.151057	1.110746	0.426803
C	-1.880258	-0.129869	0.019243
H	-2.682016	0.610265	-0.064437
H	-2.017661	-0.864917	-0.781809
H	-2.004150	-0.652581	0.974496

9. References

[1] M. Victoria, J. Waser, *Org. Lett.* **2013**, *15*, 3246.

[2] P. Eisenberger, S. Gischig, A. Togni, *Chem. Eur. J.* **2006**, *12*, 2579.

[3] V. Matousek, E. Pietrasiak, R. Schwenk, A. Togni, *J. Org. Chem.* **2013**, *78*, 6763.

[4] Z.-D. Shi, B.-H. Yang, Y.-L. Wu, *Tetrahedron* **2002**, *58*, 3287.

[5] S. Ogi, K. Sugiyasu, M. Takeuchi, *Bull. Chem. Soc. Jpn*. **2011**, *84*, 40.

[6] J. Xu, Y. Fu, D.-F. Luo, Y.-Y. Jiang, B. Xiao, Z.-J. Liu, T.-J. Gong, L. Liu, *J. Am. Chem*. Soc. **2011**, *133*, 15300.

[7] L. R. Odell, J. Lindh, T. Gustafsson, M. Larhed, *Eur. J. Org. Chem*. **2010**, 2270.

[8] H. Huang, G. Zhang, L, Gong, S. Zhang, Y. Chen. *J. Am. Chem. Soc.* **2014**, *136*, 2280.

[9] J. Xu, B. Xiao, C.-Q. Xie, D.-F. Luo, L. Liu, Y. Fu, *Angew. Chem. Int. Ed*. **2012**, *51*, 12551.

[10] R. Nandhakumar, T. Suresh, A. L. C. Jude, V. R. Kannan, P. S. Mohan, *Eur. J. Med. Chem.* **2007**, *42*, 1128.

[11] G. E. Tumambac, C. Wolf, *J. Org. Chem*. **2004**, *69*, 2048.

[12] G. A. Molander, V. Colombel, V. A. Braz, *Org. Lett*. **2011**, *13*, 1852.

[13] J-Y. Tsai, C.-S. Chang, Y.-F. Huang, H.-S. Chen, S.-K Lin, F. F. Wong, L.-J. Huang, S.-C. Kuo, *Tetrahedron* **2008**, *64*, 11751.

[14] A. P. Antonchick, L. Burgmann, *Angew. Chem. Int. Ed*. **2013**, *52*, 3267.

[15] A. Sugimori, T. Yamada, H. Ishida, M. Nose, K. Terashima, N. Oohata, *Bull. Chem. Soc. Jpn*. **1986**, *59*, 3905.

[16] R. Xia, M.-S. Xie, H.-Y. Niu, G.-R. Qu, H-M. Guo, *Org. Lett*. **2014**, *16*, 444.

[17] X. Fan, Y. Wang, Y. He, X. Zhang, J. Wang, *Tetrahedron Lett.* **2010**, *51*, 3493.

[18] H.-J. Lim, D. Myung, I. Y. C. Lee, M. H. Jung, *J. Comb. Chem*. **2008**, *10*, 501.

[19] A. Shaabani, A. Maleki, *Chem. Pharm. Bull*. **2008**, *56*, 79.

[20] J. G. Samaritoni, *Org. Prep. Proced. Int*. **1988**, *20*, 117.

[21] Y. Zhang, M. R. Sheets, E. K. Raja, K. N. Boblak, D. A. Klumpp, *J. Am. Chem. Soc*. **2011**, *133*, 8467.

[22] X. Wang, X. Wu, N. Cheng, H. Zhao, Z. Gu, S. Xiang, *Synthetic Commun*. **2007**, *37*, 519.

[23] M. Li, W. Jin, C. Jiang, C. Zheng, W. Tang, T. You, L. Lou, *Bioorg. Med. Chem. Lett*. **2009**,

19, 4107.

[24] J. E. Leffler, R. D. Faulkner, C. C. Petropoulos, *J. Am. Chem. Soc.* **1958**, *80*, 5435.

[25] Gaussian 09, Revision D.01, M. J. Frisch, G. W. Trucks, H. B. Schlegel, G. E. Scuseria, M. A. Robb, J. R. Cheeseman, G. Scalmani, V. Barone, B. Mennucci, G. A. Petersson, H. Nakatsuji, M. Caricato, X. Li, H. P. Hratchian, A. F. Izmaylov, J. Bloino, G. Zheng, J. L. Sonnenberg, M. Hada, M. Ehara, K. Toyota, R. Fukuda, J. Hasegawa, M. Ishida, T. Nakajima, Y. Honda, O. Kitao, H. Nakai, T. Vreven, J. A. Montgomery, Jr., J. E. Peralta, F. Ogliaro, M. Bearpark, J. J. Heyd, E. Brothers, K. N. Kudin, V. N. Staroverov, R. Kobayashi, J. Normand, K. Raghavachari, A. Rendell, J. C. Burant, S. S. Iyengar, J. Tomasi, M. Cossi, N. Rega, J. M. Millam, M. Klene, J. E. Knox, J. B. Cross, V. Bakken, C. Adamo, J. Jaramillo, R. Gomperts, R. E. Stratmann, O. Yazyev, A. J. Austin, R. Cammi, C. Pomelli, J. W. Ochterski, R. L. Martin, K. Morokuma, V. G. Zakrzewski, G. A. Voth, P. Salvador, J. J. Dannenberg, S. Dapprich, A. D. Daniels, Ö. Farkas, J. B. Foresman, J. V. Ortiz, J. Cioslowski, and D. J. Fox, Gaussian, Inc., Wallingford CT, 2009. [2] Hand book of Chemistry and Physics. 90th edition, David R. Lide, CRC Press, **2009-2010**.

[26] C. R. Bock, T. J. Meyer, D. G. Whitten, *J. Am. Chem. Soc.*,**1975**, *97*, 2909.

[27] A. A. Isse, A. Gennaro, *J. Phys. Chem. B* **2010**, *114*, 7894.

[28] (a) J. E. Bartmess *J. Phys. Chem.* **1994**, *98*, 6420−6424. (b) J. E. Bartmess *J. Phys. Chem.* **1995**, *99*, 6755.

10. ^{1}H-NMR and ^{13}C-NMR spectra

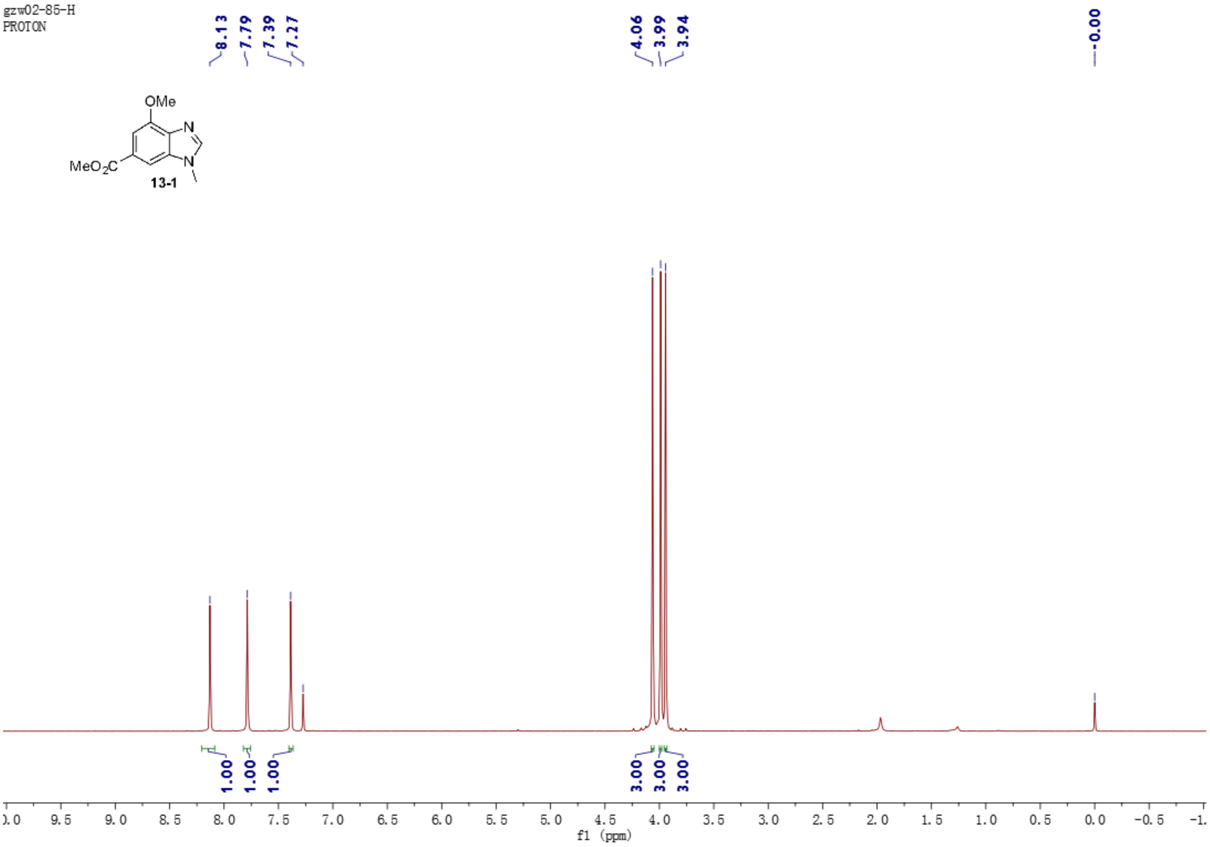

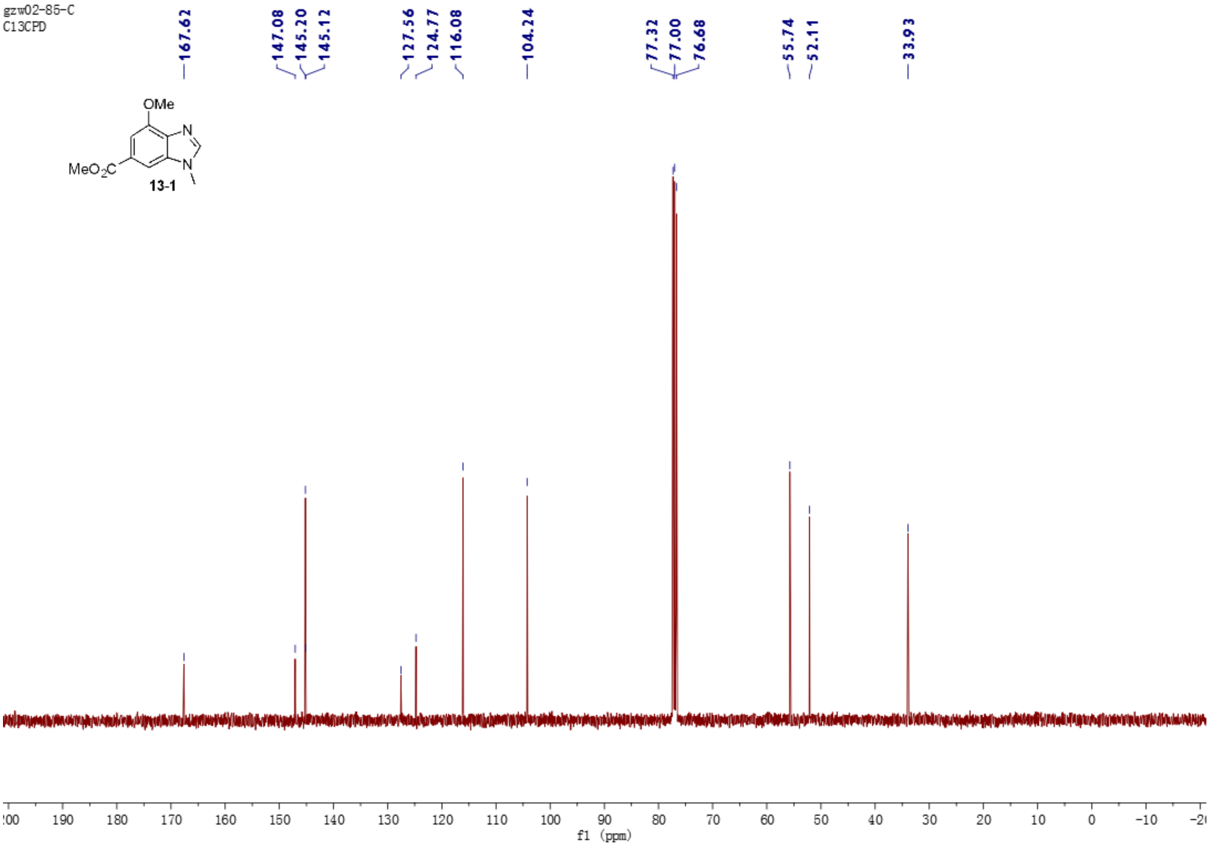

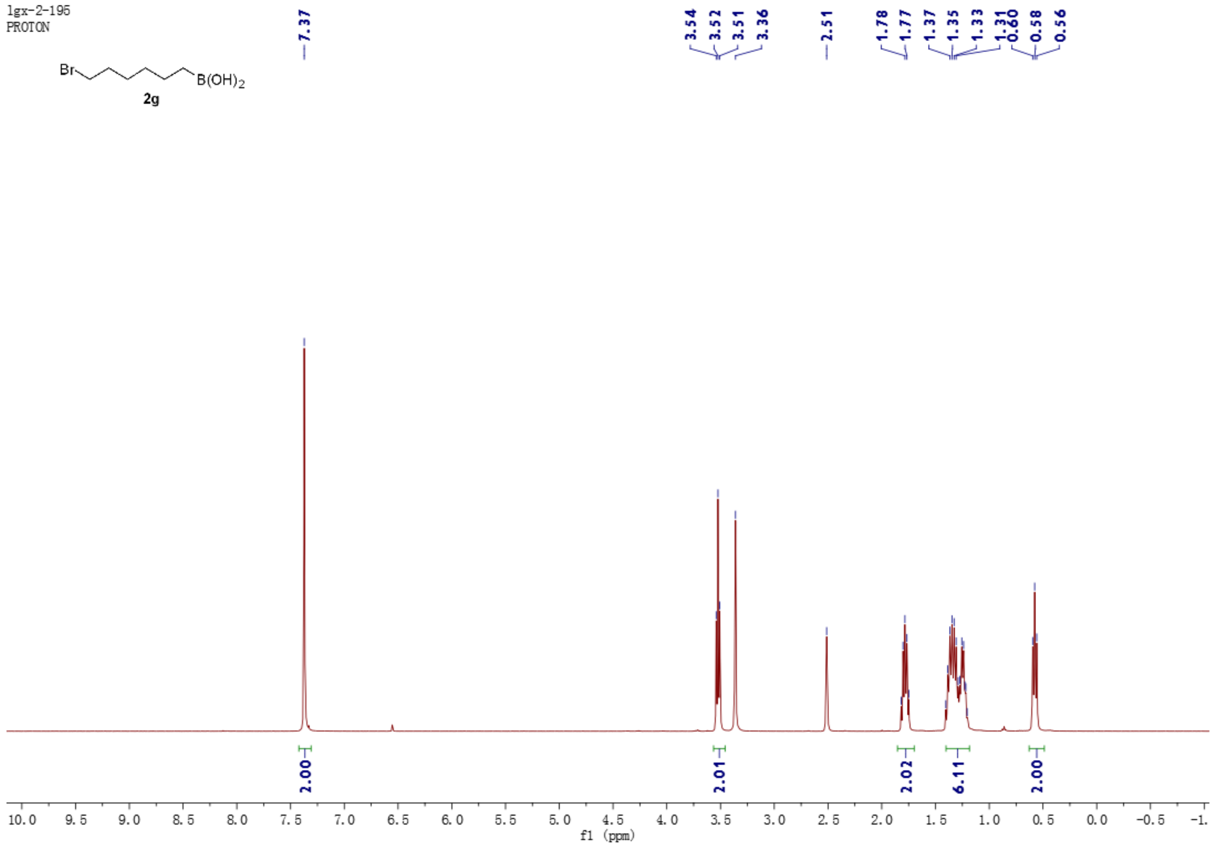
lgx-2-195
PROTON
Br
B(OH)2
2g
7.37
3.54
3.52
3.51
3.36
2.51
1.78
1.77
1.37
1.35
1.33
1.31
0.60
0.58
0.56
2.00
2.01
2.02
6.11
2.00
f1 (ppm)

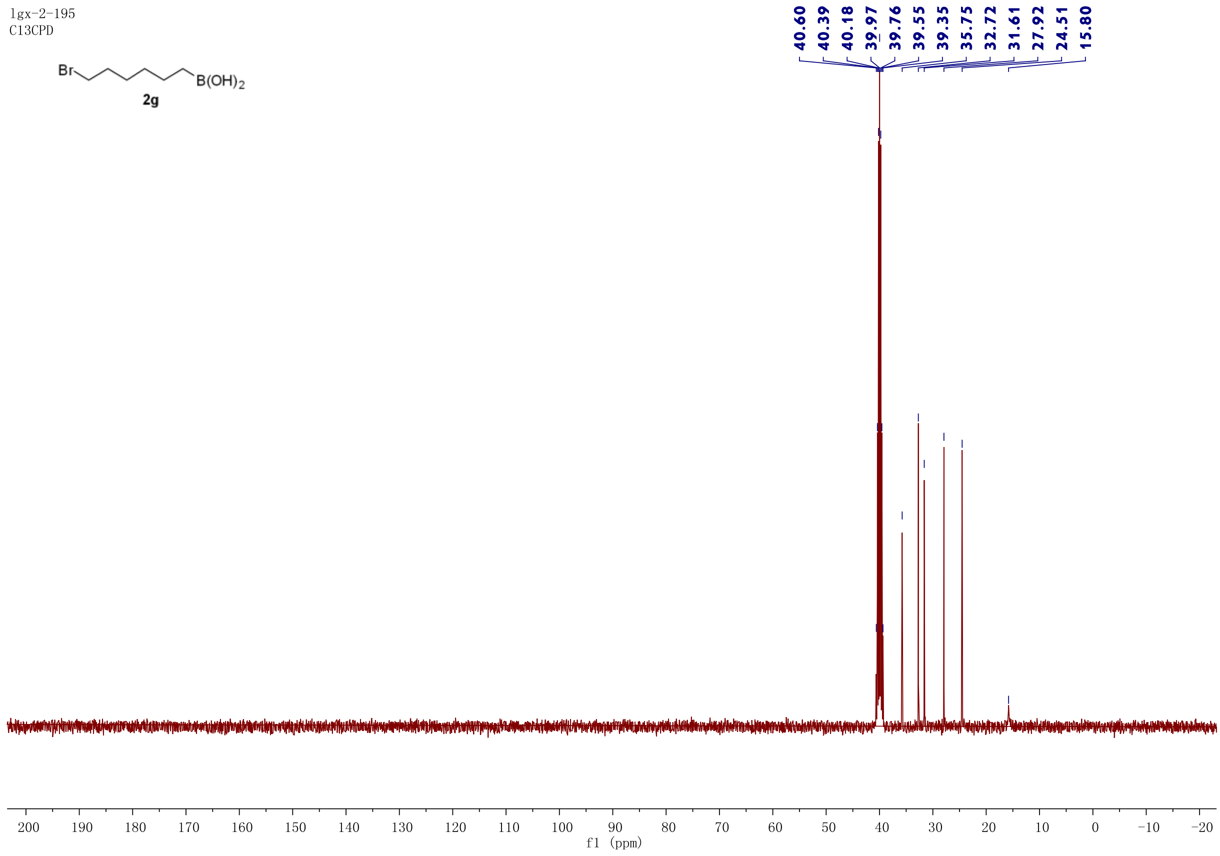
lgx-2-195
C13CPD
Br
B(OH)2
2g
40.60
40.39
40.18
39.97
39.76
39.55
39.35
35.75
32.72
31.61
27.92
24.51
15.80
f1 (ppm)

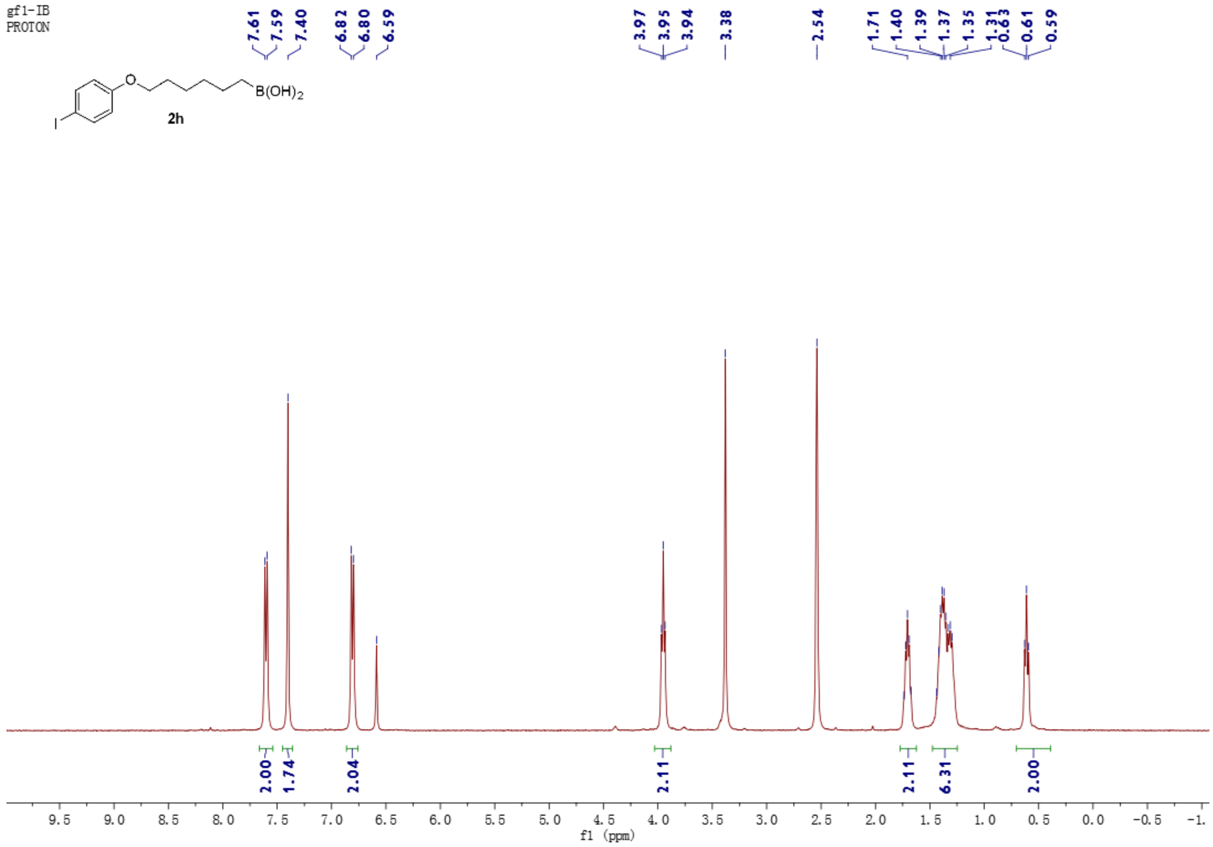
gf1-IB
PROTON
B(OH)2
2h
7.61
7.59
7.40
6.82
6.80
6.59
3.97
3.95
3.94
3.38
2.54
1.71
1.40
1.39
1.37
1.35
1.31
0.63
0.61
0.59
2.00
1.74
2.04
2.11
2.11
6.31
2.00
f1 (ppm)

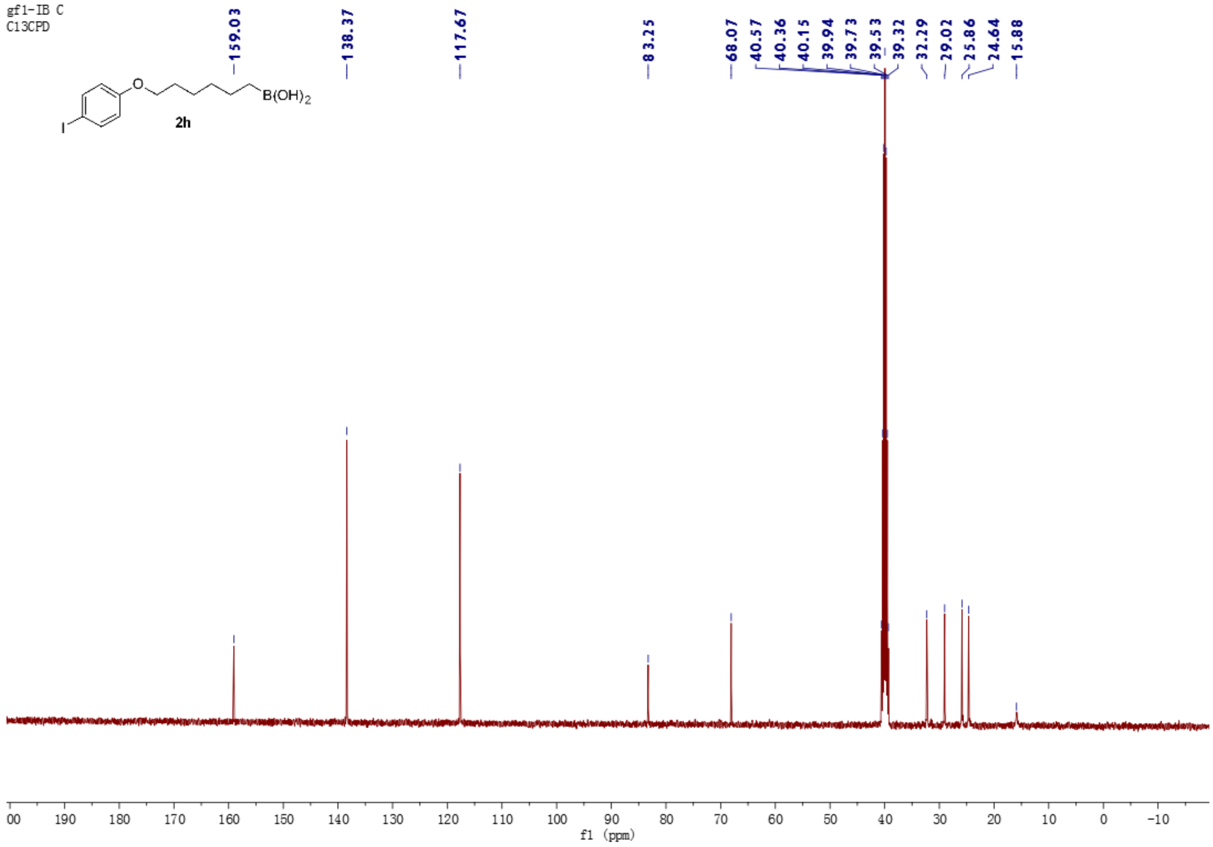
gf1-IB C
C13CPD
B(OH)2
2h
159.03
138.37
117.67
83.25
68.07
40.57
40.36
40.15
39.94
39.73
39.53
39.32
32.29
29.02
25.86
24.64
15.88
f1 (ppm)

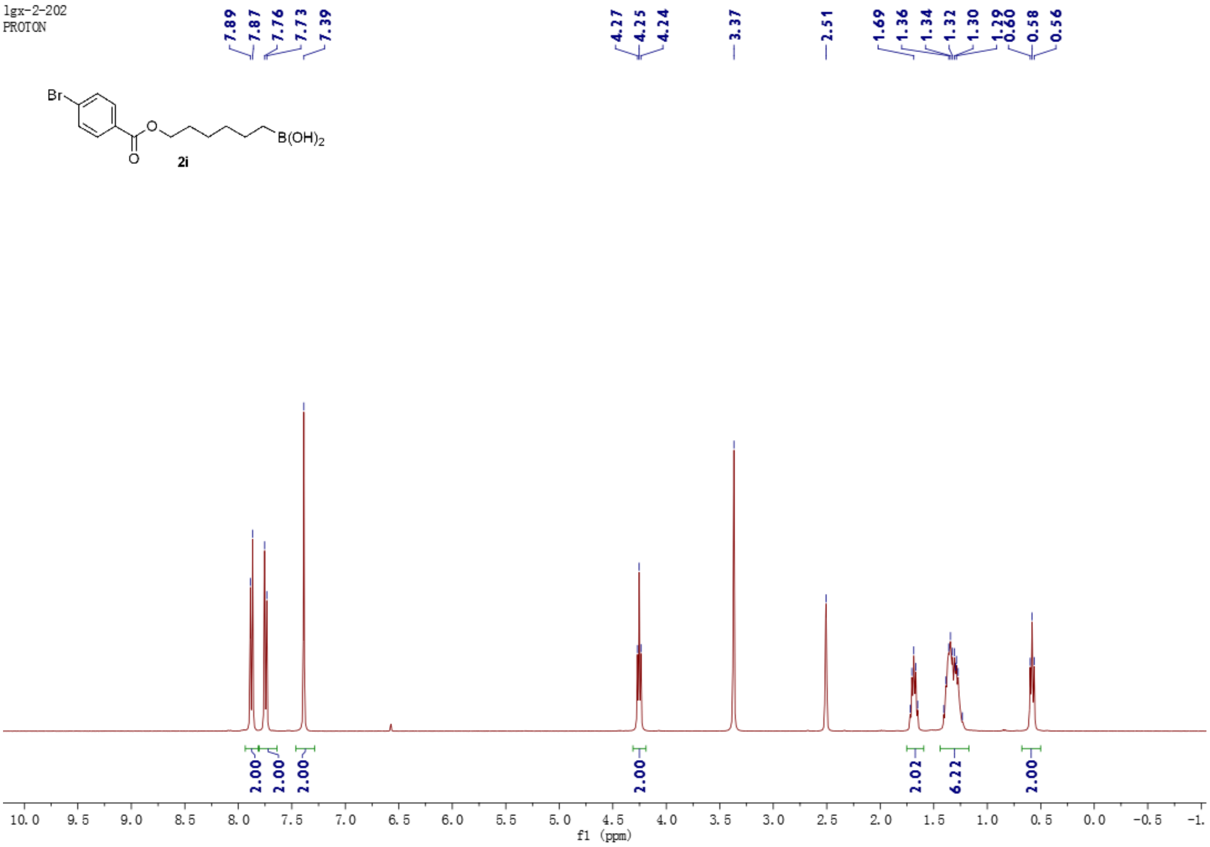
lgx-2-202
PROTON
7.89
7.87
7.76
7.73
7.39
4.27
4.25
4.24
3.37
2.51
1.69
1.36
1.34
1.32
1.30
1.29
0.60
0.58
0.56
Br
O
O
B(OH)2
2i
2.00
2.00
2.00
2.00
2.02
6.22
2.00
f1 (ppm)

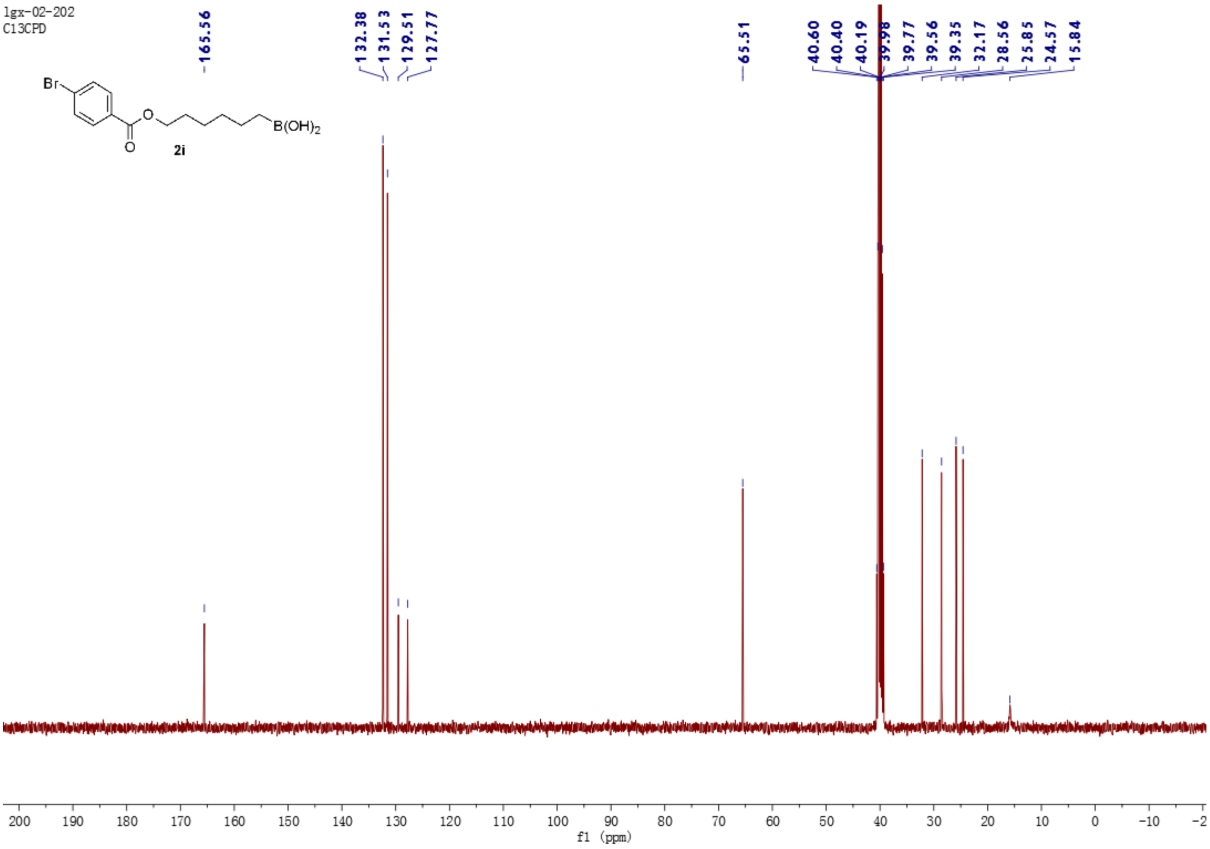
lgx-02-202
C13CPD
165.56
132.38
131.53
129.51
127.77
65.51
40.60
40.40
40.19
39.98
39.77
39.56
39.35
32.17
28.56
25.85
24.57
15.84
Br
O
O
B(OH)2
2i
f1 (ppm)

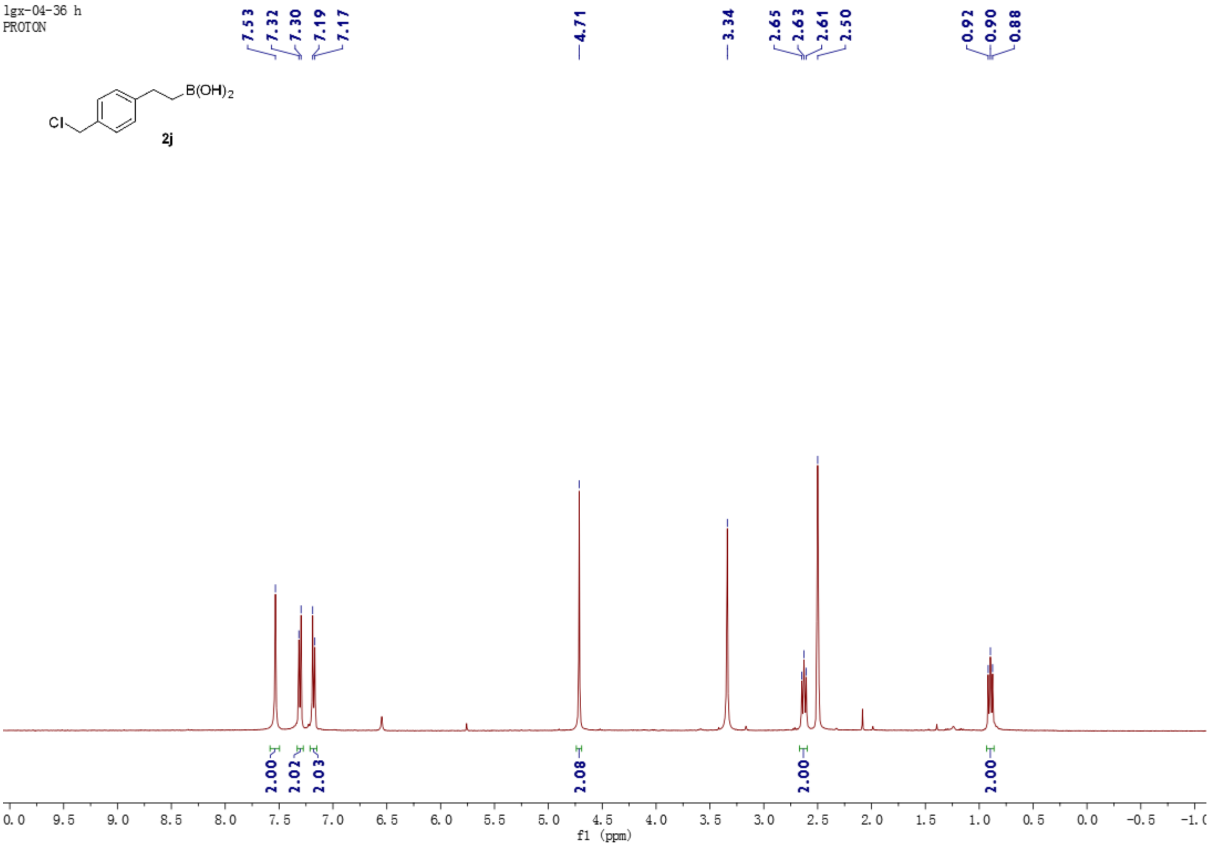
lgx-04-36 h
PROTON
B(OH)2
Cl
2j
7.53 7.32 7.30 7.19 7.17
4.71
3.34
2.65 2.63 2.61 2.50
0.92 0.90 0.88
2.00 2.02 2.03
2.08
2.00
2.00
f1 (ppm)

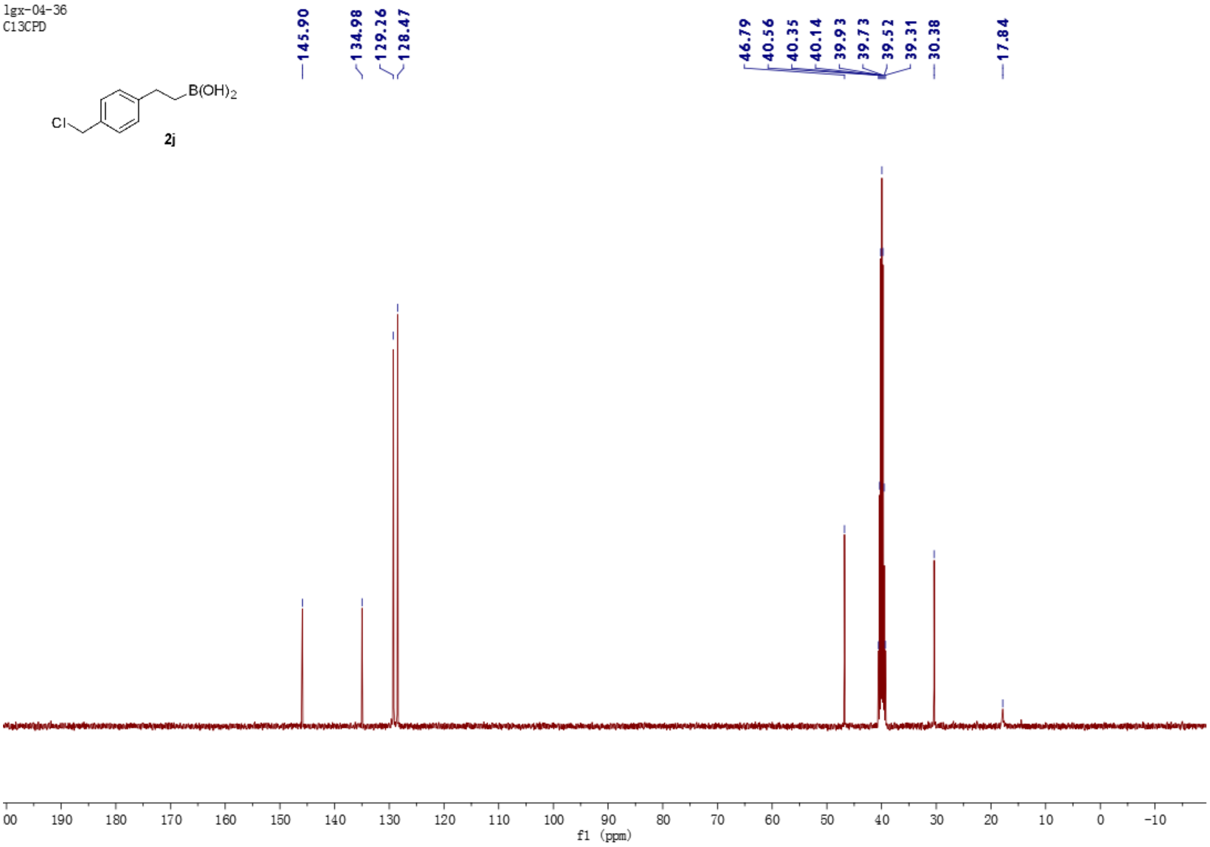
lgx-04-36
C13CPD
B(OH)2
Cl
2j
145.90
134.98 129.26 128.47
46.79 40.56 40.35 40.14 39.93 39.73 39.52 39.31 30.38
17.84
f1 (ppm)

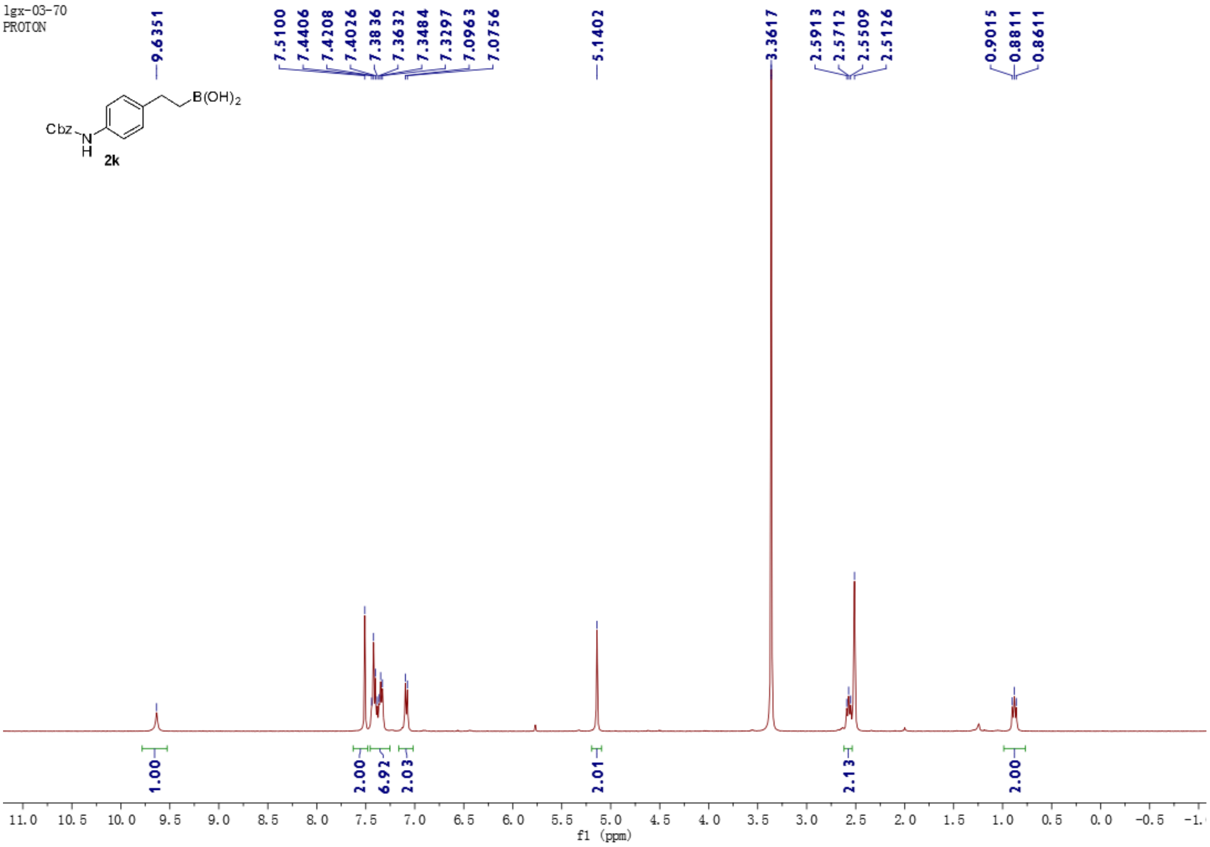
lgx-03-70
PROTON
Cbz
N
H
B(OH)2
2k
9.6351
7.5100
7.4406
7.4208
7.4026
7.3836
7.3632
7.3484
7.3297
7.0963
7.0756
5.1402
3.3617
2.5913
2.5712
2.5509
2.5126
0.9015
0.8811
0.8611
1.00
2.00
6.92
2.03
2.01
2.13
2.00
f1 (ppm)

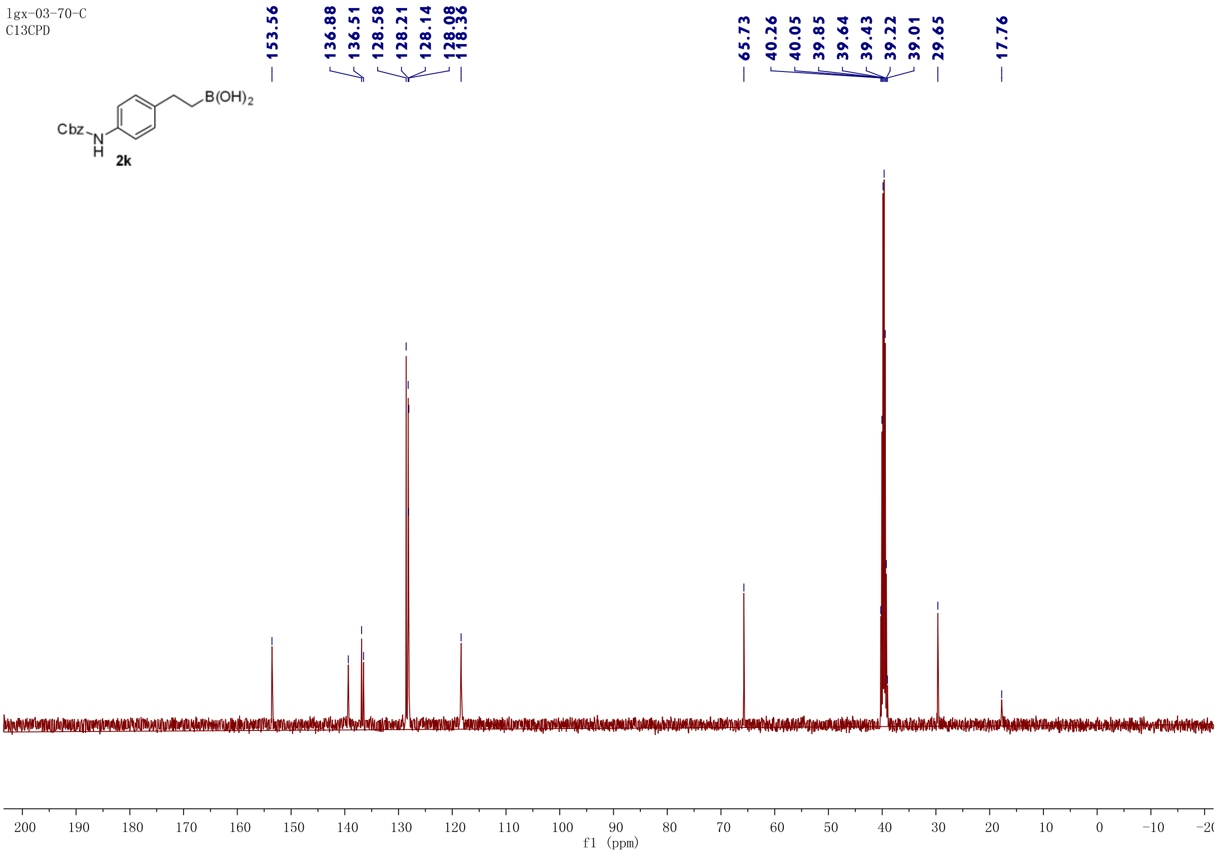
lgx-03-70-C
C13CPD
Cbz
N
H
B(OH)2
2k
153.56
136.88
136.51
128.58
128.21
128.14
128.08
118.36
65.73
40.26
40.05
39.85
39.64
39.43
39.22
39.01
29.65
17.76
f1 (ppm)

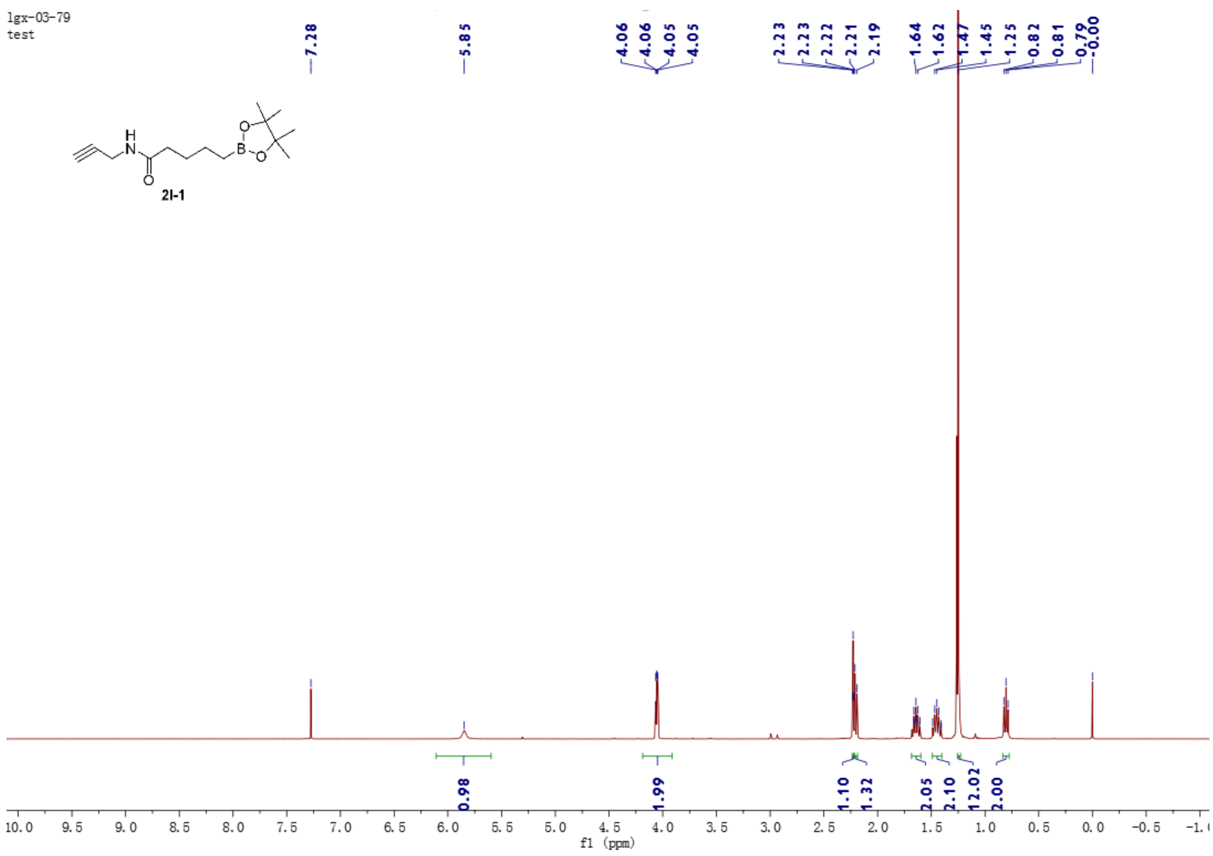
lgx-03-79
test
7.28
5.85
4.06
4.06
4.05
4.05
2.23
2.23
2.22
2.21
2.19
1.64
1.62
1.47
1.45
1.25
0.82
0.81
0.79
-0.00
2l-1
0.98
1.99
1.10
1.32
2.05
2.10
12.02
2.00
f1 (ppm)

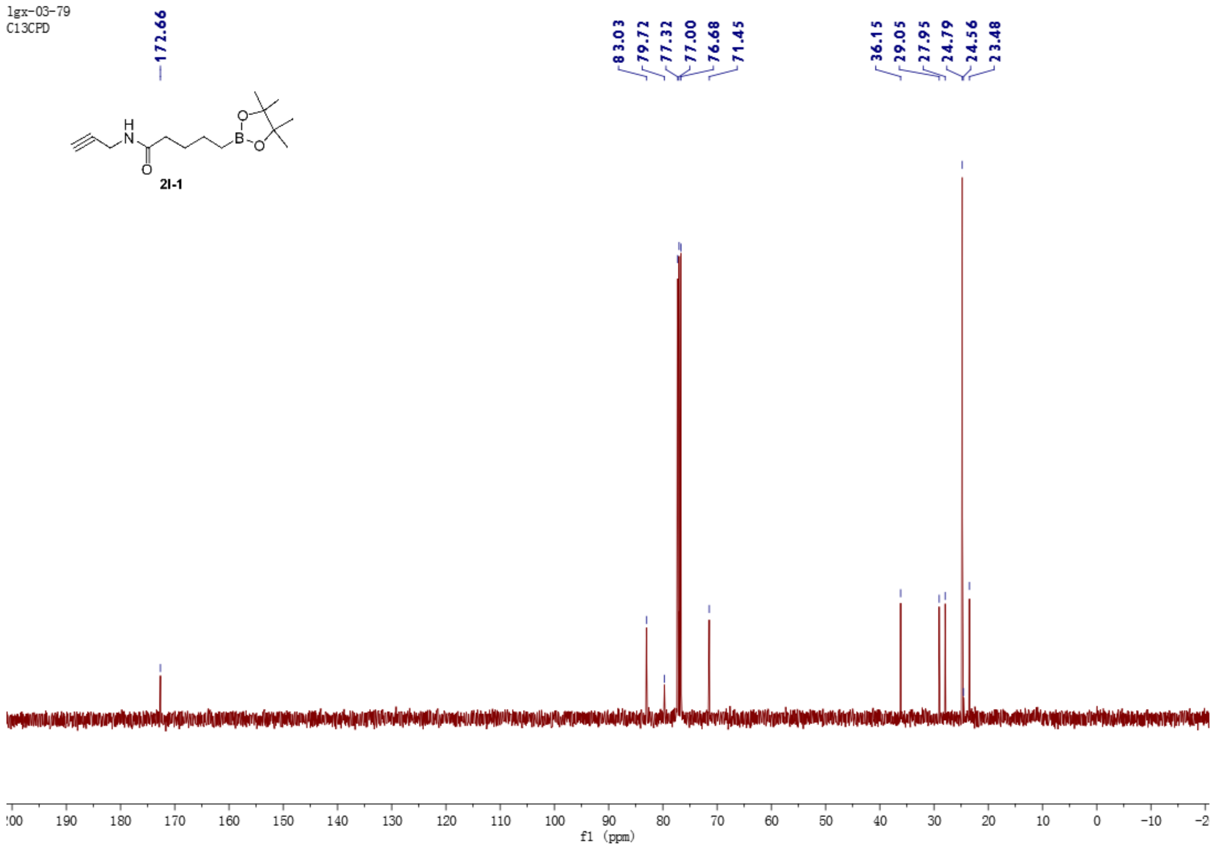
lgx-03-79
C13CPD
172.66
83.03
79.72
77.32
77.00
76.68
71.45
36.15
29.05
27.95
24.79
24.56
23.48
2l-1
f1 (ppm)

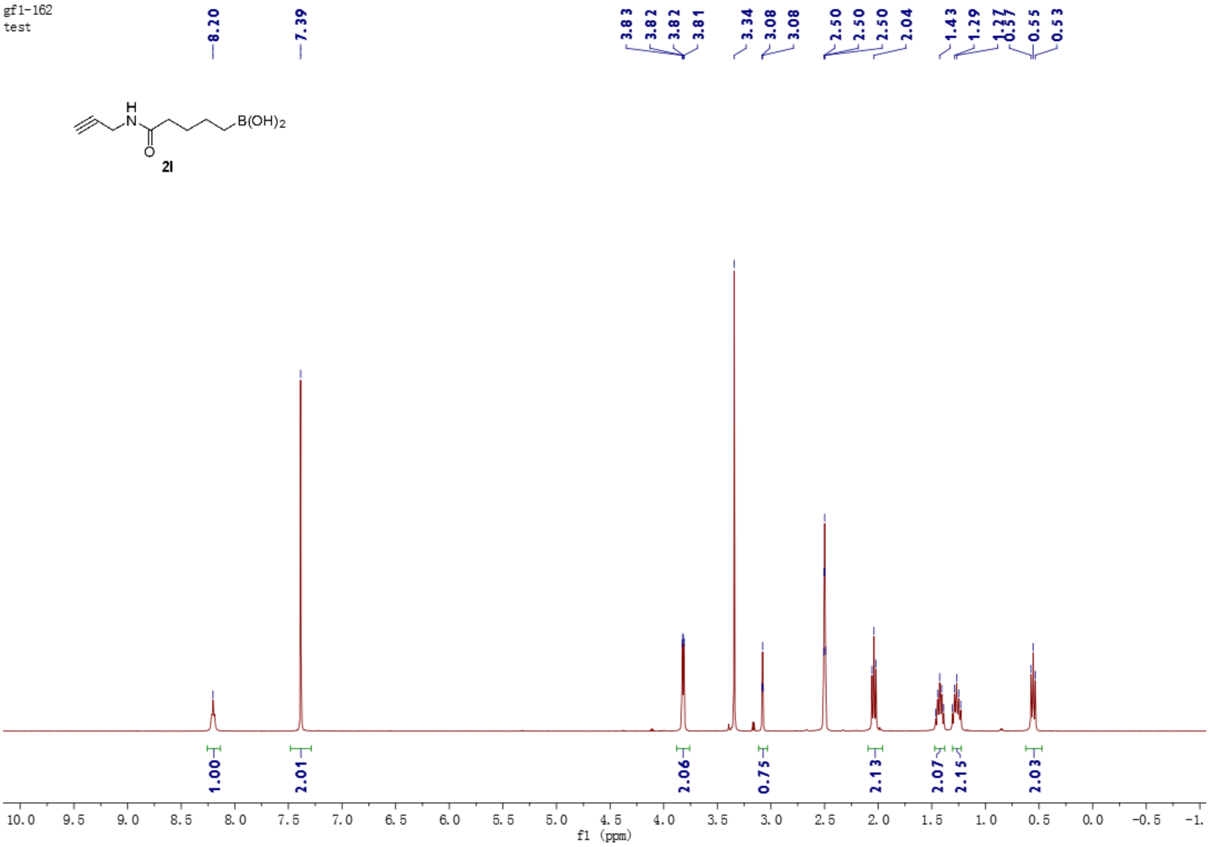
gf1-162
test
8.20
7.39
3.83
3.82
3.82
3.81
3.34
3.08
3.08
2.50
2.50
2.50
2.04
1.43
1.29
1.27
0.57
0.55
0.53
B(OH)2
H
N
O
2l
1.00
2.01
2.06
0.75
2.13
2.07
2.15
2.03
f1 (ppm)

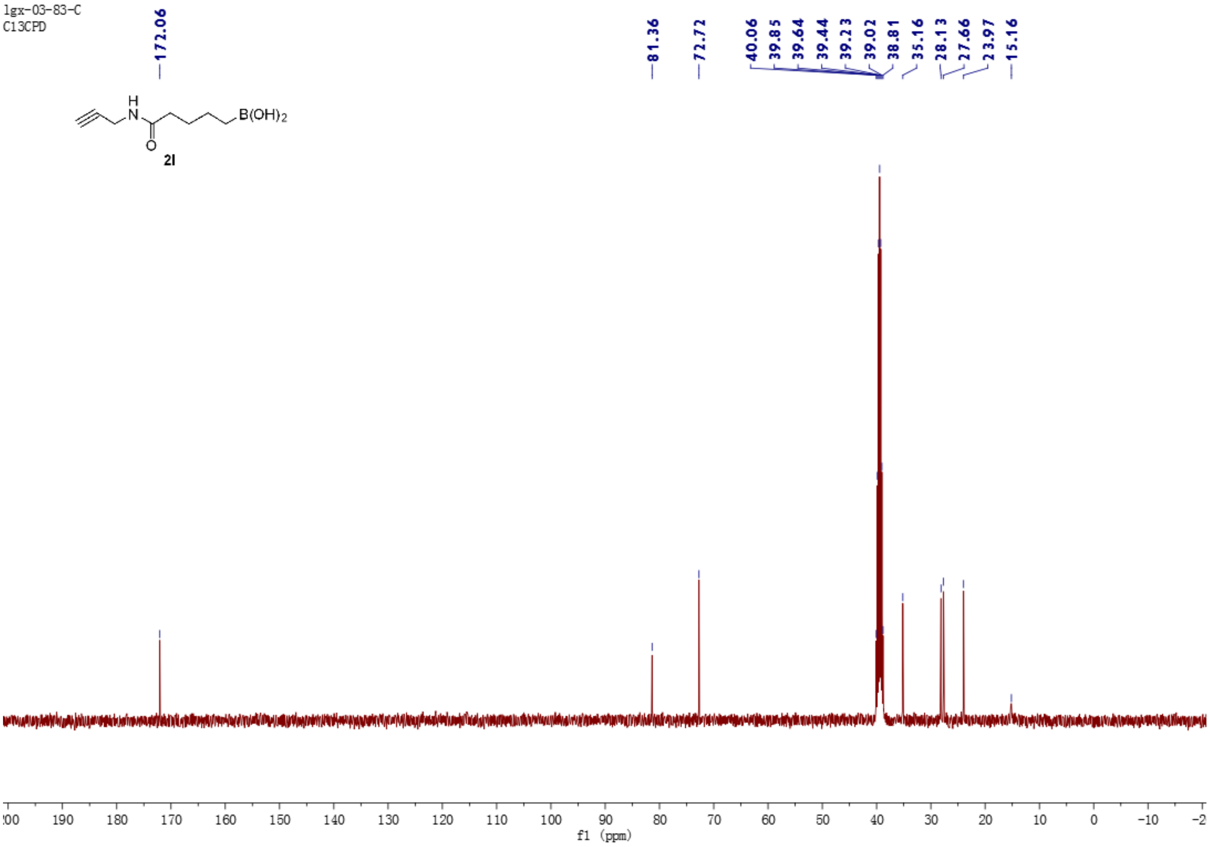
lgx-03-83-C
C13CPD
172.06
81.36
72.72
40.06
39.85
39.64
39.44
39.23
39.02
38.81
35.16
28.13
27.66
23.97
15.16
B(OH)2
H
N
O
2l
f1 (ppm)

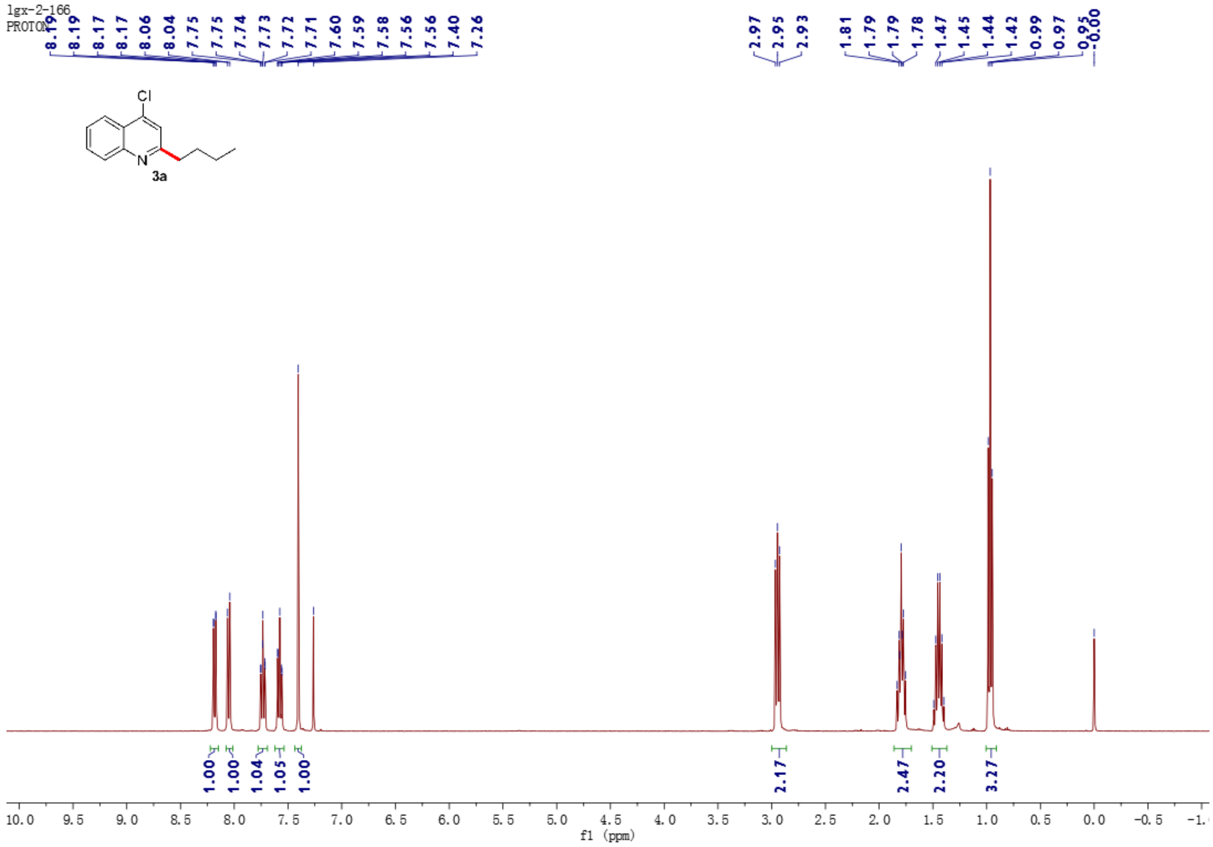
lgx-2-166
PROTON
8.19
8.19
8.17
8.17
8.06
8.04
7.75
7.75
7.74
7.73
7.72
7.71
7.60
7.59
7.58
7.56
7.56
7.40
7.26
2.97
2.95
2.93
1.81
1.79
1.79
1.78
1.47
1.45
1.44
1.42
0.99
0.97
0.95
0.00
Cl
N
3a
1.00
1.00
1.04
1.05
1.00
2.17
2.47
2.20
3.27
f1 (ppm)

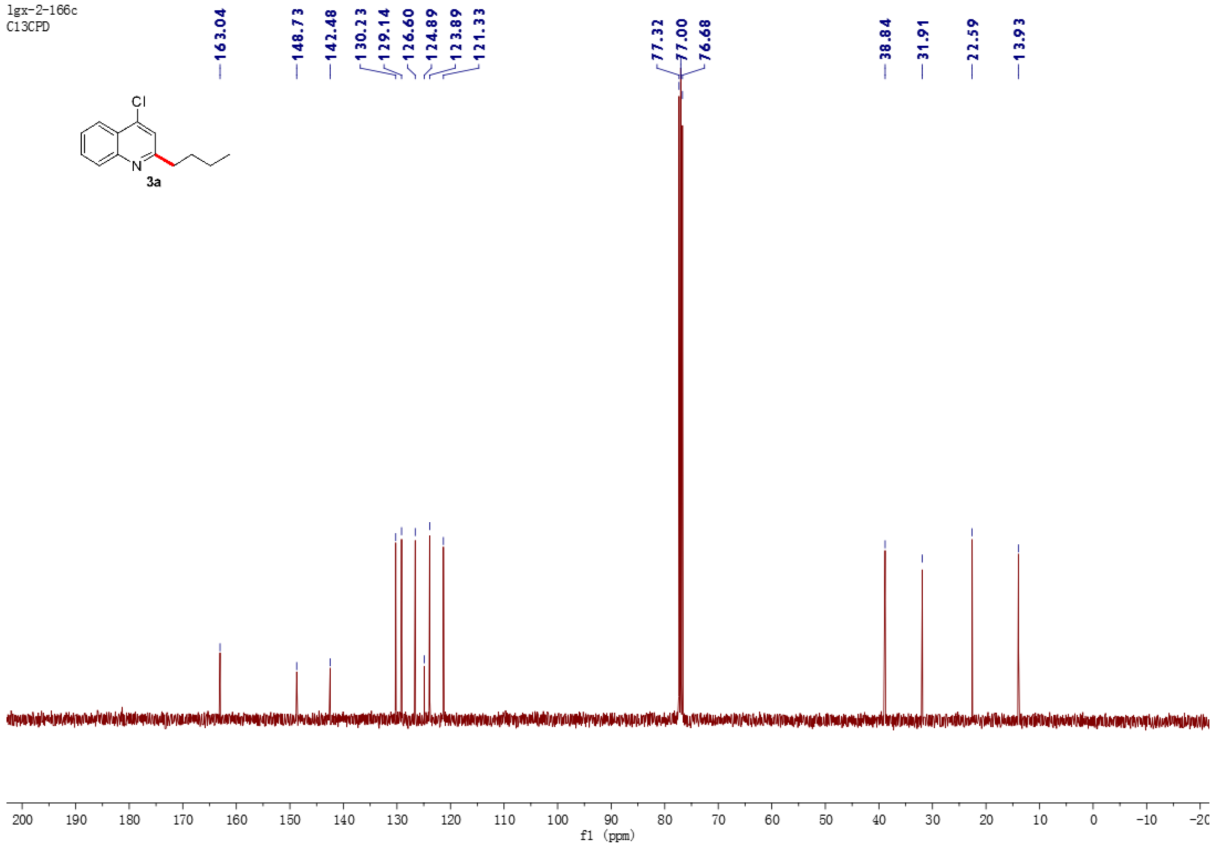
lgx-2-166c
C13CPD
163.04
148.73
142.48
130.23
129.14
126.60
124.89
123.89
121.33
77.32
77.00
76.68
38.84
31.91
22.59
13.93
Cl
N
3a
f1 (ppm)

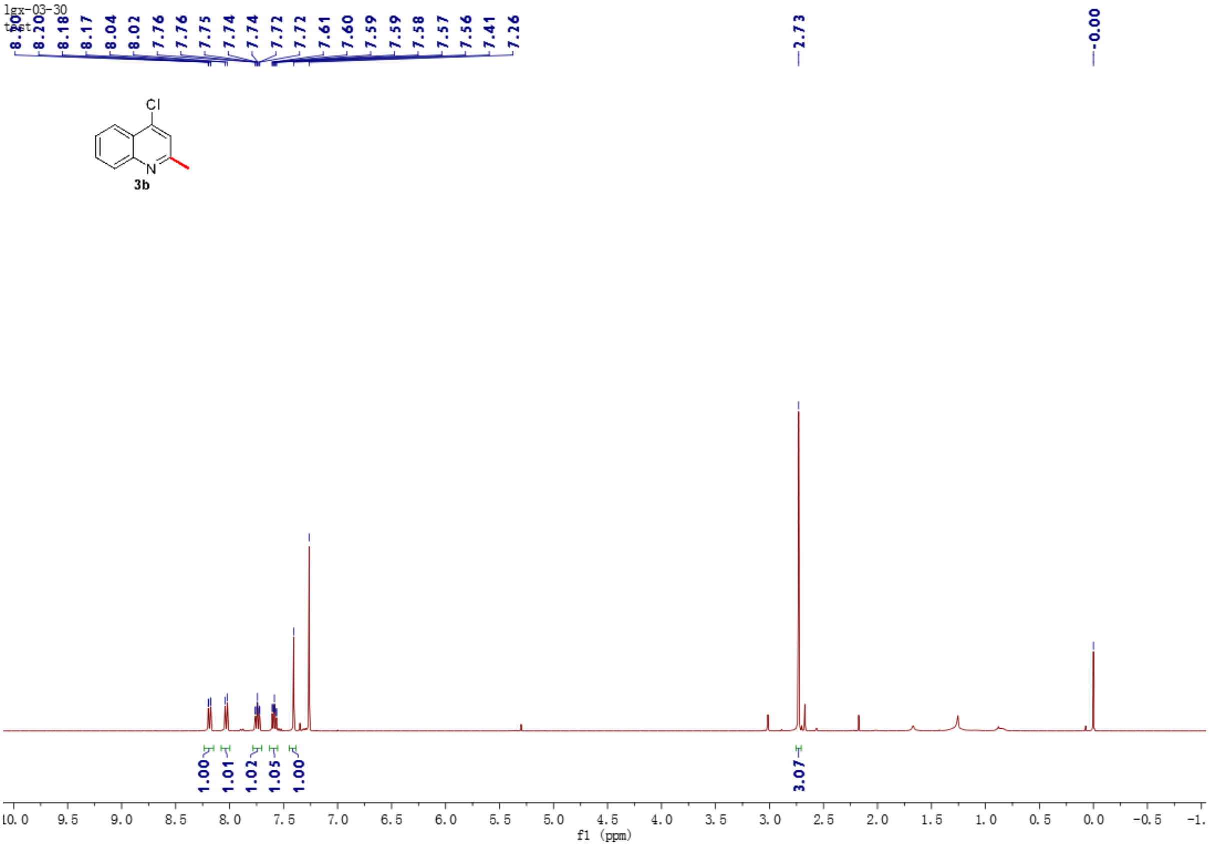
lgx-03-30
test
8.20
8.18
8.17
8.04
8.02
7.76
7.76
7.75
7.74
7.74
7.72
7.72
7.61
7.60
7.59
7.59
7.58
7.57
7.56
7.41
7.26
2.73
-0.00
Cl
N
3b
1.00
1.01
1.02
1.05
1.00
3.07
10.0 9.5 9.0 8.5 8.0 7.5 7.0 6.5 6.0 5.5 5.0 4.5 4.0 3.5 3.0 2.5 2.0 1.5 1.0 0.5 0.0 -0.5 -1.
f1 (ppm)

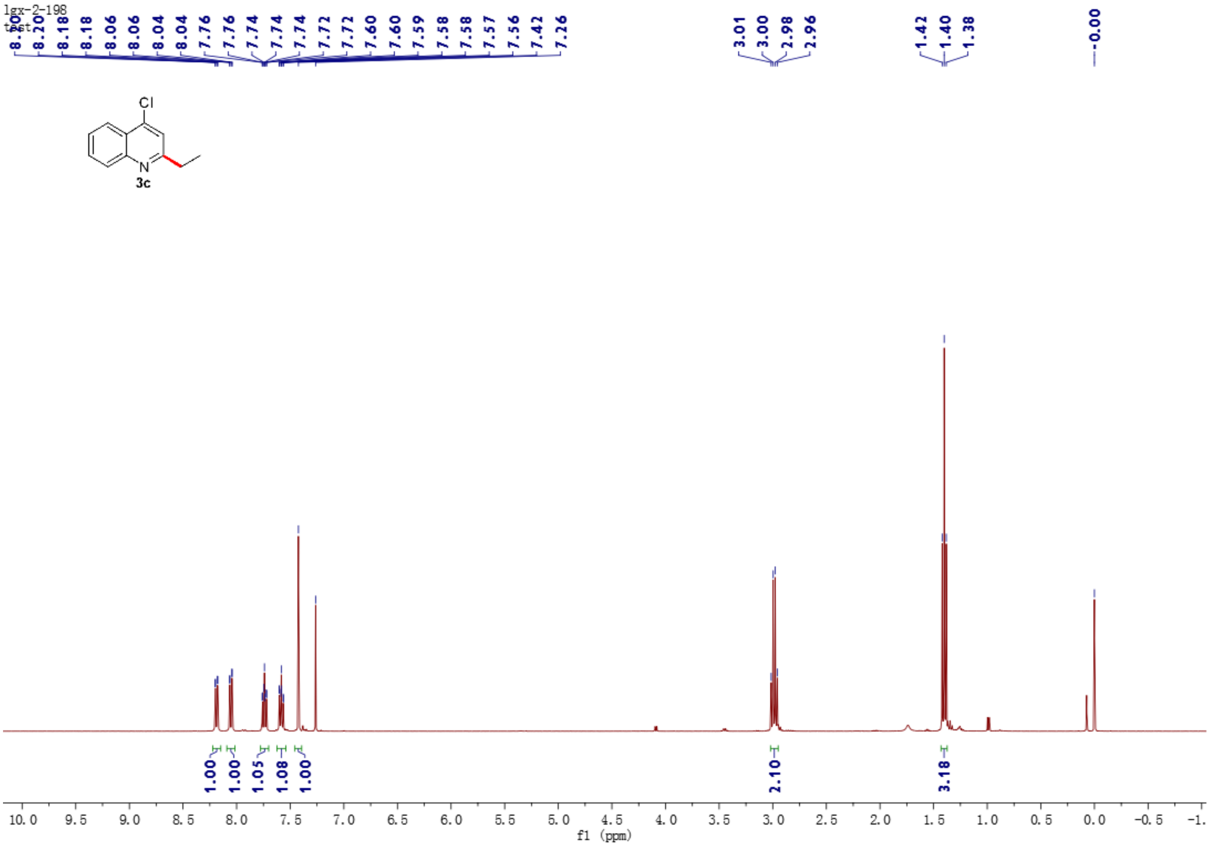
lgx-2-198
test
8.20
8.18
8.18
8.06
8.06
8.04
8.04
7.76
7.76
7.74
7.74
7.74
7.72
7.72
7.60
7.60
7.59
7.58
7.58
7.57
7.56
7.42
7.26
3.01
3.00
2.98
2.96
1.42
1.40
1.38
-0.00
Cl
N
3c
1.00
1.00
1.05
1.08
1.00
2.10
3.18
10.0 9.5 9.0 8.5 8.0 7.5 7.0 6.5 6.0 5.5 5.0 4.5 4.0 3.5 3.0 2.5 2.0 1.5 1.0 0.5 0.0 -0.5 -1.
f1 (ppm)

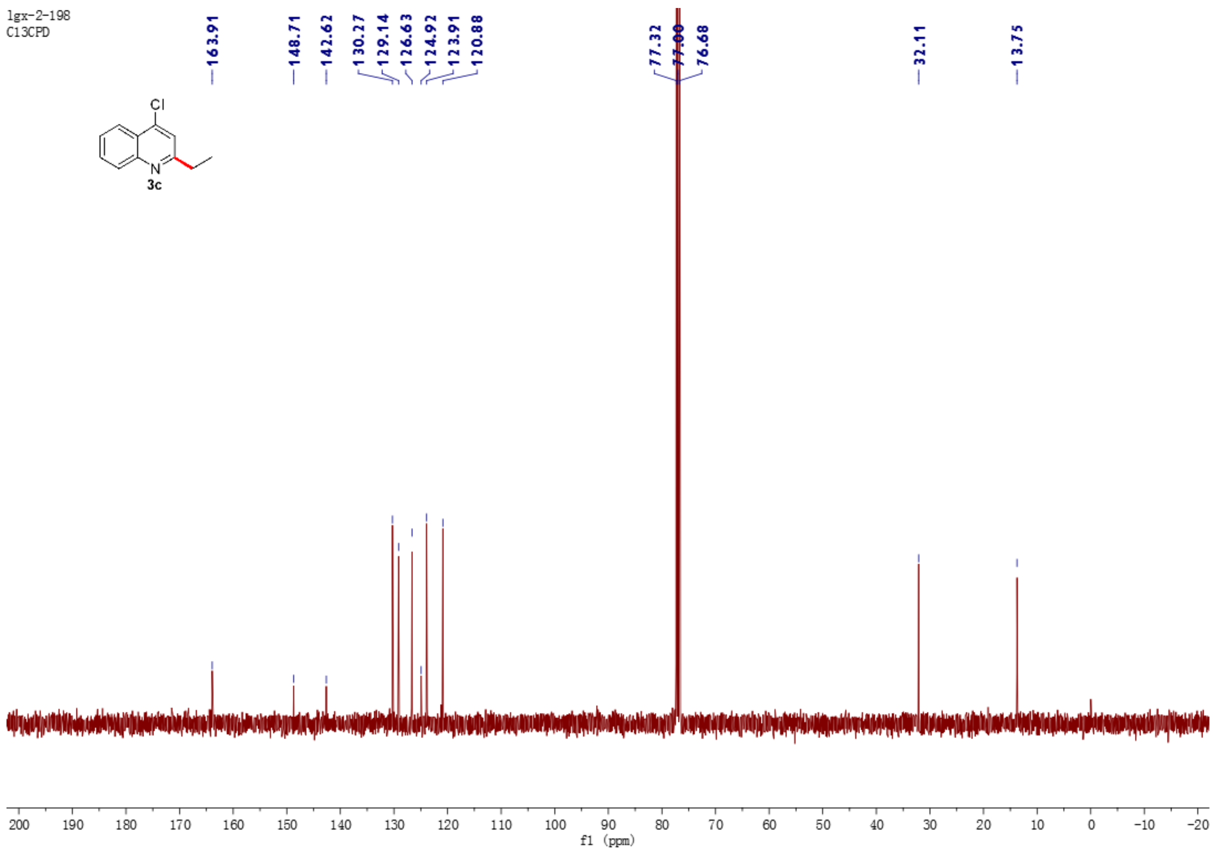

lgx-2-198
C13CPD
163.91
148.71
142.62
130.27
129.14
126.63
124.92
123.91
120.88
77.32
77.00
76.68
32.11
13.75
Cl
N
3c
f1 (ppm)

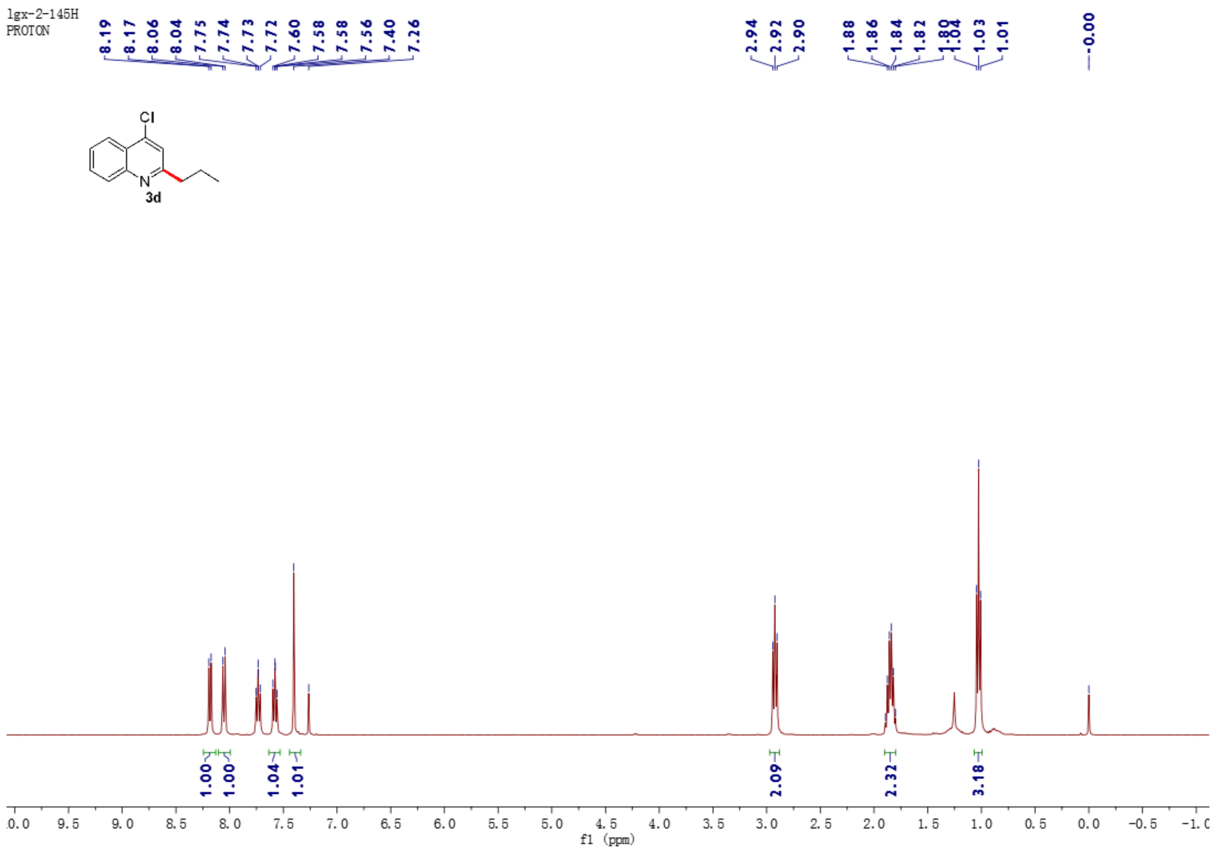

lgx-2-145H
PROTON
8.19
8.17
8.06
8.04
7.75
7.74
7.73
7.72
7.60
7.58
7.58
7.56
7.40
7.26
2.94
2.92
2.90
1.88
1.86
1.84
1.82
1.80
1.04
1.03
1.01
0.00
Cl
N
3d
1.00
1.00
1.04
1.01
2.09
2.32
3.18
f1 (ppm)

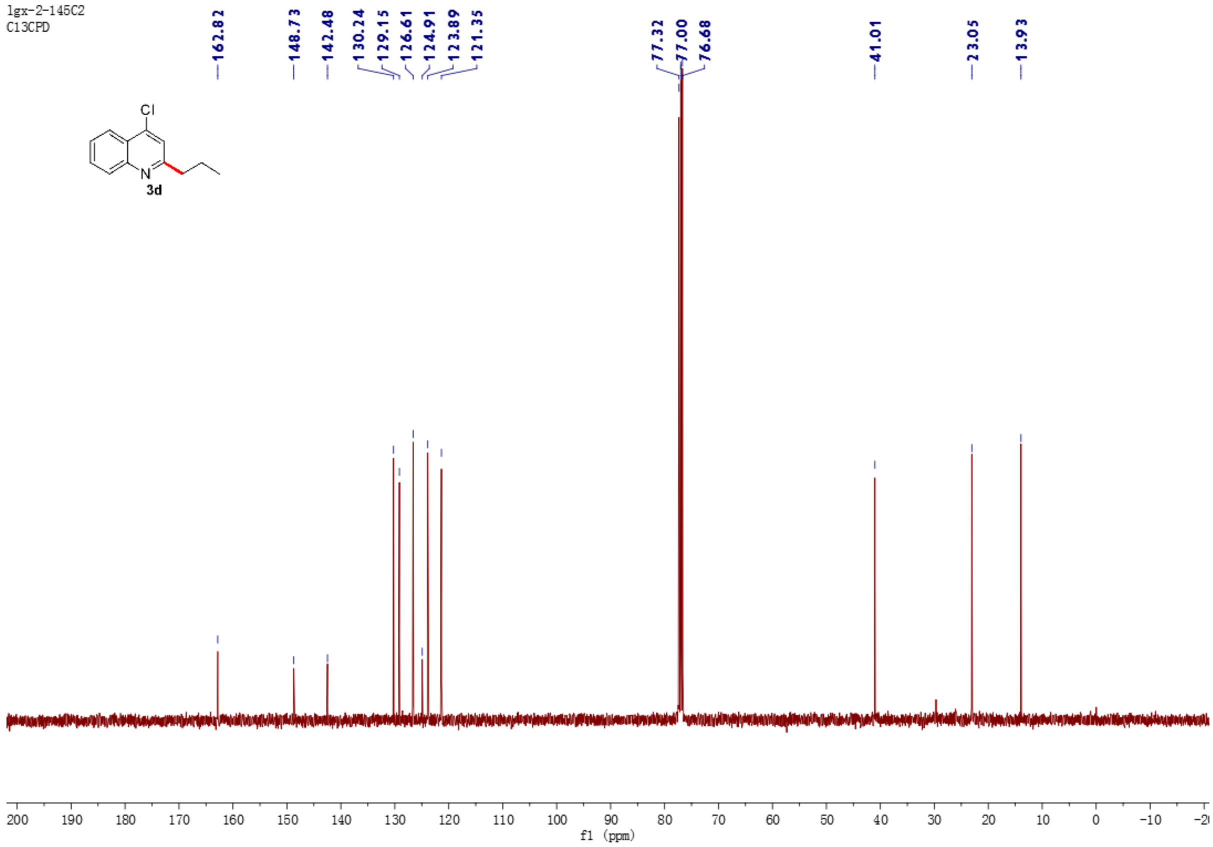
lgx-2-145C2
C13CPD
162.82
148.73
142.48
130.24
129.15
126.61
124.91
123.89
121.35
77.32
77.00
76.68
41.01
23.05
13.93
Cl
N
3d
f1 (ppm)

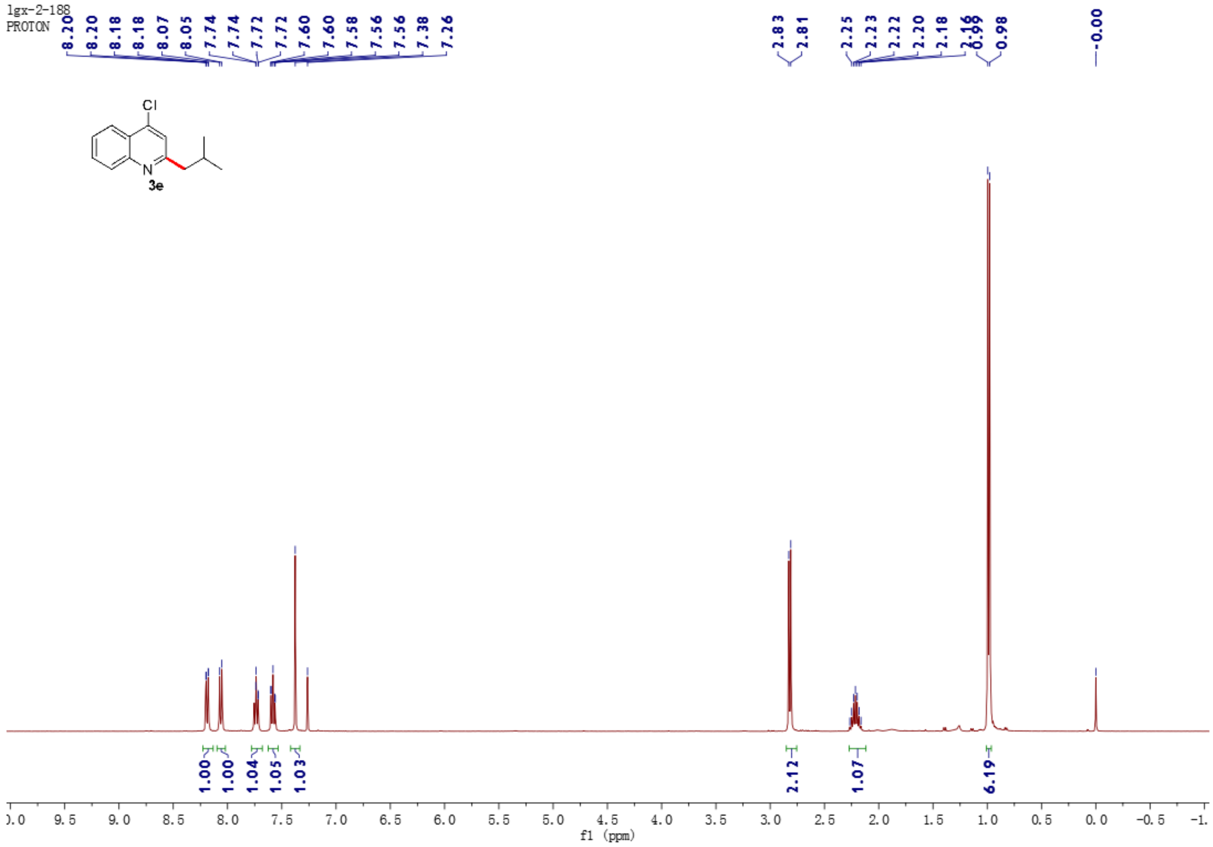
lgx-2-188
PROTON
8.20
8.20
8.18
8.18
8.07
8.05
7.74
7.74
7.72
7.72
7.60
7.60
7.58
7.56
7.56
7.38
7.26
2.83
2.81
2.25
2.23
2.22
2.20
2.18
0.98
-0.00
Cl
N
3e
1.00
1.00
1.04
1.05
1.03
2.12
1.07
6.19
f1 (ppm)

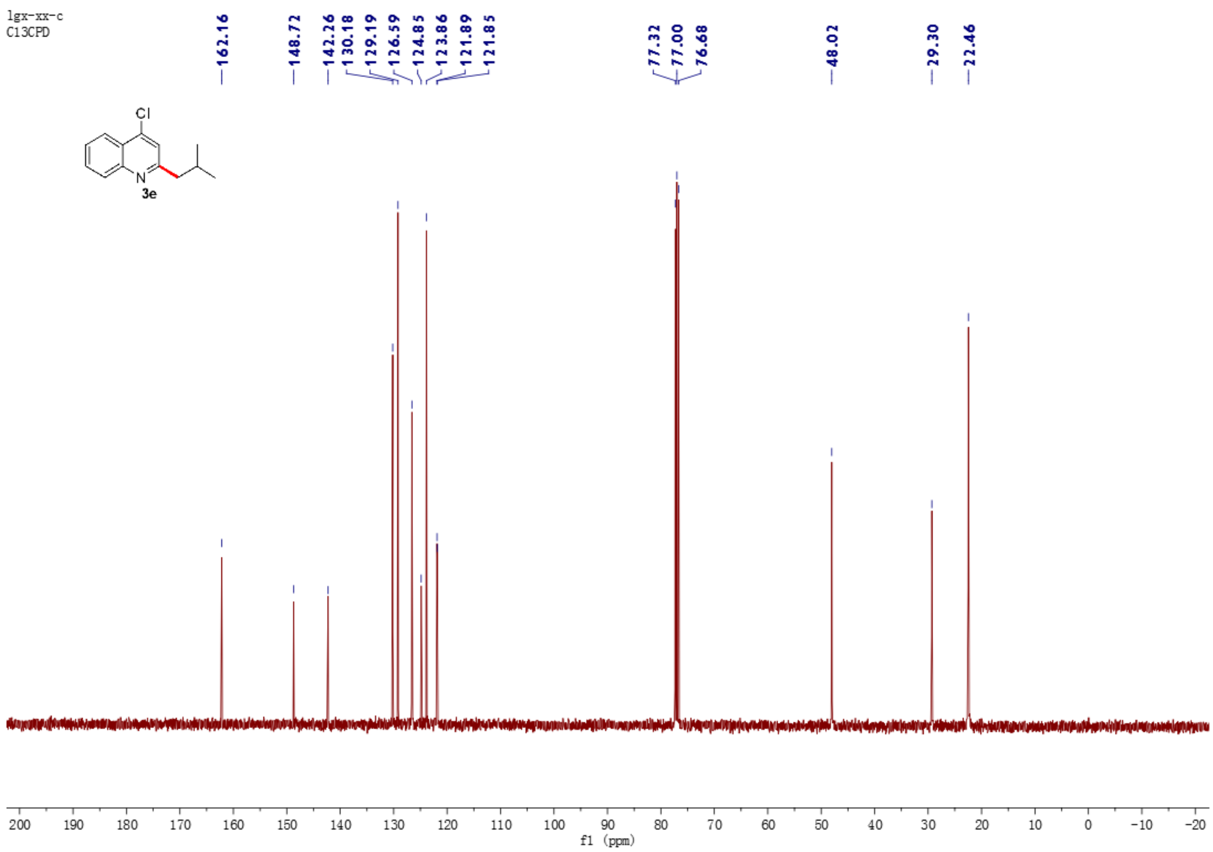

lgx-xx-c
C13CPD
162.16
148.72
142.26
130.18
129.19
126.59
124.85
123.86
121.89
121.85
77.32
77.00
76.68
48.02
29.30
22.46
Cl
N
3e
f1 (ppm)

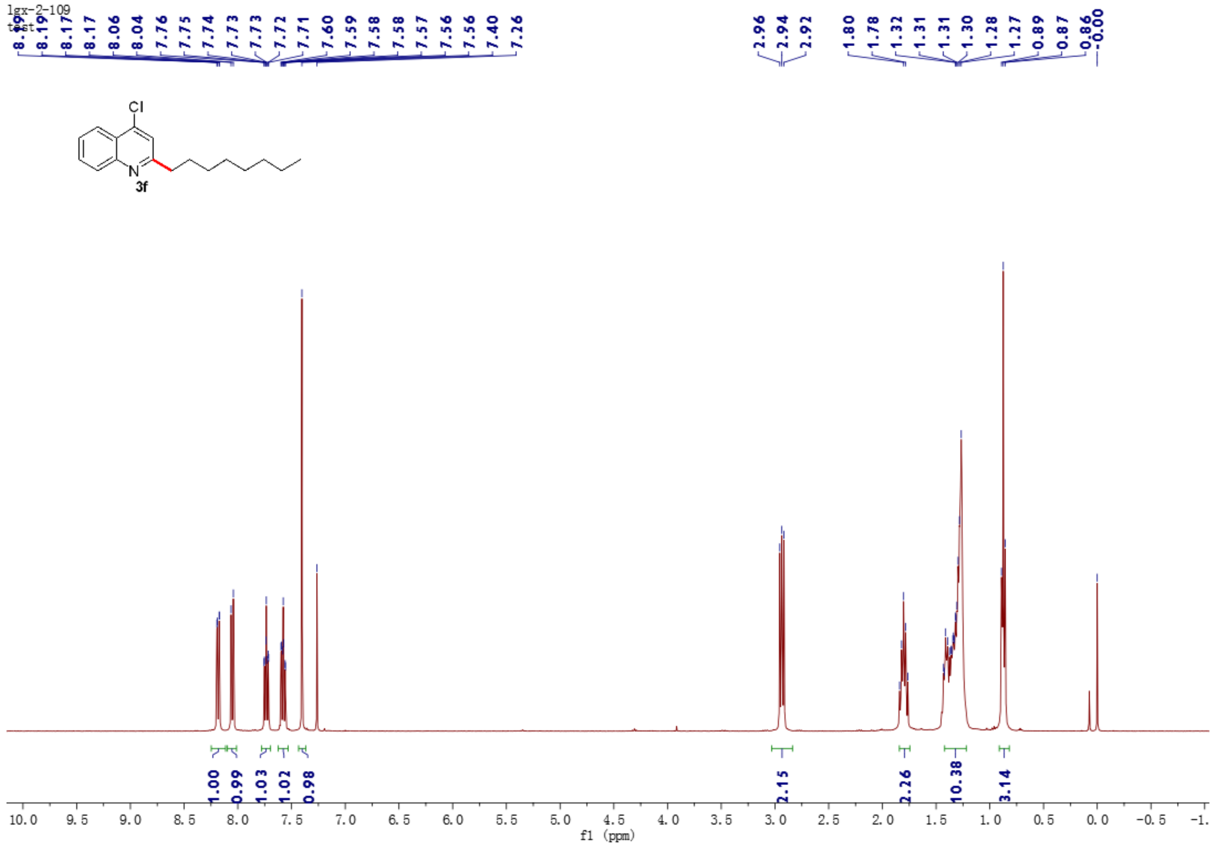

lgx-2-109
test
8.19
8.17
8.17
8.06
8.04
7.76
7.75
7.74
7.73
7.73
7.72
7.71
7.60
7.59
7.58
7.58
7.57
7.56
7.56
7.40
7.26
2.96
2.94
2.92
1.80
1.78
1.32
1.31
1.31
1.30
1.28
1.27
0.89
0.87
0.86
-0.00
Cl
N
3f
1.00
0.99
1.03
1.02
0.98
2.15
2.26
10.38
3.14
f1 (ppm)

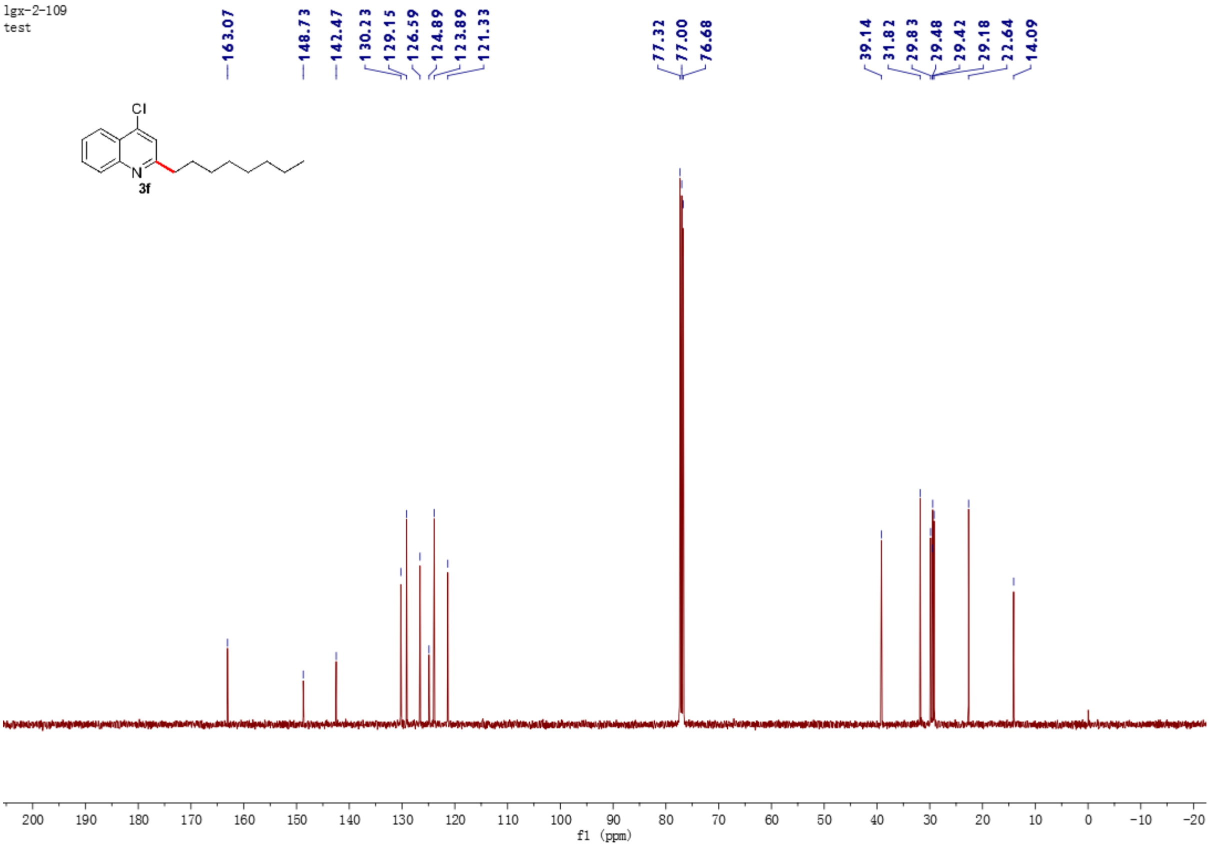
lgx-2-109
test
163.07
148.73
142.47
130.23
129.15
126.59
124.89
123.89
121.33
77.32
77.00
76.68
39.14
31.82
29.83
29.48
29.42
29.18
22.64
14.09
Cl
N
3f
f1 (ppm)

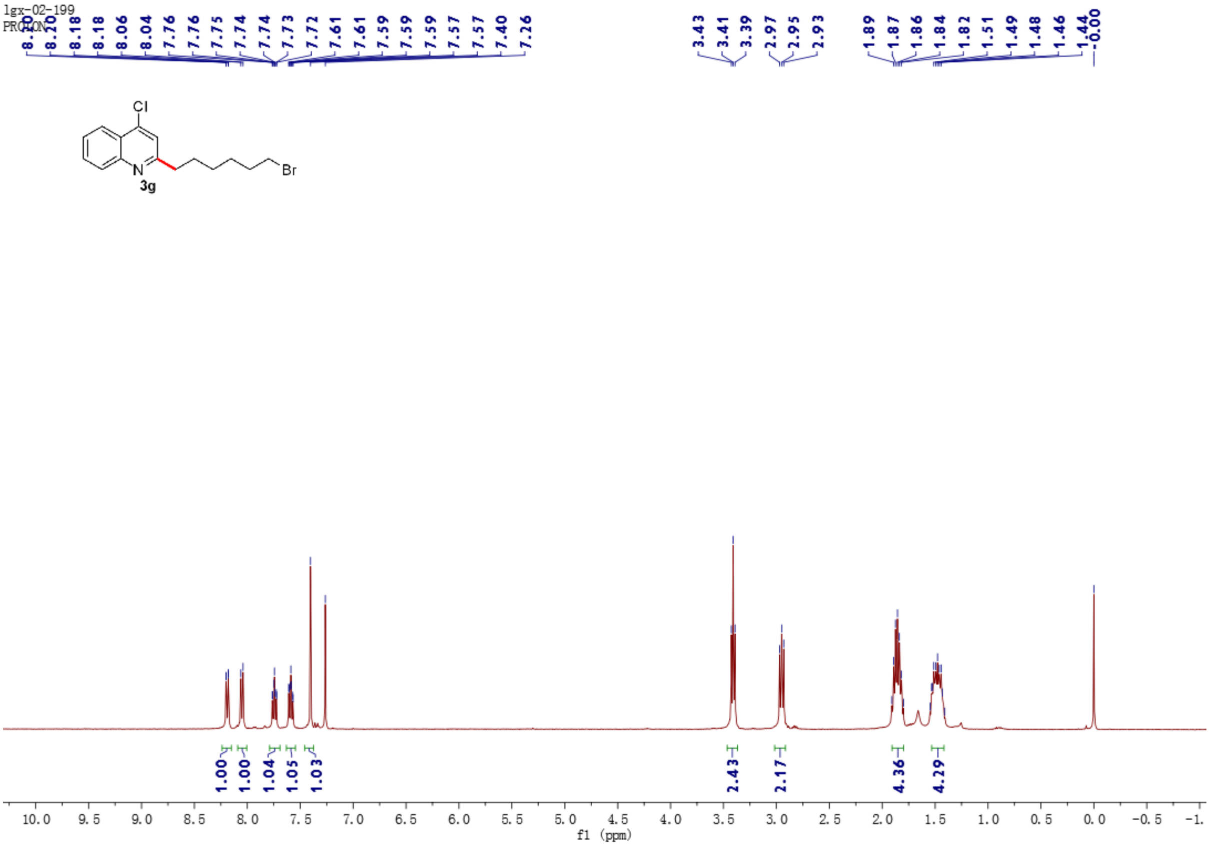
lgx-02-199
8.20
8.18
8.18
8.06
8.04
7.76
7.76
7.75
7.74
7.74
7.73
7.72
7.61
7.61
7.59
7.59
7.59
7.57
7.57
7.40
7.26
3.43
3.41
3.39
2.97
2.95
2.93
1.89
1.87
1.86
1.84
1.82
1.51
1.49
1.48
1.46
1.44
0.00
Cl
N
Br
3g
1.00
1.00
1.04
1.05
1.03
2.43
2.17
4.36
4.29
f1 (ppm)

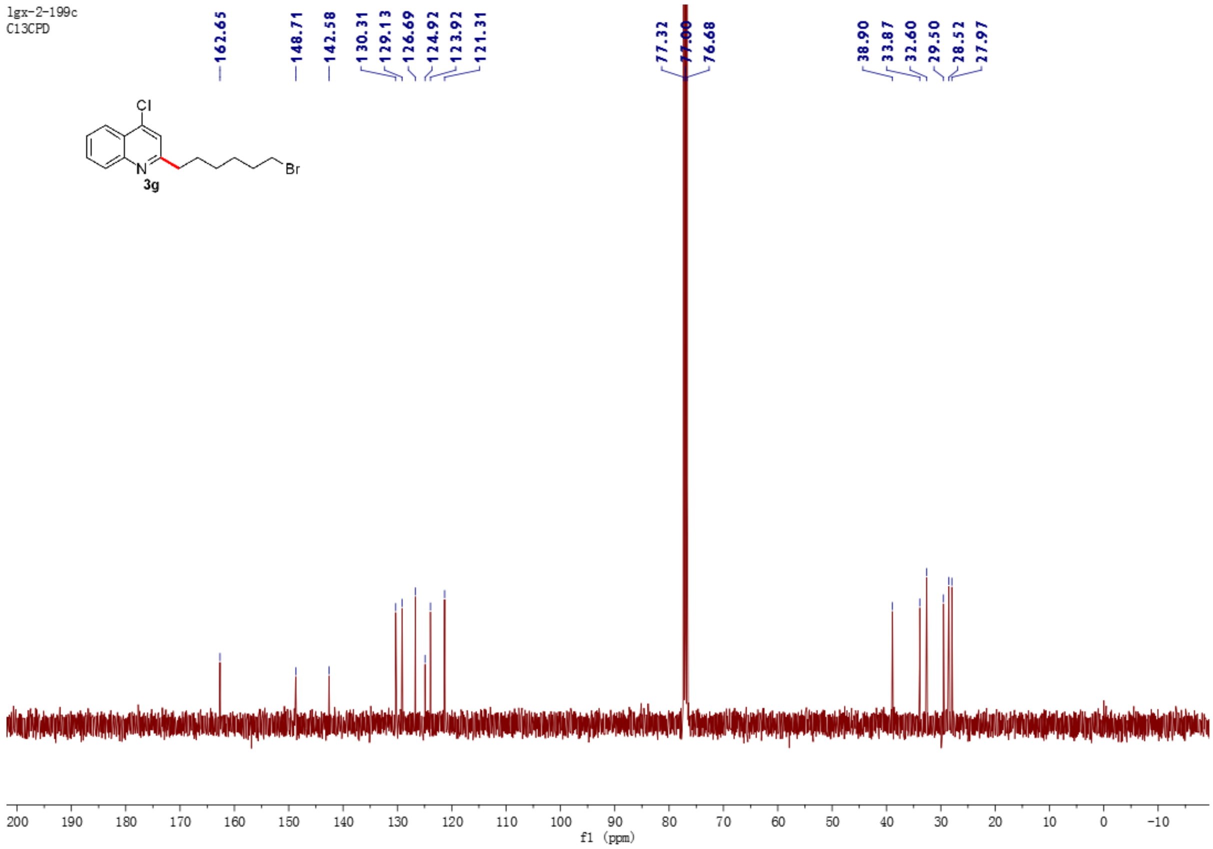
lgx-2-199c
C13CPD
162.65
148.71
142.58
130.31
129.13
126.69
124.92
123.92
121.31
77.32
77.00
76.68
38.90
33.87
32.60
29.50
28.52
27.97
Cl
N
Br
3g
f1 (ppm)

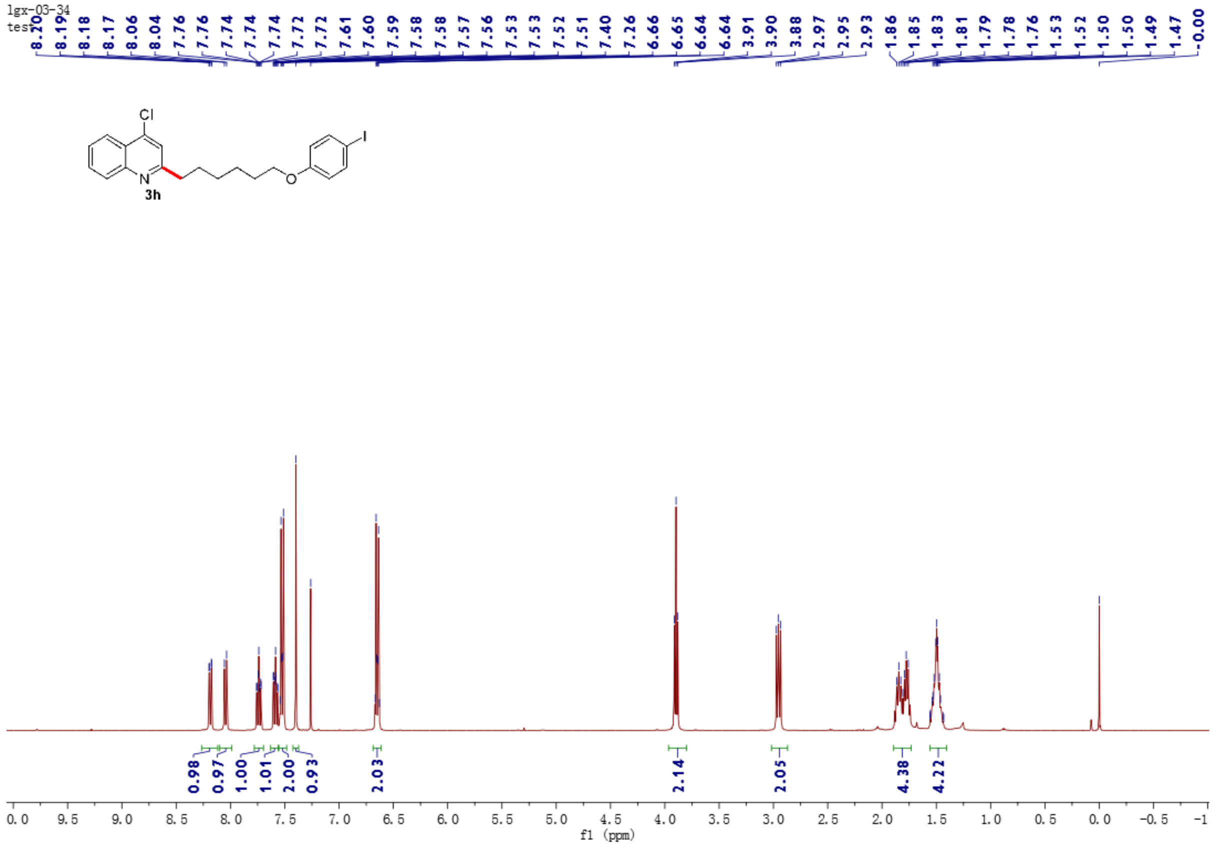
lgx-03-34
test
Cl
N
O
I
3h
0.98
0.97
1.00
1.01
2.00
0.93
2.03
2.14
2.05
4.38
4.22
f1 (ppm)

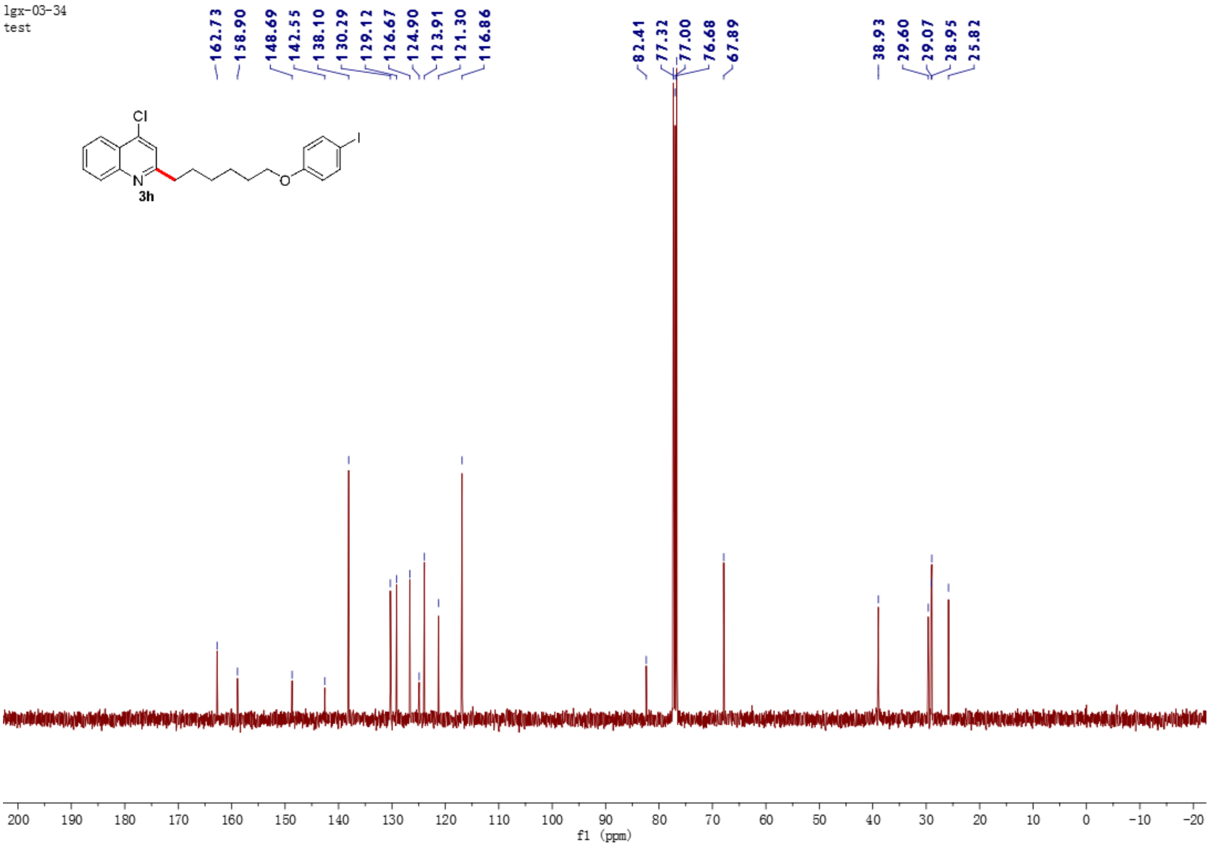
lgx-03-34
test
162.73
158.90
148.69
142.55
138.10
130.29
129.12
126.67
124.90
123.91
121.30
116.86
82.41
77.32
77.00
76.68
67.89
38.93
29.60
29.07
28.95
25.82
Cl
N
O
I
3h
f1 (ppm)

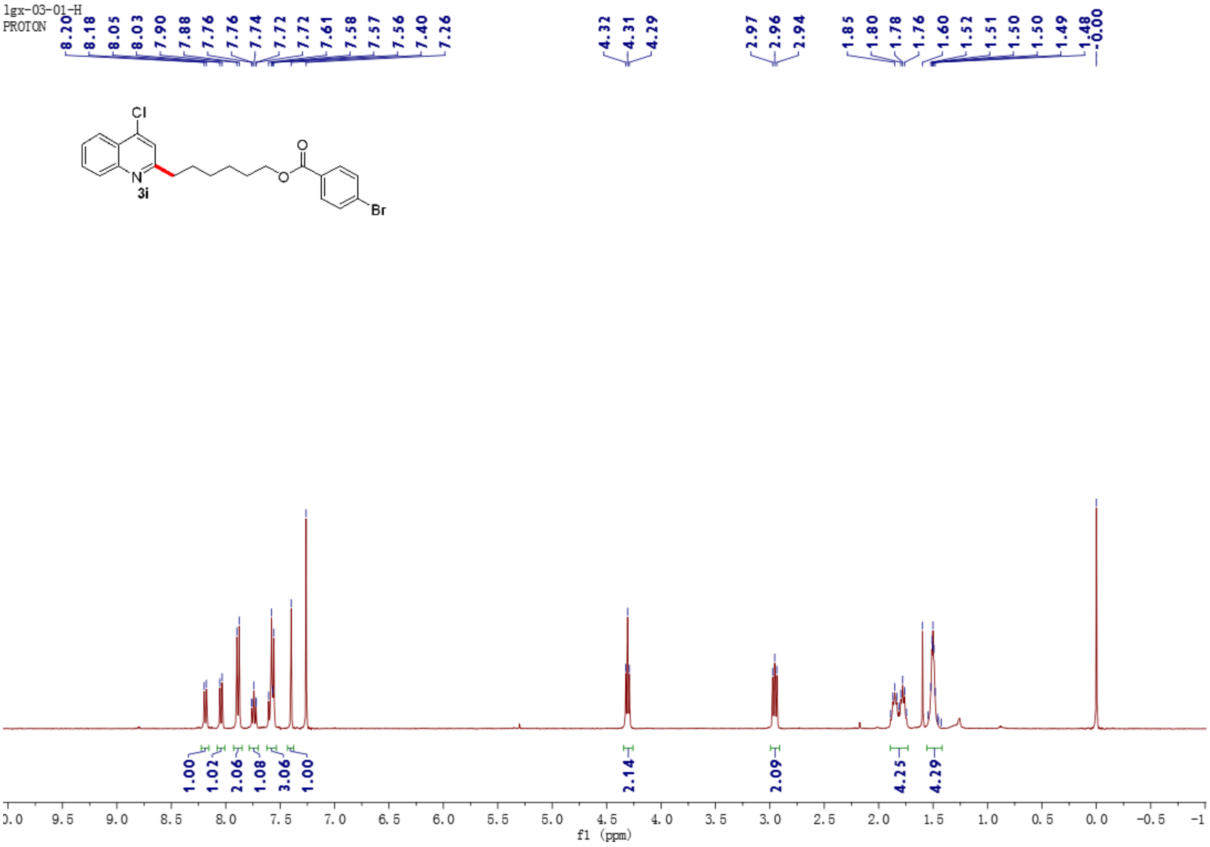
lgx-03-01-H
PROTON
8.20
8.18
8.05
8.03
7.90
7.88
7.76
7.76
7.74
7.72
7.72
7.61
7.58
7.57
7.56
7.40
7.26
4.32
4.31
4.29
2.97
2.96
2.94
1.85
1.80
1.78
1.76
1.60
1.52
1.51
1.50
1.50
1.49
1.48
0.00
Cl
N
O
O
Br
3i
1.00
1.02
2.06
1.08
3.06
1.00
2.14
2.09
4.25
4.29
f1 (ppm)

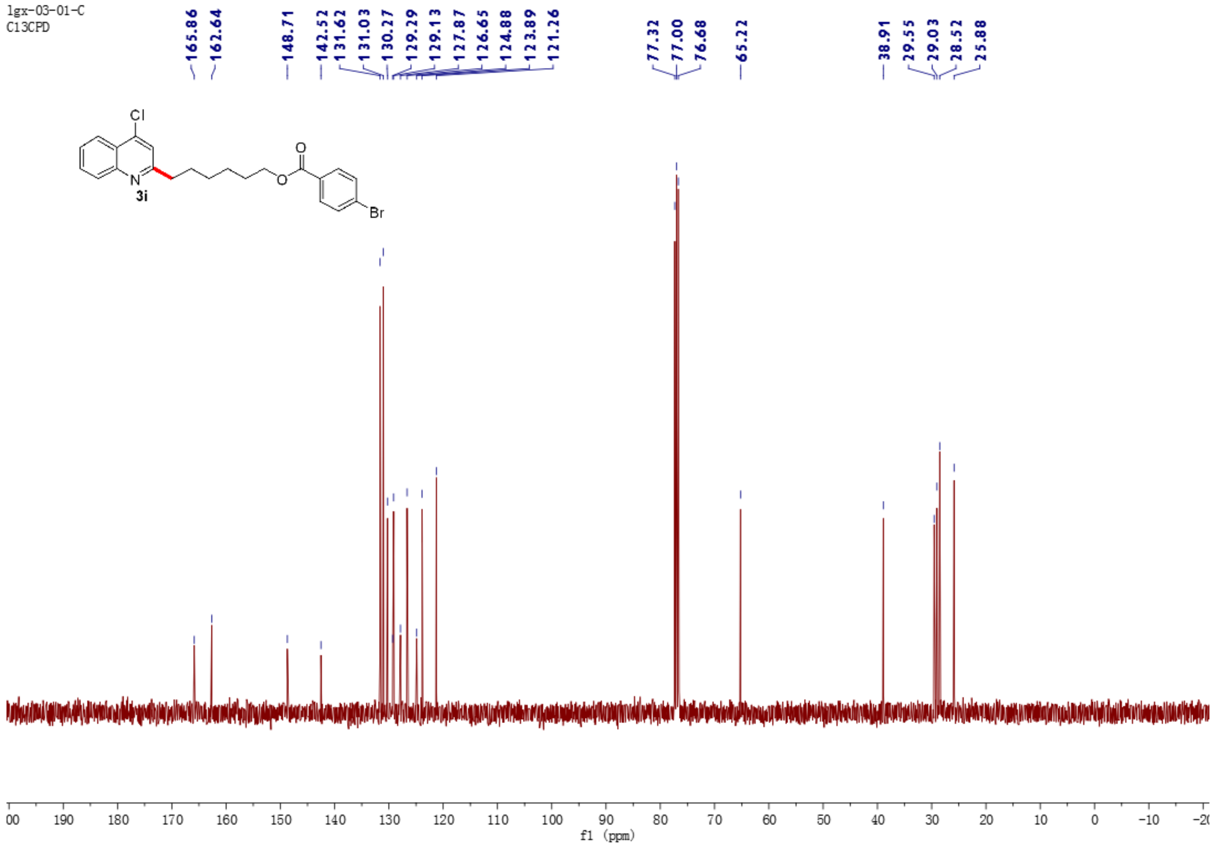
lgx-03-01-C
C13CPD
165.86
162.64
148.71
142.52
131.62
131.03
130.27
129.29
129.13
127.87
126.65
124.88
123.89
121.26
77.32
77.00
76.68
65.22
38.91
29.55
29.03
28.52
25.88
Cl
N
O
Br
3i
f1 (ppm)

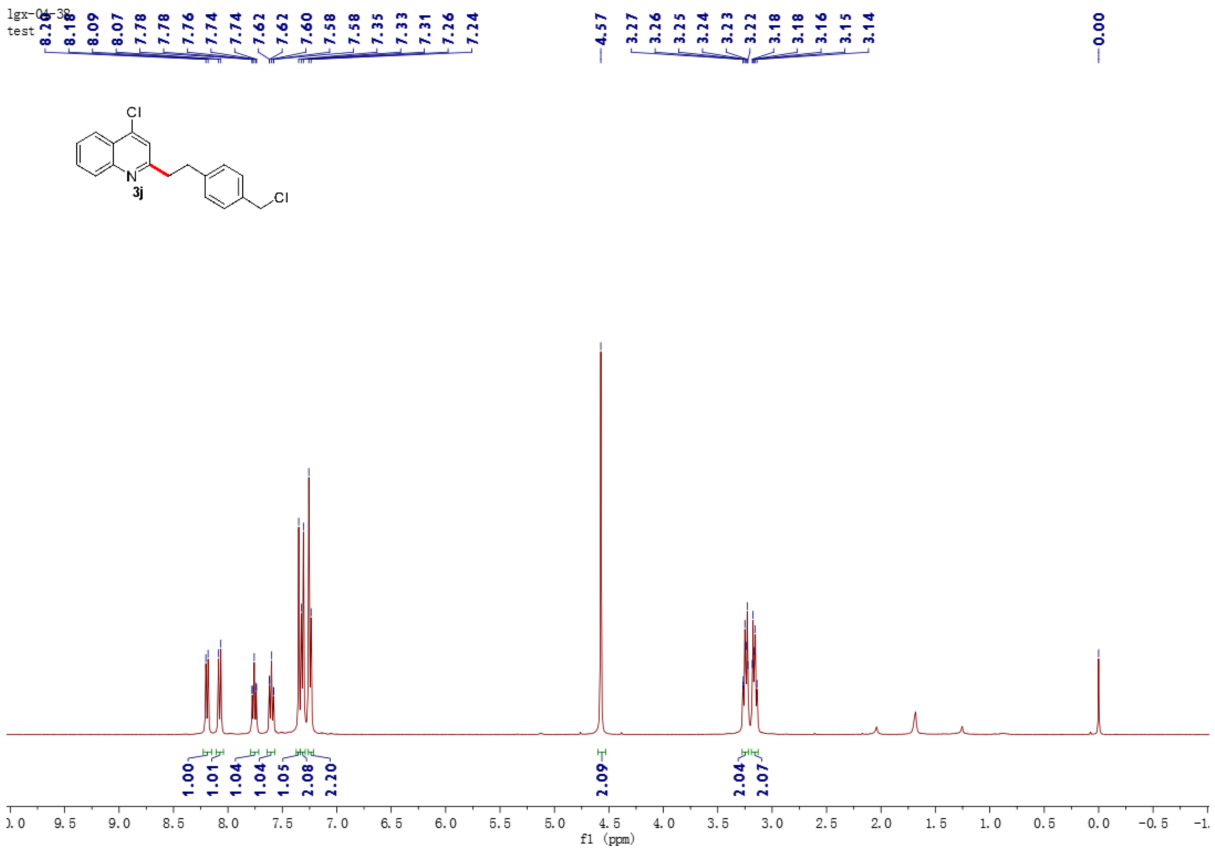
test
8.18
8.09
8.07
7.78
7.78
7.76
7.74
7.74
7.62
7.62
7.60
7.58
7.58
7.35
7.33
7.31
7.26
7.24
4.57
3.27
3.26
3.25
3.24
3.23
3.22
3.18
3.18
3.16
3.15
3.14
0.00
Cl
N
Cl
3j
1.00
1.01
1.04
1.04
1.05
2.08
2.20
2.09
2.04
2.07
f1 (ppm)

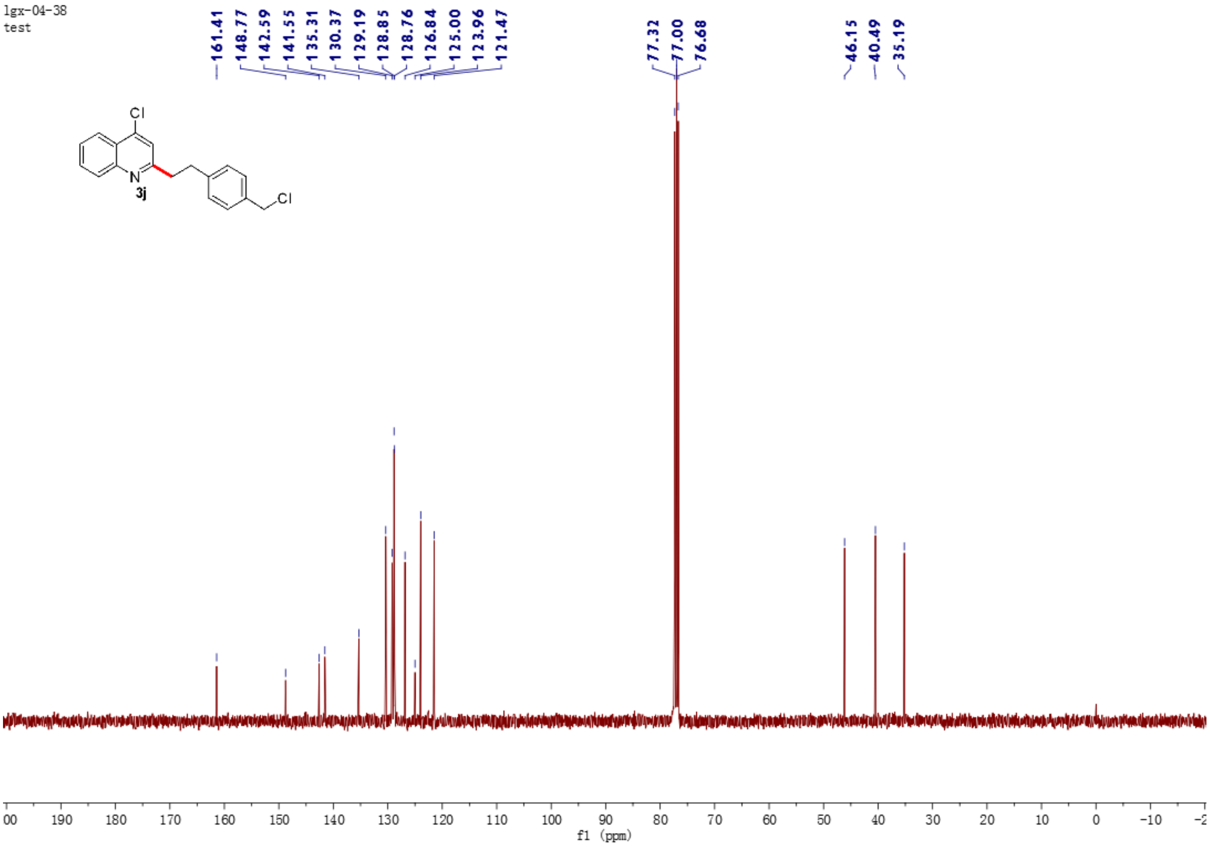
lgx-04-38
test
161.41
148.77
142.59
141.55
135.31
130.37
129.19
128.85
128.76
126.84
125.00
123.96
121.47
77.32
77.00
76.68
46.15
40.49
35.19
Cl
N
3j
Cl
f1 (ppm)

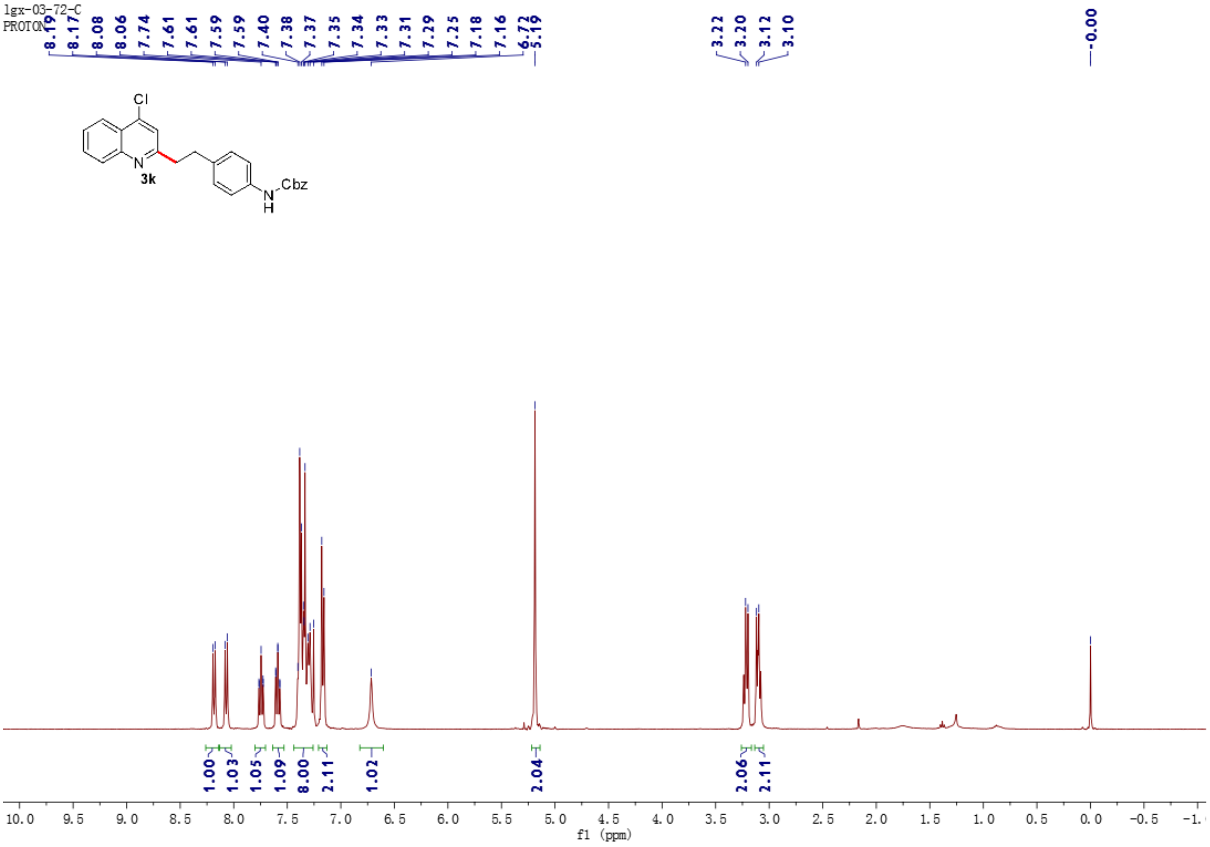
lgx-03-72-C
PROTON
8.19
8.17
8.08
8.06
7.74
7.61
7.59
7.59
7.40
7.38
7.37
7.35
7.34
7.33
7.31
7.29
7.25
7.18
7.16
6.72
5.19
3.22
3.20
3.12
3.10
-0.00
Cl
N
3k
N
H
Cbz
1.00
1.03
1.05
1.09
8.00
2.11
1.02
2.04
2.06
2.11
f1 (ppm)

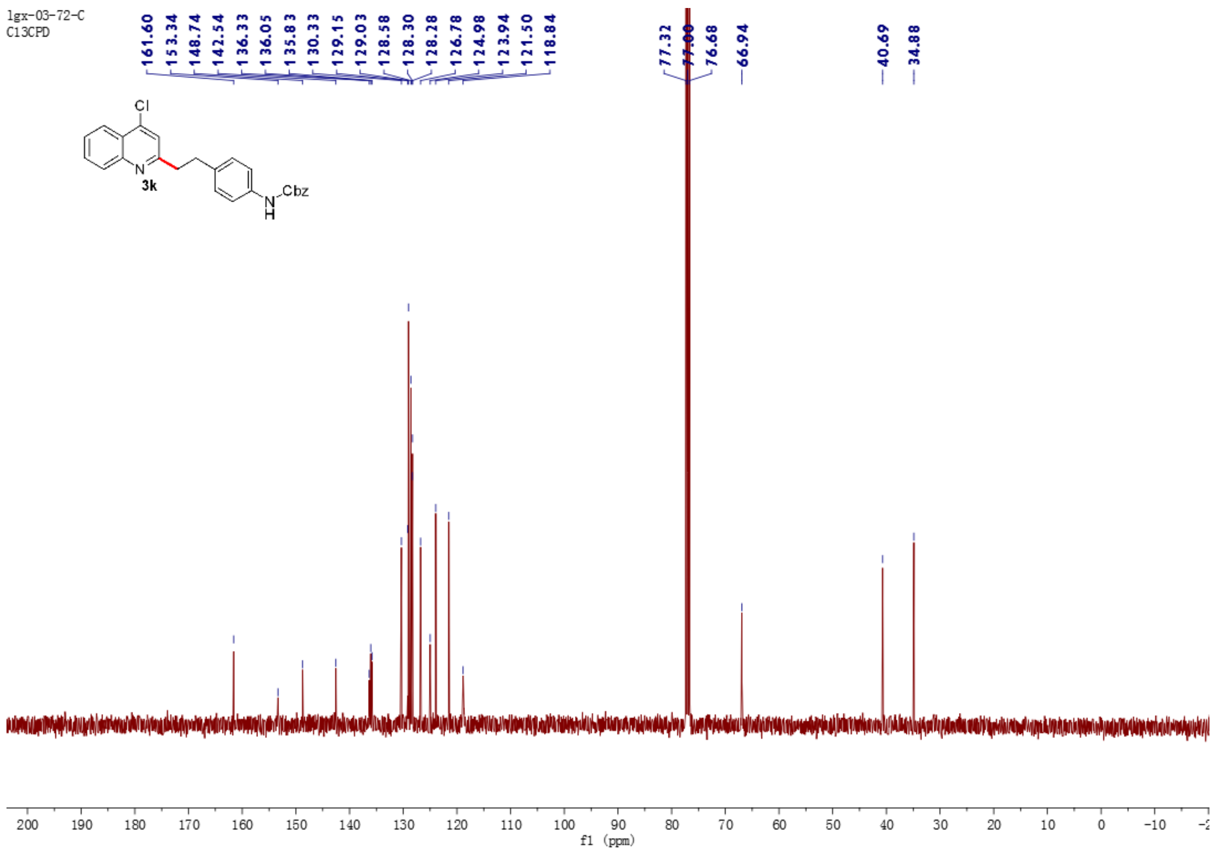
lgx-03-72-C
C13CPD
161.60
153.34
148.74
142.54
136.33
136.05
135.83
130.33
129.15
129.03
128.58
128.30
128.28
126.78
124.98
123.94
121.50
118.84
77.32
77.00
76.68
66.94
40.69
34.88
Cl
N
3k
N
H
Cbz
f1 (ppm)

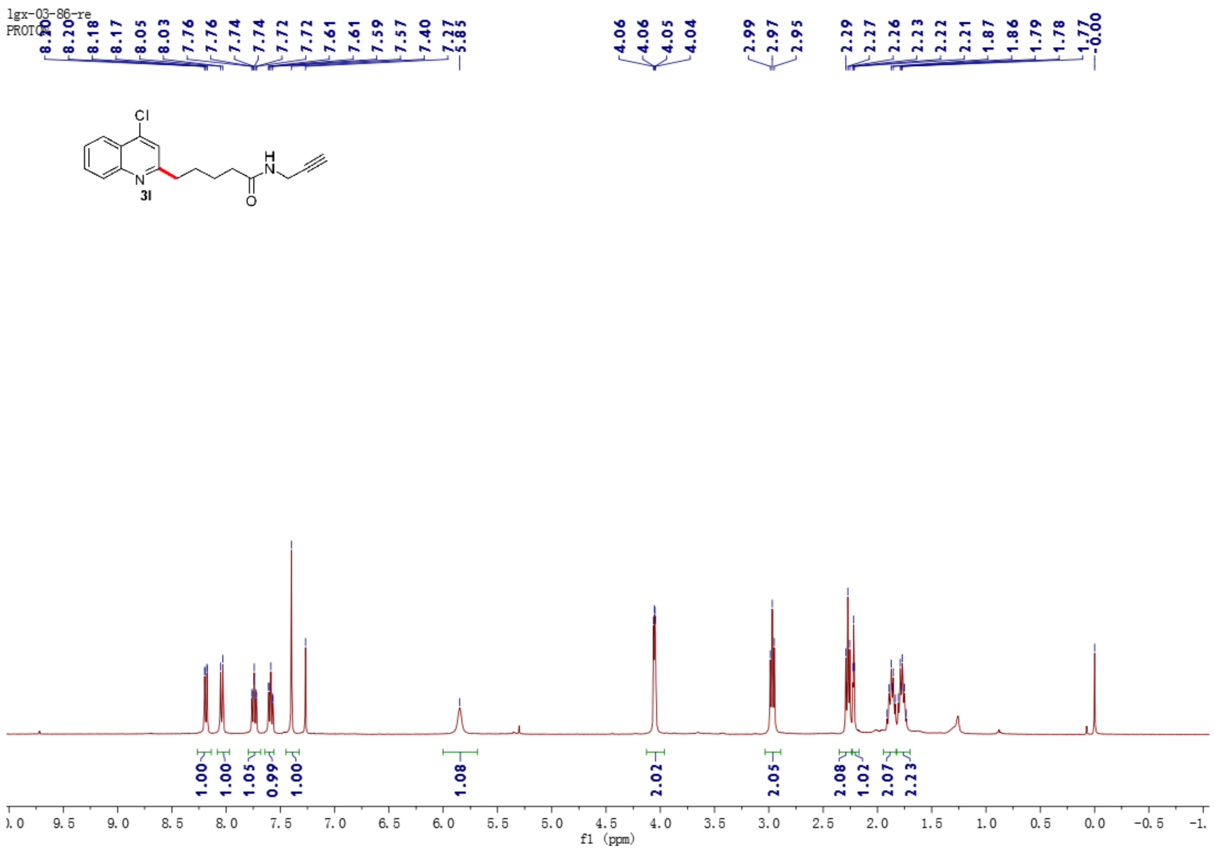
lgx-03-86-re
PROTON
8.20
8.18
8.17
8.05
8.03
7.76
7.76
7.74
7.74
7.72
7.72
7.61
7.61
7.59
7.57
7.40
7.27
5.85
4.06
4.06
4.05
4.04
2.99
2.97
2.95
2.29
2.27
2.26
2.23
2.22
2.21
1.87
1.86
1.79
1.78
1.77
0.00
Cl
N
3l
H
N
O
1.00
1.00
1.05
0.99
1.00
1.08
2.02
2.05
2.08
1.02
2.07
2.23
f1 (ppm)

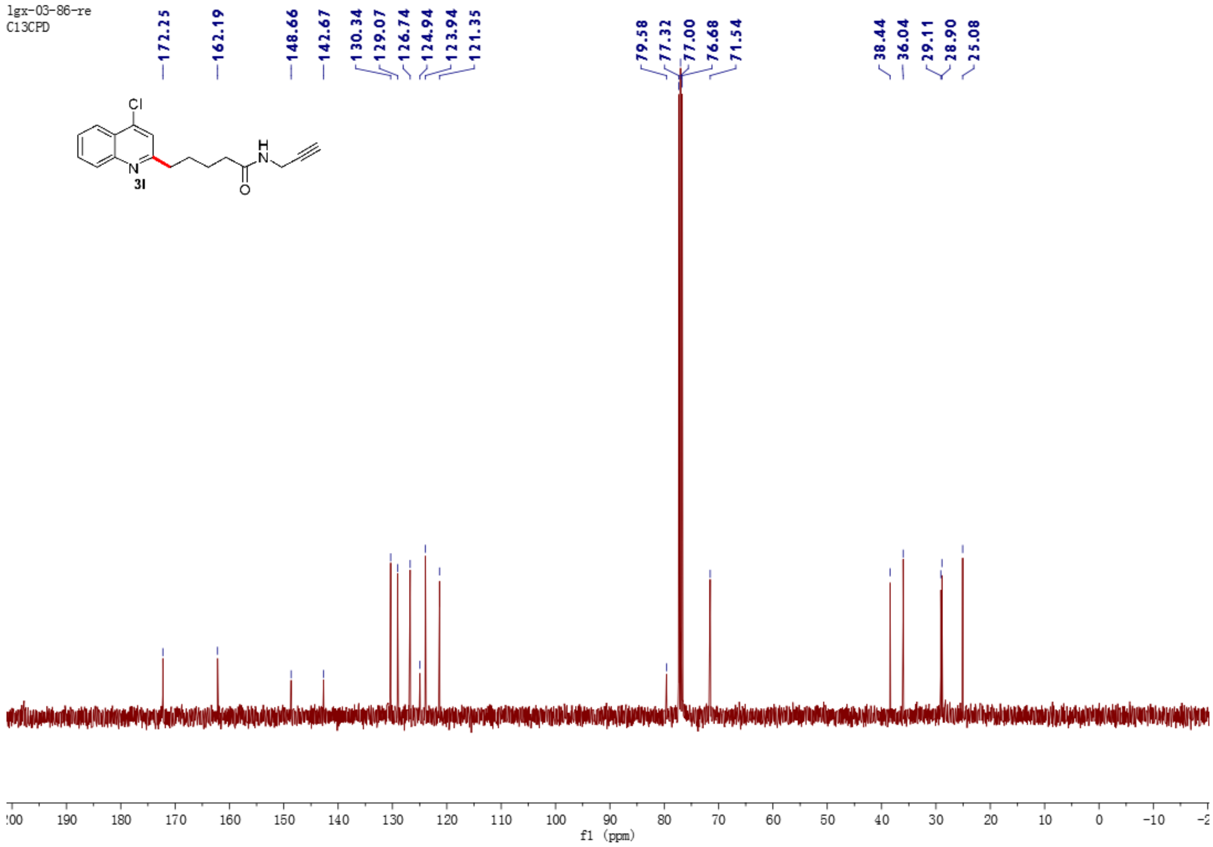
lgx-03-86-re
C13CPD
172.25
162.19
148.66
142.67
130.34
129.07
126.74
124.94
123.94
121.35
79.58
77.32
77.00
76.68
71.54
38.44
36.04
29.11
28.90
25.08
Cl
N
H
N
O
3l
f1 (ppm)

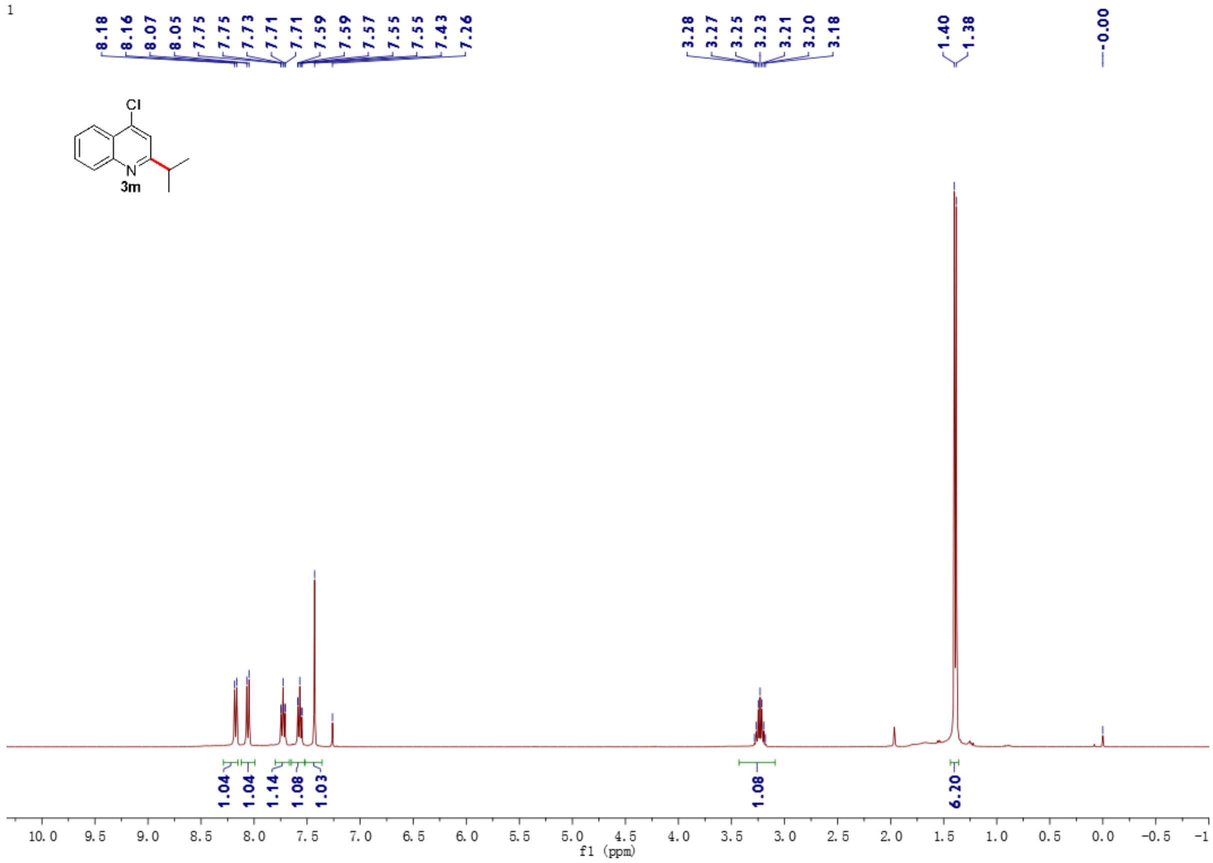
1
8.18
8.16
8.07
8.05
7.75
7.75
7.73
7.71
7.71
7.59
7.59
7.57
7.55
7.55
7.43
7.26
3.28
3.27
3.25
3.23
3.21
3.20
3.18
1.40
1.38
-0.00
Cl
N
3m
1.04
1.04
1.14
1.08
1.03
1.08
6.20
f1 (ppm)

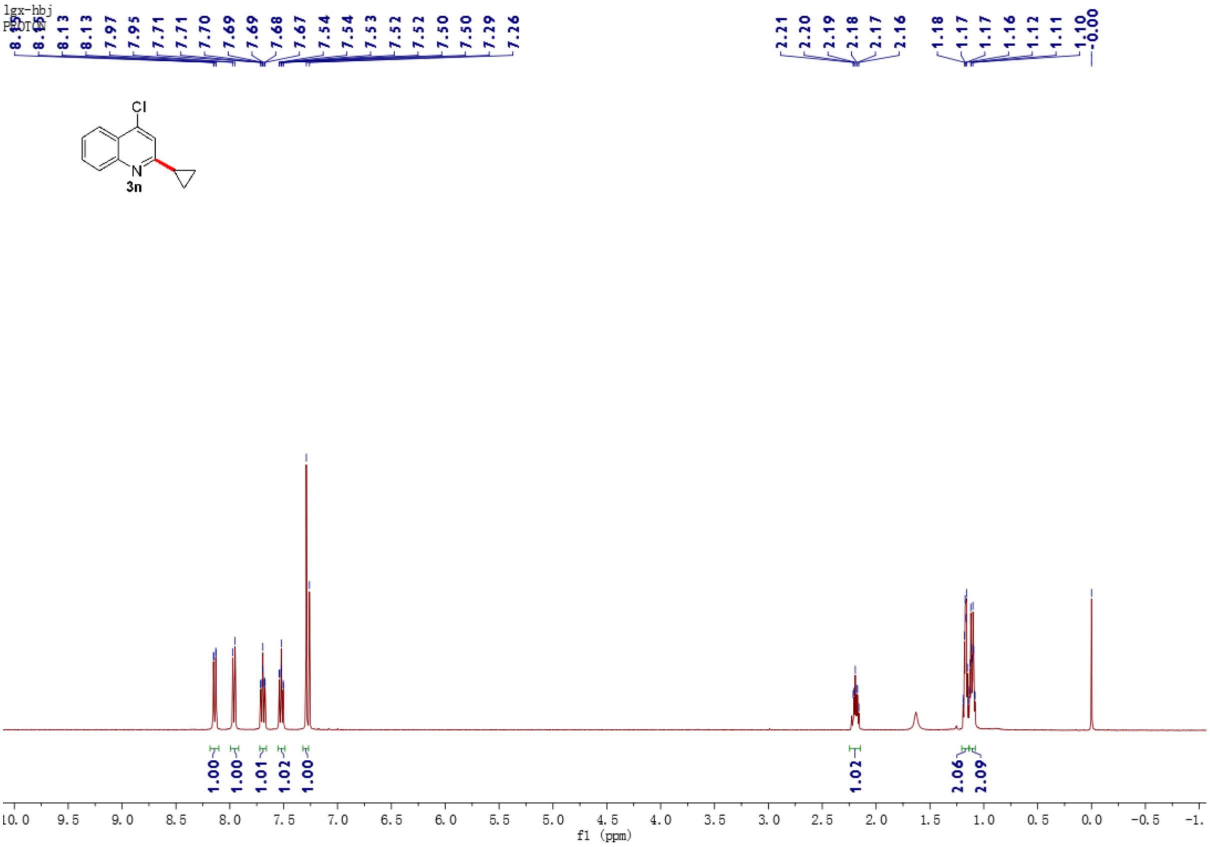
lgx-hbj
PROTON
8.13
8.13
7.97
7.95
7.71
7.71
7.70
7.69
7.69
7.68
7.67
7.54
7.54
7.53
7.52
7.52
7.50
7.50
7.29
7.26
2.21
2.20
2.19
2.18
2.17
2.16
1.18
1.17
1.17
1.16
1.12
1.11
1.10
0.00
Cl
N
3n
1.00
1.00
1.01
1.02
1.00
1.02
2.06
2.09
f1 (ppm)

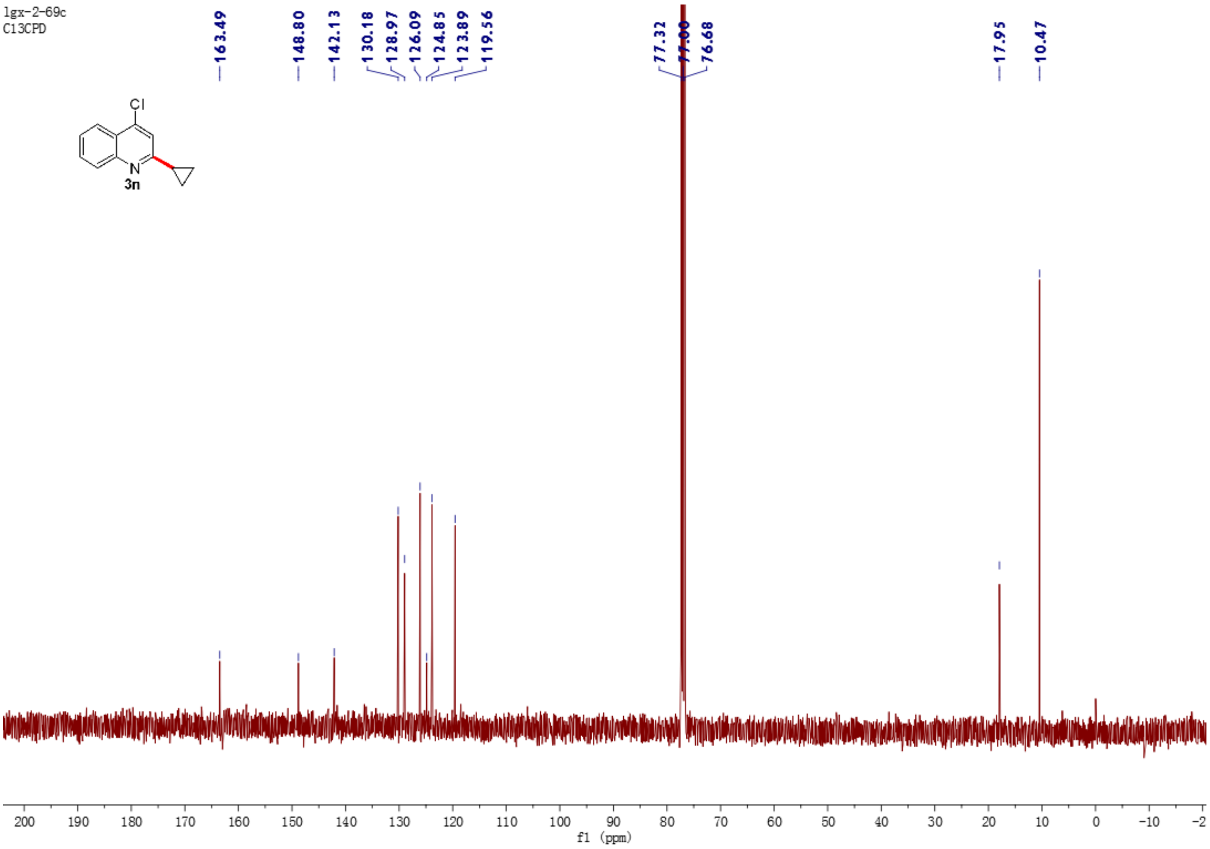
lgx-2-69c
C13CPD
163.49
148.80
142.13
130.18
128.97
126.09
124.85
123.89
119.56
77.32
77.00
76.68
17.95
10.47
Cl
N
3n
f1 (ppm)

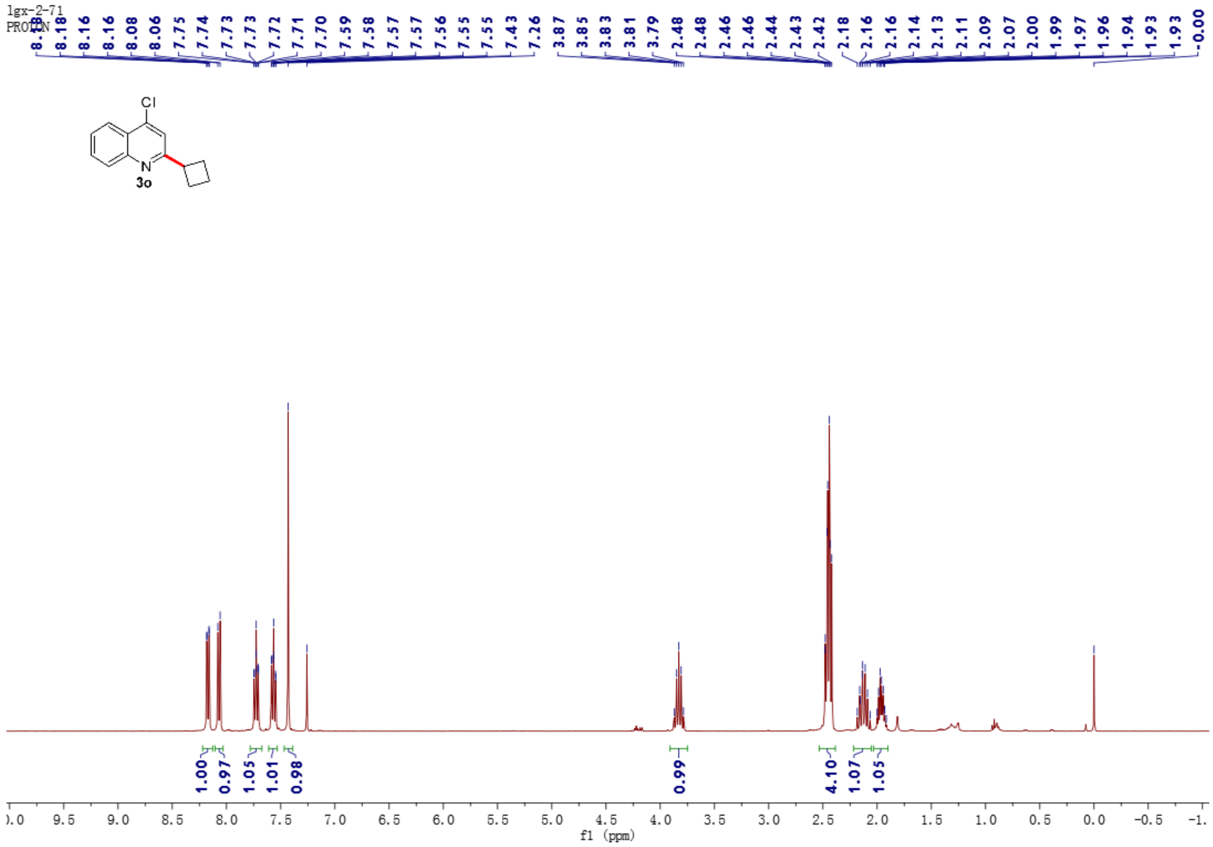
lgx-2-71
PROTON
3o
Cl
N
1.00
0.97
1.05
1.01
0.98
0.99
4.10
1.07
1.05
f1 (ppm)

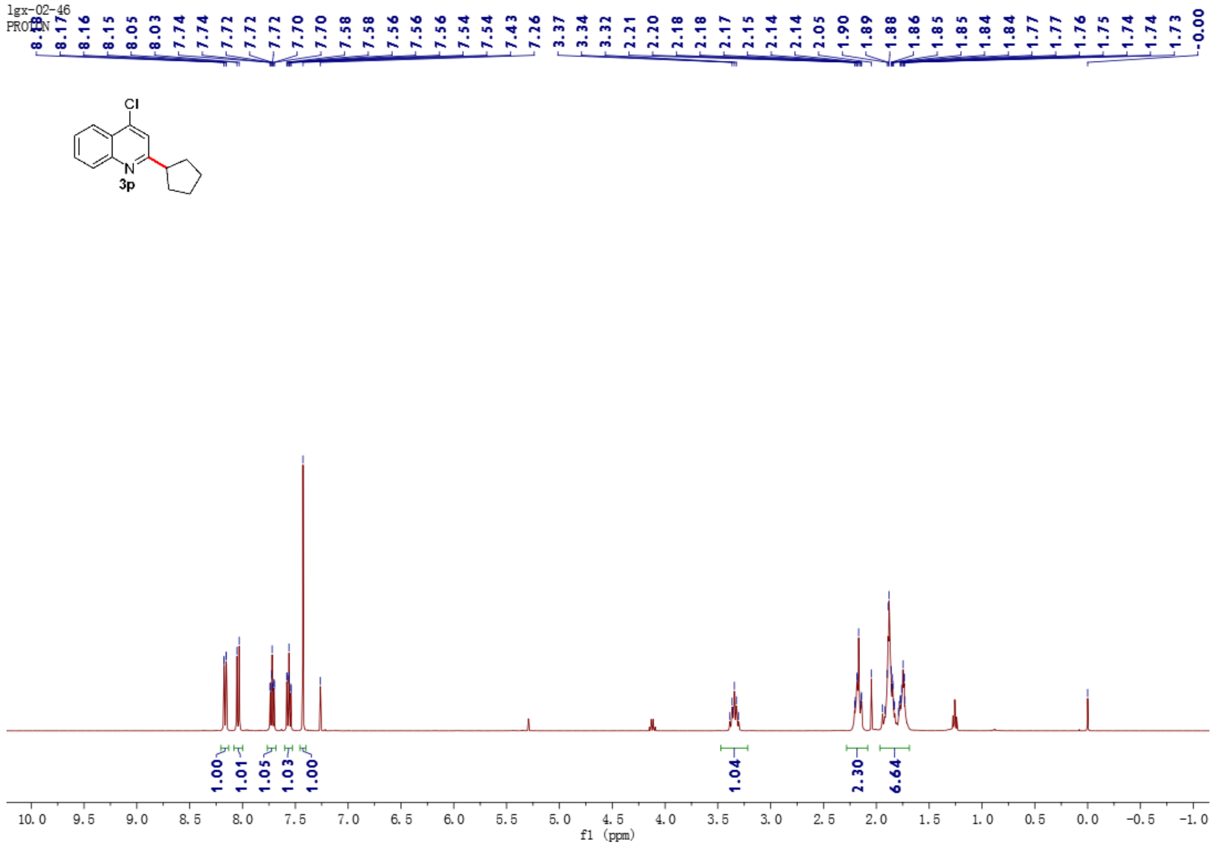
lgx-02-46
PROTON
3p
Cl
N
1.00
1.01
1.05
1.03
1.00
1.04
2.30
6.64
f1 (ppm)

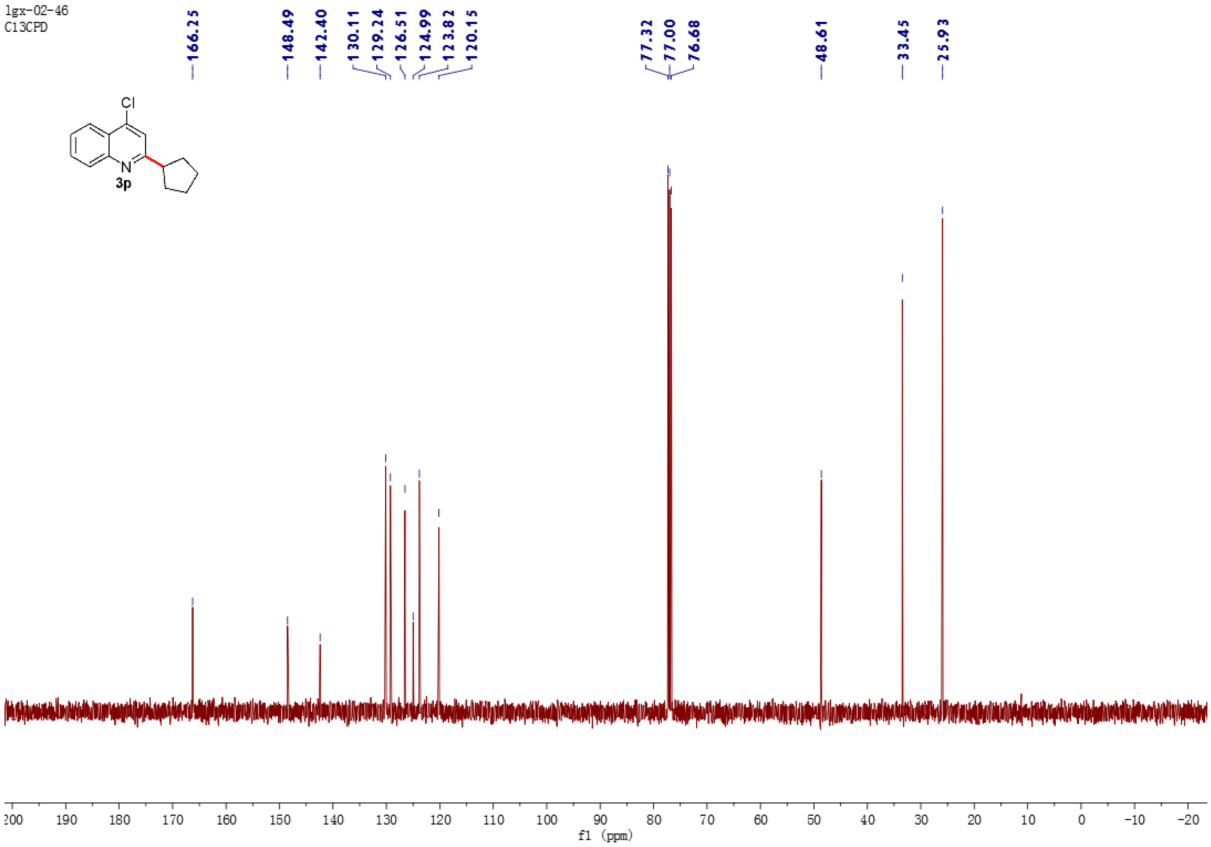
lgx-02-46
C13CPD
166.25
148.49
142.40
130.11
129.24
126.51
124.99
123.82
120.15
77.32
77.00
76.68
48.61
33.45
25.93
Cl
N
3p
f1 (ppm)

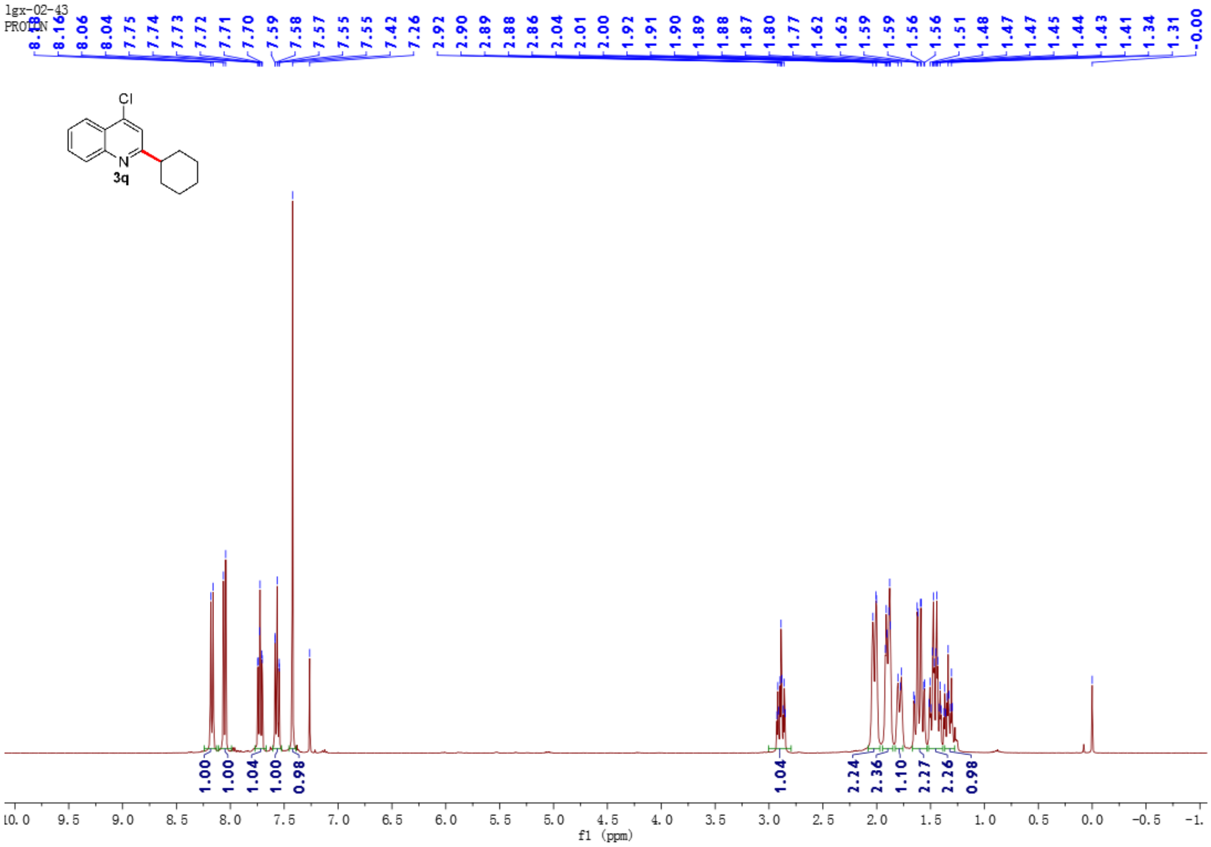
lgx-02-43
PROTON
8.16
8.06
8.04
7.75
7.74
7.73
7.72
7.71
7.70
7.59
7.58
7.57
7.55
7.55
7.42
7.26
2.92
2.90
2.89
2.88
2.86
2.04
2.01
2.00
1.92
1.91
1.90
1.89
1.88
1.87
1.80
1.77
1.62
1.62
1.59
1.59
1.56
1.56
1.51
1.48
1.47
1.47
1.45
1.44
1.43
1.41
1.34
1.31
-0.00
Cl
N
3q
1.00
1.00
1.04
1.00
0.98
1.04
2.24
2.36
1.10
2.27
2.26
0.98
f1 (ppm)

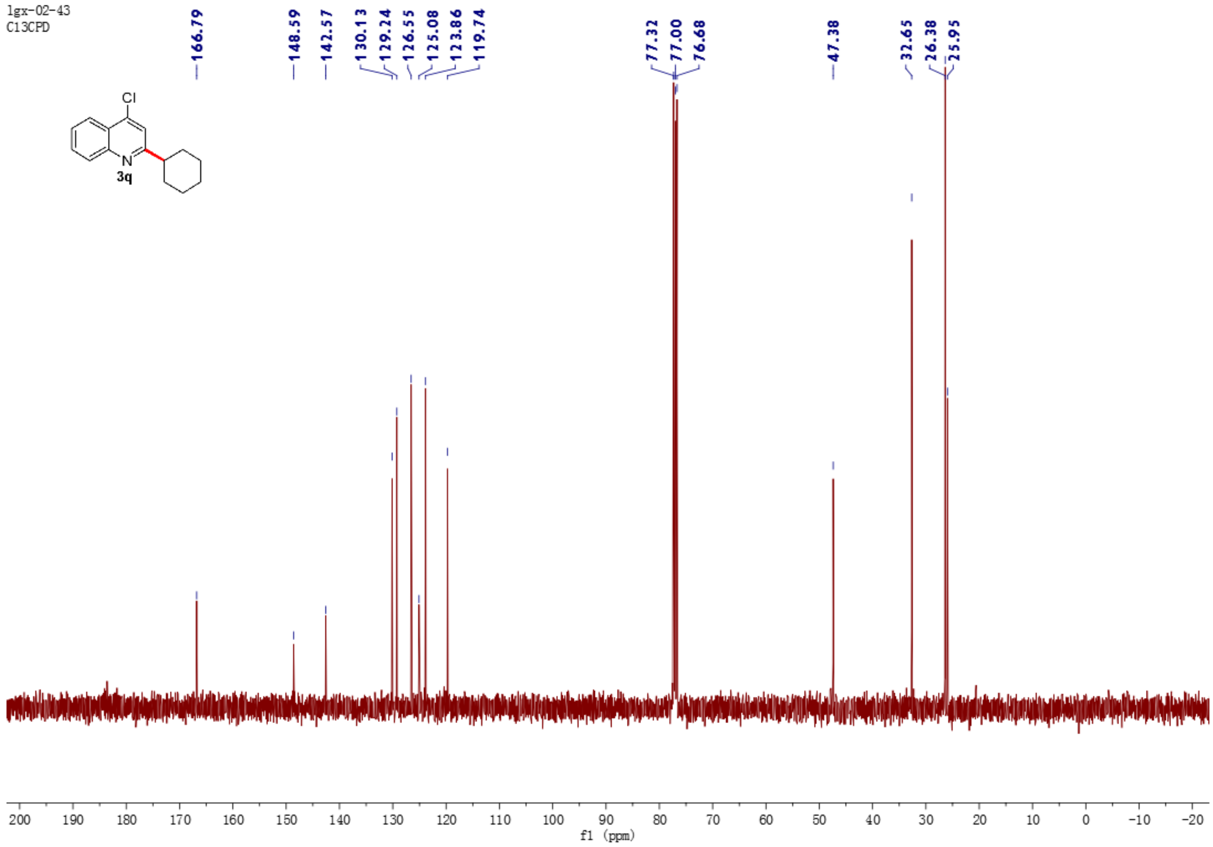
lgx-02-43
C13CPD
166.79
148.59
142.57
130.13
129.24
126.55
125.08
123.86
119.74
77.32
77.00
76.68
47.38
32.65
26.38
25.95
Cl
N
3q
f1 (ppm)

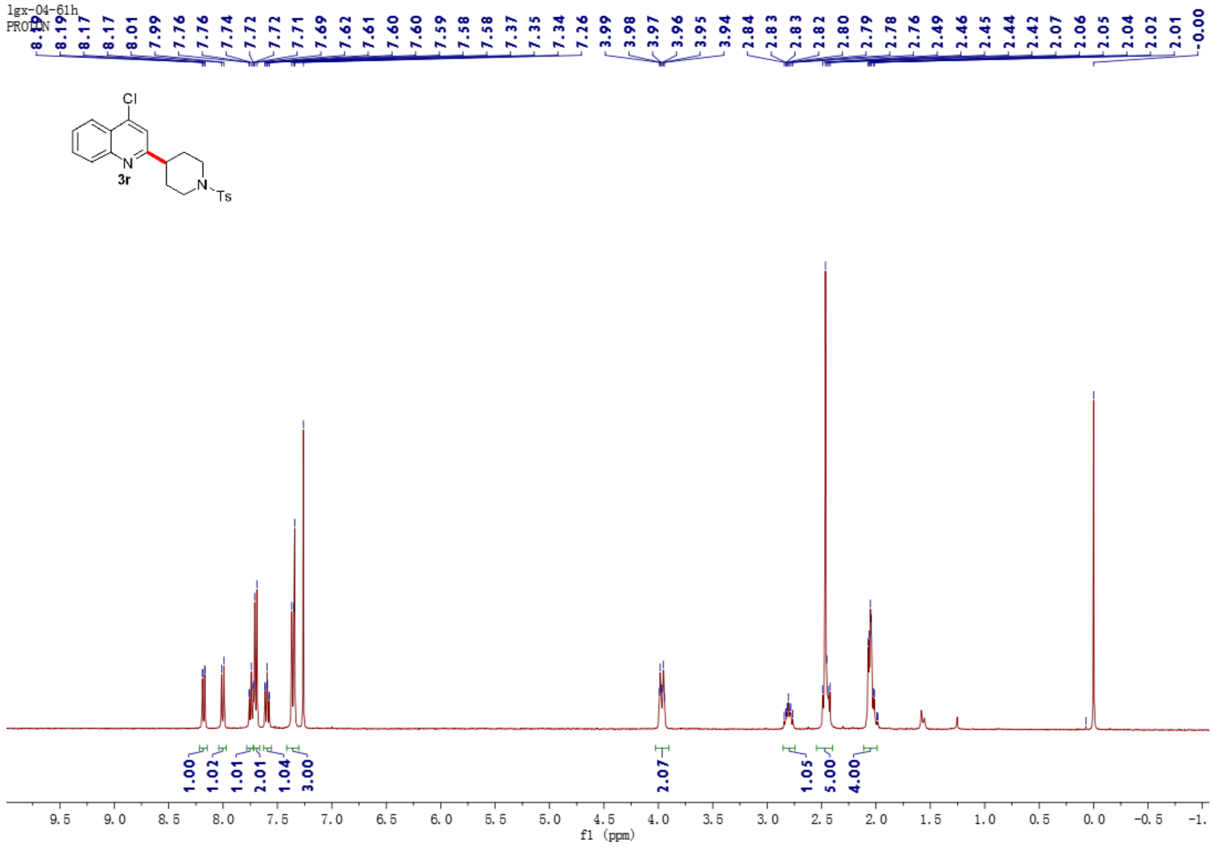
lgx-04-61h
PROTON
8.19
8.17
8.17
8.01
7.99
7.76
7.76
7.74
7.72
7.72
7.71
7.69
7.62
7.61
7.60
7.60
7.59
7.58
7.58
7.37
7.35
7.34
7.26
3.99
3.98
3.97
3.96
3.95
3.94
2.84
2.83
2.83
2.82
2.80
2.79
2.78
2.76
2.49
2.46
2.45
2.44
2.42
2.07
2.06
2.05
2.04
2.02
2.01
-0.00
Cl
N
N
Ts
3r
1.00
1.02
1.01
2.01
1.04
3.00
2.07
1.05
5.00
4.00
f1 (ppm)

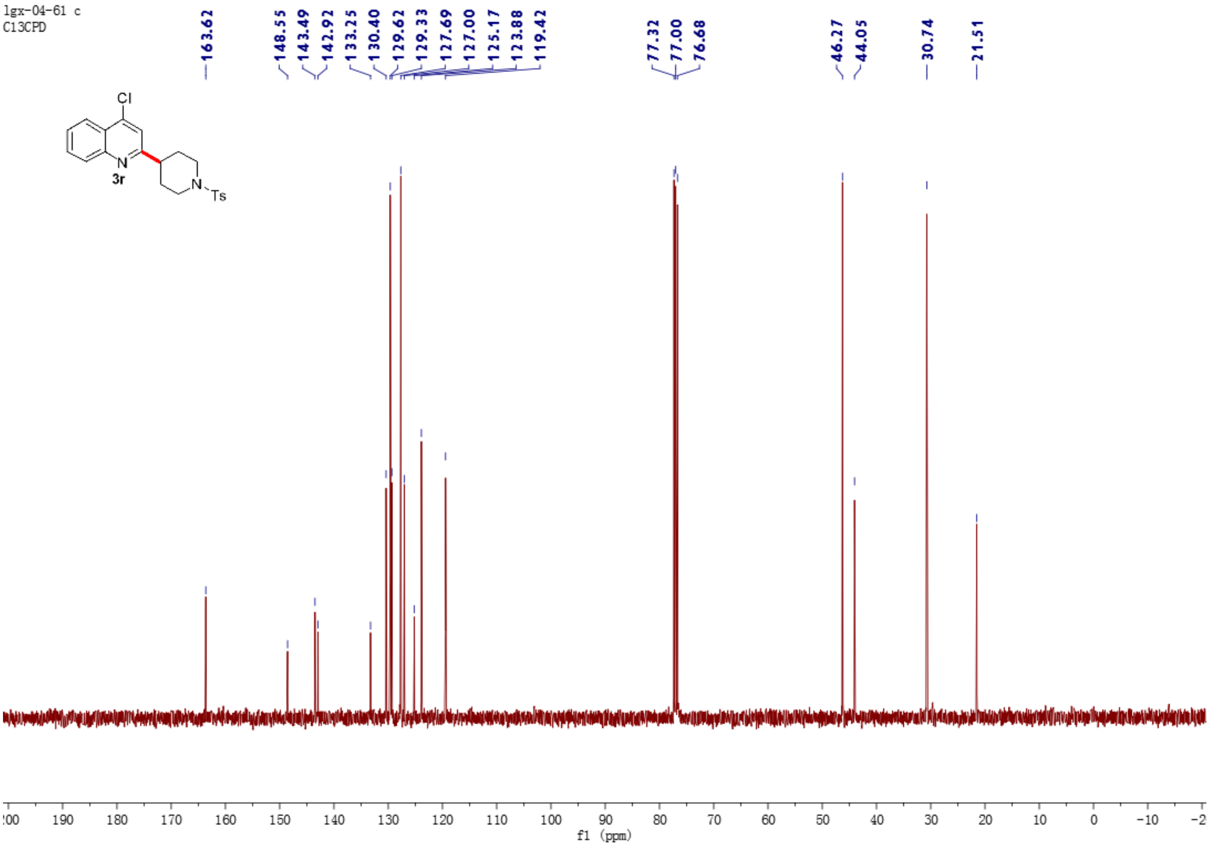

lgx-04-61 c
C13CPD
163.62
148.55
143.49
142.92
133.25
130.40
129.62
129.33
127.69
127.00
125.17
123.88
119.42
77.32
77.00
76.68
46.27
44.05
30.74
21.51
Cl
N
3r
N
Ts
f1 (ppm)

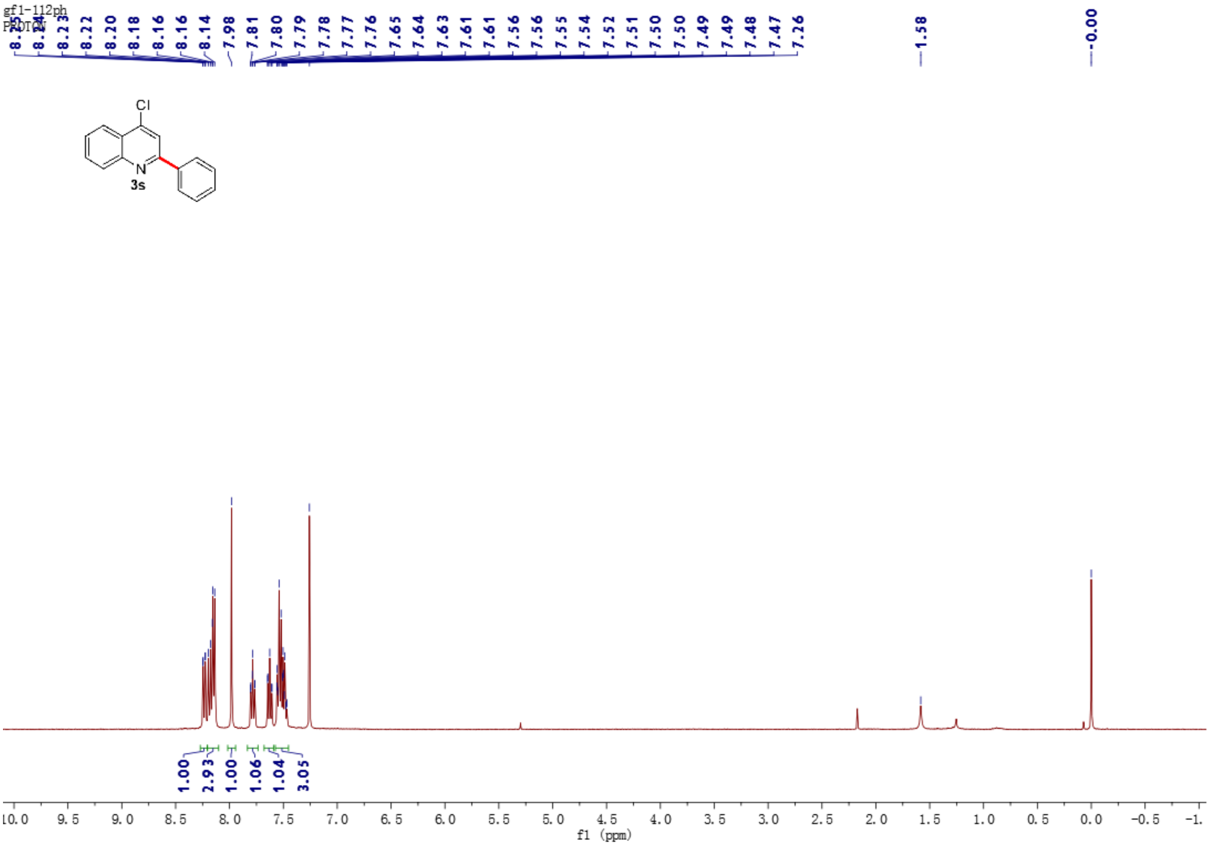

gf1-112ph
7.98
7.81
7.80
7.79
7.78
7.77
7.76
7.65
7.64
7.63
7.61
7.61
7.56
7.56
7.55
7.54
7.52
7.51
7.50
7.50
7.49
7.49
7.48
7.47
7.26
1.58
-0.00
Cl
N
3s
1.00
2.93
1.00
1.06
1.04
3.05
f1 (ppm)

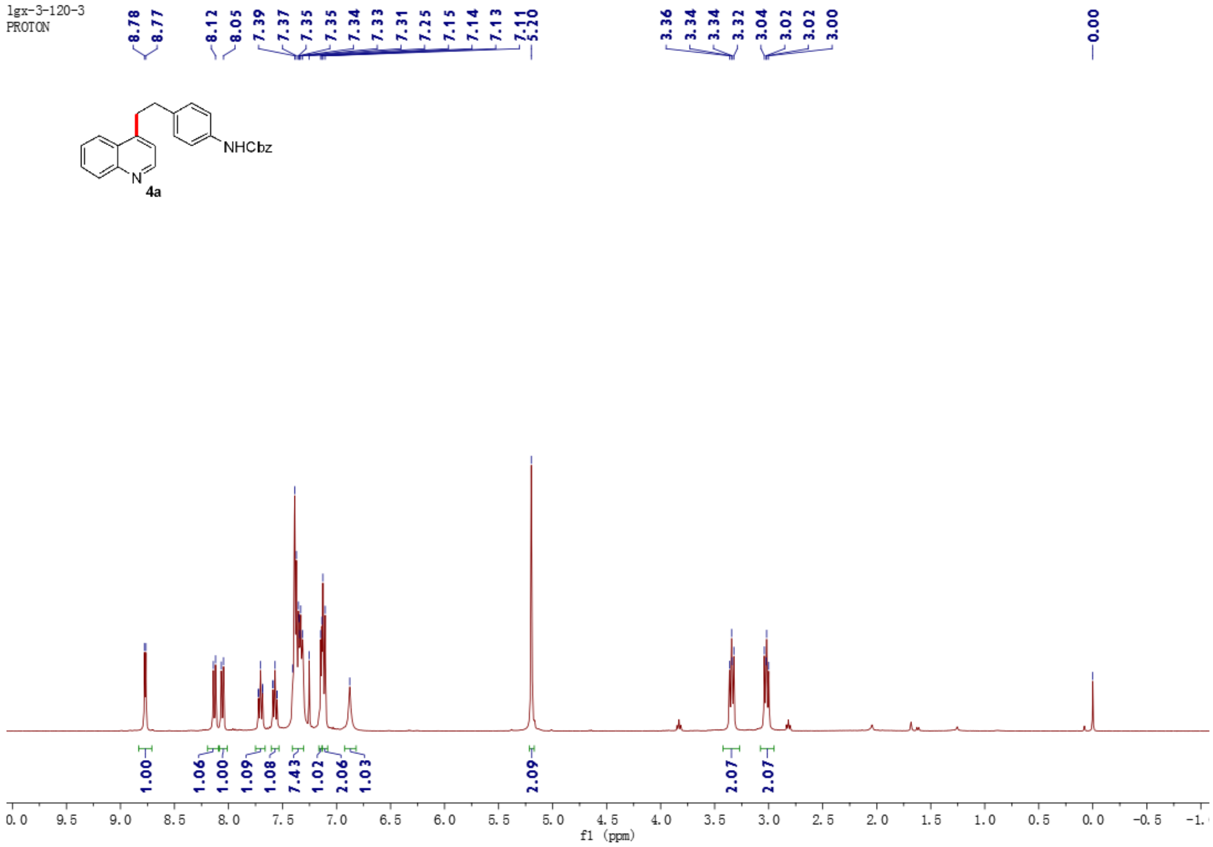

lgx-3-120-3
PROTON
8.78
8.77
8.12
8.05
7.39
7.37
7.35
7.35
7.34
7.33
7.31
7.25
7.15
7.14
7.13
7.11
5.20
3.36
3.34
3.34
3.32
3.04
3.02
3.02
3.00
0.00
NHCbz
N
4a
1.00
1.06
1.00
1.09
1.08
7.43
1.02
2.06
1.03
2.09
2.07
2.07
f1 (ppm)

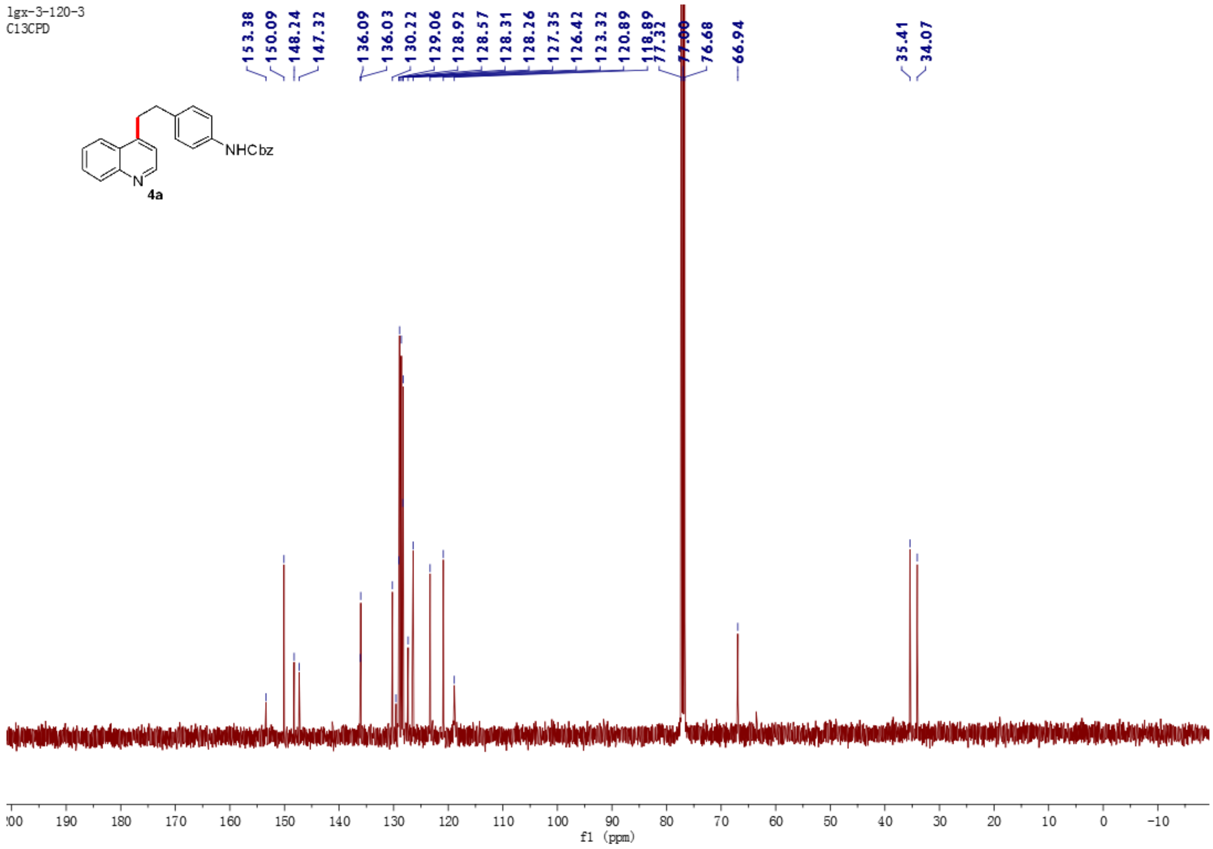

lgx-3-120-3
C13CPD
153.38
150.09
148.24
147.32
136.09
136.03
130.22
129.06
128.92
128.57
128.31
128.26
127.35
126.42
123.32
120.89
118.89
77.32
77.00
76.68
66.94
35.41
34.07
NHCbz
N
4a
f1 (ppm)

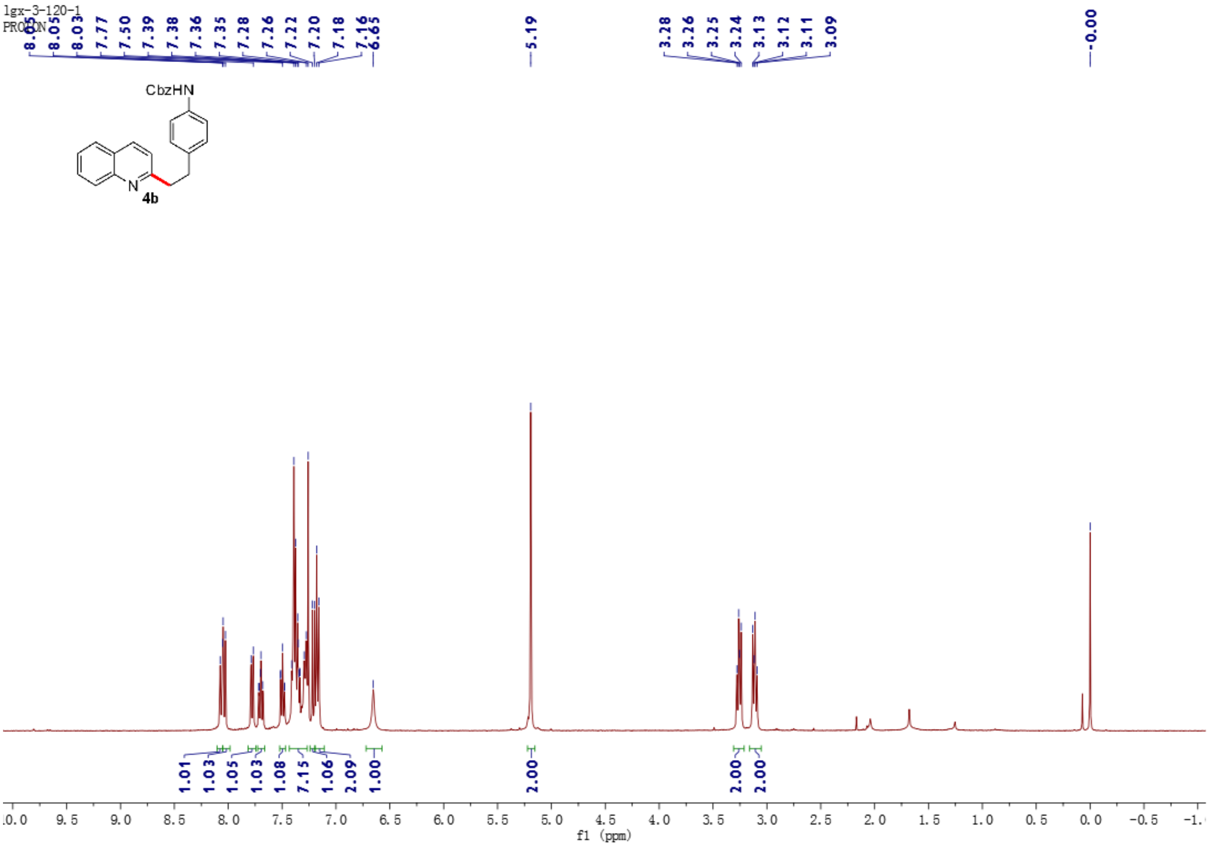
lgx-3-120-1
PROTON
CbzHN
N
4b
f1 (ppm)

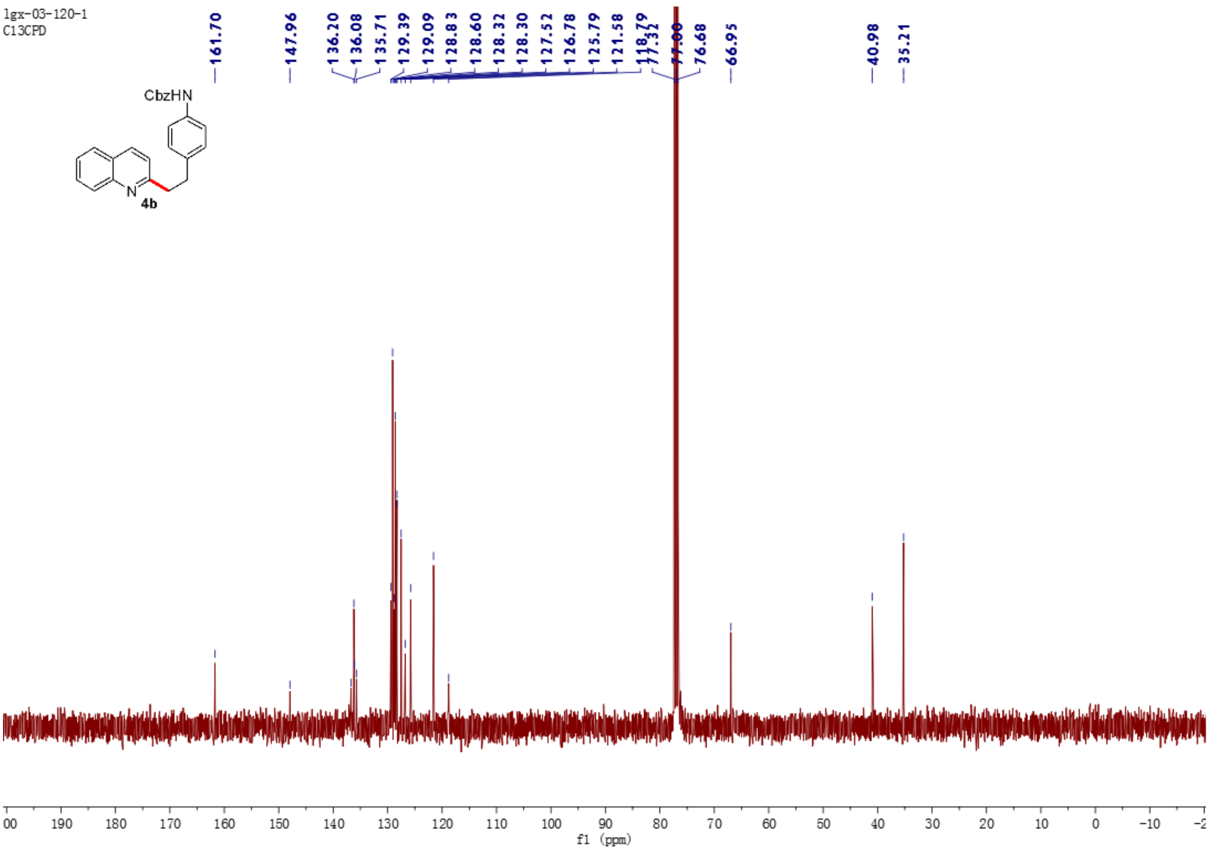
lgx-03-120-1
C13CPD
CbzHN
N
4b
f1 (ppm)

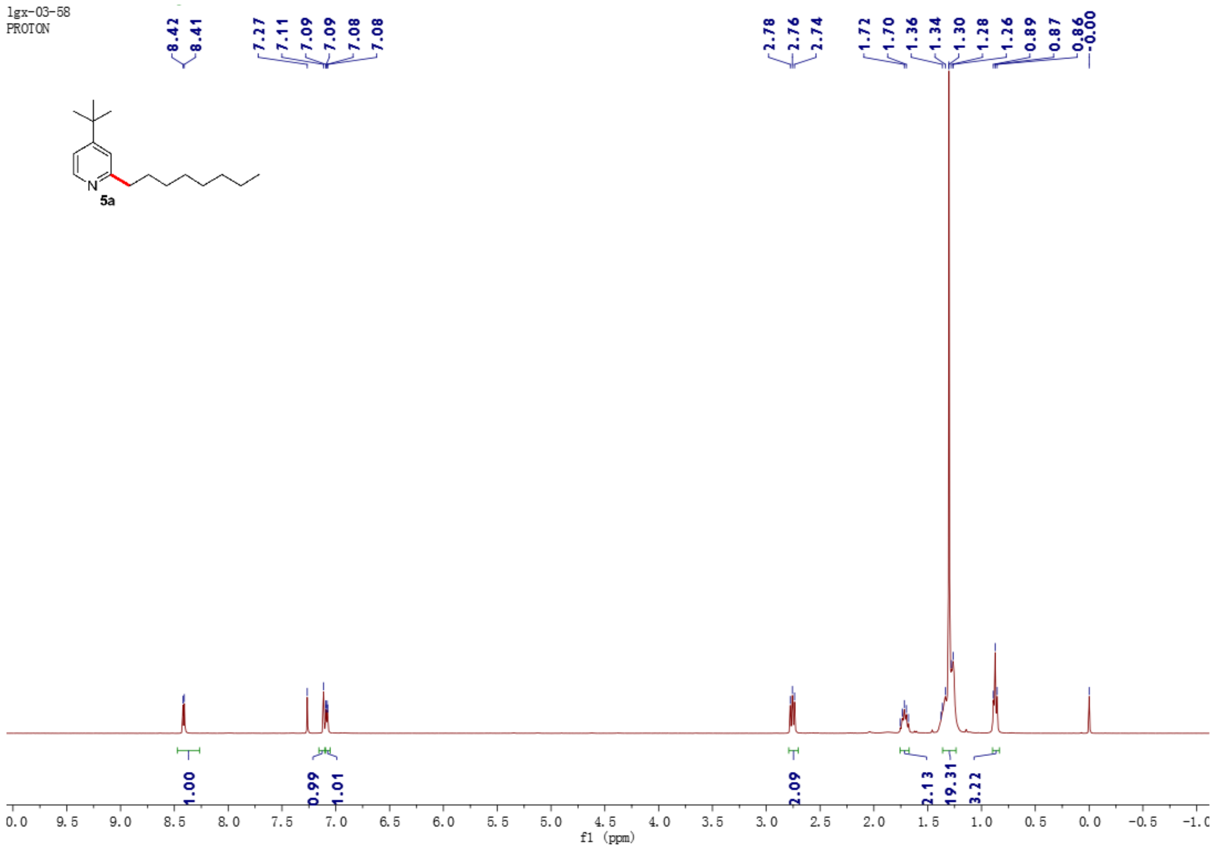
lgx-03-58
PROTON
8.42
8.41
7.27
7.11
7.09
7.09
7.08
7.08
2.78
2.76
2.74
1.72
1.70
1.36
1.34
1.30
1.28
1.26
0.89
0.87
0.86
-0.00
5a
N
1.00
0.99
1.01
2.09
2.13
19.31
3.22
f1 (ppm)

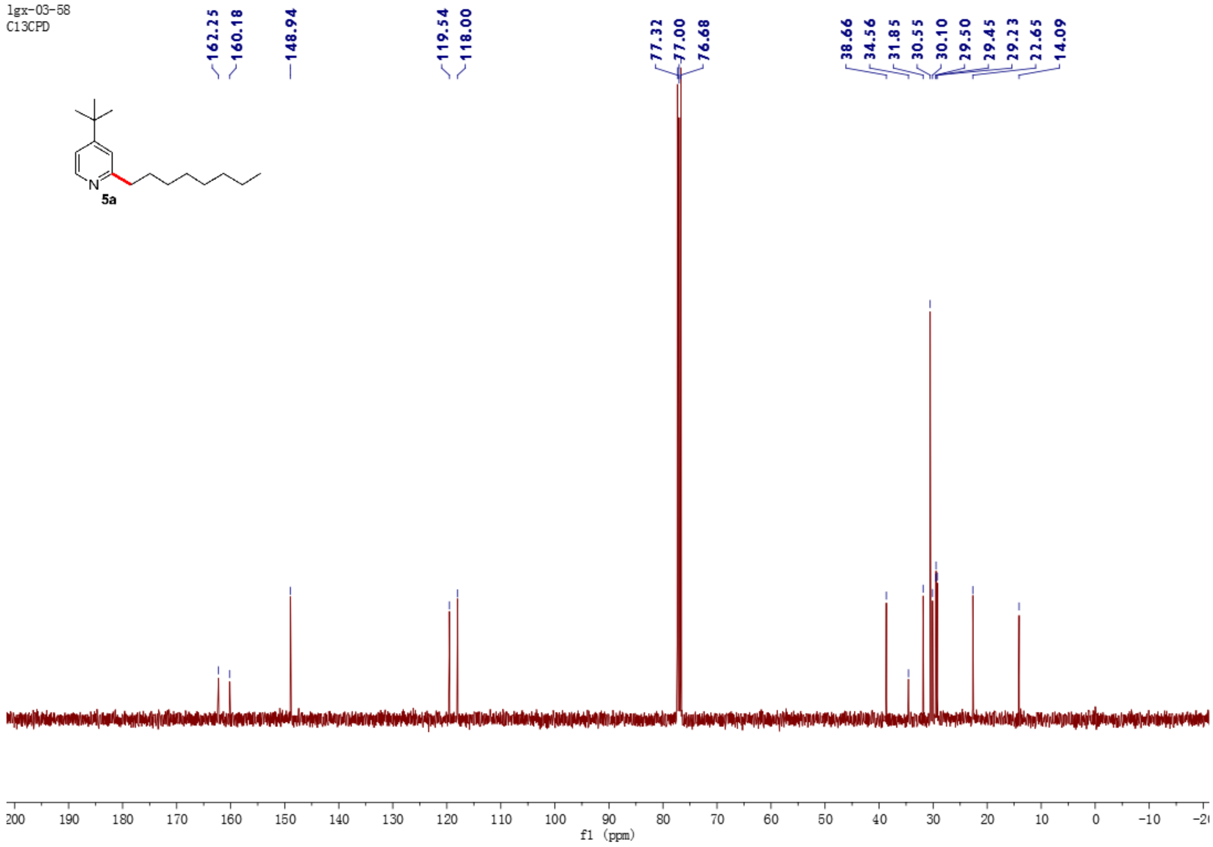
lgx-03-58
C13CPD
162.25
160.18
148.94
119.54
118.00
77.32
77.00
76.68
38.66
34.56
31.85
30.55
30.10
29.50
29.45
29.23
22.65
14.09
5a
N
f1 (ppm)

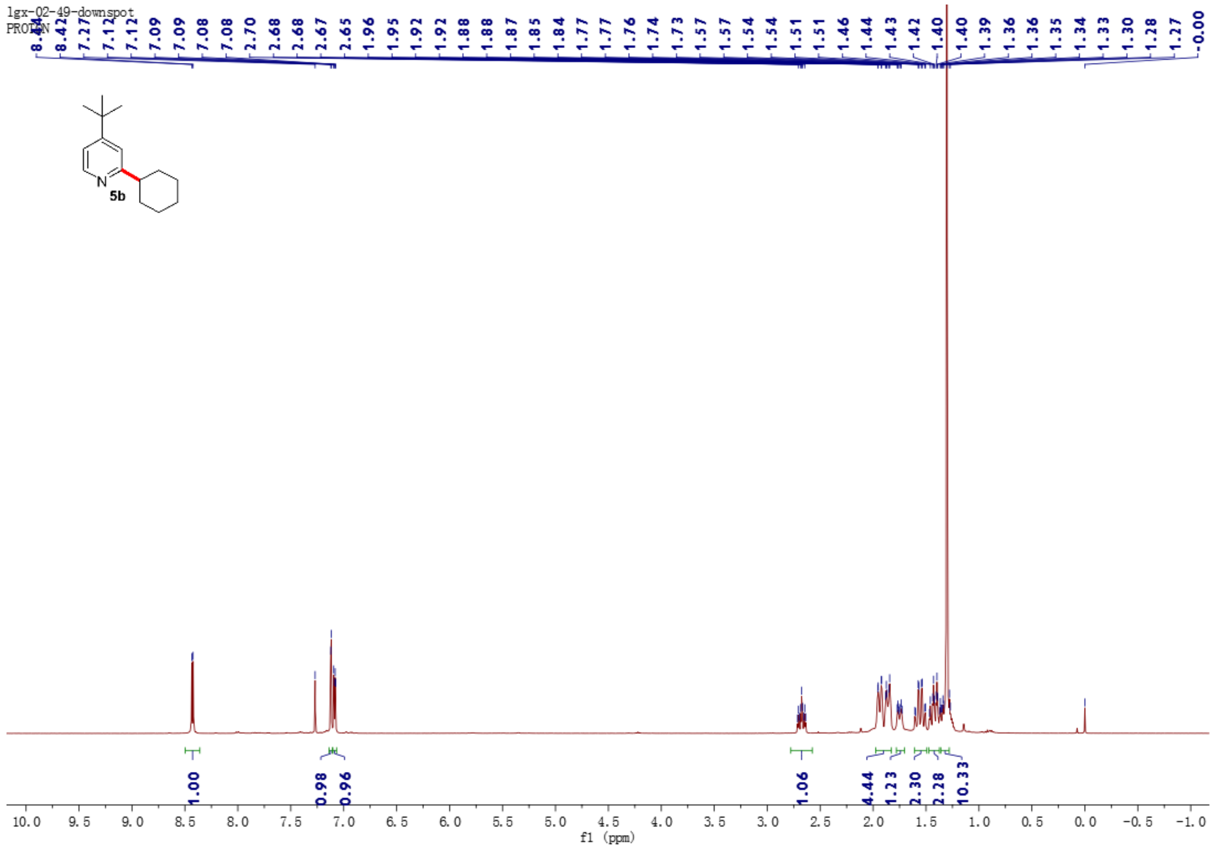
lgx-02-49-downspot
PROTON
5b
1.00
0.98
0.96
1.06
4.44
1.23
2.30
2.28
10.33
f1 (ppm)

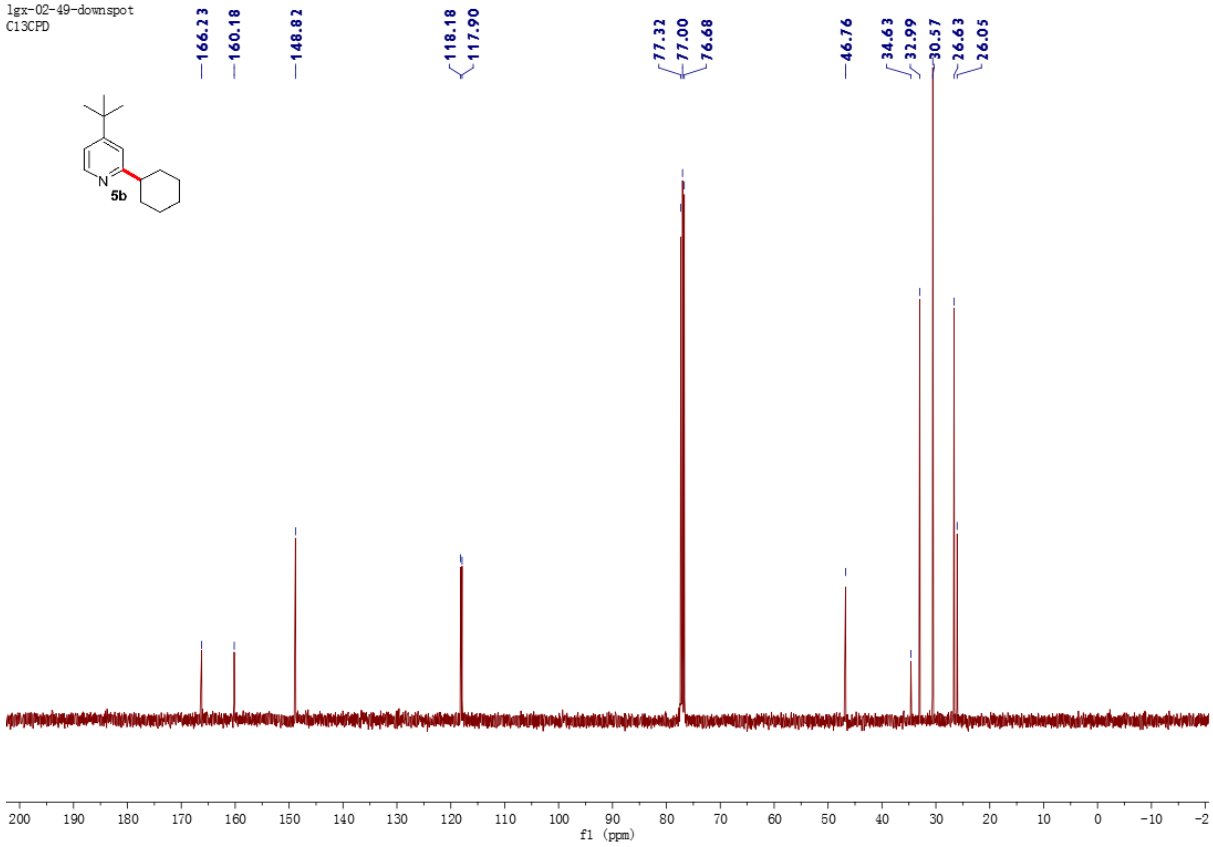
lgx-02-49-downspot
C13CPD
166.23
160.18
148.82
118.18
117.90
77.32
77.00
76.68
46.76
34.63
32.99
30.57
26.63
26.05
5b
f1 (ppm)

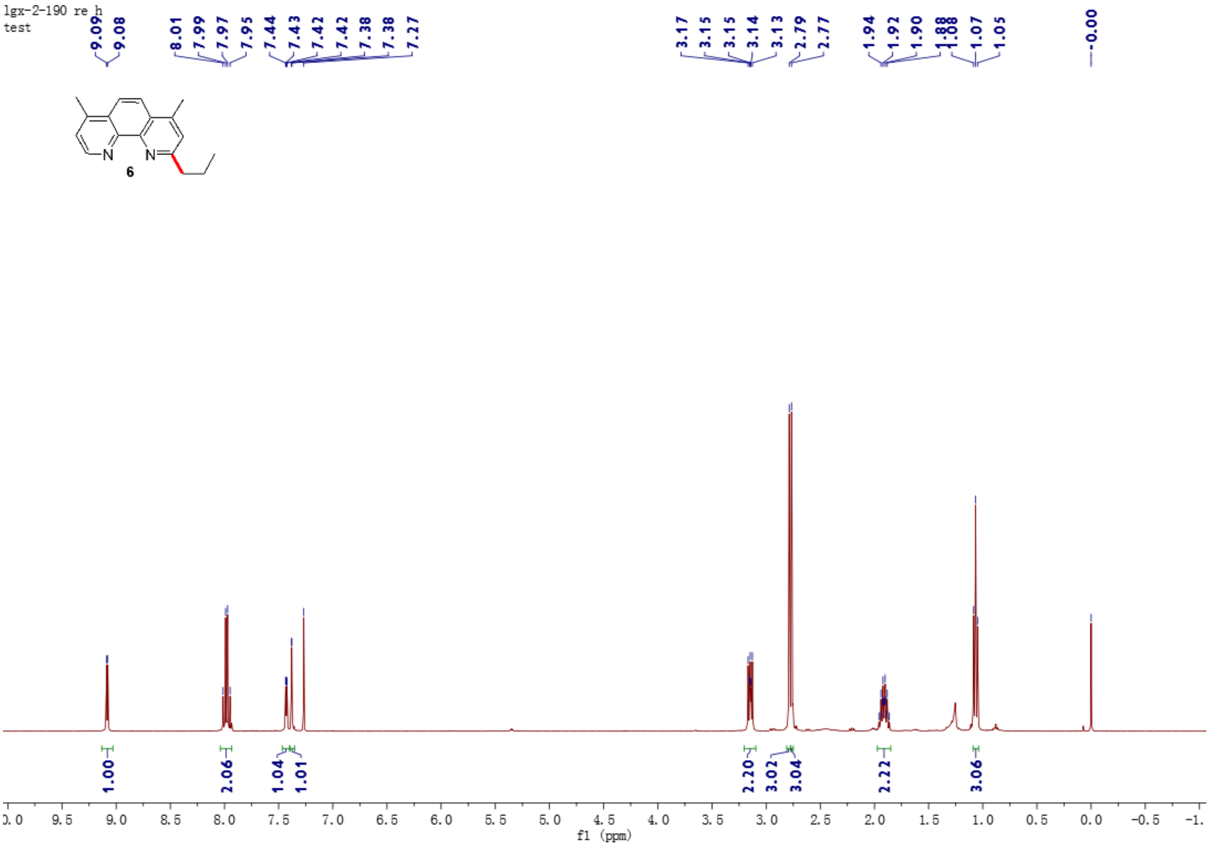

lgx-2-190 re h
test
9.09
9.08
8.01
7.99
7.97
7.95
7.44
7.43
7.42
7.42
7.38
7.38
7.27
3.17
3.15
3.15
3.14
3.13
2.79
2.77
1.94
1.92
1.90
1.88
1.88
1.07
1.05
0.00
N
N
6
1.00
2.06
1.04
1.01
2.20
3.02
3.04
2.22
3.06
f1 (ppm)

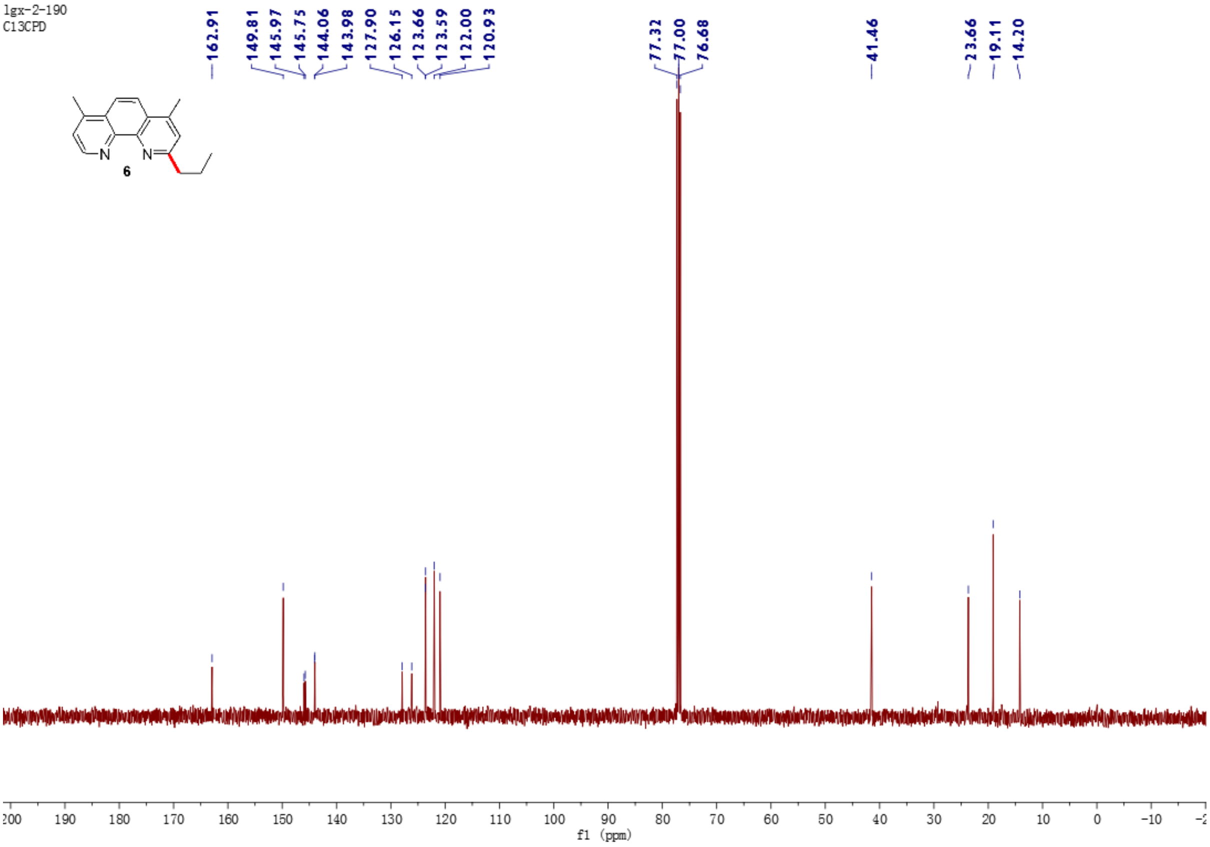

lgx-2-190
C13CPD
162.91
149.81
145.97
145.75
144.06
143.98
127.90
126.15
123.66
123.59
122.00
120.93
77.32
77.00
76.68
41.46
23.66
19.11
14.20
N
N
6
f1 (ppm)

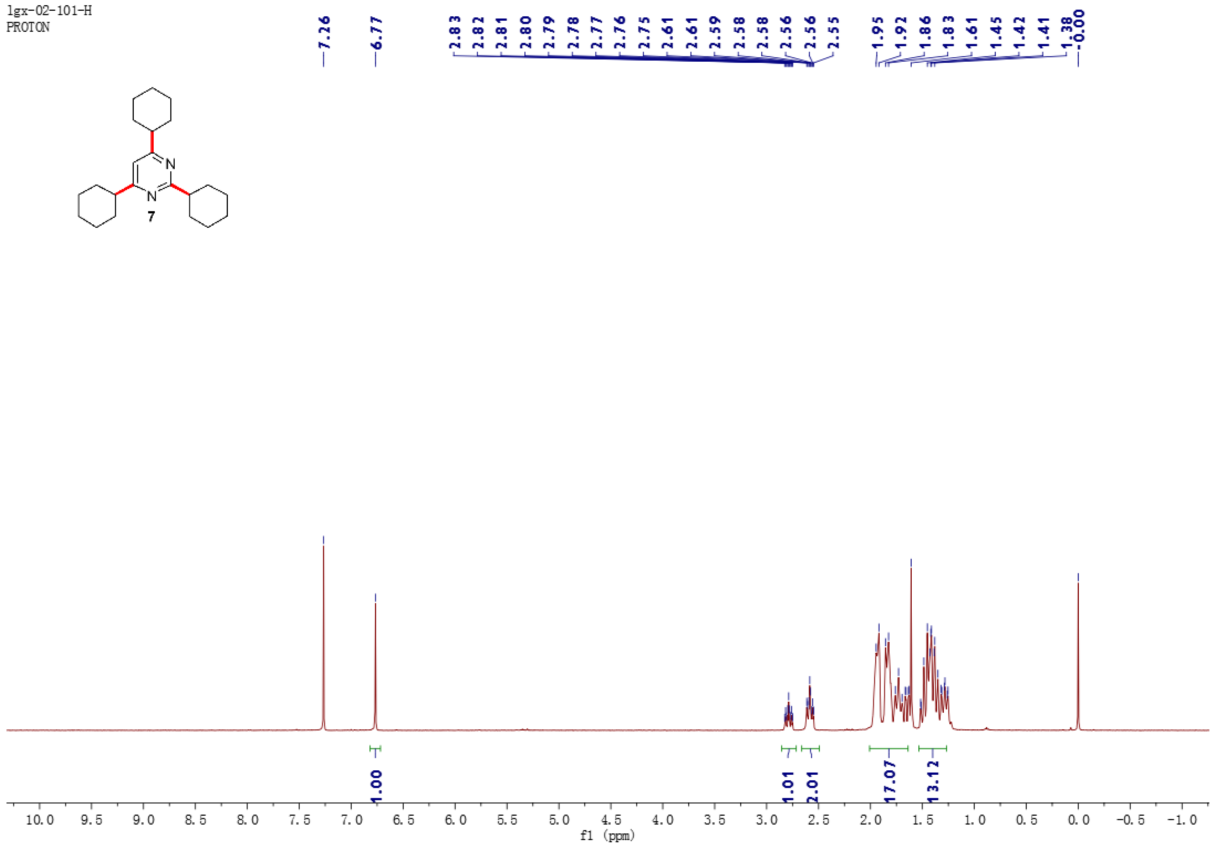
lgx-02-101-H
PROTON
7.26
6.77
2.83 2.82 2.81 2.80 2.79 2.78 2.77 2.76 2.75 2.61 2.61 2.59 2.58 2.58 2.56 2.56 2.55
1.95 1.92 1.86 1.83 1.61 1.45 1.42 1.41 1.38 0.00
N
N
7
1.00
1.01
2.01
17.07
13.12
10.0 9.5 9.0 8.5 8.0 7.5 7.0 6.5 6.0 5.5 5.0 4.5 4.0 3.5 3.0 2.5 2.0 1.5 1.0 0.5 0.0 -0.5 -1.0
f1 (ppm)

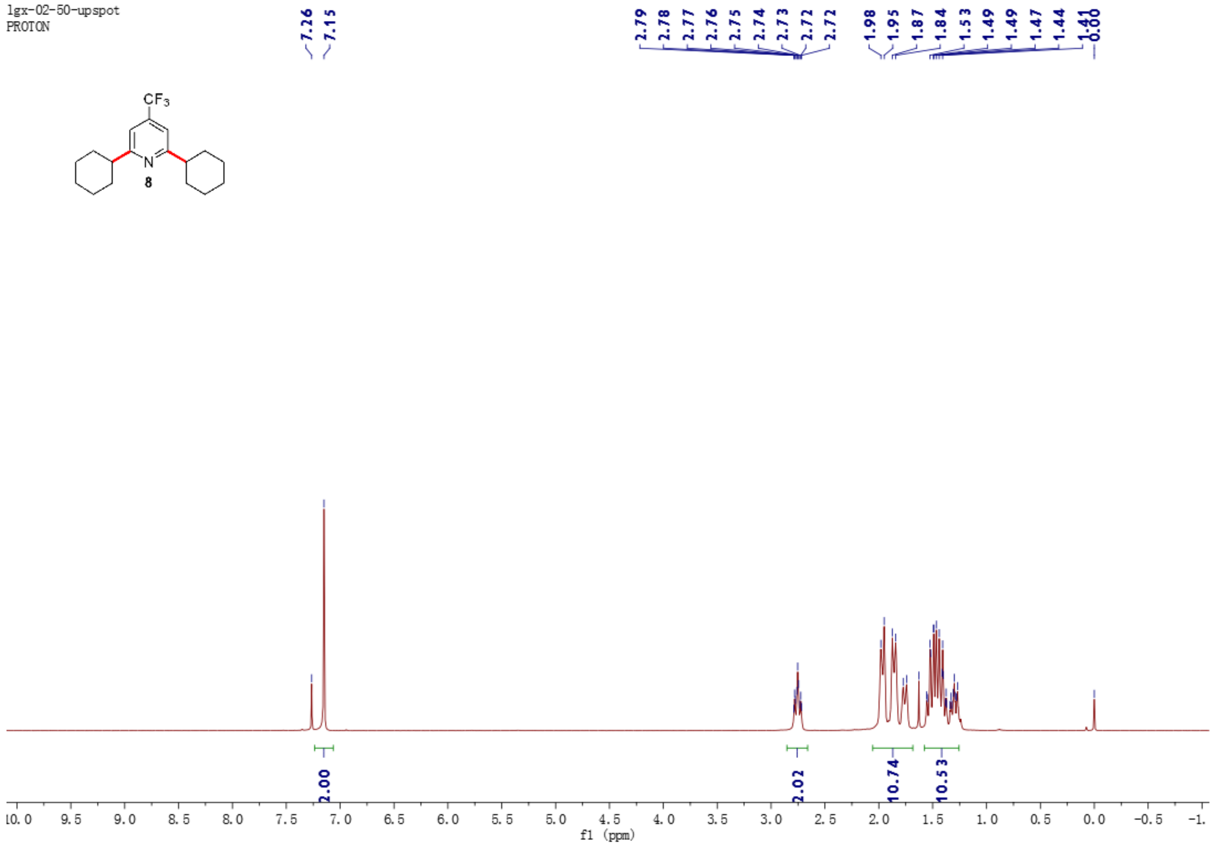
lgx-02-50-upspot
PROTON
7.26
7.15
2.79 2.78 2.77 2.76 2.75 2.74 2.73 2.72 2.72
1.98 1.95 1.87 1.84 1.53 1.49 1.49 1.47 1.44 1.41 0.00
CF3
N
8
2.00
2.02
10.74
10.53
10.0 9.5 9.0 8.5 8.0 7.5 7.0 6.5 6.0 5.5 5.0 4.5 4.0 3.5 3.0 2.5 2.0 1.5 1.0 0.5 0.0 -0.5 -1.
f1 (ppm)

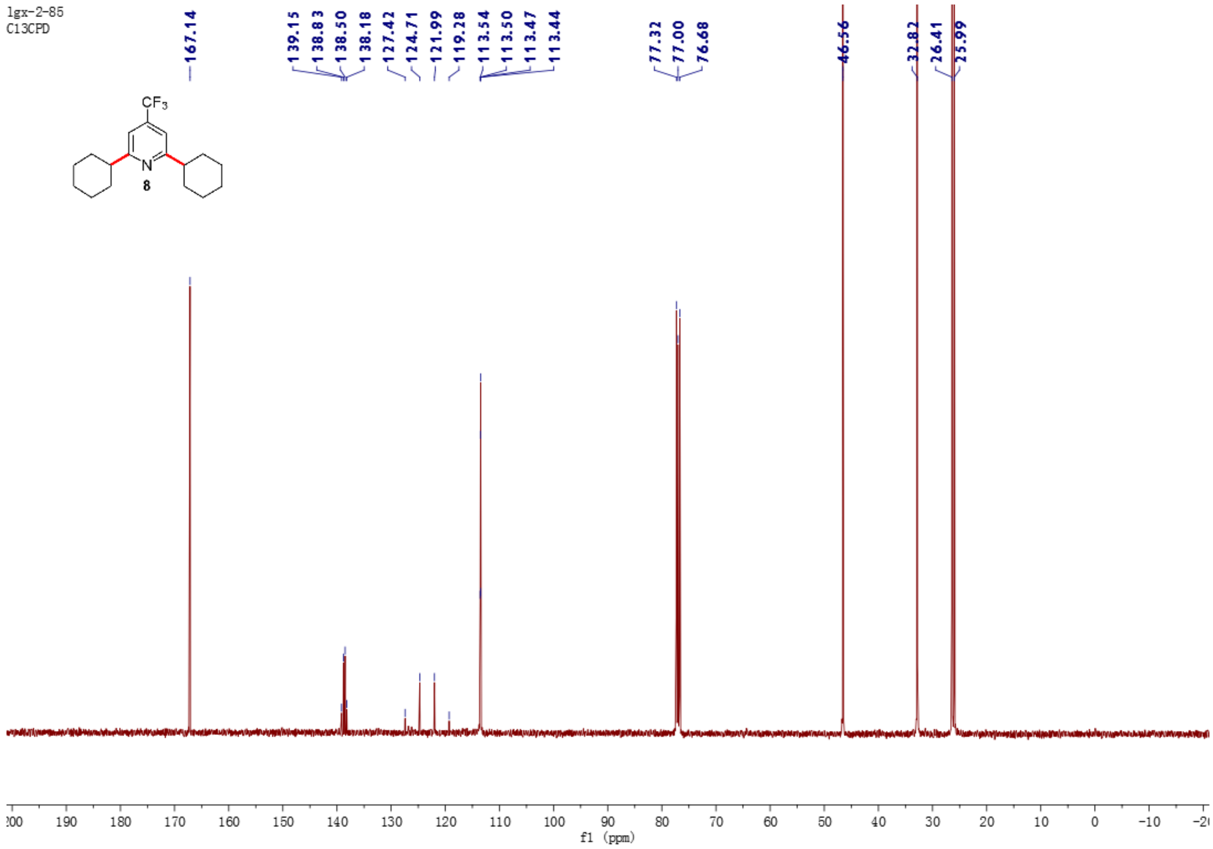
lgx-2-85
C13CPD
167.14
139.15
138.83
138.50
138.18
127.42
124.71
121.99
119.28
113.54
113.50
113.47
113.44
77.32
77.00
76.68
46.56
32.82
26.41
25.99
CF3
N
8
f1 (ppm)

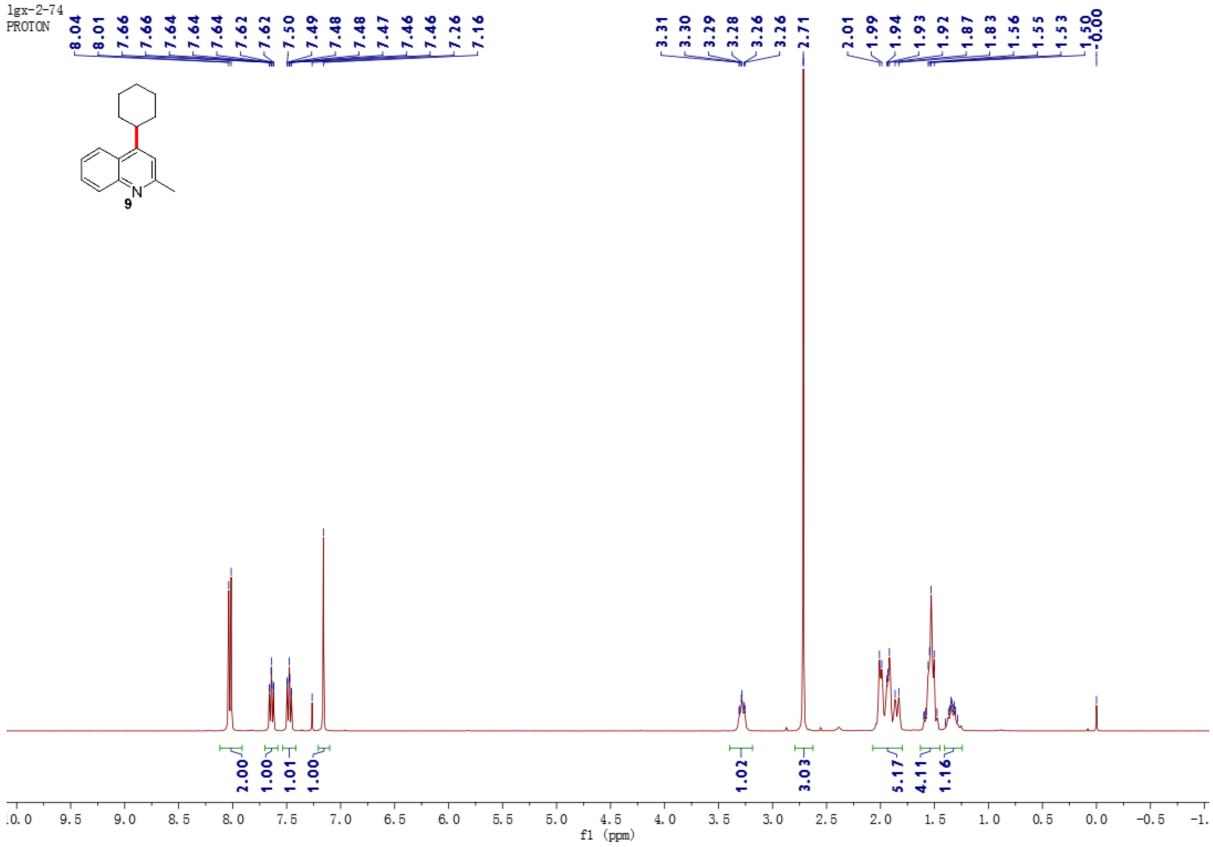
lgx-2-74
PROTON
8.04
8.01
7.66
7.66
7.64
7.64
7.62
7.62
7.50
7.49
7.48
7.48
7.47
7.46
7.46
7.26
7.16
3.31
3.30
3.29
3.28
3.26
3.26
2.71
2.01
1.99
1.94
1.93
1.92
1.87
1.83
1.56
1.55
1.53
1.50
0.00
N
9
2.00
1.00
1.01
1.00
1.02
3.03
5.17
4.11
1.16
f1 (ppm)

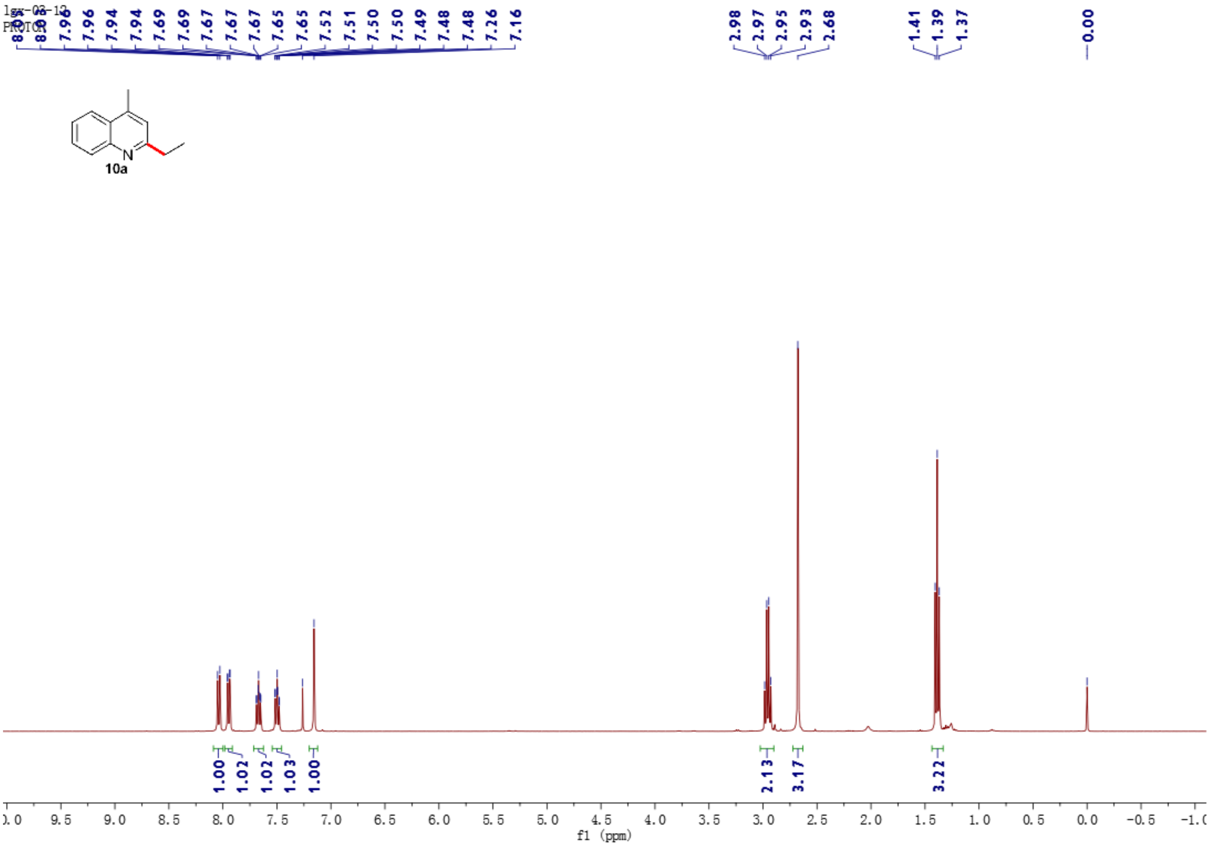

PROTON
7.96
7.94
7.94
7.69
7.69
7.67
7.67
7.67
7.65
7.65
7.52
7.51
7.50
7.50
7.49
7.48
7.48
7.26
7.16
2.98
2.97
2.95
2.93
2.68
1.41
1.39
1.37
0.00
N
10a
1.00
1.02
1.02
1.03
1.00
2.13
3.17
3.22
f1 (ppm)

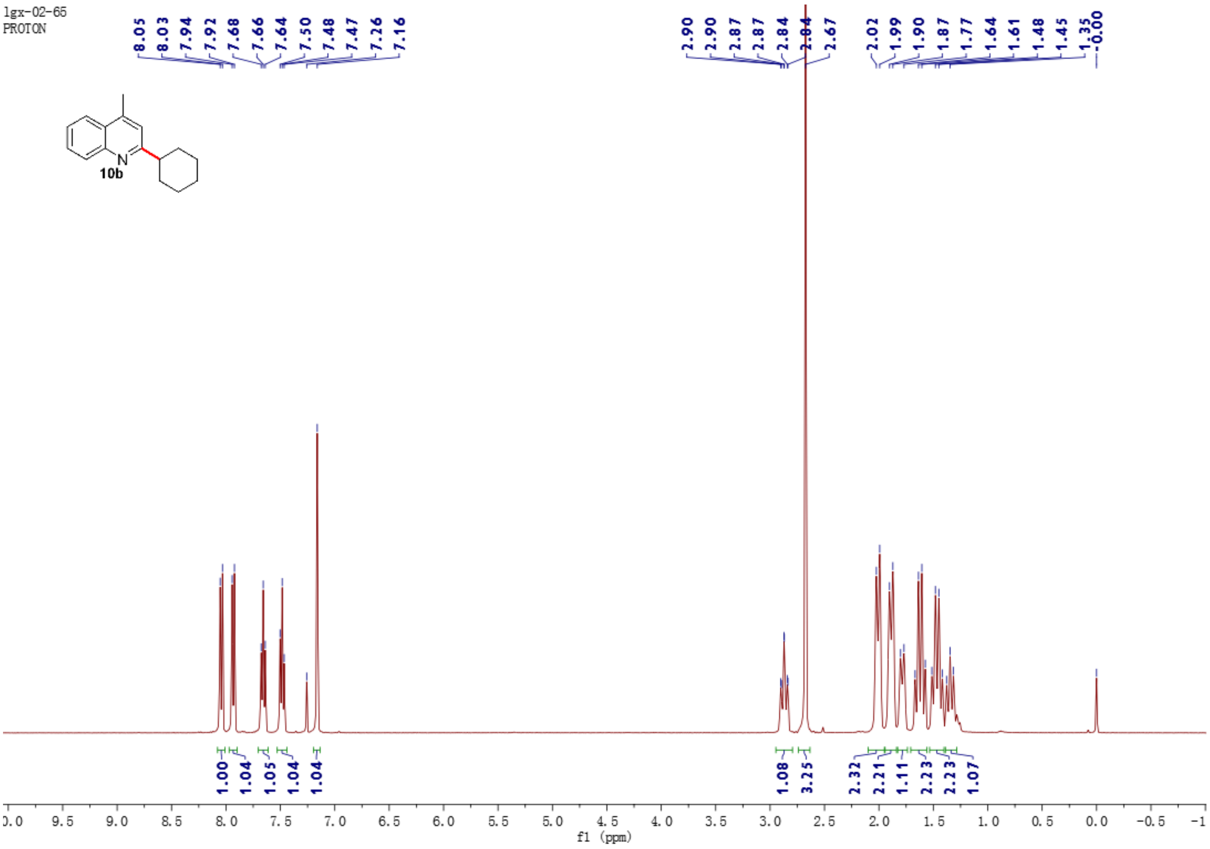

lgx-02-65
PROTON
8.05
8.03
7.94
7.92
7.68
7.66
7.64
7.50
7.48
7.47
7.26
7.16
2.90
2.90
2.87
2.87
2.84
2.84
2.67
2.02
1.99
1.90
1.87
1.77
1.64
1.61
1.48
1.45
1.35
0.00
N
10b
1.00
1.04
1.05
1.04
1.04
1.08
3.25
2.32
2.21
1.11
2.23
2.23
1.07
f1 (ppm)

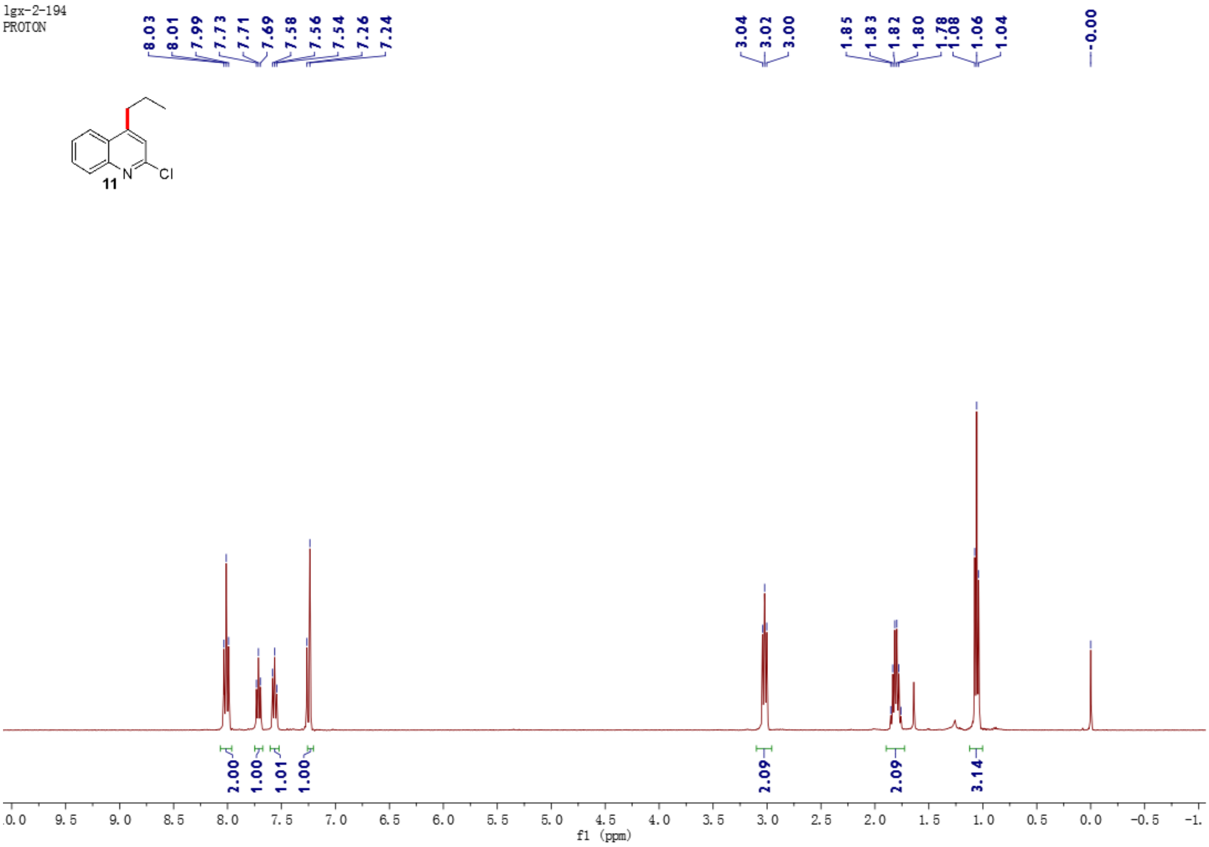

lgx-2-194
PROTON
8.03
8.01
7.99
7.73
7.71
7.69
7.58
7.56
7.54
7.26
7.24
3.04
3.02
3.00
1.85
1.83
1.82
1.80
1.78
1.08
1.06
1.04
-0.00
N
Cl
11
2.00
1.00
1.01
1.00
2.09
2.09
3.14
f1 (ppm)

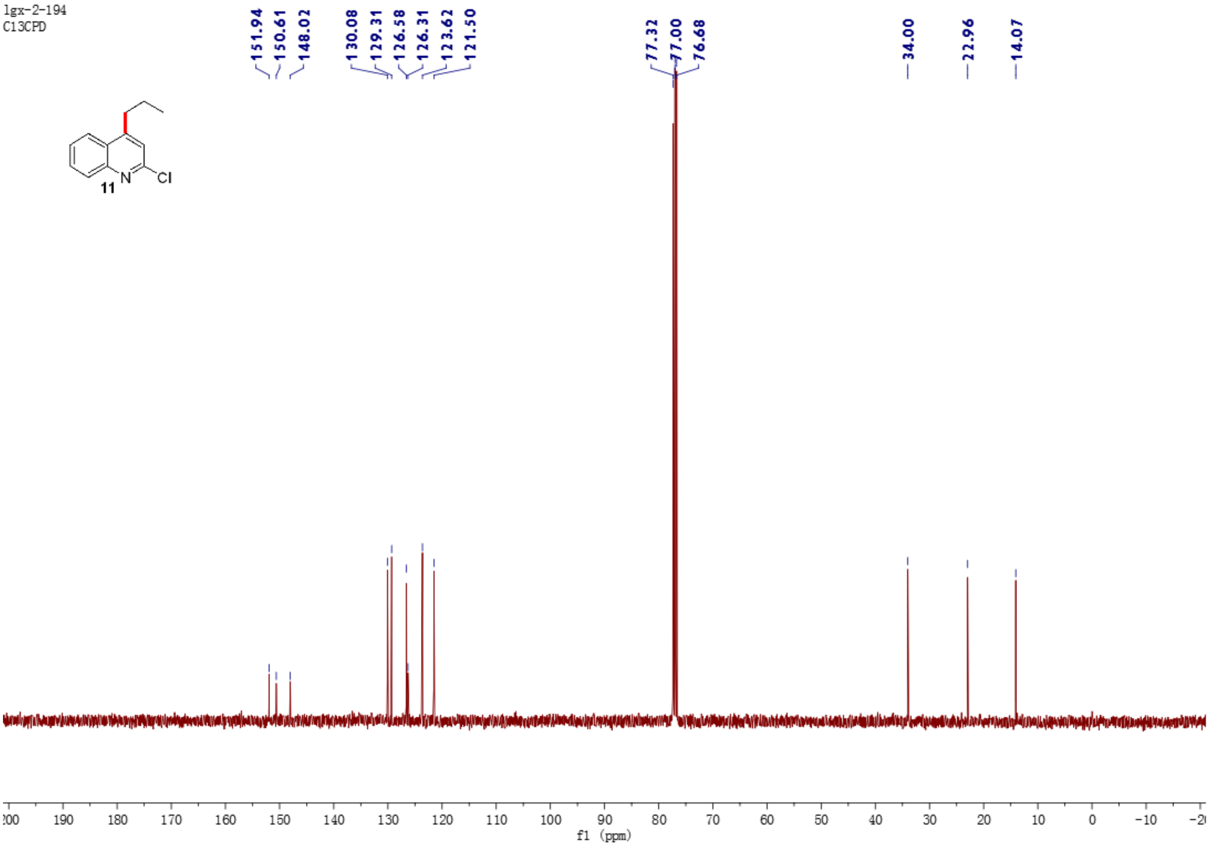

lgx-2-194
C13CPD
151.94
150.61
148.02
130.08
129.31
126.58
126.31
123.62
121.50
77.32
77.00
76.68
34.00
22.96
14.07
N
Cl
11
f1 (ppm)

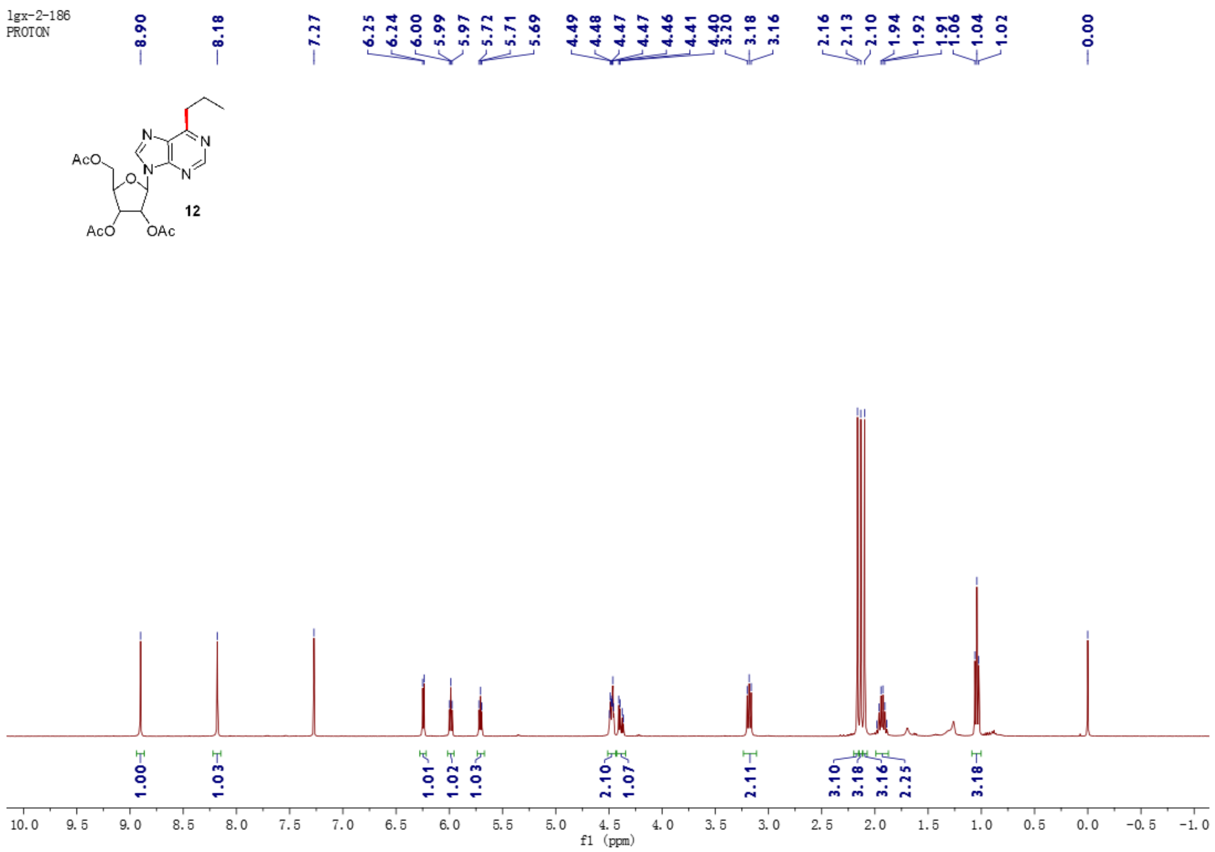
lgx-2-186
PROTON
8.90
8.18
7.27
6.25
6.24
6.00
5.99
5.97
5.72
5.71
5.69
4.49
4.48
4.47
4.47
4.46
4.41
4.40
3.20
3.18
3.16
2.16
2.13
2.10
1.94
1.92
1.91
1.06
1.04
1.02
0.00
AcO
N
N
N
N
O
AcO
OAc
12
1.00
1.03
1.01
1.02
1.03
2.10
1.07
2.11
3.10
3.18
3.16
2.25
3.18
f1 (ppm)

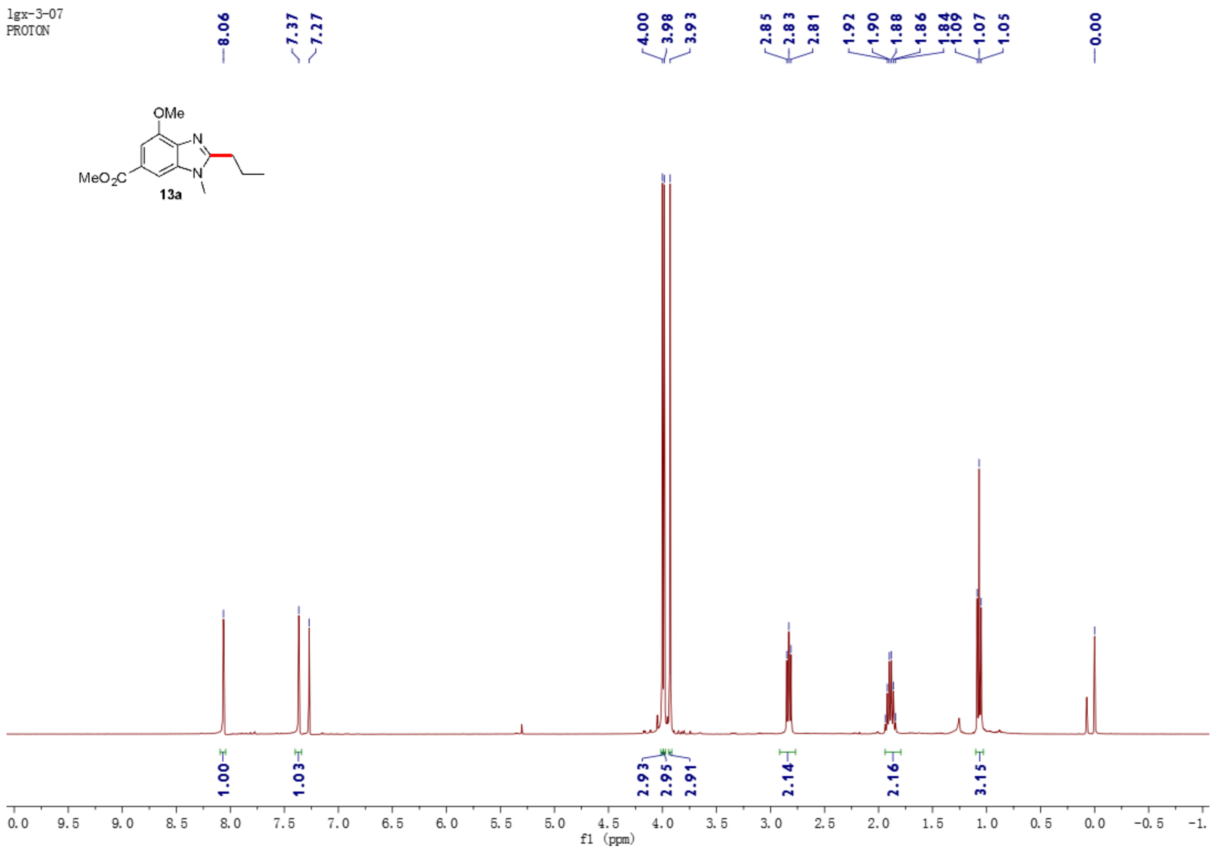
lgx-3-07
PROTON
8.06
7.37
7.27
4.00
3.98
3.93
2.85
2.83
2.81
1.92
1.90
1.88
1.86
1.84
1.09
1.07
1.05
0.00
OMe
N
N
MeO2C
13a
1.00
1.03
2.93
2.95
2.91
2.14
2.16
3.15
f1 (ppm)

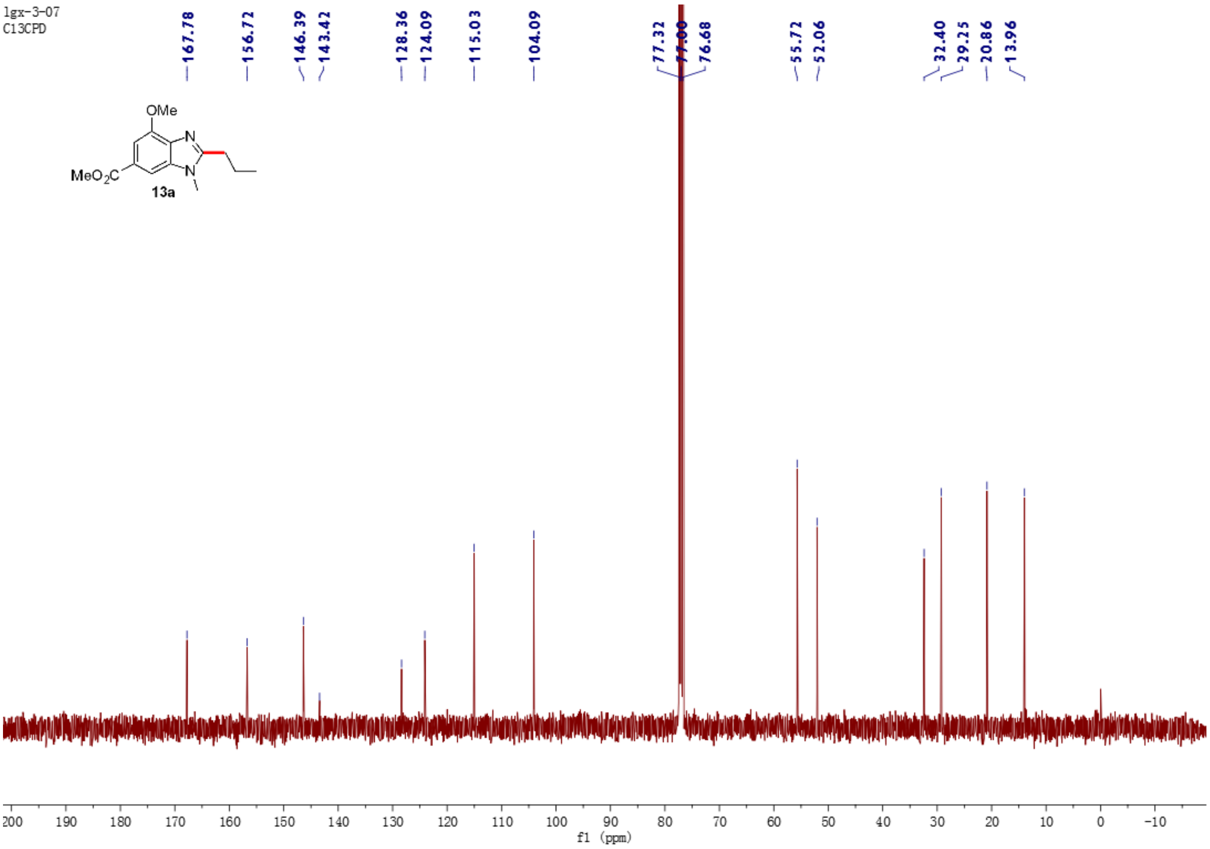

lgx-3-07
C13CPD
167.78
156.72
146.39
143.42
128.36
124.09
115.03
104.09
77.32
77.00
76.68
55.72
52.06
32.40
29.25
20.86
13.96
OMe
N
N
MeO2C
13a
200 190 180 170 160 150 140 130 120 110 100 90 80 70 60 50 40 30 20 10 0 -10
f1 (ppm)

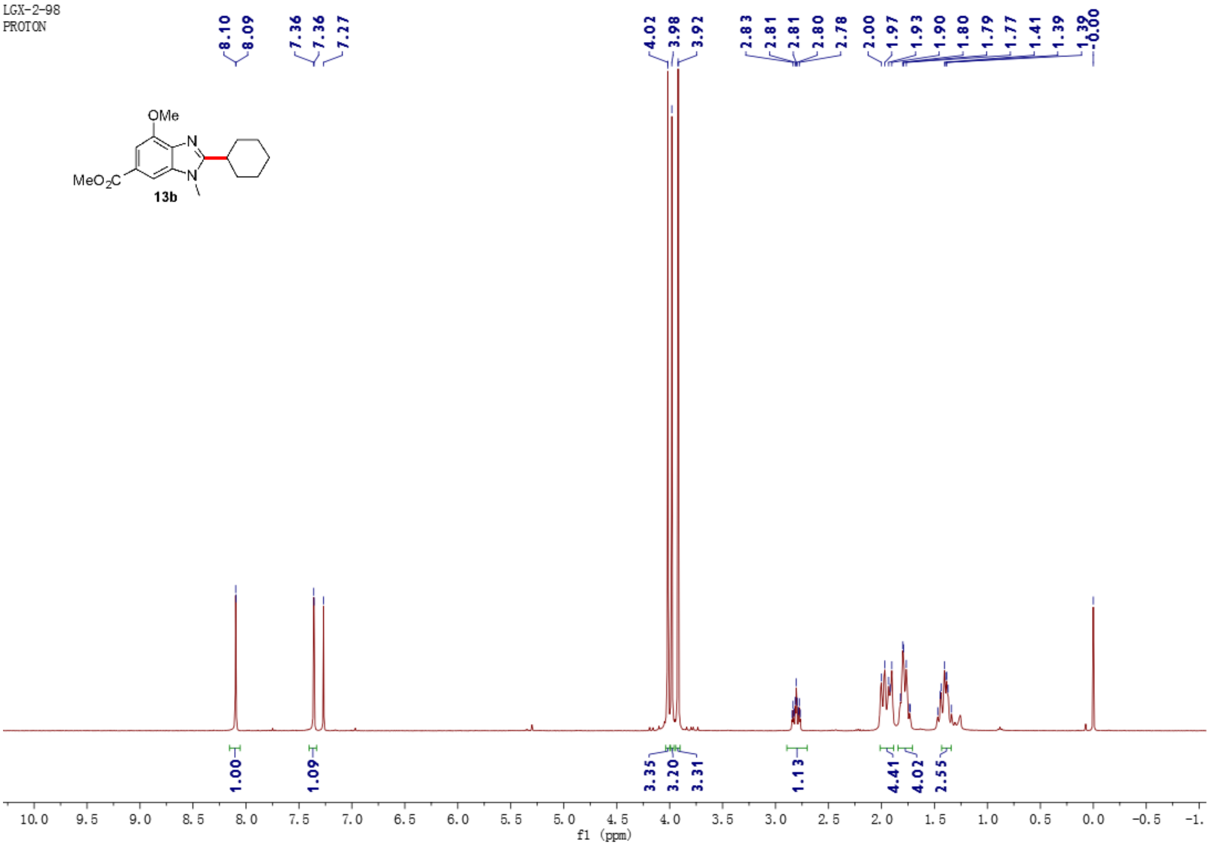

LGX-2-98
PROTON
8.10
8.09
7.36
7.36
7.27
4.02
3.98
3.92
2.83
2.81
2.81
2.80
2.78
2.00
1.97
1.93
1.90
1.80
1.79
1.77
1.41
1.39
1.39
0.00
OMe
N
N
MeO2C
13b
1.00
1.09
3.35
3.20
3.31
1.13
4.41
4.02
2.55
10.0 9.5 9.0 8.5 8.0 7.5 7.0 6.5 6.0 5.5 5.0 4.5 4.0 3.5 3.0 2.5 2.0 1.5 1.0 0.5 0.0 -0.5 -1.
f1 (ppm)

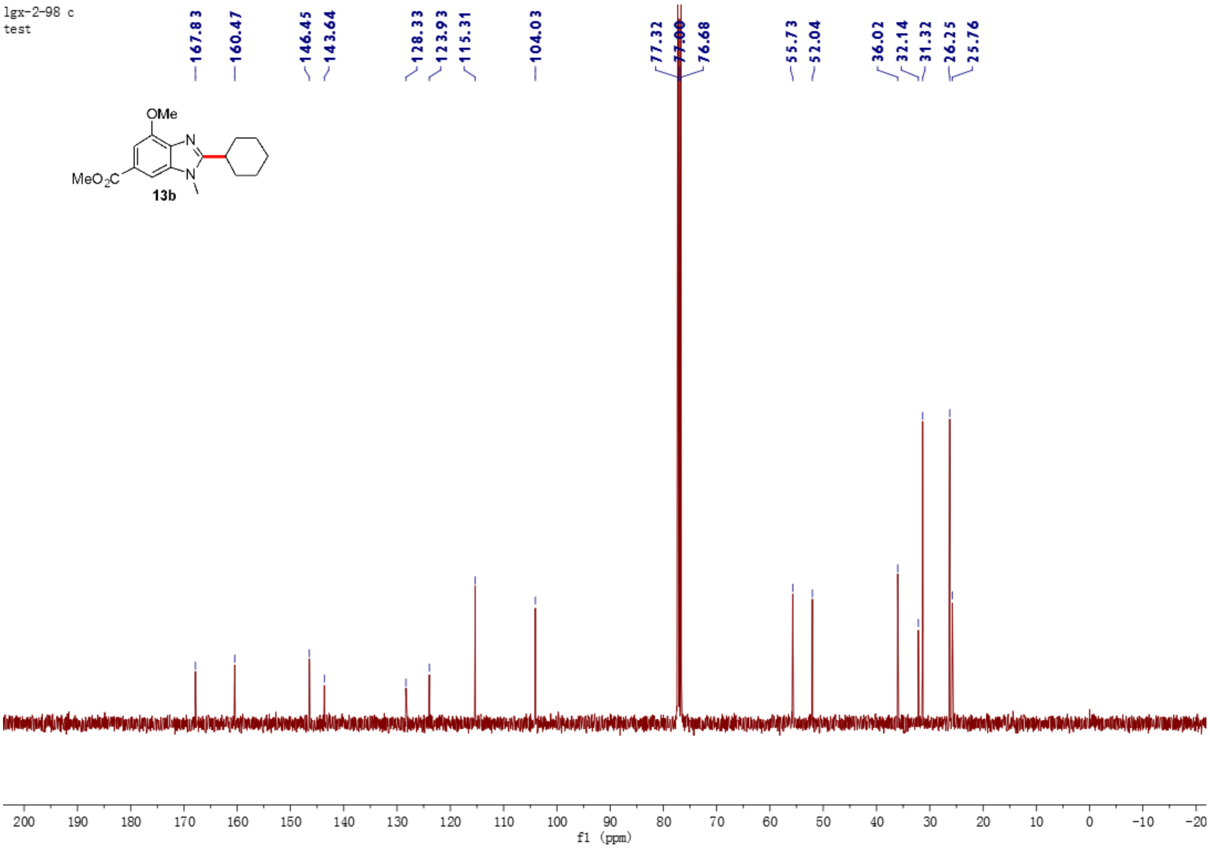
lgx-2-98 c
test
167.83
160.47
146.45
143.64
128.33
123.93
115.31
104.03
77.32
77.00
76.68
55.73
52.04
36.02
32.14
31.32
26.25
25.76
OMe
N
N
MeO2C
13b
f1 (ppm)

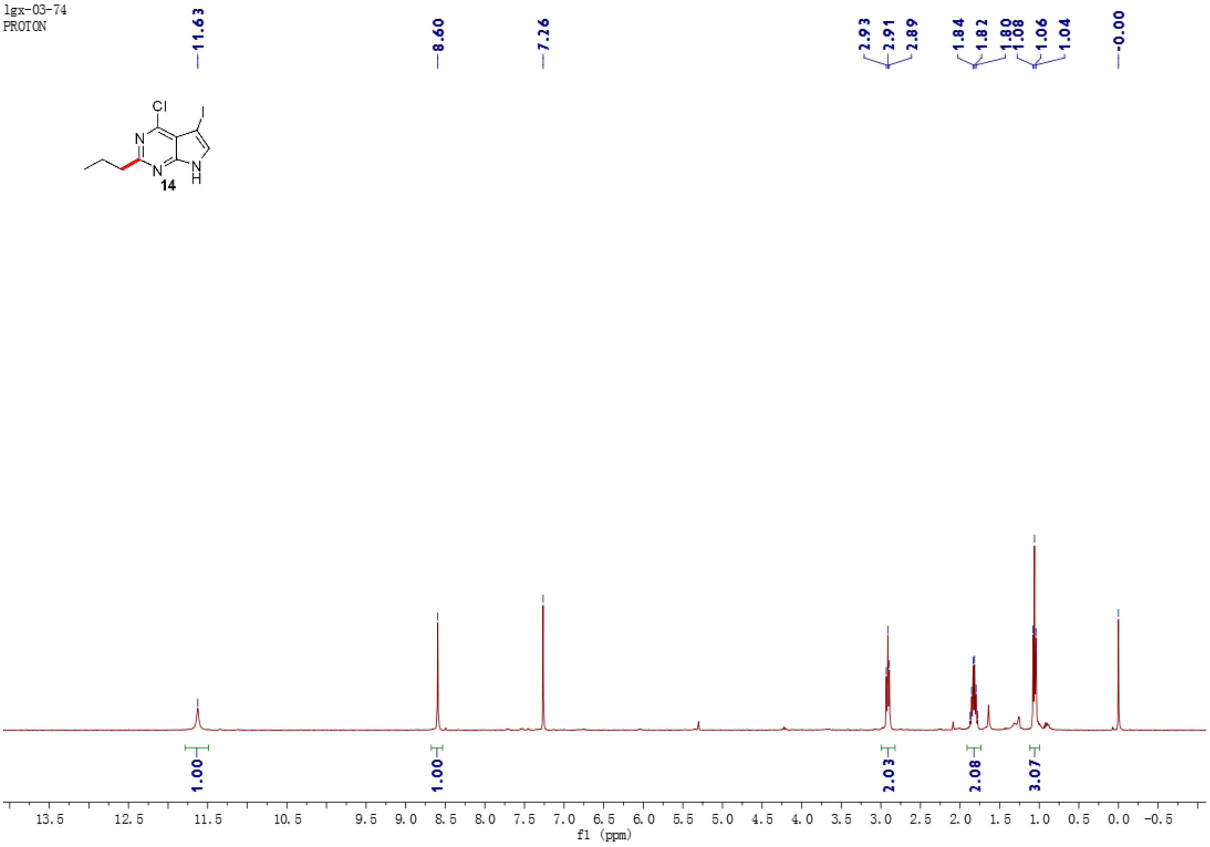
lgx-03-74
PROTON
11.63
8.60
7.26
2.93
2.91
2.89
1.84
1.82
1.80
1.08
1.06
1.04
-0.00
Cl
I
N
N
H
14
1.00
1.00
2.03
2.08
3.07
f1 (ppm)

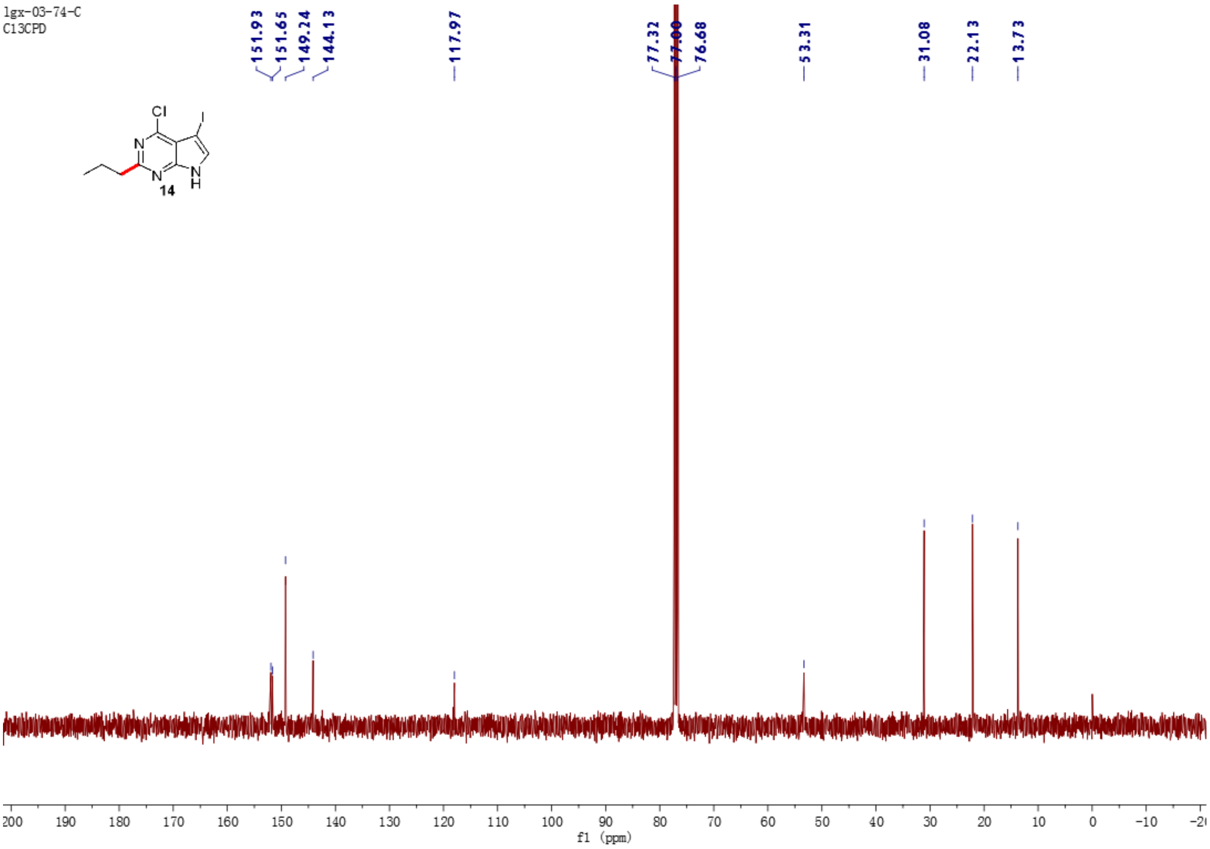

lgx-03-74-C
C13CPD
151.93
151.65
149.24
144.13
117.97
77.32
77.00
76.68
53.31
31.08
22.13
13.73
Cl
I
N
N
N
H
14
200 190 180 170 160 150 140 130 120 110 100 90 80 70 60 50 40 30 20 10 0 -10
f1 (ppm)

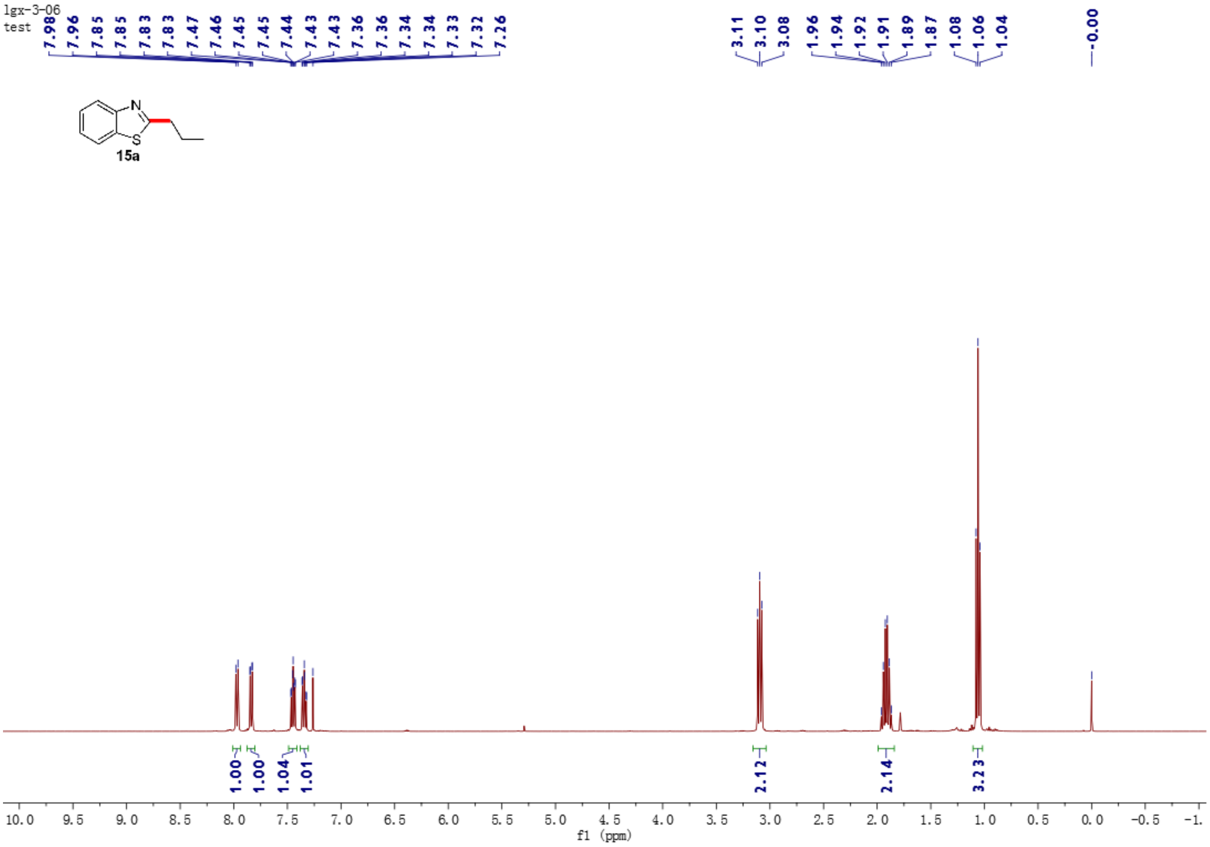

lgx-3-06
test
7.98
7.96
7.85
7.85
7.83
7.83
7.47
7.46
7.45
7.45
7.44
7.43
7.43
7.36
7.34
7.34
7.33
7.32
7.26
3.11
3.10
3.08
1.96
1.94
1.92
1.91
1.89
1.87
1.08
1.06
1.04
-0.00
N
S
15a
1.00
1.00
1.04
1.01
2.12
2.14
3.23
10.0 9.5 9.0 8.5 8.0 7.5 7.0 6.5 6.0 5.5 5.0 4.5 4.0 3.5 3.0 2.5 2.0 1.5 1.0 0.5 0.0 -0.5
f1 (ppm)

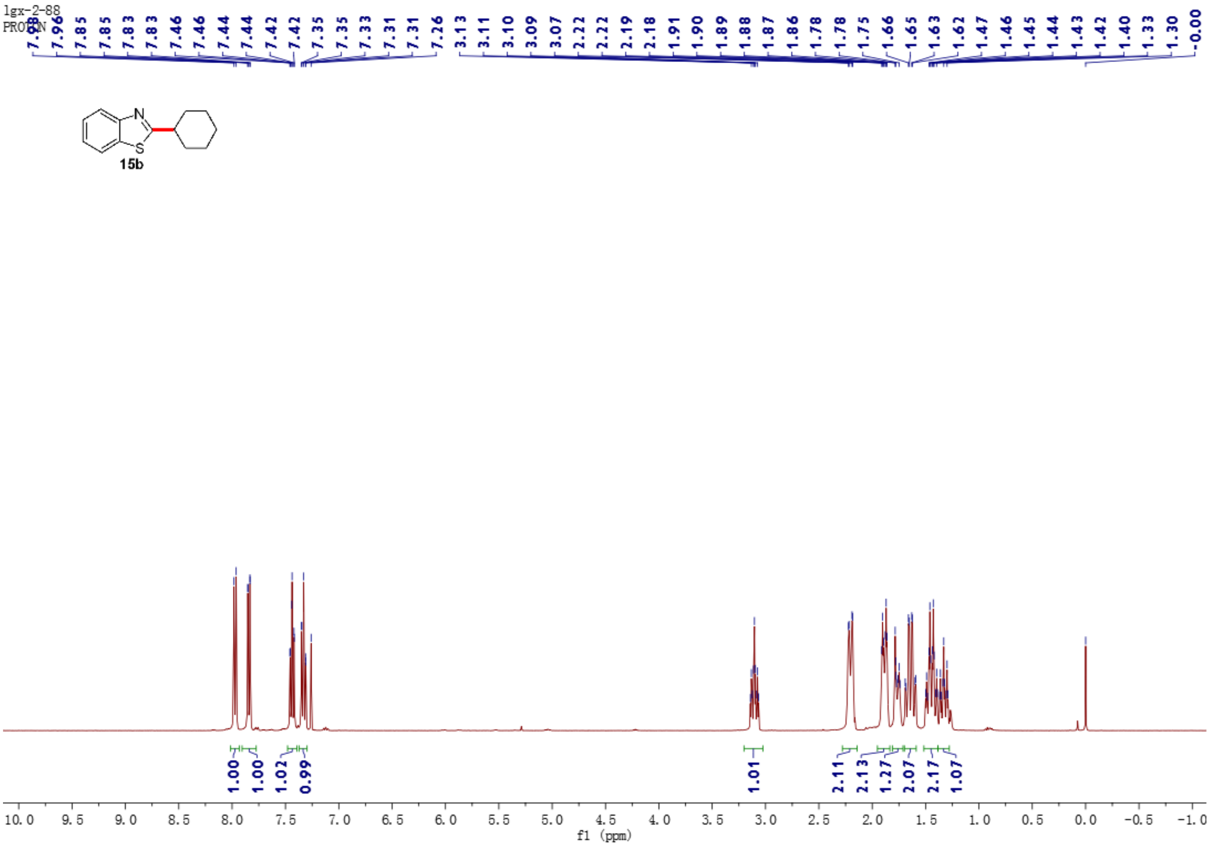
lgx-2-88
PROTON
7.96
7.85
7.85
7.83
7.83
7.46
7.46
7.44
7.44
7.42
7.42
7.35
7.35
7.33
7.31
7.31
7.26
3.13
3.11
3.10
3.09
3.07
2.22
2.22
2.19
2.18
1.91
1.90
1.89
1.88
1.87
1.86
1.78
1.78
1.75
1.66
1.65
1.63
1.62
1.47
1.46
1.45
1.44
1.43
1.42
1.40
1.33
1.30
-0.00
15b
1.00
1.00
1.02
0.99
1.01
2.11
2.13
1.27
2.07
2.17
1.07
f1 (ppm)

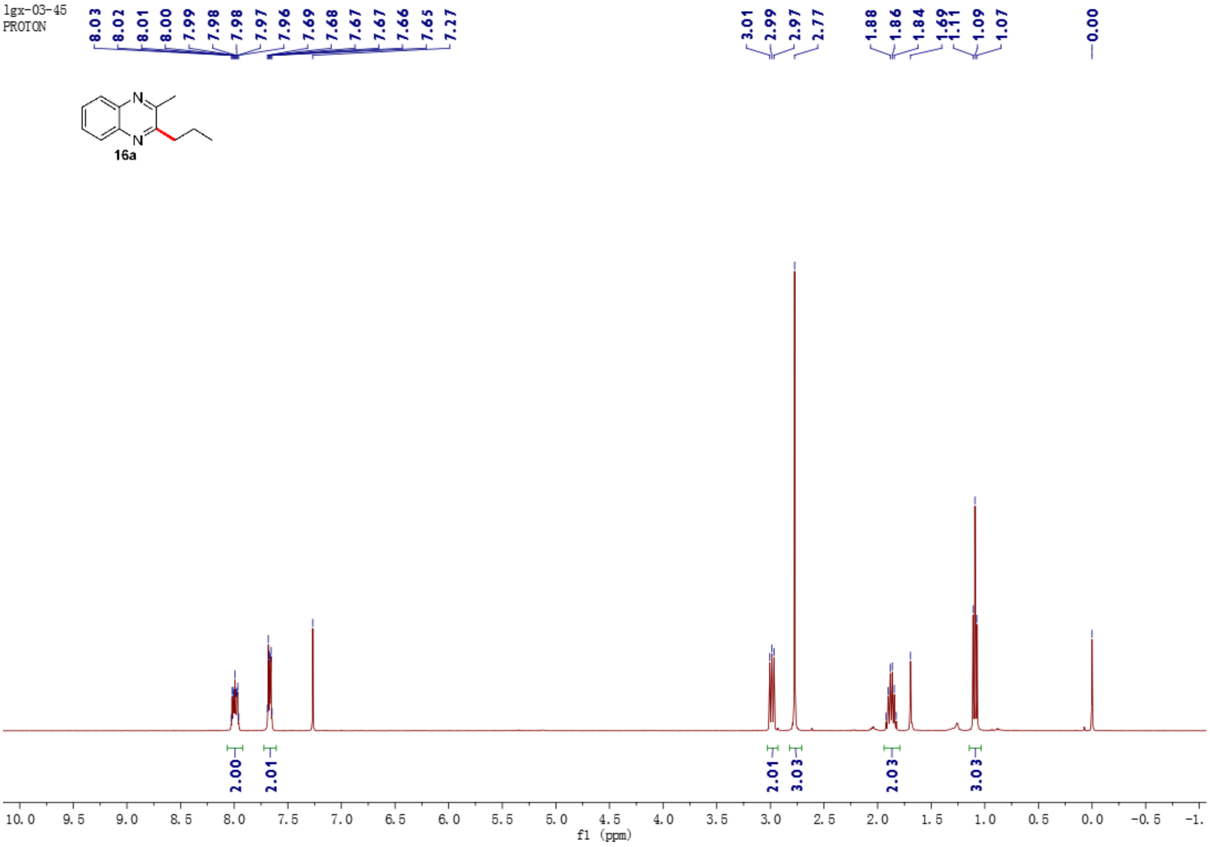
lgx-03-45
PROTON
8.03
8.02
8.01
8.00
7.99
7.98
7.98
7.97
7.96
7.69
7.68
7.67
7.67
7.66
7.65
7.27
3.01
2.99
2.97
2.77
1.88
1.86
1.84
1.69
1.11
1.09
1.07
0.00
16a
2.00
2.01
2.01
3.03
2.03
3.03
f1 (ppm)

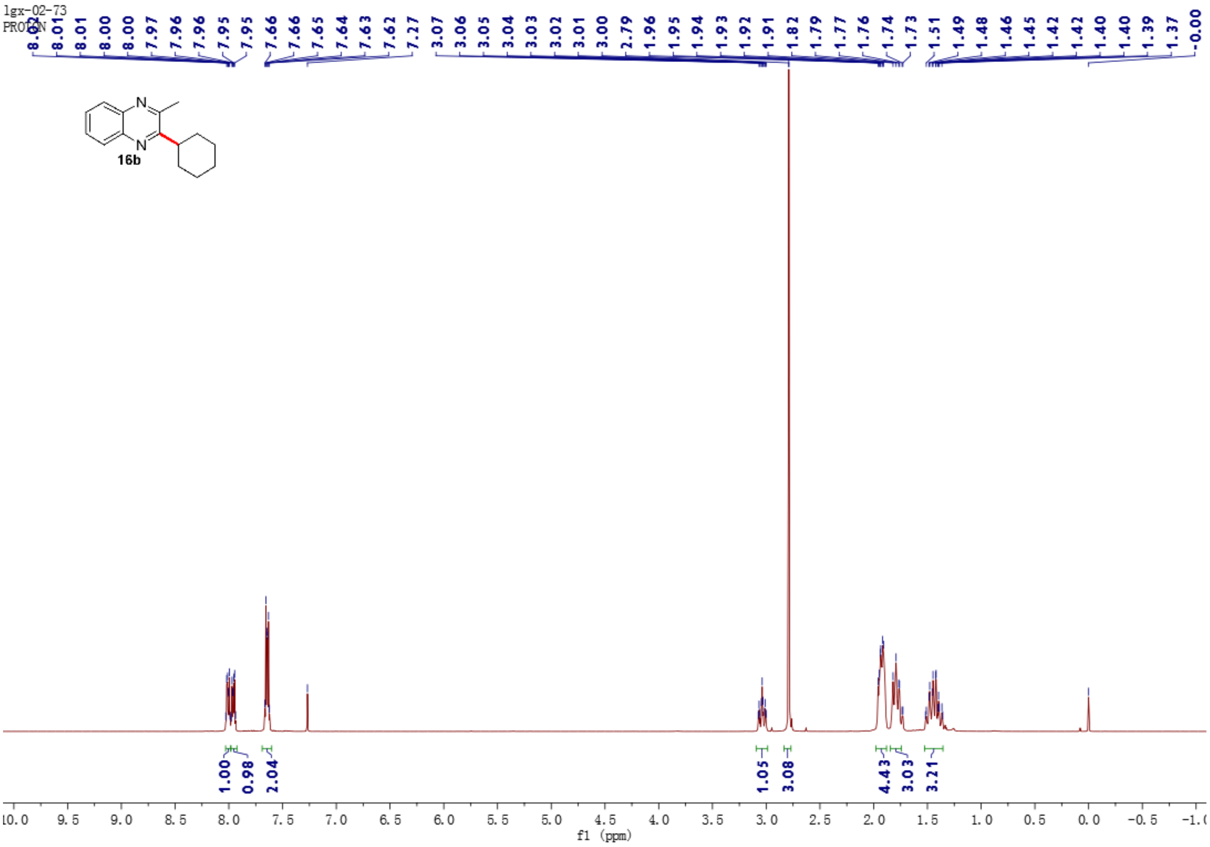
lgx-02-73
PROTON
16b
f1 (ppm)

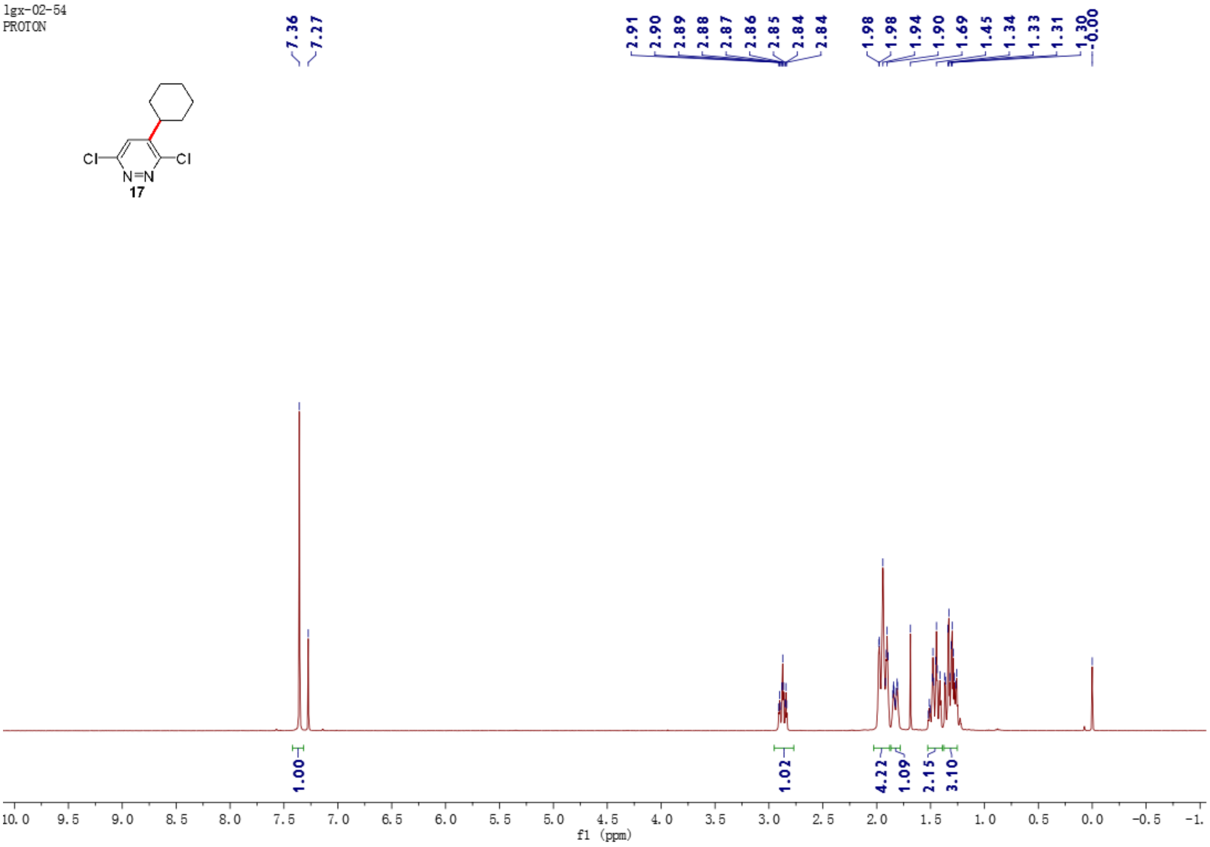
lgx-02-54
PROTON
17
f1 (ppm)

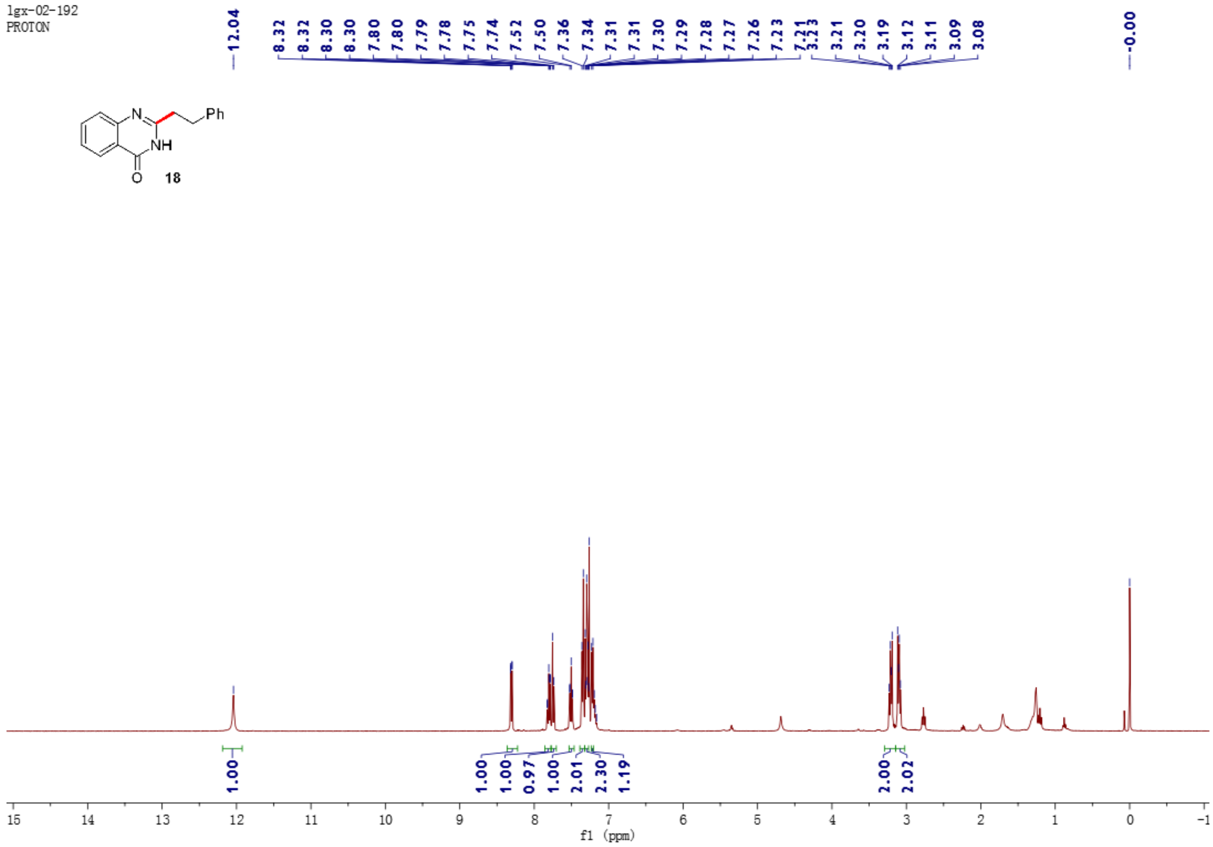
lgx-02-192
PROTON
18
f1 (ppm)

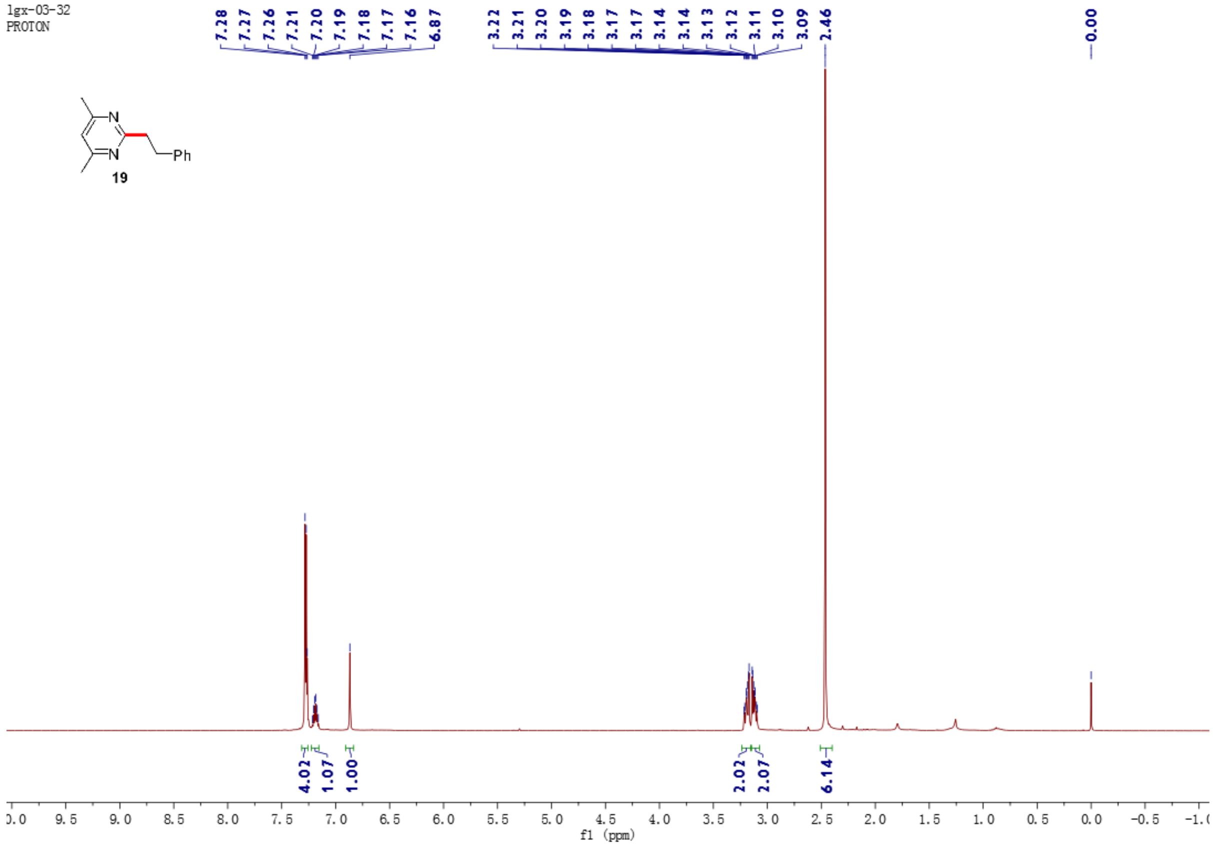
lgx-03-32
PROTON
19
f1 (ppm)

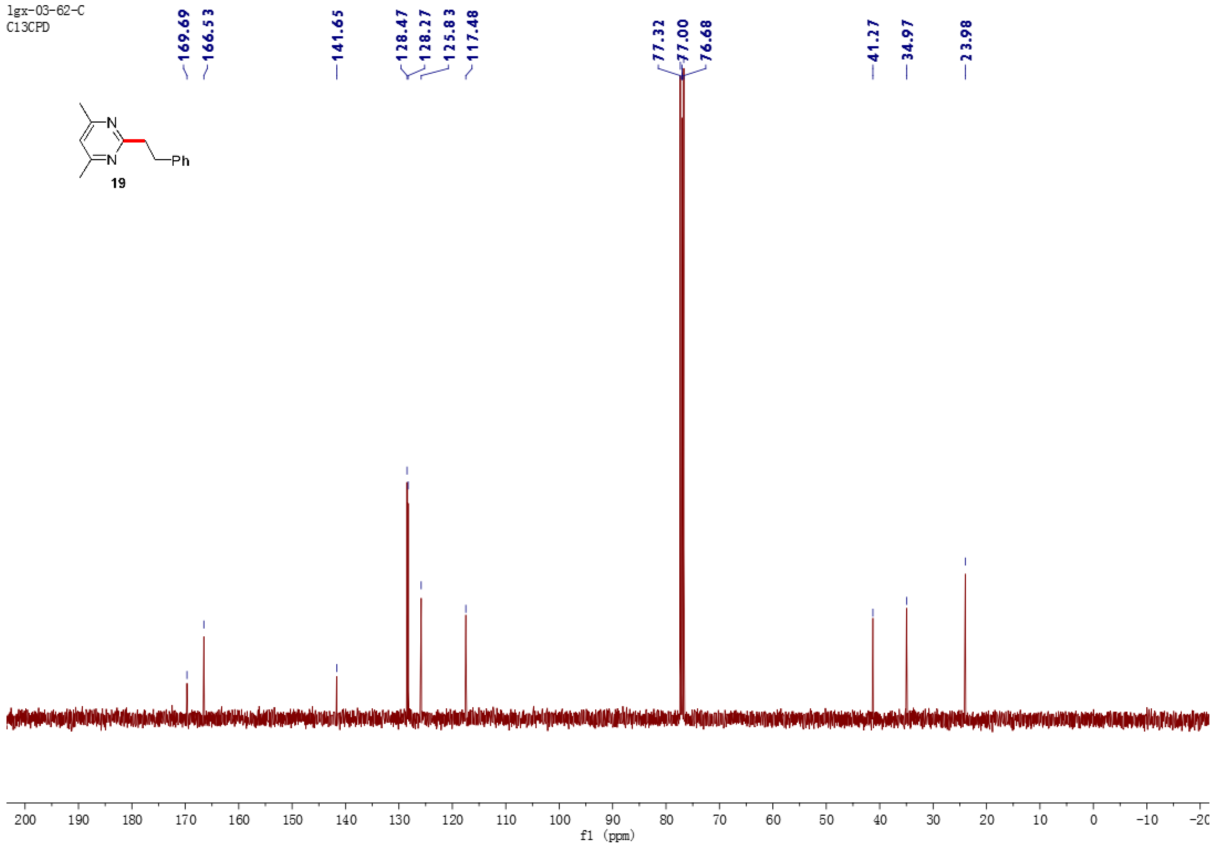
lgx-03-62-C
C13CPD
169.69
166.53
141.65
128.47
128.27
125.83
117.48
77.32
77.00
76.68
41.27
34.97
23.98
N
N
Ph
19
f1 (ppm)

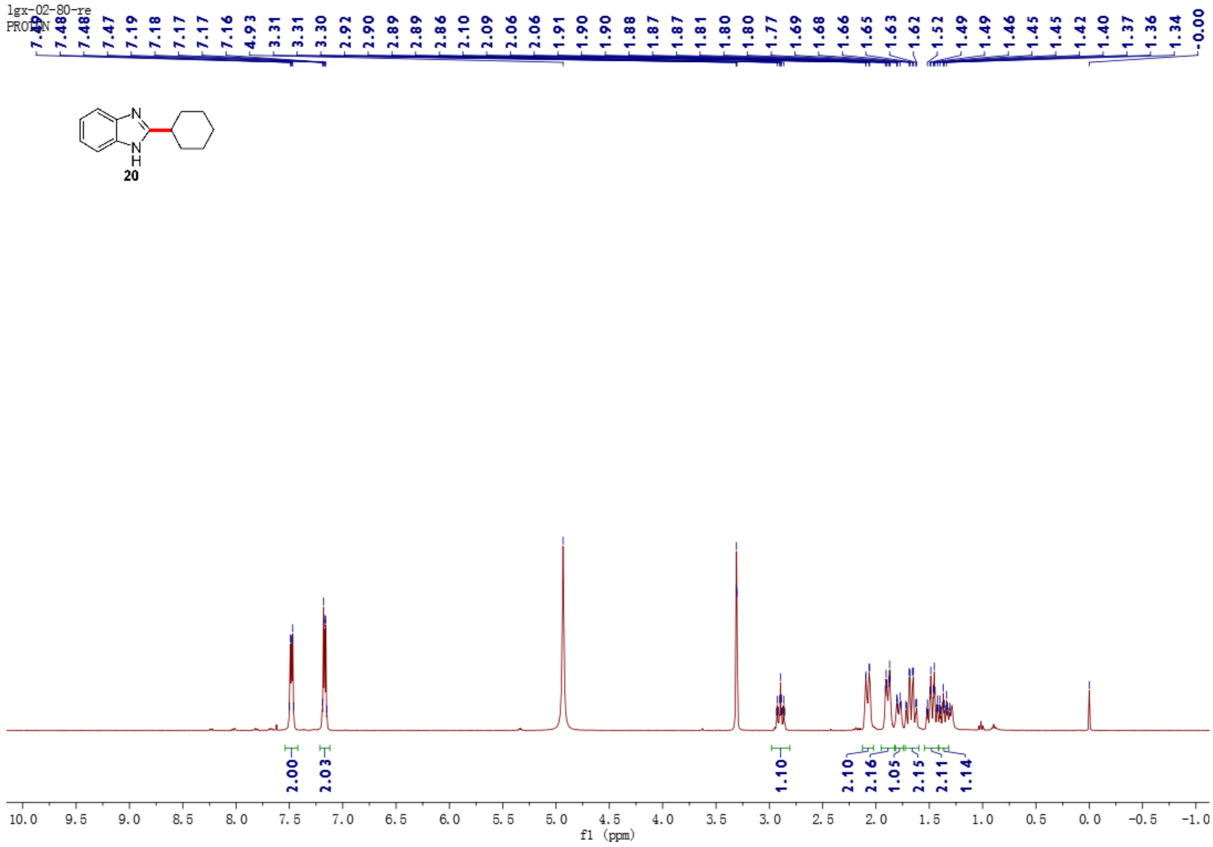
lgx-02-80-re
7.48
7.47
7.19
7.18
7.17
7.17
7.16
4.93
3.31
3.31
3.30
2.92
2.90
2.89
2.89
2.86
2.10
2.09
2.06
2.06
1.91
1.90
1.90
1.88
1.87
1.87
1.81
1.80
1.80
1.77
1.69
1.68
1.66
1.65
1.63
1.62
1.52
1.49
1.49
1.46
1.45
1.45
1.42
1.40
1.37
1.36
1.34
-0.00
N
N
H
20
2.00
2.03
1.10
2.10
2.16
1.05
2.15
2.11
1.14
f1 (ppm)

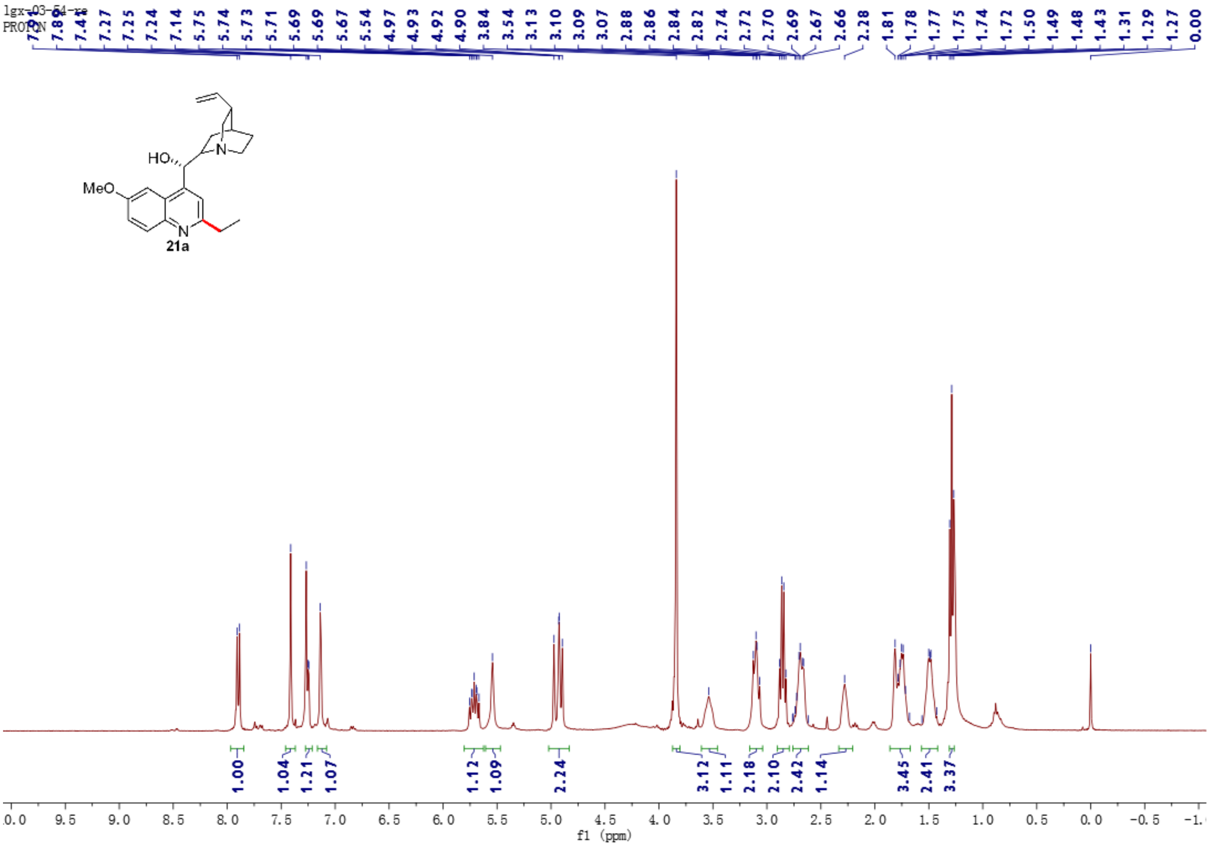
PROTON
7.89 7.41 7.27 7.25 7.24 7.14 5.75 5.74 5.73 5.71 5.69 5.69 5.67 5.54 4.97 4.93 4.92 4.90 3.84 3.54 3.13 3.10 3.09 3.07 2.88 2.86 2.84 2.82 2.74 2.72 2.70 2.69 2.67 2.66 2.28 1.81 1.78 1.77 1.75 1.74 1.72 1.50 1.49 1.48 1.43 1.31 1.29 1.27 0.00
HO
MeO
N
21a
1.00 1.04 1.21 1.07 1.12 1.09 2.24 3.12 1.11 2.18 2.10 2.42 1.14 3.45 2.41 3.37
f1 (ppm)

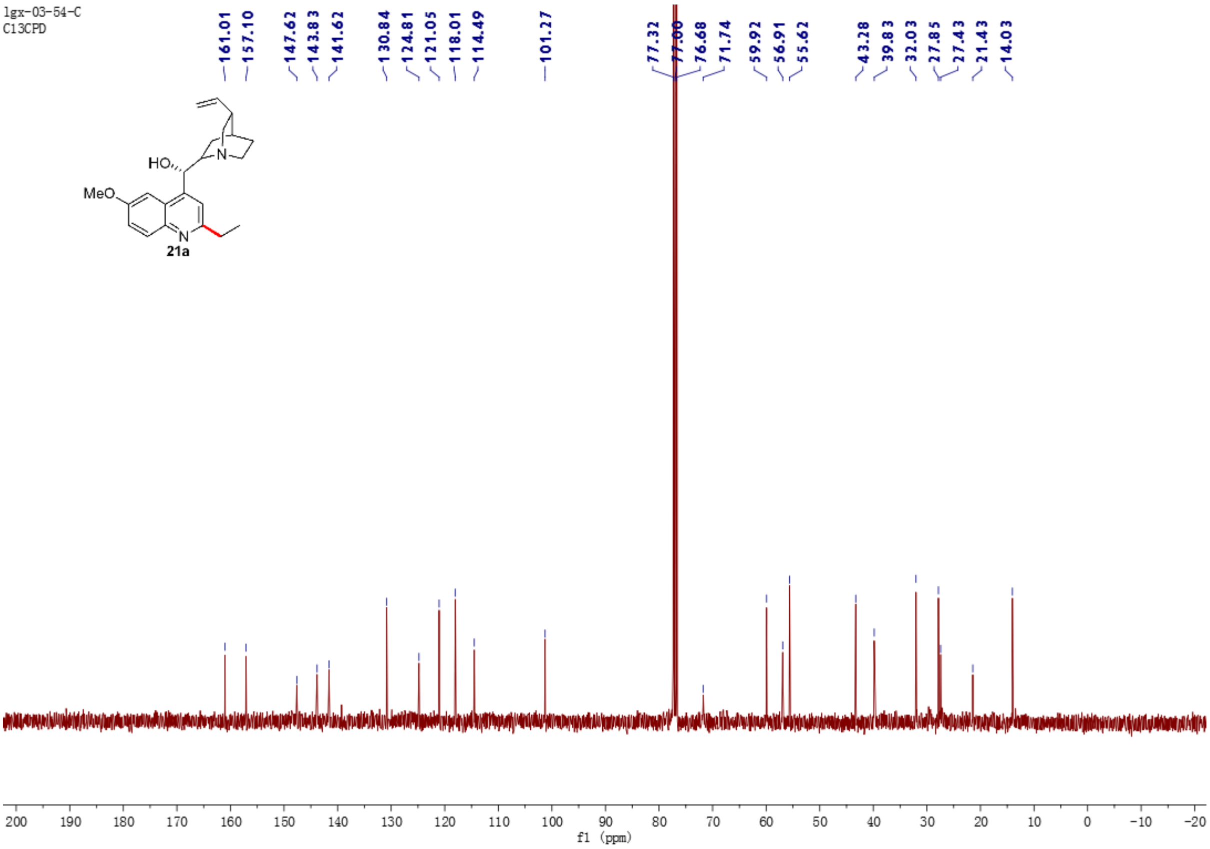
lgx-03-54-C
C13CPD
161.01 157.10 147.62 143.83 141.62 130.84 124.81 121.05 118.01 114.49 101.27 77.32 77.00 76.68 71.74 59.92 56.91 55.62 43.28 39.83 32.03 27.85 27.43 21.43 14.03
HO
MeO
N
21a
f1 (ppm)

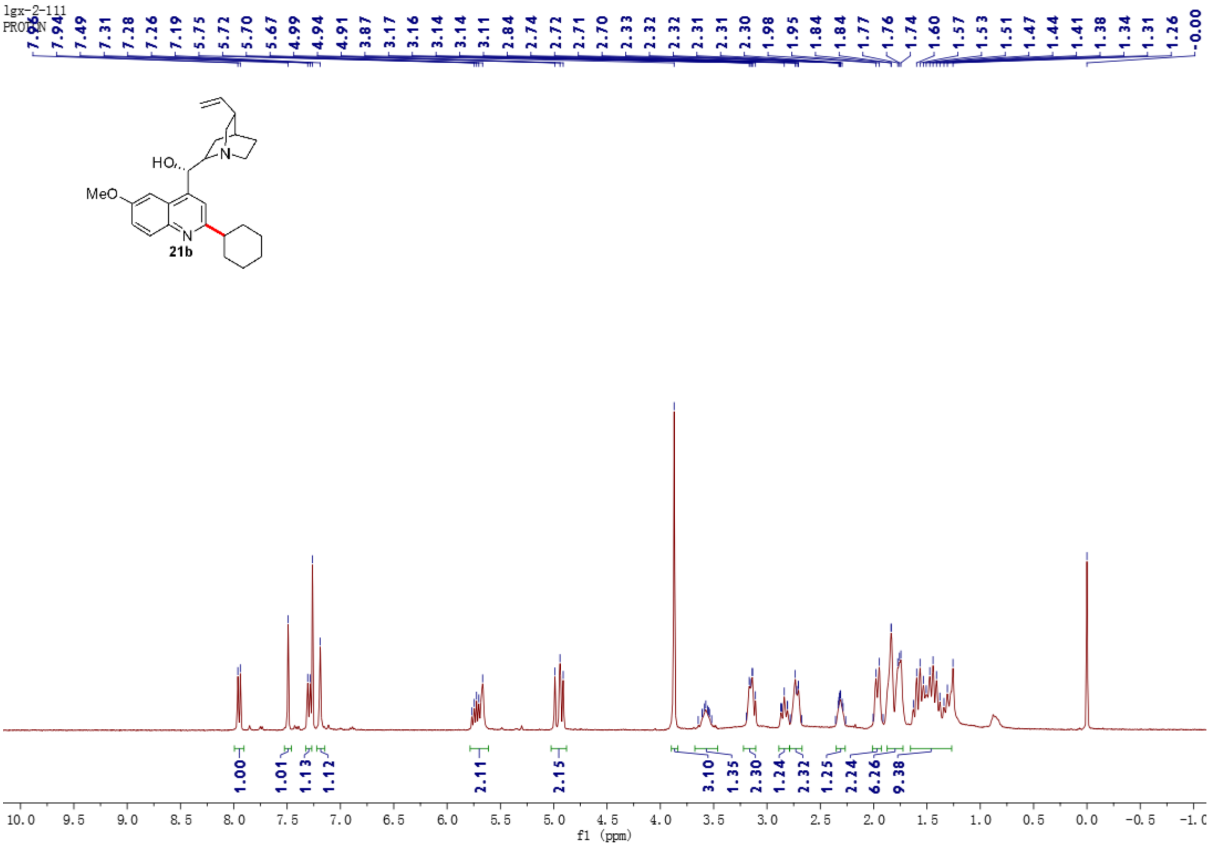

lgx-2-111
PROTON
7.94
7.49
7.31
7.28
7.26
7.19
5.75
5.72
5.70
5.67
4.99
4.94
4.91
3.87
3.17
3.16
3.14
3.14
3.11
2.84
2.74
2.72
2.71
2.70
2.33
2.32
2.32
2.31
2.31
2.30
1.98
1.95
1.84
1.84
1.77
1.76
1.74
1.60
1.57
1.53
1.51
1.47
1.44
1.41
1.38
1.34
1.31
1.26
-0.00
HO
MeO
N
21b
1.00
1.01
1.13
1.12
2.11
2.15
3.10
1.35
2.30
1.24
2.32
1.25
2.24
6.26
9.38
f1 (ppm)

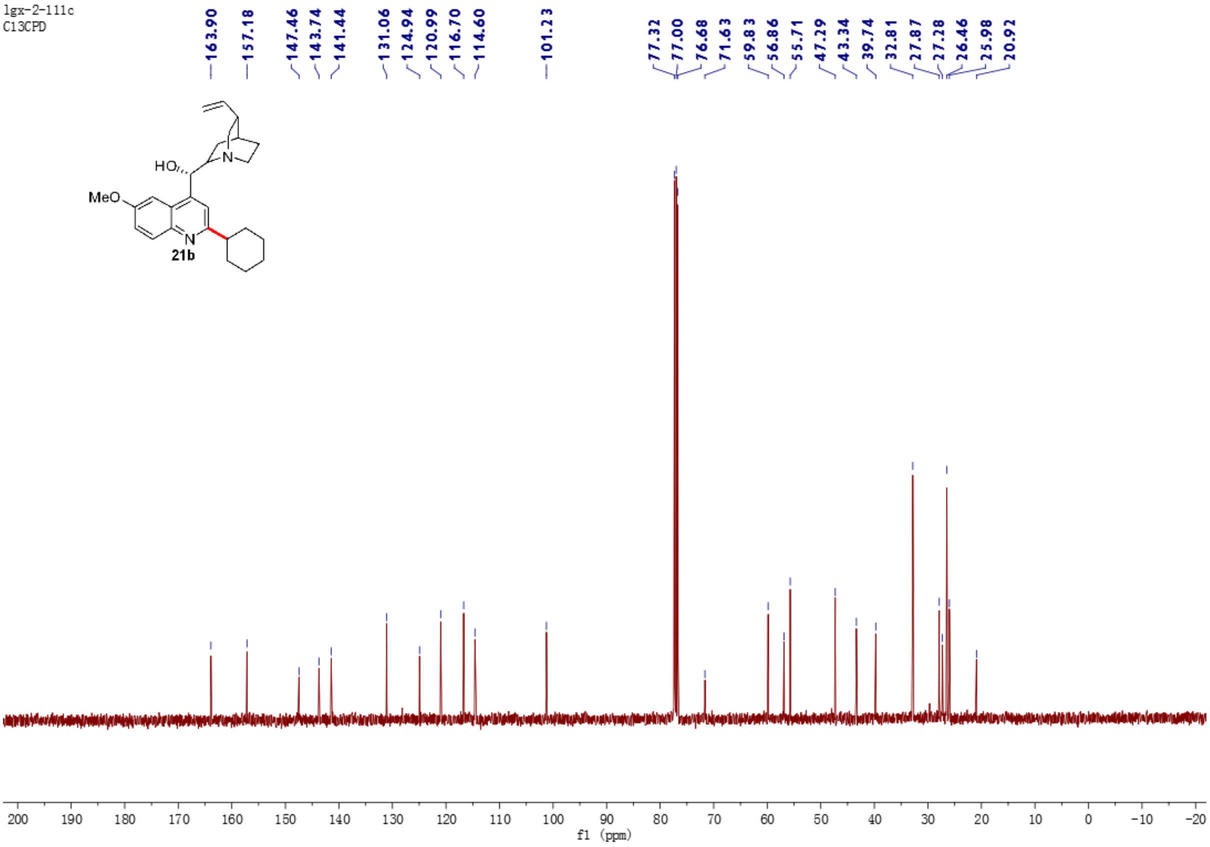

lgx-2-111c
C13CPD
163.90
157.18
147.46
143.74
141.44
131.06
124.94
120.99
116.70
114.60
101.23
77.32
77.00
76.68
71.63
59.83
56.86
55.71
47.29
43.34
39.74
32.81
27.87
27.28
26.46
25.98
20.92
HO
MeO
N
21b
f1 (ppm)

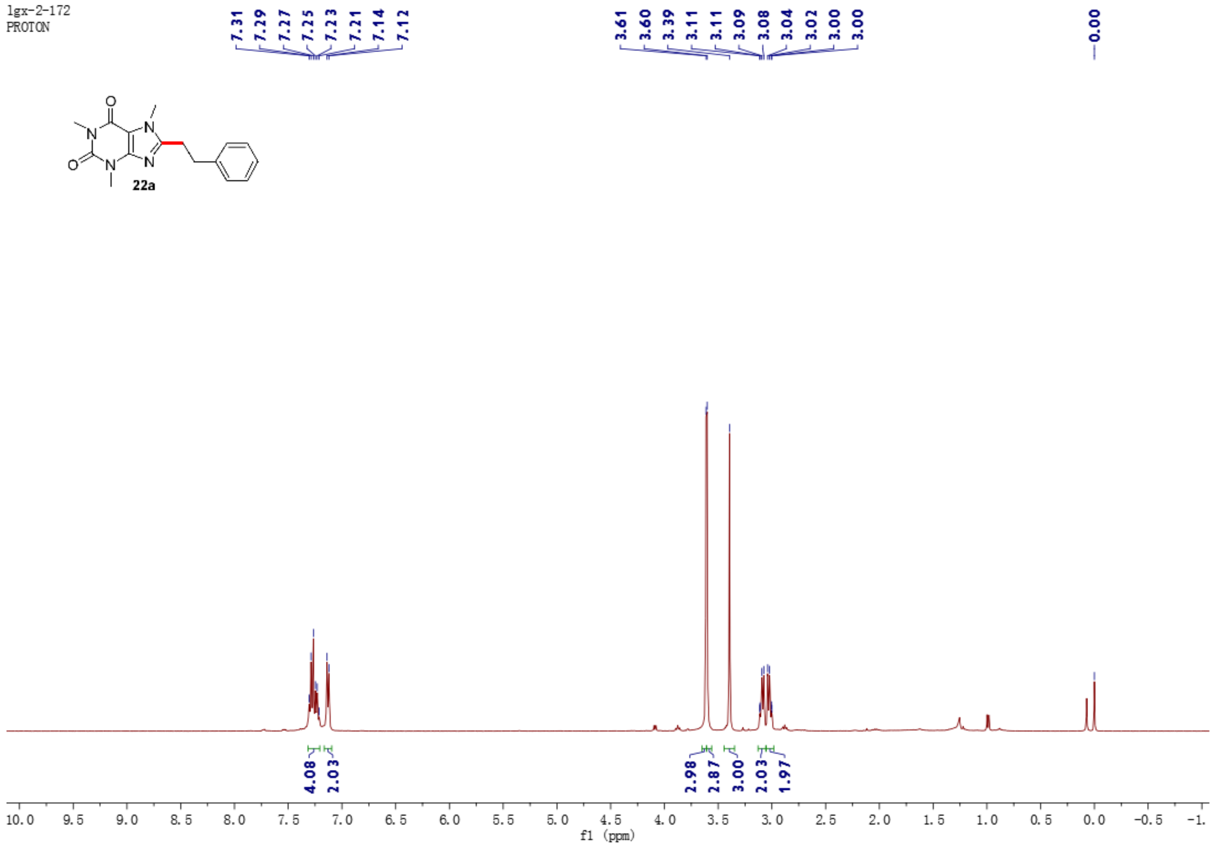
lgx-2-172
PROTON
7.31
7.29
7.27
7.25
7.23
7.21
7.14
7.12
3.61
3.60
3.39
3.11
3.11
3.09
3.08
3.04
3.02
3.00
3.00
0.00
22a
4.08
2.03
2.98
2.87
3.00
2.03
1.97
f1 (ppm)

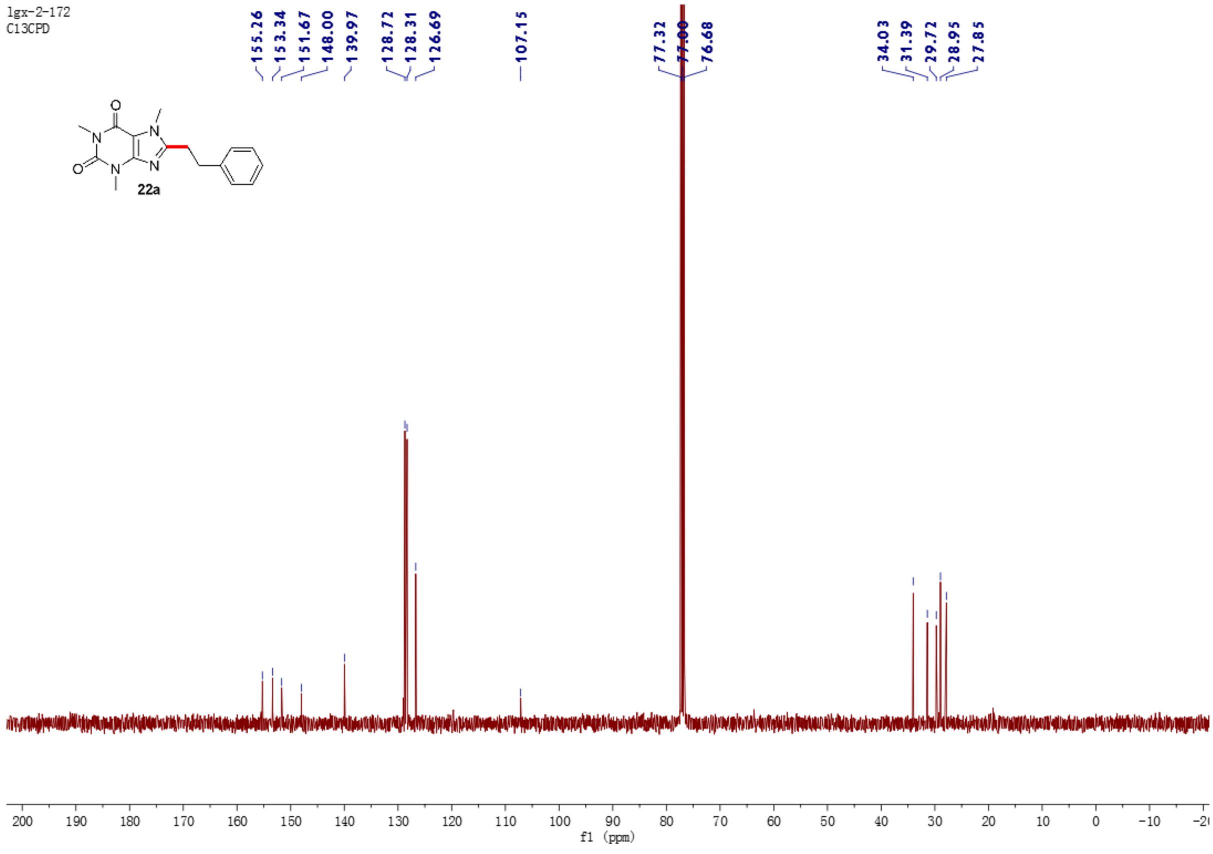
lgx-2-172
C13CPD
155.26
153.34
151.67
148.00
139.97
128.72
128.31
126.69
107.15
77.32
77.00
76.68
34.03
31.39
29.72
28.95
27.85
22a
f1 (ppm)

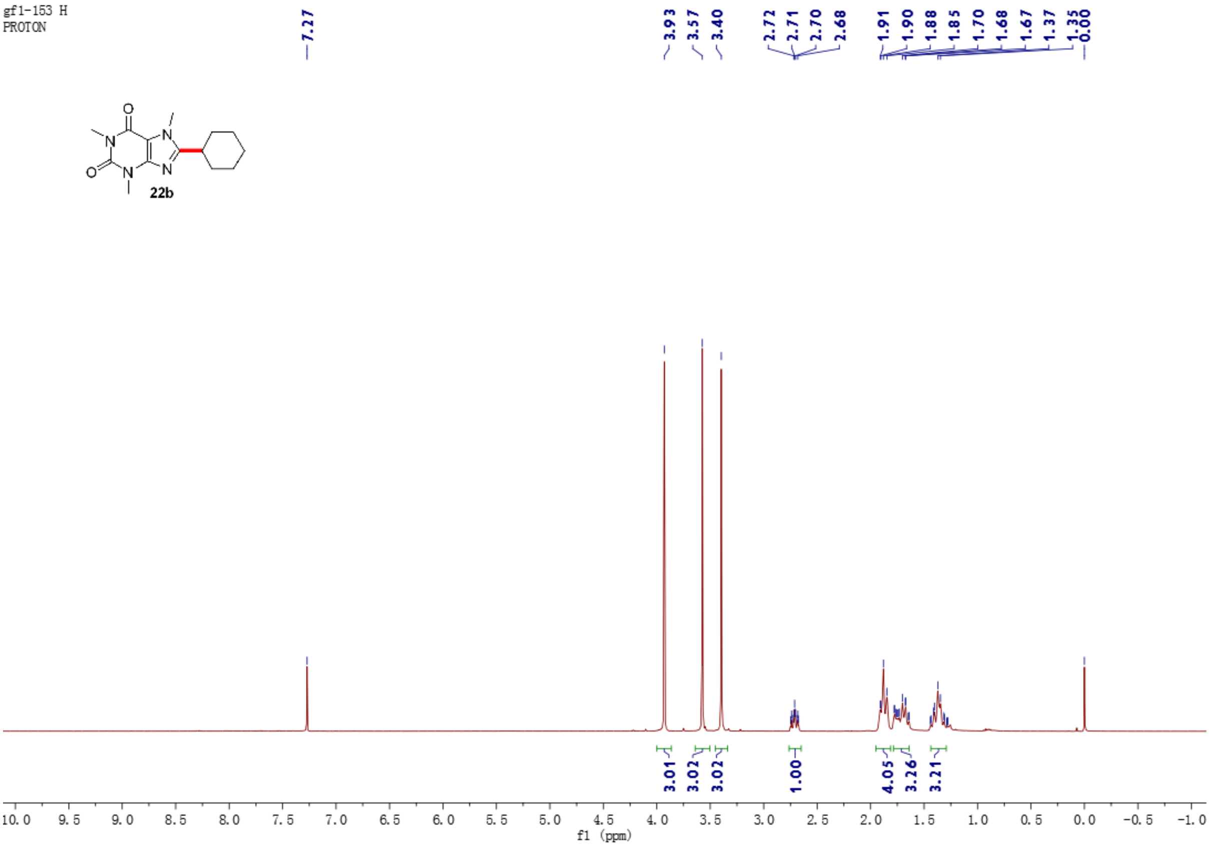
gf1-153 H
PROTON
7.27
3.93
3.57
3.40
2.72
2.71
2.70
2.68
1.91
1.90
1.88
1.85
1.70
1.68
1.67
1.37
1.35
0.00
22b
3.01
3.02
3.02
1.00
4.05
3.26
3.21
10.0 9.5 9.0 8.5 8.0 7.5 7.0 6.5 6.0 5.5 5.0 4.5 4.0 3.5 3.0 2.5 2.0 1.5 1.0 0.5 0.0 -0.5 -1.0
f1 (ppm)

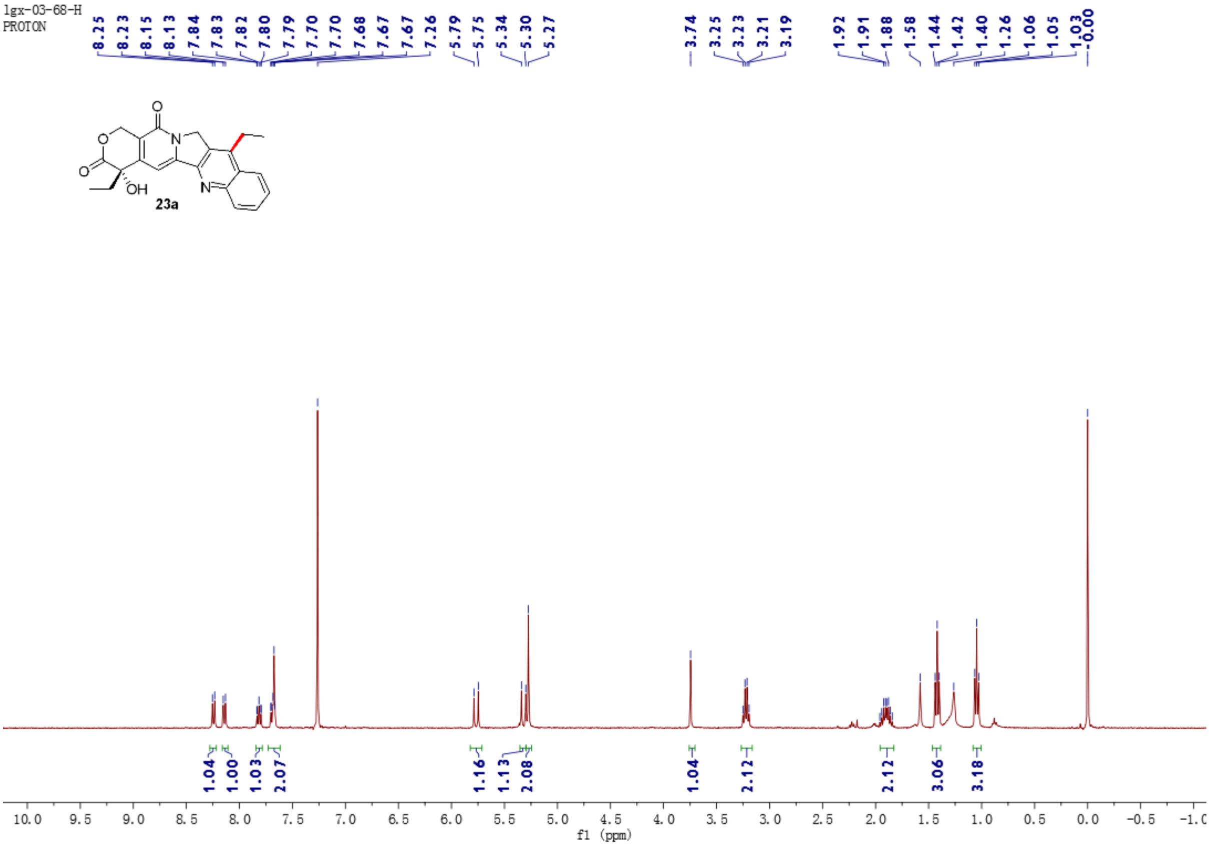
lgx-03-68-H
PROTON
8.25
8.23
8.15
8.13
7.84
7.83
7.82
7.80
7.79
7.70
7.70
7.68
7.67
7.67
7.26
5.79
5.75
5.34
5.30
5.27
3.74
3.25
3.23
3.21
3.19
1.92
1.91
1.88
1.58
1.44
1.42
1.40
1.26
1.06
1.05
1.03
0.00
23a
1.04
1.00
1.03
2.07
1.16
1.13
2.08
1.04
2.12
2.12
3.06
3.18
10.0 9.5 9.0 8.5 8.0 7.5 7.0 6.5 6.0 5.5 5.0 4.5 4.0 3.5 3.0 2.5 2.0 1.5 1.0 0.5 0.0 -0.5 -1.0
f1 (ppm)

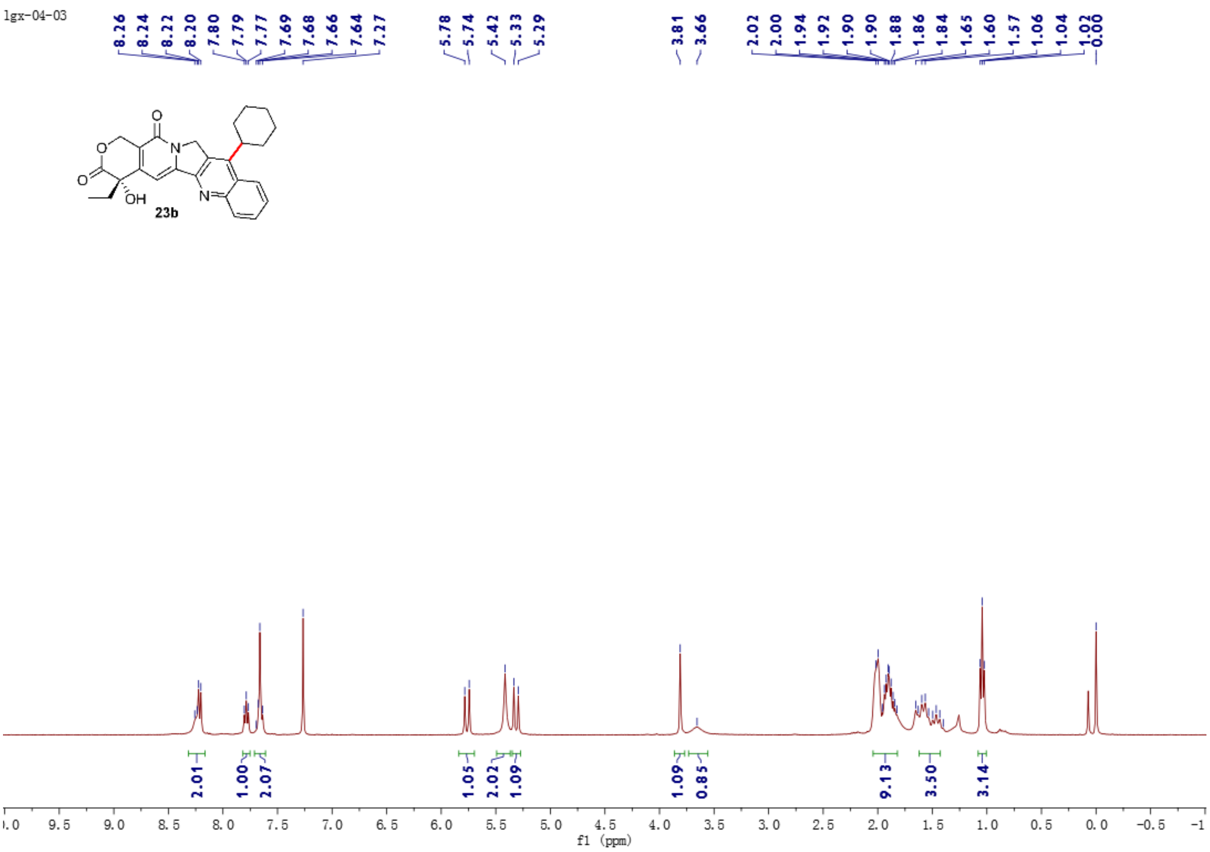
lgx-04-03
8.26 8.24 8.22 8.20 7.80 7.79 7.77 7.69 7.68 7.66 7.64 7.27
5.78 5.74 5.42 5.33 5.29
3.81 3.66
2.02 2.00 1.94 1.92 1.90 1.90 1.88 1.86 1.84 1.65 1.60 1.57 1.06 1.04 1.02 0.00
OH
N
O
23b
2.01 1.00 2.07 1.05 2.02 1.09 1.09 0.85 9.13 3.50 3.14
f1 (ppm)

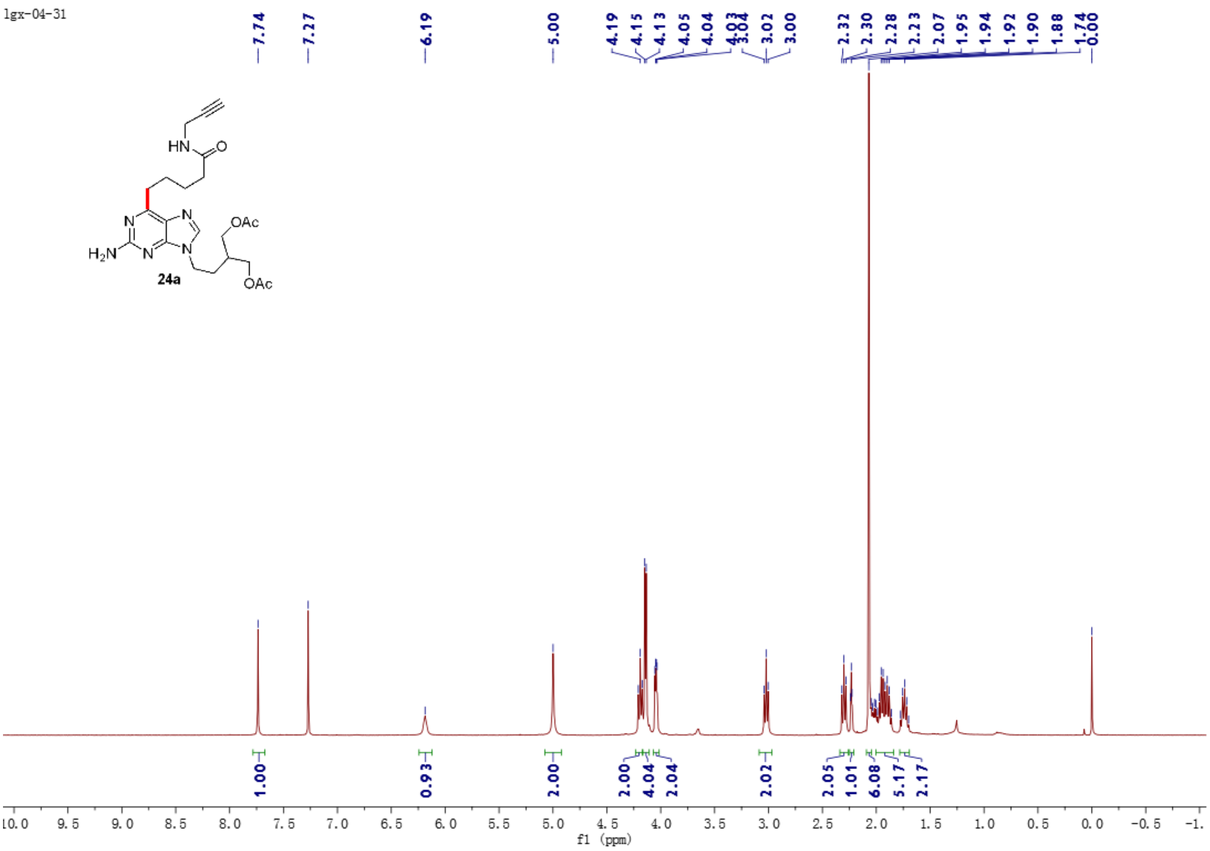
lgx-04-31
7.74 7.27
6.19
5.00
4.19 4.15 4.13 4.05 4.04 4.03 3.04 3.02 3.00
2.32 2.30 2.28 2.23 2.07 1.95 1.94 1.92 1.90 1.88 1.74 0.00
HN
O
OAc
H_2N
24a
1.00 0.93 2.00 2.00 4.04 2.04 2.02 2.05 1.01 6.08 5.17 2.17
f1 (ppm)

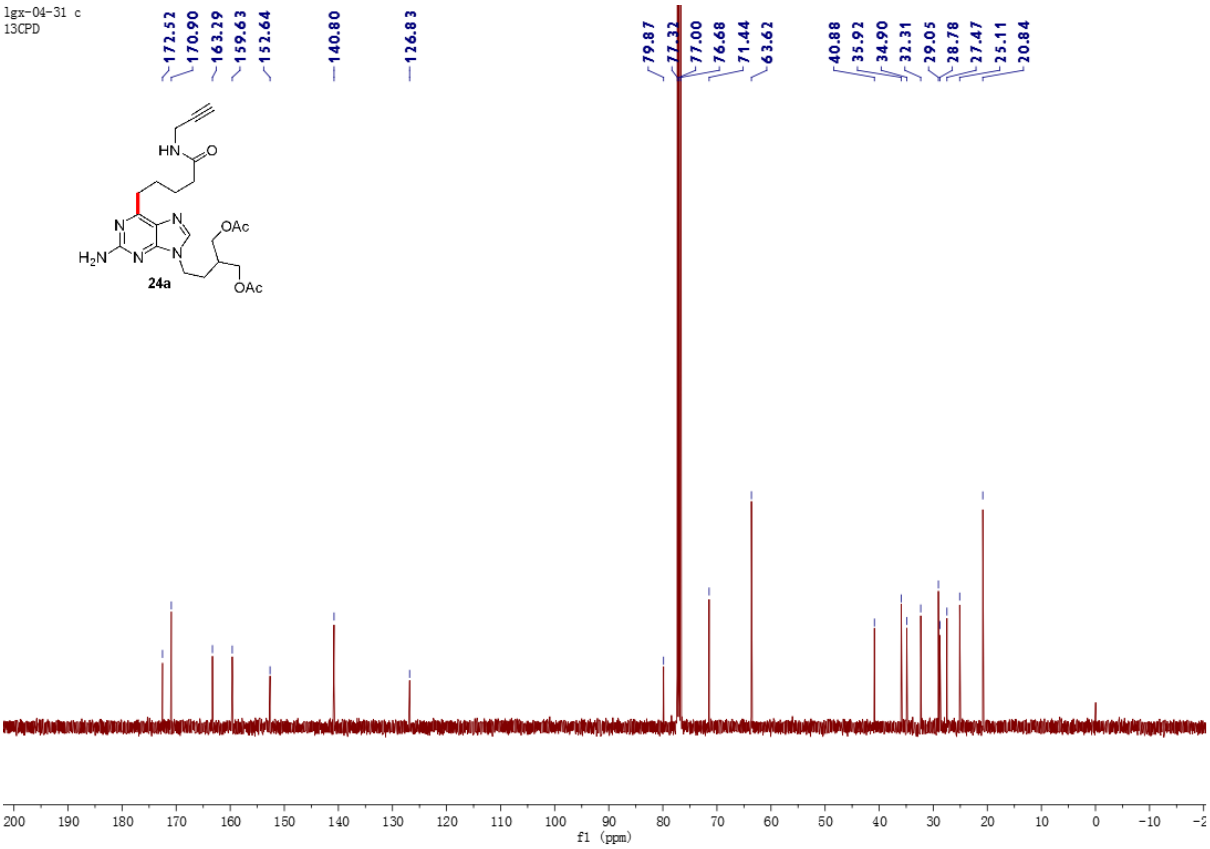
lgx-04-31 c
13CPD
172.52
170.90
163.29
159.63
152.64
140.80
126.83
79.87
77.32
77.00
76.68
71.44
63.62
40.88
35.92
34.90
32.31
29.05
28.78
27.47
25.11
20.84
HN
O
N
N
N
N
H2N
OAc
OAc
24a
f1 (ppm)

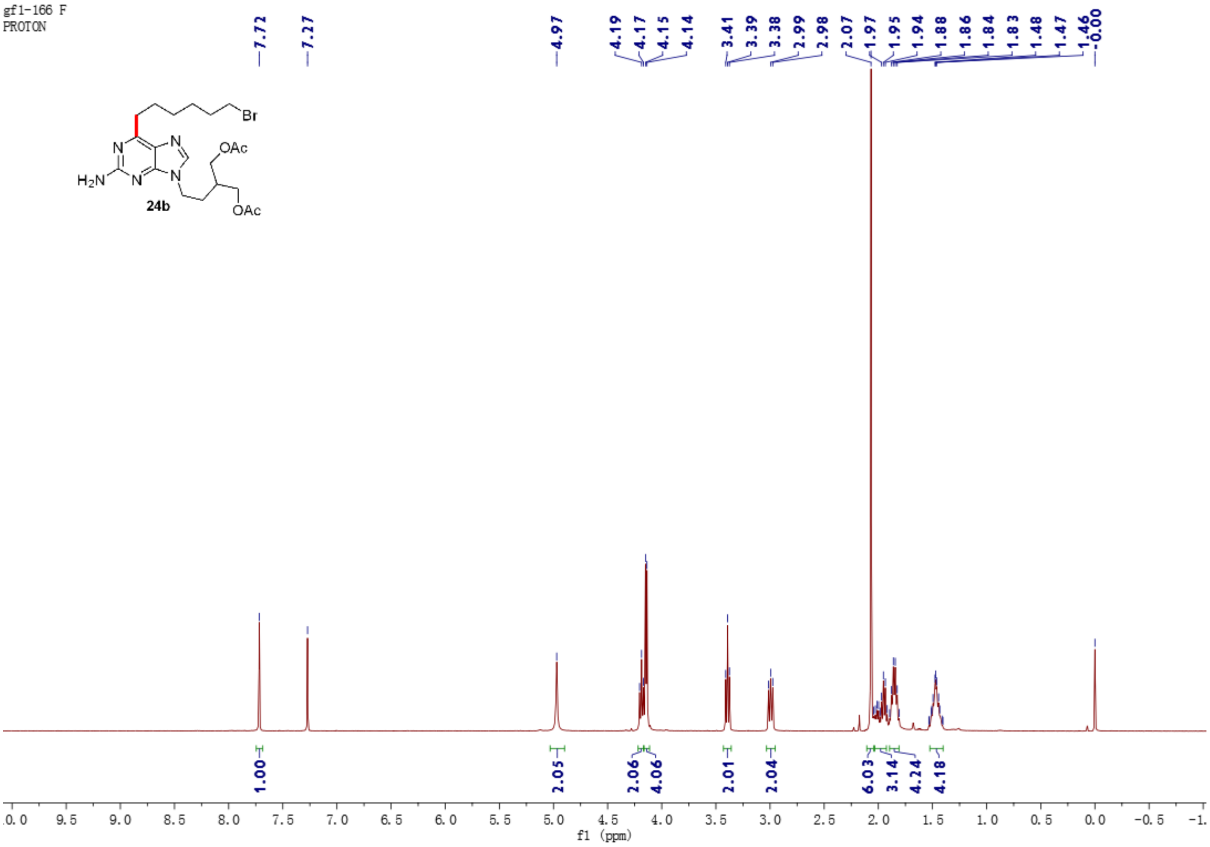
gf1-166 F
PROTON
7.72
7.27
4.97
4.19
4.17
4.15
4.14
3.41
3.39
3.38
2.99
2.98
2.07
1.97
1.95
1.94
1.88
1.86
1.84
1.83
1.48
1.47
1.46
0.00
Br
N
N
N
N
H2N
OAc
OAc
24b
1.00
2.05
2.06
4.06
2.01
2.04
6.03
3.14
4.24
4.18
f1 (ppm)

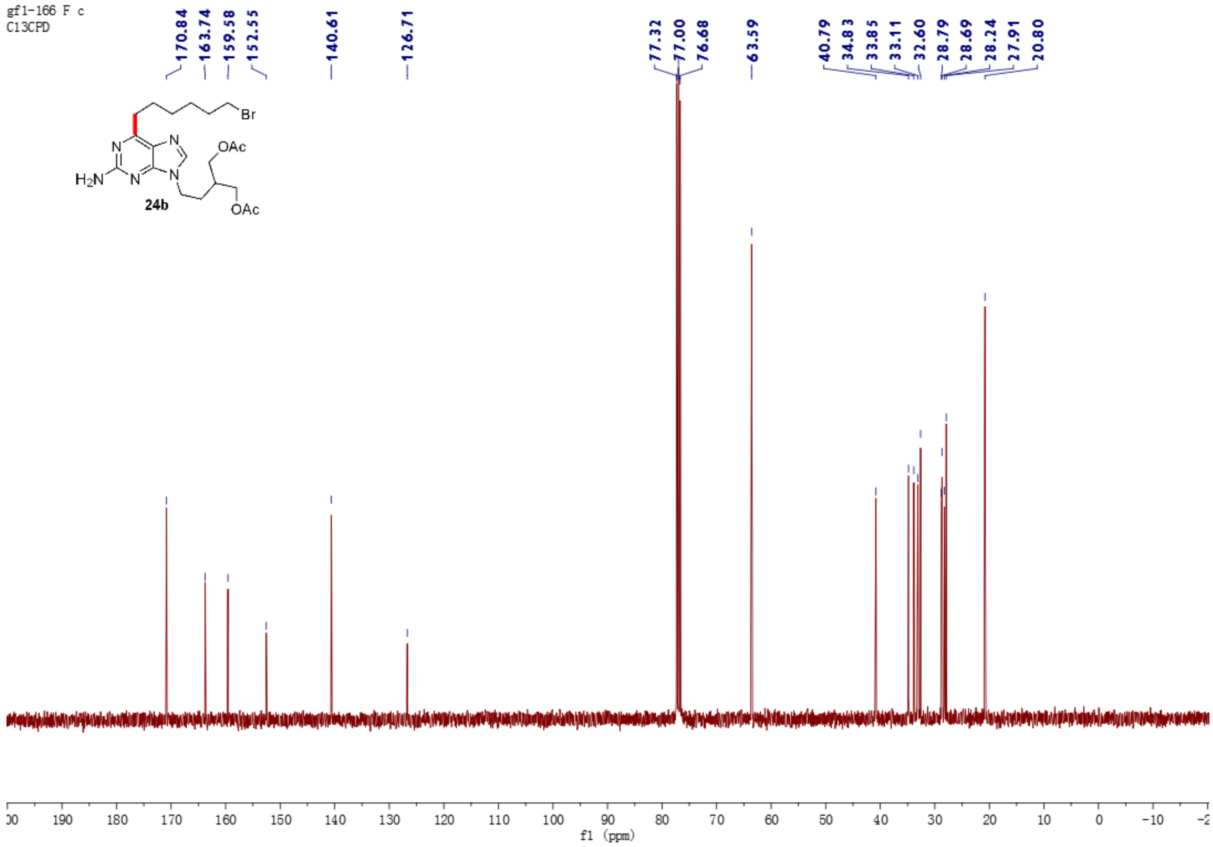
gf1-166 F c
C13CPD
170.84
163.74
159.58
152.55
140.61
126.71
77.32
77.00
76.68
63.59
40.79
34.83
33.85
33.11
32.60
28.79
28.69
28.24
27.91
20.80
Br
OAc
H2N
24b
OAc
f1 (ppm)

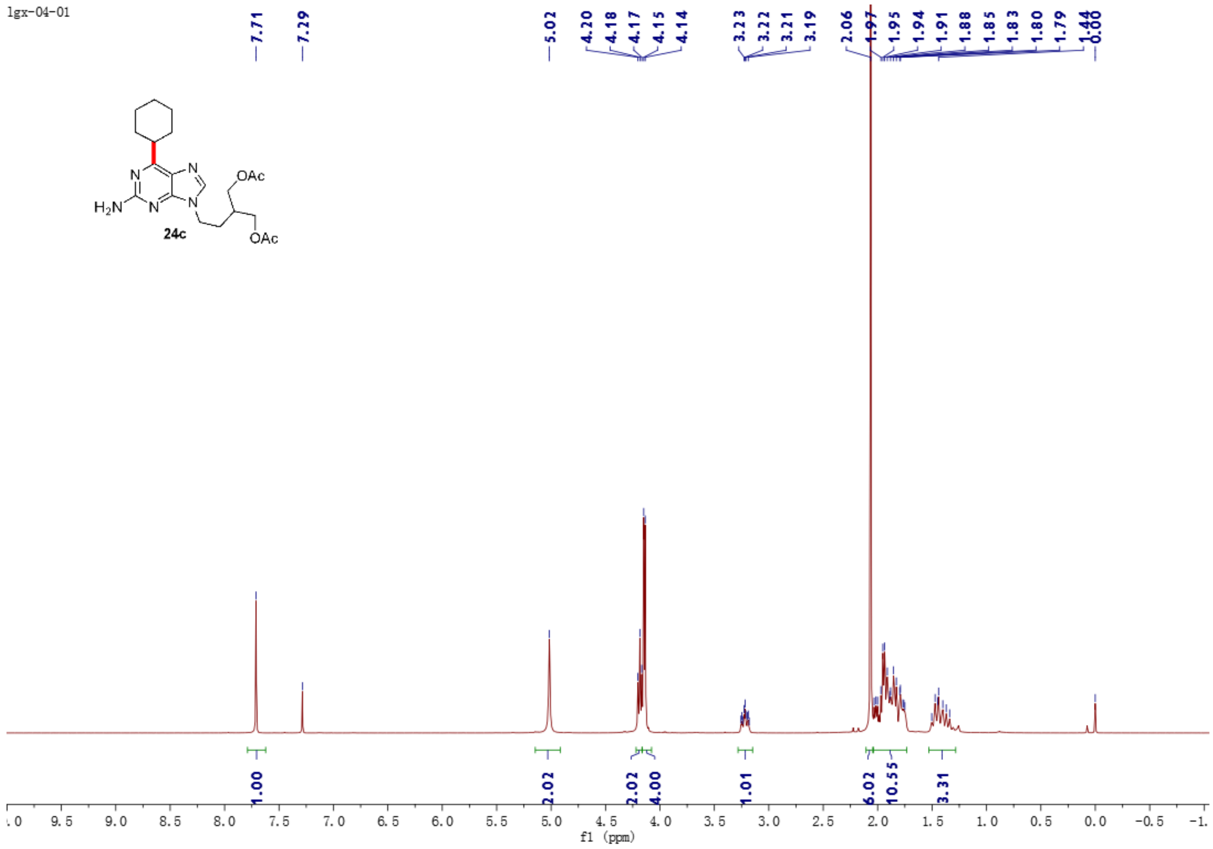
lgx-04-01
7.71
7.29
5.02
4.20
4.18
4.17
4.15
4.14
3.23
3.22
3.21
3.19
2.06
1.97
1.95
1.94
1.91
1.88
1.85
1.83
1.80
1.79
OAc
H2N
24c
OAc
1.00
2.02
2.02
4.00
1.01
6.02
10.55
3.31
f1 (ppm)

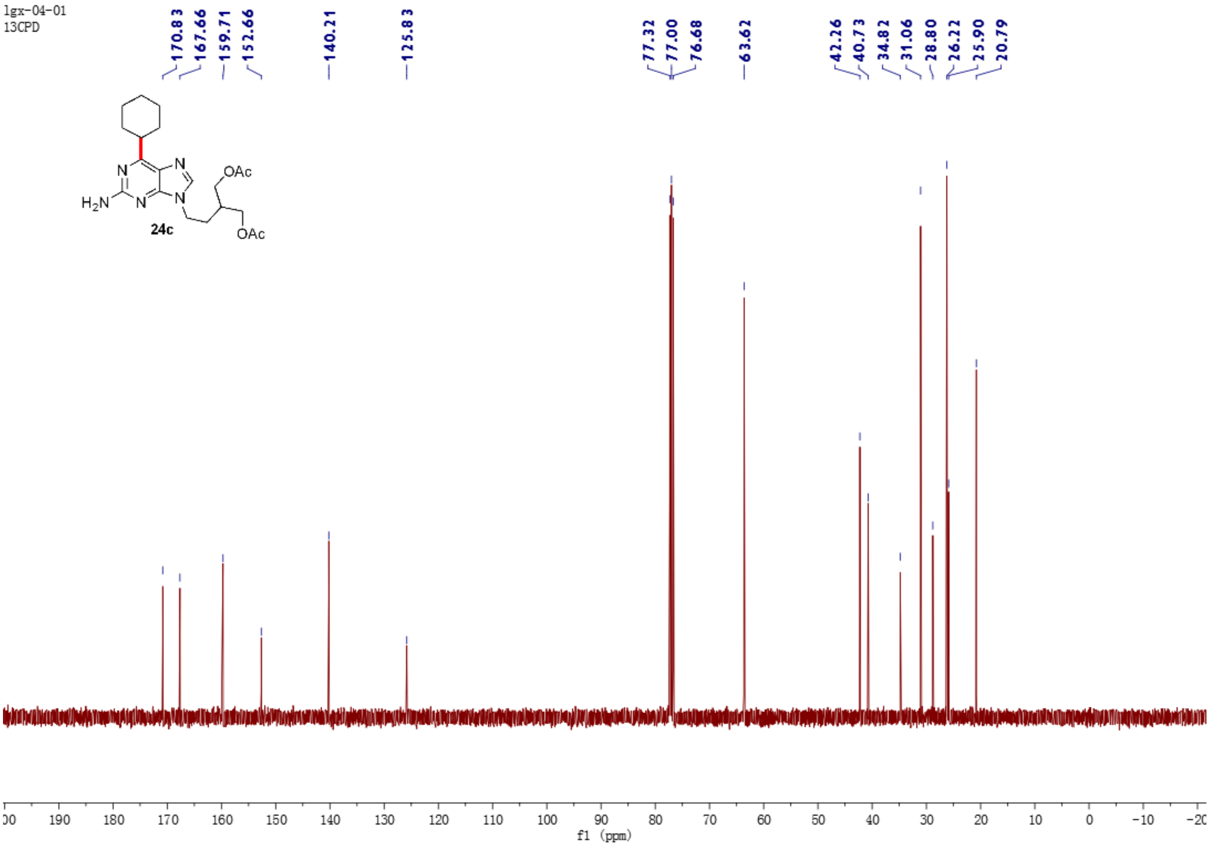

lgx-04-01
13CPD
170.83
167.66
159.71
152.66
140.21
125.83
77.32
77.00
76.68
63.62
42.26
40.73
34.82
31.06
28.80
26.22
25.90
20.79
N
N
N
N
H2N
OAc
OAc
24c
f1 (ppm)

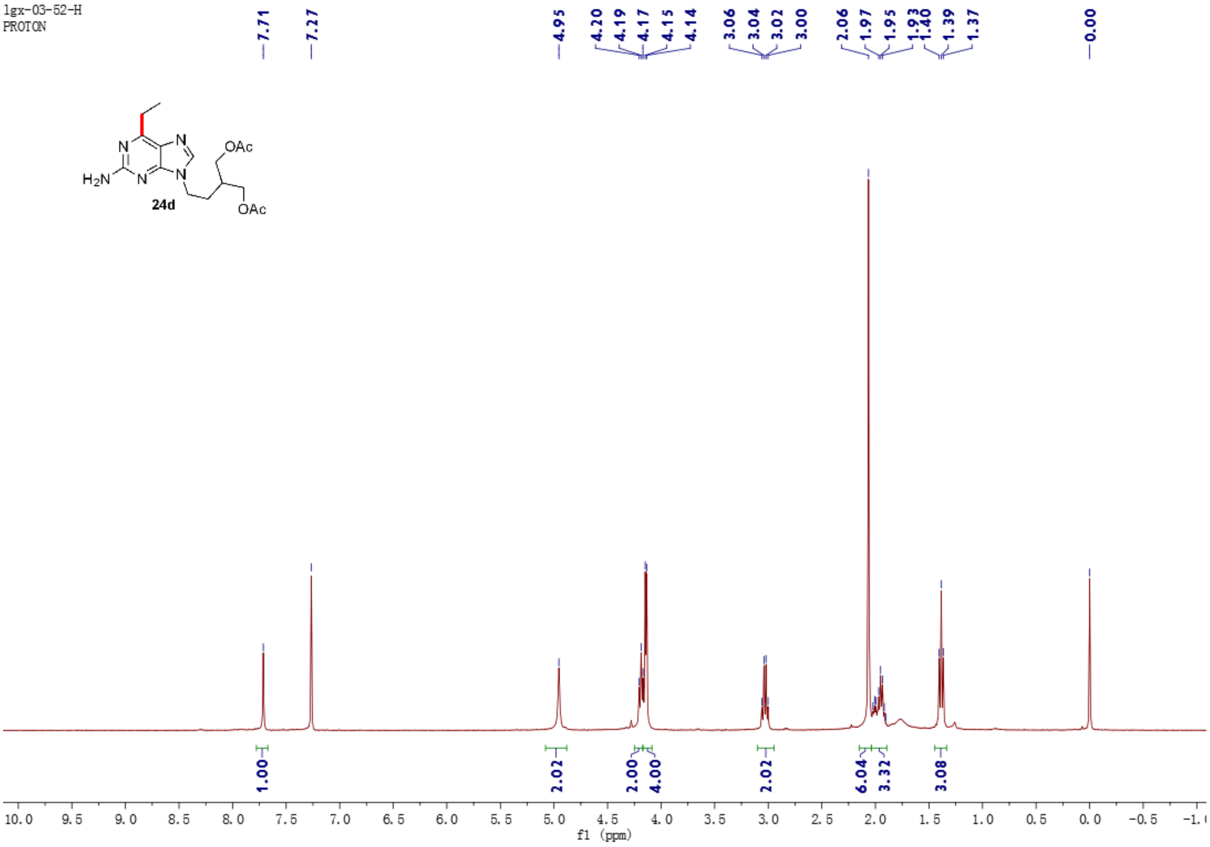

lgx-03-52-H
PROTON
7.71
7.27
4.95
4.20
4.19
4.17
4.15
4.14
3.06
3.04
3.02
3.00
2.06
1.97
1.95
1.93
1.40
1.39
1.37
0.00
N
N
N
N
H2N
OAc
OAc
24d
1.00
2.02
2.00
4.00
2.02
6.04
3.32
3.08
f1 (ppm)

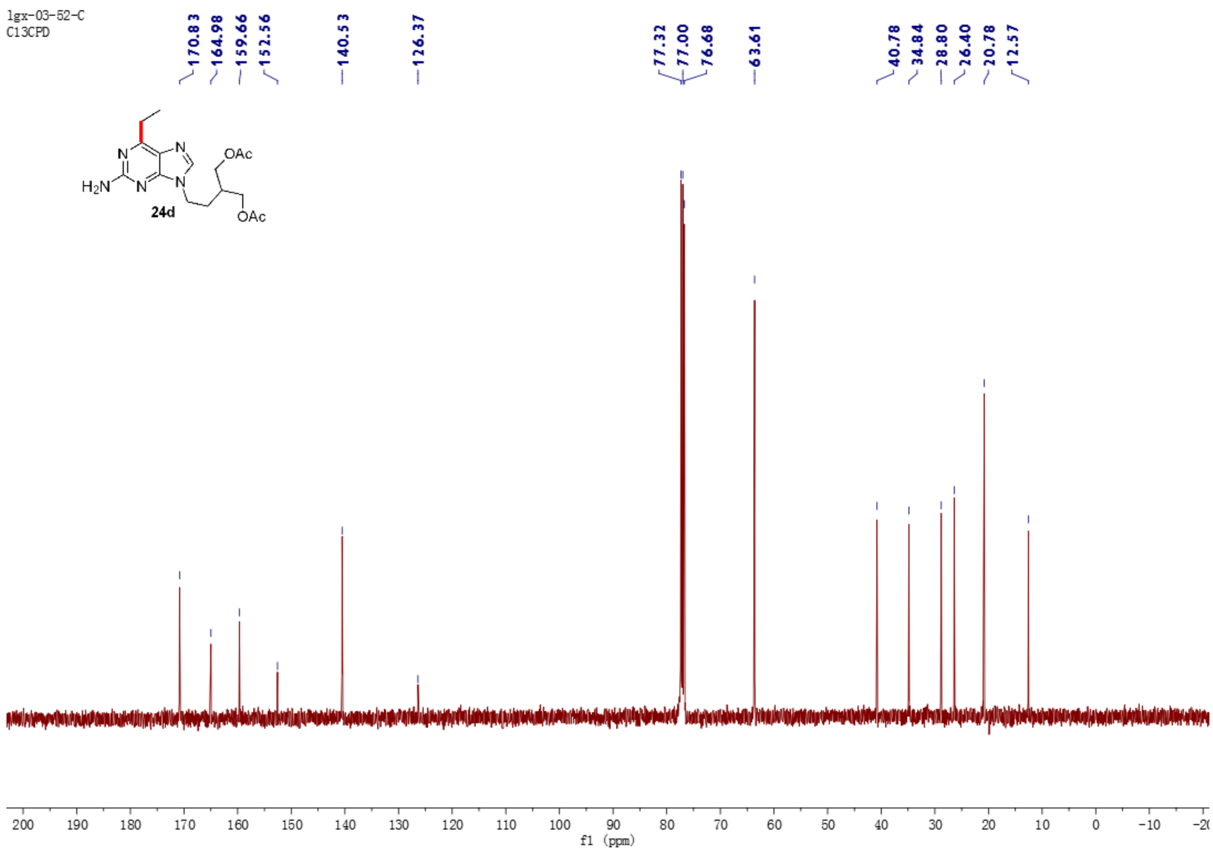
lgx-03-52-C
C13CPD
170.83
164.98
159.66
152.56
140.53
126.37
77.32
77.00
76.68
63.61
40.78
34.84
28.80
26.40
20.78
12.57
N
N
N
N
H2N
OAc
OAc
24d
f1 (ppm)

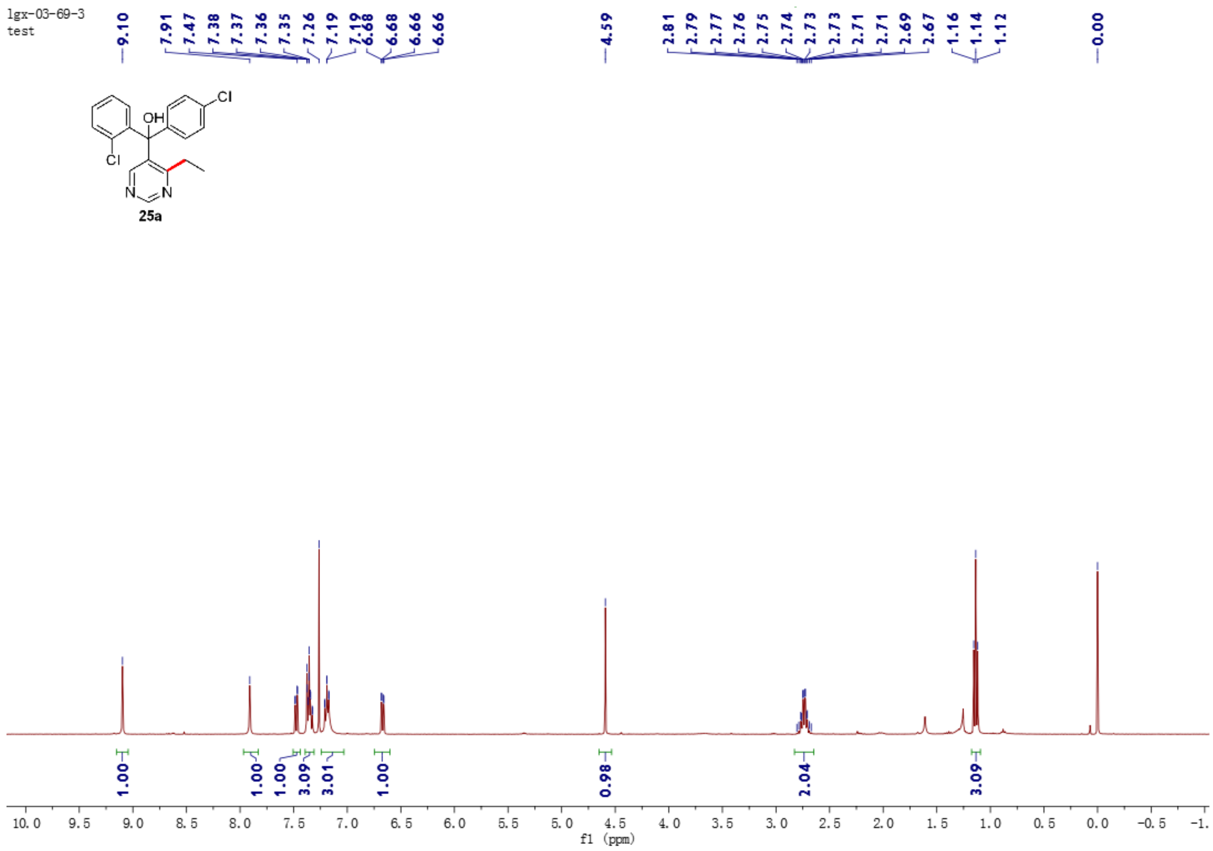
lgx-03-69-3
test
9.10
7.91
7.47
7.38
7.37
7.36
7.35
7.26
7.19
7.19
6.68
6.68
6.66
6.66
4.59
2.81
2.79
2.77
2.76
2.75
2.74
2.73
2.73
2.71
2.71
2.69
2.67
1.16
1.14
1.12
0.00
OH
Cl
Cl
N
N
25a
1.00
1.00
1.00
3.09
3.01
1.00
0.98
2.04
3.09
f1 (ppm)

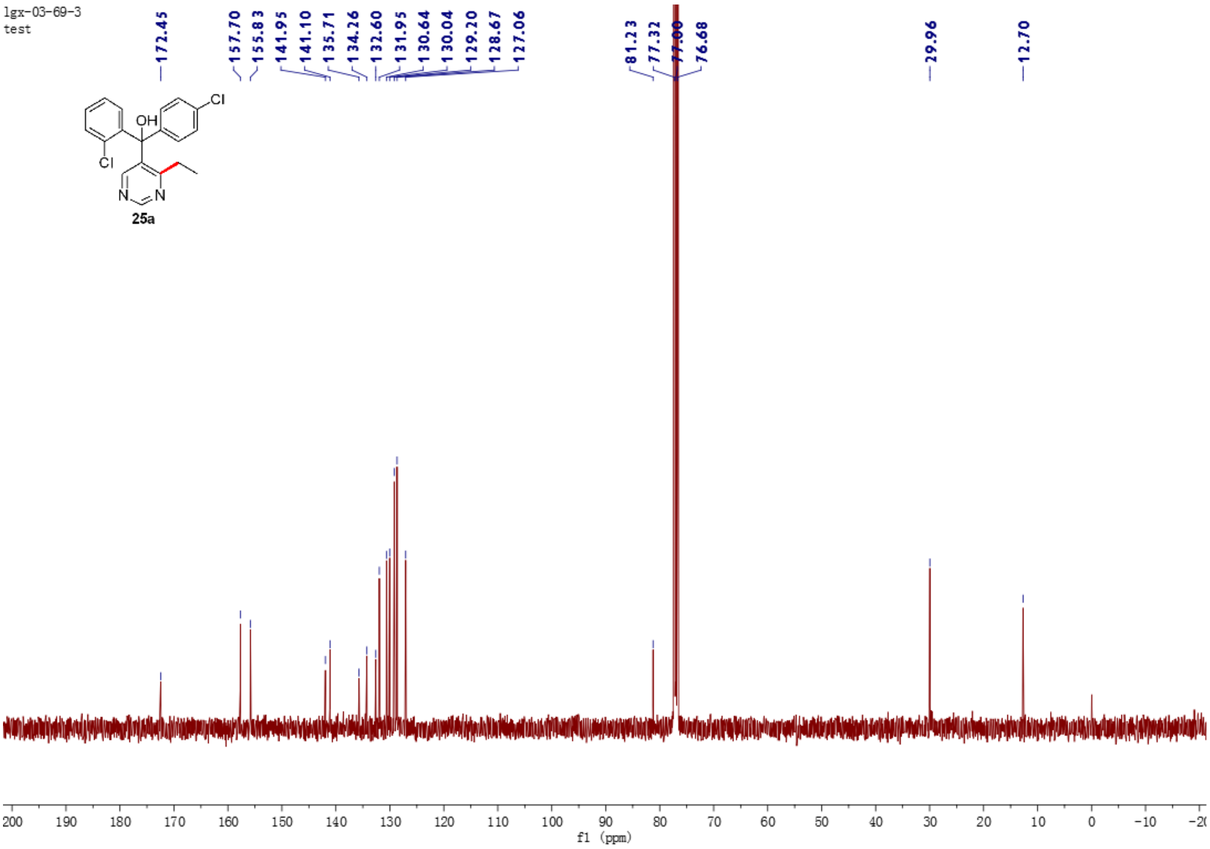
lgx-03-69-3
test
172.45
157.70
155.83
141.95
141.10
135.71
134.26
132.60
131.95
130.64
130.04
129.20
128.67
127.06
81.23
77.32
77.00
76.68
29.96
12.70
OH
Cl
Cl
N
N
25a
f1 (ppm)

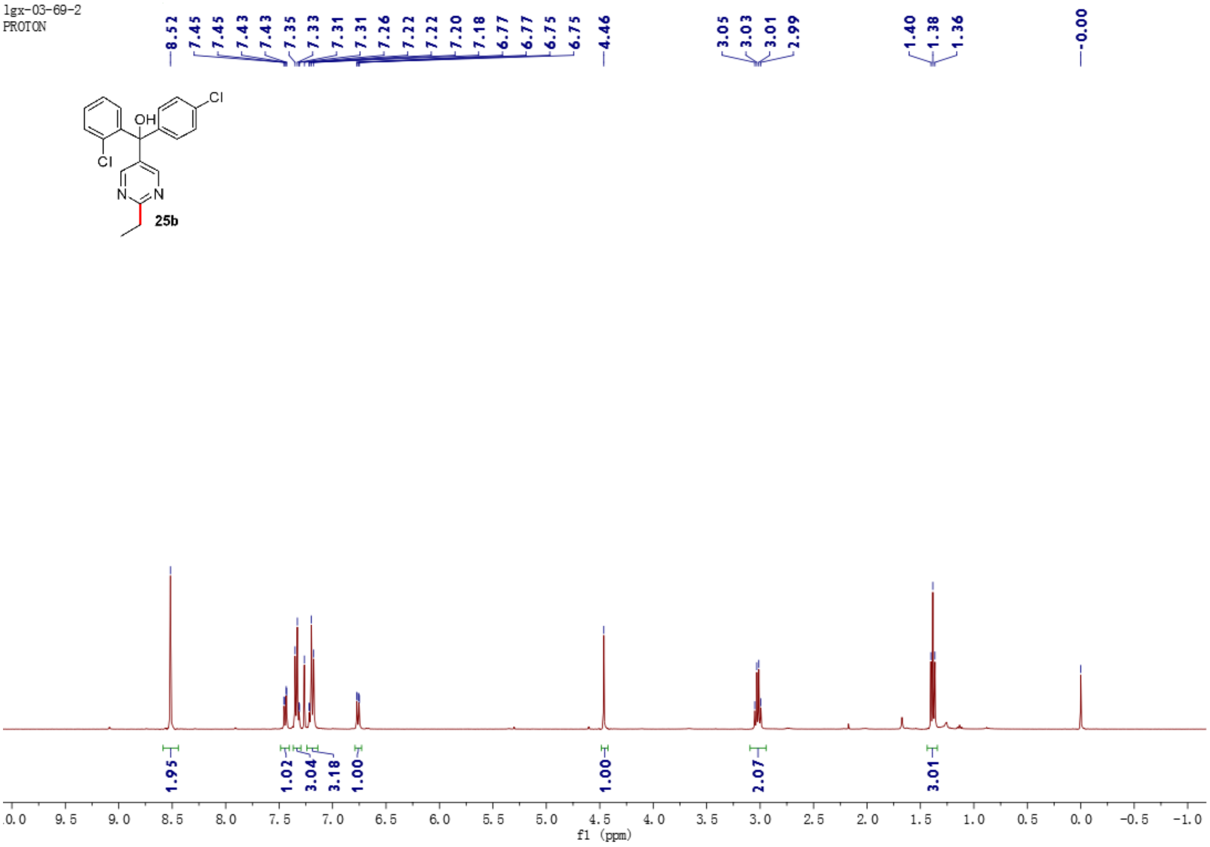
lgx-03-69-2
PROTON
8.52
7.45
7.45
7.43
7.43
7.35
7.33
7.31
7.31
7.26
7.22
7.22
7.20
7.18
6.77
6.77
6.75
6.75
4.46
3.05
3.03
3.01
2.99
1.40
1.38
1.36
-0.00
OH
Cl
Cl
N
N
25b
1.95
1.02
3.04
3.18
1.00
1.00
2.07
3.01
f1 (ppm)

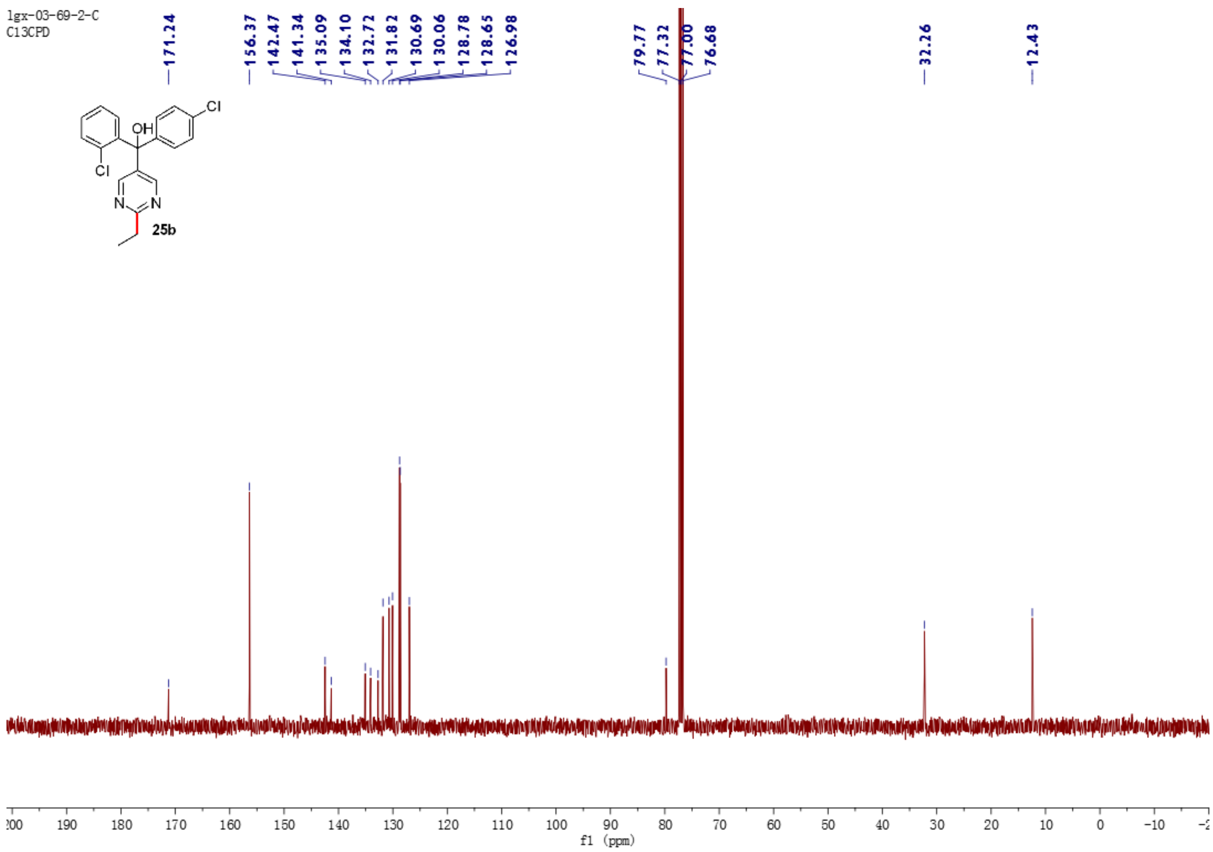
lgx-03-69-2-C
C13CPD
171.24
156.37
142.47
141.34
135.09
134.10
132.72
131.82
130.69
130.06
128.78
128.65
126.98
79.77
77.32
77.00
76.68
32.26
12.43
25b
f1 (ppm)

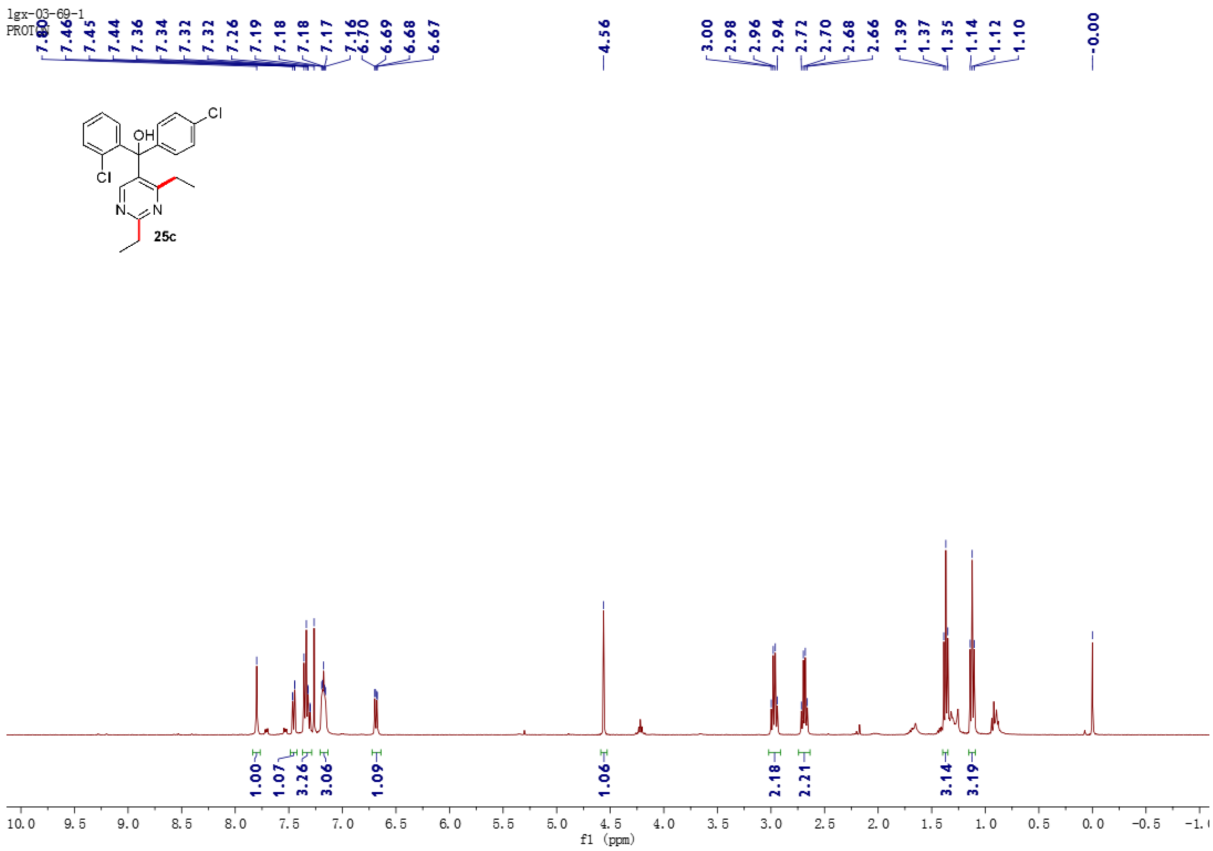
lgx-03-69-1
PROTON
7.46
7.45
7.44
7.36
7.34
7.32
7.32
7.26
7.19
7.18
7.18
7.17
7.16
6.70
6.69
6.68
6.67
4.56
3.00
2.98
2.96
2.94
2.72
2.70
2.68
2.66
1.39
1.37
1.35
1.14
1.12
1.10
-0.00
25c
1.00
1.07
3.26
3.06
1.09
1.06
2.18
2.21
3.14
3.19
f1 (ppm)

lgx-03-69-1-C
C13CPD

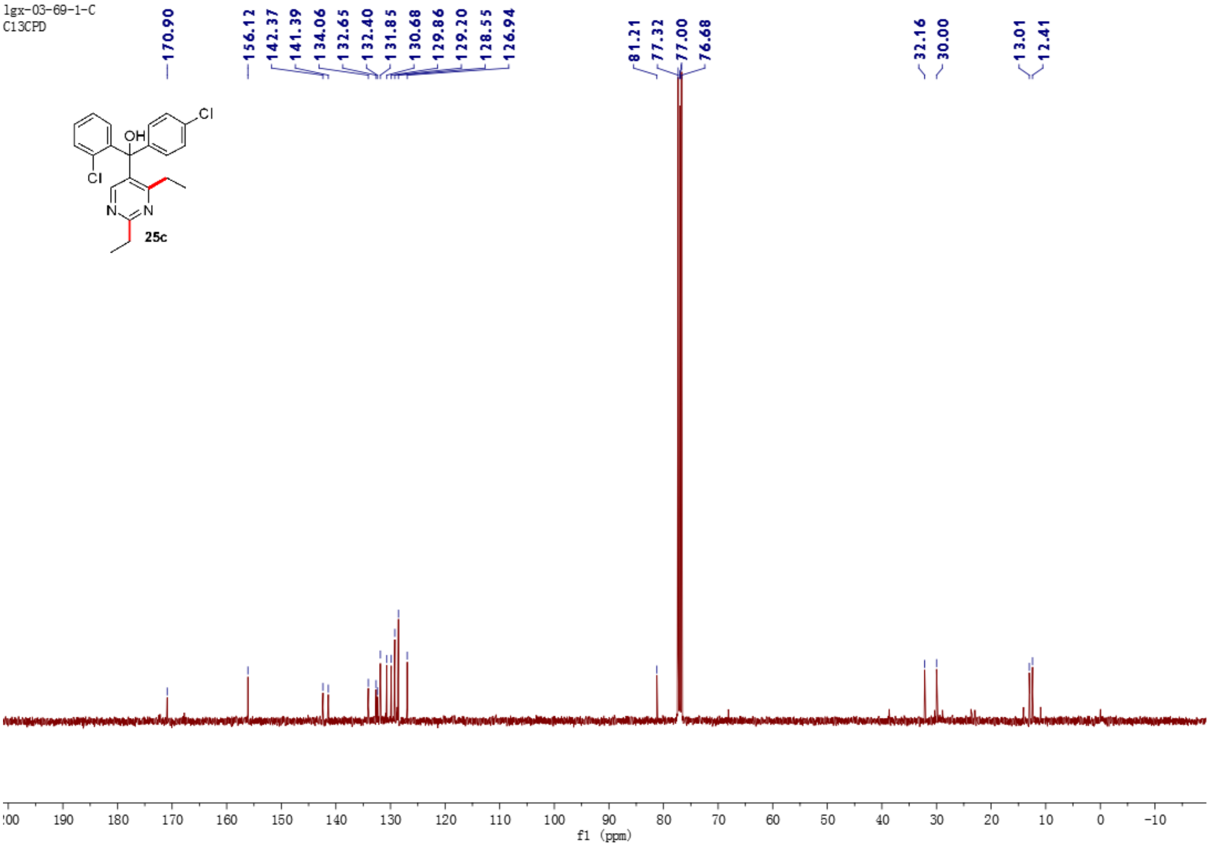

LGX-3-93
PROTON

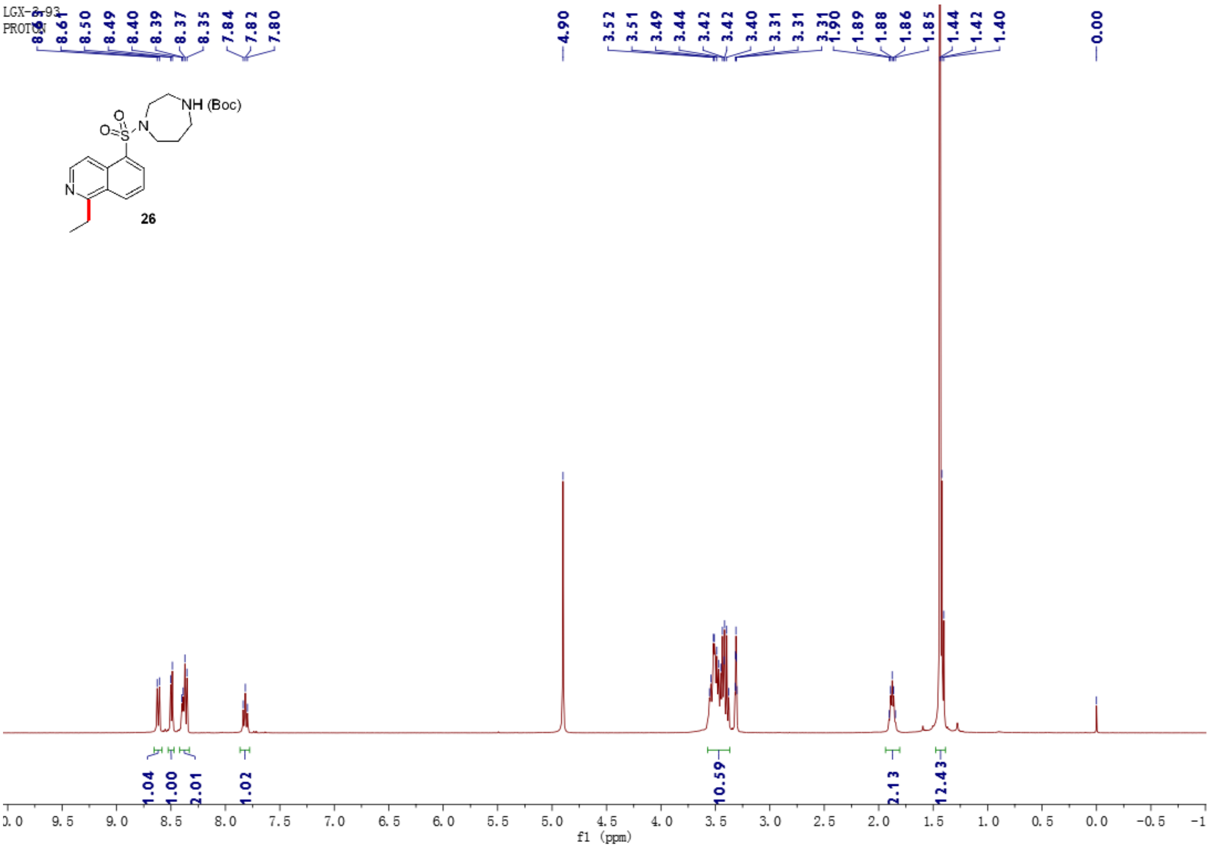

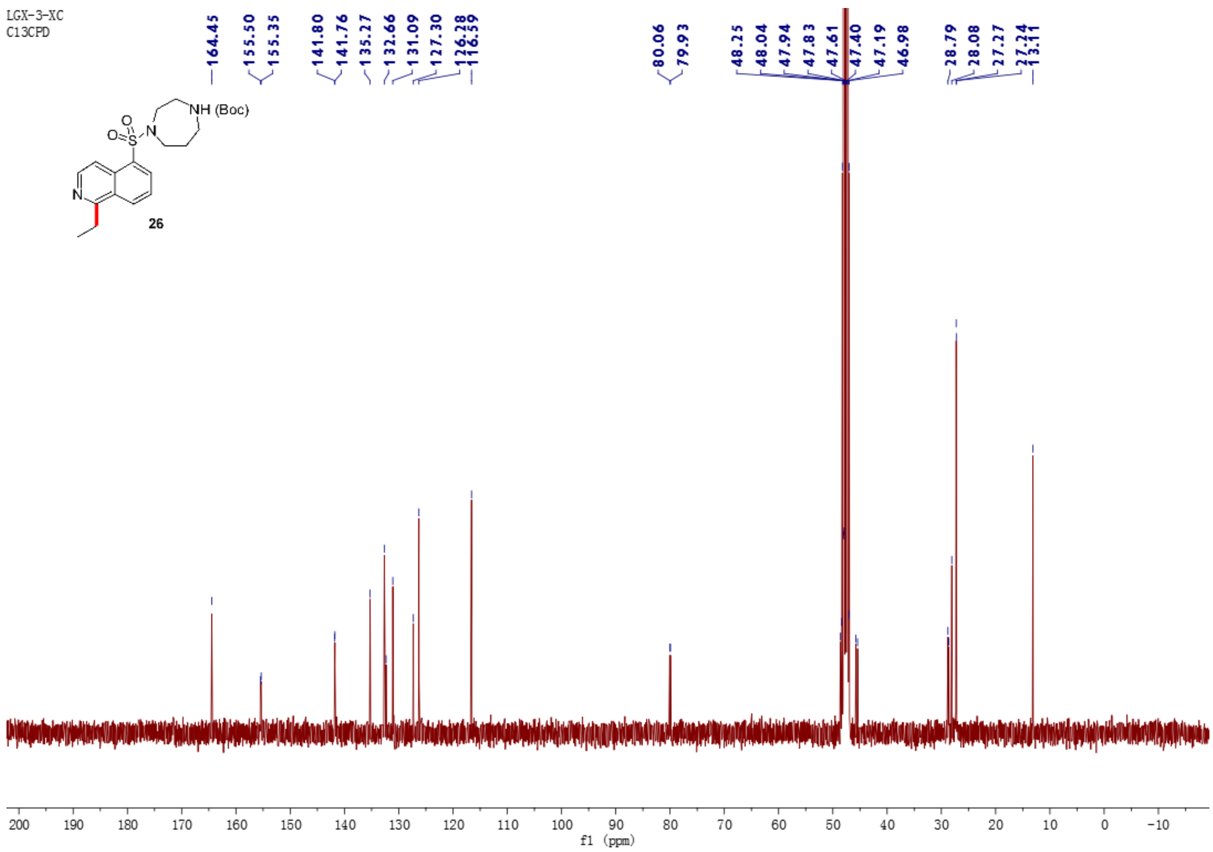
LGX-3-XC
C13CPD
164.45
155.50
155.35
141.80
141.76
135.27
132.66
131.09
127.30
126.28
116.59
80.06
79.93
48.25
48.04
47.94
47.83
47.61
47.40
47.19
46.98
28.79
28.08
27.27
27.24
13.11
NH (Boc)
O
O
S
N
N
26
200
190
180
170
160
150
140
130
120
110
100
90
80
70
60
50
40
30
20
10
0
-10
f1 (ppm)
